# Transcriptome of the Southern Muriqui *Brachyteles arachnoides* (Primates:Platyrrhini), a Critically Endangered New World Monkey: Evidence of Adaptive Evolution

**DOI:** 10.3389/fgene.2020.00831

**Published:** 2020-07-31

**Authors:** Daniel A. Moreira, Alessandra P. Lamarca, Rafael Ferreira Soares, Ana M. A. Coelho, Carolina Furtado, Nicole M. Scherer, Miguel A. M. Moreira, Hector N. Seuánez, Mariana Boroni

**Affiliations:** ^1^Laboratory of Bioinformatics and Computational Biology, Division of Experimental and Translational Research, Brazilian National Cancer Institute (INCA), Rio de Janeiro, Brazil; ^2^Laboratory of Bioinformatics and Molecular Evolution, Department of Genetics, Institute of Biology, Federal University of Rio de Janeiro (UFRJ), Rio de Janeiro, Brazil; ^3^Laboratory for Functional Genomics and Bioinformatics, Oswaldo Cruz Institute, Fiocruz, Rio de Janeiro, Brazil; ^4^Department of Genetics, Institute of Biology, Federal University of Rio de Janeiro (UFRJ), Rio de Janeiro, Brazil; ^5^Genetics Program, Division of Experimental and Translational Research, Brazilian National Cancer Institute (INCA), Rio de Janeiro, Brazil

**Keywords:** RNA-seq, *de novo* transcriptome assembly, primate, positive selection, immune system

## Abstract

The southern muriqui (*Brachyteles arachnoides*) is the largest neotropical primate. This species is endemic to Brazil and is currently critically endangered due to its habitat destruction. The genetic basis underlying adaptive traits of New World monkeys has been a subject of interest to several investigators, with significant concern about genes related to the immune system. In the absence of a reference genome, RNA-seq and *de novo* transcriptome assembly have proved to be valuable genetic procedures for accessing gene sequences and testing evolutionary hypotheses. We present here a first report on the sequencing, assembly, annotation and adaptive selection analysis for thousands of transcripts of *B. arachnoides* from two different samples, corresponding to 13 different blood cells and fibroblasts. We assembled 284,283 transcripts with N50 of 2,940 bp, with a high rate of complete transcripts, with a median high scoring pair coverage of 88.2%, including low expressed transcripts, accounting for 72.3% of complete BUSCOs. We could predict and extract 81,400 coding sequences with 79.8% of significant BLAST hit against the Euarchontoglires SwissProt dataset. Of these 64,929 sequences, 34,084 were considered homologous to Supraprimate proteins, and of the remaining sequences (30,845), 94% were associated with a protein domain or a KEGG Orthology group, indicating potentially novel or specific protein-coding genes of *B. arachnoides*. We use the predicted protein sequences to perform a comparative analysis with 10 other primates. This analysis revealed, for the first time in an Atelid species, an expansion of *APOBEC3G*, extending this knowledge to all NWM families. Using a branch-site model, we searched for evidence of positive selection in 4,533 orthologous sets. This evolutionary analysis revealed 132 amino acid sites in 30 genes potentially evolving under positive selection, shedding light on primate genome evolution. These genes belonged to a wide variety of categories, including those encoding the innate immune system proteins (*APOBEC3G*, *OAS2*, and *CEACAM1*) among others related to the immune response. This work generated a set of thousands of complete sequences that can be used in other studies on molecular evolution and may help to unveil the evolution of primate genes. Still, further functional studies are required to provide an understanding of the underlying evolutionary forces modeling the primate genome.

## Introduction

The southern muriqui (*Brachyteles arachnoides*), also known as the wooly spider monkey, is a neotropical primate species endemic to Brazil and distributed in the states of Paraná, São Paulo, Rio de Janeiro and Minas (Koehler et al., 2005; Talebi and Soares, 2005). The genus *Brachyteles* is composed of two species, *B. arachnoides* and *Brachyteles hypoxanthus* (Chaves et al., 2019). The southern and northern muriquis are the largest neotropical primates, with males attaining 55-78 cm of head-body length, 74–80 cm of tail length, and weighing around 8–11 kg. With long, thin arms and legs, and thumbless hands, muriquis sway in trees hanging only by their prehensile tails (Talebi and Lee, 2010). They are mainly folivorous, though they may also feed on flowers, bark, bamboo, ferns, nectar, pollen, and seeds (Talebi et al., 2005). Muriquis are diurnal primates living in groups of 4–43 individuals at altitudes ranging from sea level up to 2,000 m above sea level. Both muriqui species are currently critically endangered, primarily due to their habitat destruction (Talebi et al., 2019), and the *B. arachnoides* population is estimated at only 1,100-1,200 individuals (Strier et al., 2017).

Phylogenetic reconstructions based on molecular data consistently places the genus *Brachyteles* in the Atelidae family (Schneider et al., 1993, 2001; Perelman et al., 2011; Di Fiore et al., 2015; Dumas and Mazzoleni, 2017), within which its relative position to other genera is established as {*Alouatta* [*Ateles* (*Brachyteles*, *Lagothrix*)]}. Divergences from the remaining neotropical families Cebidae and Pitheciidae are dated between 20–23 mya and 22–26, respectively. While relationship between the three families was previously disputed, multiple works in the last decade have clarified this conflict as caused by incomplete lineage sorting events. Karyotypic analyses with chromosome banding shows a diploid number (2n) of 62 chromosomes and comparative painting with whole chromosome probes revealed several interspecific chromosome homologies between *Brachyteles*, the human, and other non-human primates, mainly *Lagothrix*, a taxon sharing a very similar karyotype (de Oliveira et al., 2005). Interestingly, *Brachyteles* and *Lagothrix* show the highest diploid chromosome count among neotropical primates (2*n* = 62).

In the last two decades the genetic basis underlying adaptive traits of New World monkeys (parvorder Platyrrhini) has been a subject of interest to several investigators (Ribeiro et al., 2005a; Vargas-Pinilla et al., 2015; Fam et al., 2019). There has been a significant concern about genes related to the immune system (Soares et al., 2010; Darc et al., 2012), which have shown higher evolution rates in Platyrrhines than in other clades (Ribeiro et al., 2005b). The coevolution between pathogens and host defensive systems may, eventually, result in zoonotic transmissions from mammals to humans, thus heightening public health concern, as seen in the recent coronavirus pandemic outbreak (Olival et al., 2017; Kaján et al., 2020; Perlman, 2020).

High throughput sequencing technologies have flourished this past decade in the field of primate genomes, scanning thousands of genes (Nielsen et al., 2005; Kosiol et al., 2008; George et al., 2011). RNA-sequencing requires less sequencing effort than whole genome sequencing for acquiring desirable coverage and depth of genes, since less than 2% of the human genome codes for proteins (International Human Genome Sequencing Consortium, 2004). This makes *de novo* assembled transcriptome a powerful approach for identifying positively selected genes and testing evolutionary hypotheses, particularly when dealing with a non-model species for which a reference genome is not available (Stapley et al., 2010; Perry et al., 2012; Yang et al., 2014; Lan et al., 2018; Bentz et al., 2019).

Here we report for the first time the assembly, annotation and expression profile of *B. arachnoides* transcriptome from two different cell samples. Ortholog sequences from 10 other primates were retrieved and compared. Subsequently, a branch-site model analysis identified genes under positive selection in *B. arachnoides*, revealing positive selection at 132 amino acid sites in 30 genes, with several sites located in genes related to the immune response. We also showed an expansion on *APOBEC3G* increasing the knowledge of retrocopy gene birth to an Atelid species.

## Materials and Methods

### Sample Collection and Ethical Considerations

Peripheral blood samples and a skin biopsy were collected from a captive male *B. arachnoides* (CPRJ2506) kept in the Centro de Primatologia do Rio de Janeiro (CPRJ-INEA; see Schrago et al., 2012), where blood samples were regularly collected for checkups and control of captive animals. Since this species is critically endangered, minor invasive methods (blood sample and skin biopsy) were adopted minimizing the risk for the specimen. Sample collection was carried out according to the IBAMA (Instituto Brasileiro do Meio Ambiente e dos Recursos Naturais Renováveis, Brazil; permanent license number 111375-1) national guidelines and provisions. This license was granted by IBAMA following the approval of its Ethics Committee.

### Establishment of Cell Cultures From Skin Biopsy

A skin biopsy (0.5 cm × 0.5 cm), aseptically collected from the animal, was minced into small fragments of approximately 1 mm^2^ and digested with 10 mL aliquots of a trypsin-collagenase solution prepared with 500 ml of RPMI medium, 2.5 g of collagenase, 50 mL of 2.5% trypsin, and 5 mL of each of the following antimicrobial agents: gentamicin, fungizone, penicillin-streptomycin and mycostatin, and HEPES buffer to a final concentration of 25 mM. Fragments were digested under continuous agitation using a magnetic stir plate. At 15 m intervals, supernatants were collected, mixed with Dulbecco MEM and 10% fetal calf serum, centrifuged at 500 *g*. Pellets were suspended in Dulbecco MEM medium with 10% fetal calf serum and incubated in flasks at 37°C and 5% CO_2_. This procedure was repeated by adding fresh solution to remaining fragments until all were digested. Confluent flasks were harvested and cell pellets stored at −20°C for nucleic acid isolation.

### RNA Isolation, Preparation of Transcriptomic Libraries and Sequencing

Total RNA was isolated from blood cells and fibroblast cell culture with RNeasy^®^ (Qiagen). RNA was subsequently quantified with a NanoDrop spectrophotometer (Thermo Scientific). Quality and integrity were checked by electrophoresis in 1% agarose gels. Two aliquots (4 μg) of total RNA (one from blood and another one from fibroblasts) were used for preparing 300–400 bp cDNA libraries with TruSeq RNA Sample Prep Kit v2^®^ (Illumina) following the protocol recommended by the manufacturer. Library size was checked with an Agilent Bioanalyzer 2100 DNA using a high sensitivity DNA chip. Library concentrations were estimated by qPCR using a Library Quantification kit (KAPA – KK4824). Two single-end runs from blood and three paired-end runs from fibroblasts were carried out in an Illumina HiSeq 2500 platform generating a total of 350 million reads.

### Data Preprocessing, Transcriptome Assembly and Annotation

Adapter sequences were removed from raw reads with Cutadapt v1.14 (Martin, 2011). Low quality reads and poly-A tails were trimmed with Trimmomatic v0.38 (Bolger et al., 2014) with the following parameters: *leading:20 trailing:20 slidingwindow:4:20 minlen:36* and Prinseq v0.20.4 (Schmieder and Edwards, 2011) with the following parameters: *trim_tail_left 7 trim_tail_right 7 min_len 36*. Following this preprocessing step, data quality was assessed with FastQC v0.11.5. In order to improve assembly efficiency, the dataset was *in silico* normalized using a perl script built-in Trinity software following best practices for *de novo* assembly using Trinity. This normalization step reduced the quantity of input reads to be assembled, while maintaining transcriptome complexity and capability for full-length transcript reconstruction (Haas et al., 2013).

All five runs were combined and used for *de novo* assembly with Trinity v2.8.4 (Grabherr et al., 2011; Haas et al., 2013) in three different runs, altering *–min_kmer_cov*, which stands for the minimal count for *K*-mers to be assembled by Inchworm (parameters: *–no_normalize_reads –run_as_paired –min_kmer_cov 2/3/5*). The three assemblies were evaluated with Detonate software v1.11 RSEM-EVAL built-in package (Li et al., 2014). This package combined multiple factors into a single score, including the compactness of assemblies and the read support. The assembly with the highest score was selected for subsequent analyses.

The completeness of the transcriptome was evaluated by a quantitative assessment of transcriptome assembly provided by BUSCO v3 (Simão et al., 2015). The number of full-length assembled transcripts was evaluated using BLASTX (SwissProt database for *Homo sapiens*, *e*-value < 1*e*-20 (Altschul et al., 1990) and examining the percentage of the target aligned to the best matching transcript with a perl script built-in Trinity software.

In order to decrease the number of fragmented transcripts, the transcriptome was filtered for assembled transcripts with at least 300 nt. Subsequently, Transdecoder v5.5 was used for identifying candidate coding regions (CDS) within assembled transcript sequences. In order to functionally annotate these potential proteins, all predicted CDS were searched in SwissProt (Euarchontoglires), eggNOG 5.0, KEGG, and Pfam public databases, using BLASTP (best top hit, *e*-value < 1*e*-10), eggNOG-mapper (min. hit *e*-value: 0.001, min. hit bit-score: 60, min.% of query cov.: 20) (Huerta-Cepas et al., 2017, 2019) and HMMER v3.1b2.

The transcript expression level of each cell sample was estimated by RSEM v1.2.28 (Li and Dewey, 2011) after aligning reads to the transcriptome with Bowtie2 v2.3.4.3 (Langmead and Salzberg, 2012). Transcripts with a Transcripts Per Million (TPM) ≥ 1 were used to compare the expression profiles of the two RNAs, and this comparison was visualized with Venn diagrams. Predicted CDS were annotated using the top hit of comparisons with the SwissProt dataset for Supraprimates (Euarchontoglires) using BLASTP (*e*-value < 1*e*-10). The 500 transcripts with the highest TPM estimates of each cell sample and with alignment coverage >80% of high scoring pair (HSP) length were used for pathway enrichment analysis (Over-Representation Analysis – ORA) with the Reactome database using the WebGestalt web tool (Wang et al., 2017). In order to get a better sense of the richness of the blood sample, we run the CIBERSORTx on a dataset containing TPM values of transcripts that had a significant BLAST hit against a Euarchontoglires protein and showed identity and HSP coverage over 60%. The CIBERSORTx was performed using a validated leukocyte gene signature matrix that contains 547 genes distinguishing 22 human hematopoietic cell phenotypes (Newman et al., 2019).

### Adaptive Selection Analysis

Predicted coding sequences with alignment coverage of HSP length > 80% were further used for positive selection analysis and only isoforms with the highest TPM value were kept for downstream analysis. In order to represent the evolutionary dynamics within the neotropical primates lineage, we selected the best-annotated genomes available for this group for the selection tests; namely *Callithrix jacchus* (Ensembl release 96), *Saimiri boliviensis* (Ensembl release 96), and the outgroup *Homo sapiens* (Ensembl release 96). Orthologs were then obtained by best reciprocal hit against predicted coding sequences. Alignments between the orthologous CDS identified were subsequently converted to amino acids, aligned using MAFFT v7.4.27 (Katoh and Standley, 2013), and converted back to nucleotides. Terminal stop codons were also removed from alignments. Reference topology for all subsequent analyses was generated by retaining only the taxa of interest in the primate phylogeny reconstructed by Perelman et al. (2011).

With the purpose of reducing the number of false-positives, we tested orthologs for positive selection using three independent validation processes. Firstly, the aBSREL model (Smith et al., 2015) available in the HyPhy 12.14 software package (Pond and Muse, 2005) was used for testing the four species for branch-site positive selection. Branches were considered under selection when the model of multiple ω rate classes along the sequence alignment was the best fit (Likelihood Ratio Test, adjusted *p*-value < 0.05). Findings were then confirmed by testing the predicted CDS for positive selection with the BUSTED model (Murrell et al., 2015) in HyPhy, which attributed three rate classes, one with ω ≥ 1 and two other with ω ≤ 1. The model fit was tested against the null hypothesis with a higher ω fixed to 1 (LRT, adjusted *p*-value < 0.05). Lastly, CDS showing positive selection in both tests were thereafter analyzed with a higher number of taxonomic units (up to 11 species depending on the availability of ortholog sequence in datasets). These included the primate species *Aotus nancymaae*, *Cebus capucinus*, *Chlorocebus sabaeus*, *Macaca nemestrina*, *Otolemur garnettii*, *Papio anubis*, and *Pongo abelii*. Because alignment errors highly increase the false-positive rate of the branch-site test (Fletcher and Yang, 2010), we manually verified these sequence alignments, discarding misaligned regions and subsequently used the branch-site test of CODEML built-in the PAML 4 package (Yang, 2007).

The genes identified as potentially evolving under positive selection were associated with Gene Ontology (GO) terms, Reactome and Panther pathways^1^ (accessed: 14 October 2019). We also identified pervasive selection by comparing the synonymous and non-synonymous rates using the fixed effects likelihood test (FEL) (Kosakovsky Pond and Frost, 2005). In this last analysis, we only used the alignments with 11 species that returned positive to all selection tests. Finally, we reconstructed the evolutionary trees of the final positively selected CDS using IQTREE 1.7 software (Nguyen et al., 2015) with 1000 bootstrap replicates. Evolutionary models were selected employing the Model Finder algorithm available with IQTREE. Trees were rooted on the outgroup species *Otolemur garnetti* or, when orthologous sequence for this species was not available, on the diversification between New World and Old World primates.

### Comparative Genomic Analysis and Orthogroup Identification

For the comparative analysis, we downloaded the proteomes of the same 10 primate species used in adaptive selection analysis (Ensembl release 100). To increase accuracy of the orthology assignment, we used a script provided with OrthoFinder to extract just the longest transcript variant per gene. In the same way, to avoid misassembled transcripts and reduce redundancy, we used only peptide sequences predicted from transcripts with TPM > 1 and we ran CD-HIT (Fu et al., 2012) to cluster similar sequences with a threshold of 95%. The identification of orthogroups and the comparative analysis were performed by OrthoFinder v2 (Emms and Kelly, 2019). Briefly, OrthoFinder analysis consists of an all-to-all BLASTP step and clustering step using MCL algorithm to build the orthogroups.

The sequences of *APOBEC3G* identified by the OrthoFinder were aligned using Clustal Omega (Thompson et al., 1994) and the maximum likelihood tree was constructed using PHYML (Guindon et al., 2010) with NNIs as tree topology search, BioNJ initial tree, Blosum62 + GAMMA amino acid substitution model and 100 bootstraps. Tree was rooted using human and NWM *APOBEC3A* sequences.

### Molecular Modeling

In order to understand whether the positive selection on gene sequences affects their structure, we build and compare 3D models for Human and *B. arachnoides* of APOBEC3G and CEACAM1 proteins using homology modeling approach. BLASTP algorithm (Altschul et al., 1990), with default settings, was used to obtain the two template structures from protein data bank. The structure of APOBEC3G of *Macaca mulatta* under PDB ID 6P3X (Yang H. et al., 2020) was used as a template for modeling the APOBEC sequences of the *H. sapiens* and *B. arachnoides*, and the structure CEA (expressed by *CEACAM5* gene) from *H. sapiens* under PDB ID 1E07 (Boehm and Perkins, 2000) was used to build the CEACAM1 proteins for *H. sapiens* and *B. arachnoides*.

All models were built using the software Modeller v9.24 (Webb and Sali, 2016) and the primary structures of templates and models were aligned using the web server Clustal Omega (Madeira et al., 2019). One thousand models were generated for each model, giving a total of four thousand structures, the models with the best objective function value were chosen. For APOBEC3G, the best models were 359 and 823 for *H. sapiens* and *B. arachnoides*, respectively. And the best models for CEACAM1 were 326 and 158 for *H. sapiens* and *B. arachnoides*, respectively. The electrostatic profile of the protein models were calculated by APBS and PDB2PQR (Baker et al., 2001; Dolinsky et al., 2007; Unni et al., 2011) and rendered on Pymol v2.3 (DeLano, 2002).

## Results

### *De novo* Transcriptome Assembly and Quality Assessment

More than 350 million reads of blood and fibroblast transcripts of *B. arachnoides* were generated by the Illumina HiSeq 2500 platform (Supplementary Material 1). Following adapter removal and low-quality base trimming, 315,430,518 (89.26%) reads, ranging from 36 to 124 nucleotide in length and with a mean Phred score > 30, were used for assembling and comparing the three assemblies. The assembly with a minimum *k*-mer count of 2 performed slightly better than the others, presenting a higher number of transcripts (284,283), a re-mapping ratio of 93.5%, a percentage of complete BUSCOs (72.3%), and N50 length (2,940 bp). We found 9,826 proteins (SwissProt *Homo sapiens*) represented by nearly full-length transcripts, with >80% alignment coverage (Table 1 and Supplementary Material 2), with a better RSEM-EVAL score than that of the other two assemblies (RSEM-EVAL score = −9,831,876,879). We predicted the likely protein coding sequences from these transcripts and annotated them with BLASTP. We also predicted 81,400 coding sequences from 185,831 transcripts with ≥300 nt. From these sequences, 79.8% (64,929) showed a significant (*e*-value < 1*e*-10) BLAST hit against an Euarchontoglires protein SwissProt dataset; the vast majority of hits being with human proteins, the most complete and curated protein dataset. As for full-length or nearly full-length transcripts, 24,016 of them cover at least 80% of the SwissProt proteins (HSP coverage).

**TABLE 1:** Summary of *Brachyteles arachnoides* transcriptome assembly, CDS prediction, annotation, and completeness according to BUSCO.

	All transcripts	Transcripts ≥ 300 nt	Coding sequences (CDS)
**No of Transcripts**	284,283	185,831	81,400
**No of BLAST Hits**	94,853	83,864	64,929
**Full Length (≥80%)**			24,016
**BUSCO Statistic**			
Complete BUSCOs	4,479 (72.3%)	4,479 (72.3%)	4,335 (70.0%)
Complete – single-copy BUSCOs	1,450 (23.4%)	1,450 (23.4%)	1,785 (28.8%)
Complete – duplicated BUSCOs	3,029 (48.9%)	3,029 (48.9%)	2,550 (41.2%)
Fragmented BUSCOs	465 (7.5%)	450 (7.3%)	504 (8.1%)
Missing BUSCOs	1,248 (20.2%)	1,263 (20.4%)	1,353 (21.9%)

**Coding sequences were predicted by Transdecoder, with full length annotation performed using BLAST*.*

### Expression Profiles and Transcriptome Annotation

**FIGURE 1:**
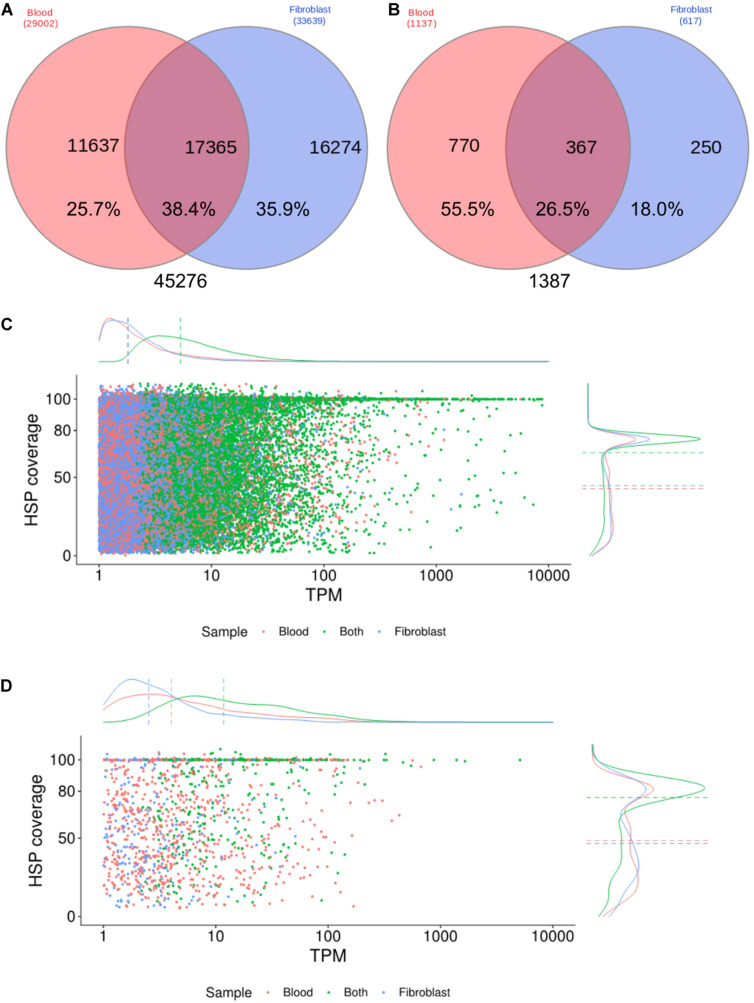
Expression profiles of the *Brachyteles arachnoides* transcriptome from blood cells and fibroblasts. **(A)** Venn diagram comparing all CDS with BLAST hit expressed (TPM ≥ 1) by blood cells and fibroblasts. **(B)** Venn diagram comparing the number of CDS homologous to proteins of the innate immune system. **(C)** HSP coverage plotted against TPM values with marginal density plots considering all CDS. **(D)** HSP coverage plotted against TPM values with marginal density plots considering CDS homologous to proteins of the innate immune system.

Transcript expression profiles were estimated by comparing annotations (SwissProt database) between the two cell samples, considering only transcripts with TPM estimates ≥ 1, accounting for 45,276, with 38.4% of them expressed in both samples (Figure 1A). Our transcriptome assembling strategy was highly geared to assemble a high rate of complete or almost-complete transcripts, with a median HSP coverage of 88.2% (Figure 1C), including low expressed transcripts, since 50% of all transcripts were expressed below 10 TPM. On the other hand, transcripts exclusively expressed in one cell sample showed lower HSP coverage (median = 57.4% and 60.1% in blood and fibroblasts, respectively) and expression level estimates (2.2 and 2.3 median TPM, in blood and fibroblasts, respectively) (Figure 1C). When considering only transcripts with homology to innate immune system proteins (Figures 1B,D), a similar pattern was observed though with a higher median TPM and a higher number of transcripts for innate immunity genes in blood cells, as expected, depicting its complexity in comparison to the fibroblast transcriptome.

Functional enrichment analysis using the Reactome database was subsequently performed to identify pathways that were significantly over-represented in each sample. Enrichments were mainly found in pathways related to the immune system (enrichment ratio 3.9, FDR = 0) and hemostasis in blood cells (enrichment ratio 3.0, FDR = 5.7E-07) (Table 2) and those associated with collagen and extracellular matrix metabolism (enrichment ratio 5.3, FDR = 4.7E-12) in fibroblasts (Table 3). Using the deconvolution method with CIBERSORTx we found 13 out of 22 cell signatures in the blood sample (Supplementary Figure 1).

**TABLE 2:** Enrichment analysis of the 500 transcripts with the highest TPM estimates in blood cells.

Gene set	Description	Size of gene set	Overlap	Expect	Enrichment ratio	Adj. *p*-value
R-HSA-168249	Innate immune system	535	88	22.6	3.9	0
R-HSA-109582	Hemostasis	279	35	11.8	3.0	5.7E-07
R-HSA-372790	Signaling by GPCR	252	33	10.7	3.1	5.7E-07
R-HSA-375276	Peptide ligand-binding receptors	42	10	1.8	5.6	5.6E-04
R-HSA-202403	TCR signaling	69	12	2.9	4.1	1.8E-03
R-HSA-76005	Response to elevated platelet cytosolic Ca^2+^	61	11	2.6	4.3	2.4E-03
R-HSA-202430	Translocation of ZAP-70 to Immunological synapse	6	4	0.3	15.8	2.5E-03
R-HSA-2029480	Fcgamma receptor (FCGR) dependent phagocytosis	57	10	2.4	4.1	5.4E-03
R-HSA-2871809	FCERI mediated Ca^2+^ mobilization	20	6	0.8	7.1	5.8E-03
R-HSA-449147	Signaling by Interleukins	237	23	10.0	2.3	5.9E-03

**Reactome database was used for enrichment*.*

**TABLE 3:** Enrichment analysis of the 500 transcripts with the highest TPM estimates in fibroblasts.

Gene set	Description	Size of gene set	Overlap	Expect	Enrichment ratio	Adj. *p*-value
R-HSA-1474244	Extracellular matrix organization	128	31	5.8	5.3	4.7E-12
R-HSA-8948216	Collagen chain trimerization	14	9	0.6	14.1	3.2E-07
R-HSA-8951671	RUNX3 regulates YAP1-mediated transcription	7	5	0.3	15.7	3.8E-04
R-HSA-381426	Regulation of Insulin-like Growth Factor (IGF) transport and uptake by Insulin-like Growth Factor Binding Proteins (IGFBPs)	52	11	2.4	4.6	1.5E-03
R-HSA-8874081	MET activates PTK2 signaling	16	6	0.7	8.2	3.9E-03
R-HSA-446353	Cell-extracellular matrix interactions	11	5	0.5	10.0	5.5E-03

**Reactome database was used for enrichment*.*

### Classification of Known and Novel Protein-Coding Genes

In addition to the functional annotation of the CDS carried out with the Supraprimates SwissProt database, searches were performed in the eggNOG and Pfam databases (Supplementary Material 3). From the 64,929 CDS with a significant BLAST hit against a Euarchontoglires protein, 34,084 sequences showed identity and HSP coverage over 60%, and these were considered homologs (orthologs or recent paralogs) to Supraprimates proteins. Of the remaining sequences (30,845), 94% (29,056) were associated with a protein domain or a KEGG Orthology (KO) group (Supplementary Material 4) and were indicated as potential novel or specific protein-coding genes of *B. arachnoides*. In addition, 4,537 sequences without BLAST hits, showed hits with a protein domain or KO, increasing the number of potential novel isoforms or genes to 33,593. The top three COG categories of these sequences were ‘signal transduction mechanisms,’ ‘function unknown,’ and ‘transcription’ (Supplementary Figure 2). The vast majority of the predicted peptides (11,274 from 11,934) which were not associated with any element of the databases searched herein were shorter than 200 amino acids, either too fragmented for proper identification or spuriously predicted (Supplementary Material 4).

### Identification of Gene Duplication and Species-Specific Orthogroups

**FIGURE 2:**
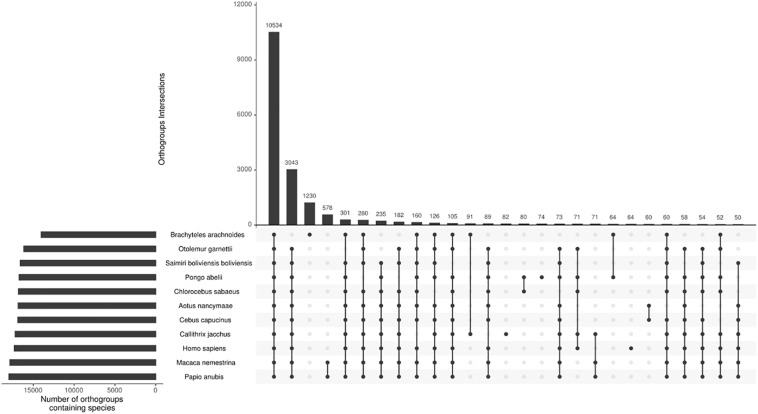
Orthogroups intersections among 11 primate species. The top bar plot represents the number of orthogroups intersections. The bottom-left bar plot represents the number of orthogroups per species. The dots indicate the intersections among the species with at least 50 shared orthogroups.

From the total 81,400 ORF’s predicted from the transcriptome sequences, 58,138 have TMP estimates ≥ 1, and using CD-HIT with a threshold of 95% of similarity, we reduce the sequence redundancy to 37,987 predicted protein sequences. Adding this to the proteomes of the ten other selected primates, we end up with 245,988 sequences for orthogroups inference. The comparative analysis of the 11 primate species assigned 92.9% of the sequences in orthogroups (Supplementary Material 5). The southern muriqui showed the lowest percentage of genes assigned to an orthogroup (Figure 2), this might be a result of spurious ORF’s prediction from non-coding RNA’s present in the transcriptome. However, the absolute number of assigned genes of *B. arachnoides* is comparable to the other species (Supplementary Material 5). Of 21,369 inferred orthogroups, 10,534 (49.3%) have all species present (Figure 2), further validating the completeness and quality of the transcriptome presented here. This analysis was able to identify 4,101 single-copy orthogroups representing a core set of conserved ortholog genes among primates. In addition to these conserved genes, 1,230 orthogroups were species-specific to *B. arachnoides*, due to in-paralogs grouping. Although we know that these in-paralogous sequences are the result of duplications after speciation, this high number might be due to the use of a transcriptome instead of a reference genome, which is absent for this species.

**FIGURE 3:**
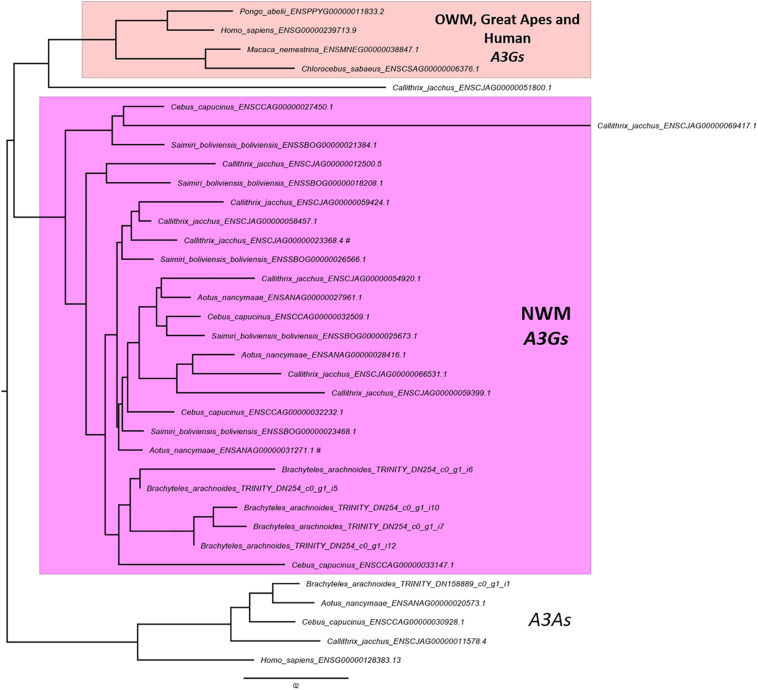
Maximum likelihood phylogeny of primate APOBEC3Gs. A PhyML tree of aligned amino acid sequences. Human and NWM APOBEC3As were used to root the tree. A cluster with NWM APOBEC3Gs and retrocopies are shown in pink. The sister clade of OWM, Great Apes and Human APOBEC3Gs are shown in light pink. The scale bar represents the amino acid substitution rate, using the Blosum62 + GAMMA model. NWM APOBEC3Gs sequences associated with RefSeq accessions are marked with hash sign (#).

The OrthoFinder also performs the identification of gene duplication events across the studied lineages analyzing the gene trees of each orthogroup and comparing with the species tree. This analysis inferred 3,877 orthogroups with a duplication event on the terminal branch of southern muriqui, of which 3,642 shared orthologs with human proteome. This data constitutes a rich and useful resource that can be further used for a variety of evolutionary genomics projects aiming recent gene family expansions. This analysis also detected well-supported 45 duplication events in the common ancestor of NWM. Among these events we identified an expansion of *APOBEC3G* in Platyrrhini clade, including three copies in *A. nancymaae* (Nancy Ma’s night monkey), nine copies in *C. jacchus* (common marmoset), four copies in *C. capucinus* (White-faced capuchin), five copies in *S. boliviensis boliviensis* (Bolivian squirrel monkey) and five copies in *B. arachnoides*. On the other hand just one ortholog was identified in the Old World monkeys, Great Apes, and Human genomes (Figure 3). In fact, Trinity has assembled 12 transcripts coding for *APOBEC3G*, of which 9 presented TPM estimates greater than 1 and these were clustered in five representative sequences that seem to have originated from different genes. A maximum likelihood phylogenetic tree using the aligned *APOBEC3G* amino acid sequences revealed a great cluster of NWM sequences separated from Catarrhini sequences (Figure 3). However, the tree reconstruction was not able to recover the relation among NWM *APOBEC3G* sequences, since the canonical genes of *A. nancymaae* and *C. jacchus* appeared in different branches.

### Adaptive Selection Analysis

Identification of genes under adaptive selection in the *B. arachnoides* lineage was performed with an initial set of 4,533 transcripts successfully identified as having homologous sequences in *C. jacchus*, *S. boliviensi* and *H. sapiens*. This analysis identified 30 genes in *B. arachnoides* (adjusted *p*-value < 0.05) which, according to GO, were associated with the biological processes and molecular functions listed in Supplementary Material 3. These positively selected genes belong to a wide variety of categories, such as the innate immune system (*APOBEC3G*, *OAS2*, and *CEACAM1*), the immune response (*CLEC17A*, *ITGAM*, *SLC15A2*, *ECM1*, *HLA-DRB1*, and *GZMB*), ion transport (*SLC39A13*), rRNA processing and translation (*BMS1*, *DDX52*, and *MRPS14*), and DNA binding proteins (*TAF9B*, *ZSWIM8*, and *ZBTB44*). The protein alignments with domain information and the reconstructed gene trees of each positively selected CDS using the 11 different primate species are displayed in the Supplementary Figures 3, 4, respectively. Main lineages presented a monophyletic grouping in most trees, with exceptions being caused in each case by the anomalous position of a single species.

**FIGURE 4:**
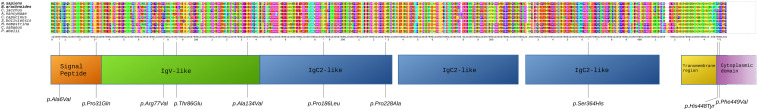
Alignment of CEACAM1 proteins from nine primate species. Human and *B. arachnoides* sequences are displayed in the first lines to allow a direct comparison of the altered amino acids. Nine primate sequences were compared to the predicted peptide sequence of *B. arachnoides*. Numbering corresponds to amino acid (aa) positions considered in the FEL analysis for positively selected sites. Boxes correspond to protein domains and lines point to positively selected aa substitutions.

In order to identify sites under positive selection, FEL was used for estimating rates of non-synonymous (dN) and synonymous (dS) nucleotide substitutions (ω) at each site in a sequence alignment comprising 11 primate species. Among the 30 genes under adaptive selection in *B. arachnoides* (Supplementary Material 6), significant estimates of positive selection with ω > 1.0 (LRT, adj-*p* < 0.05) were found at 132 sites (Supplementary Material 7). Two genes related to the innate immune response (*OAS2* and *CEACAM1*) showed the highest number of sites under positive selection, with 13 and 10 sites with ω > 1.0, respectively. Most *OAS2* sites were located in *OAS* domain 1, while two *CEACAM1* sites occurred at a signal peptide region, three at the immunoglobulin variable-region-like (IgV-like) domain, three other distributed along two of the three immunoglobulin constant-region type-2-like (IgC2-like) domains and the last two at the initial portion of the cytoplasmic domain (Figure 4). A third gene related to the innate immune response (*APOBEC3G*), showed seven sites inside the cytidine and deoxycytidylate deaminase domain. Interestingly, 13 of the 27 remaining genes under adaptive selection encoded membrane or extracellular matrix proteins, nine of which at the plasma membrane (Supplementary Material 6).

### Molecular Modeling of APOBEC3G and CEACAM1

**FIGURE 5:**
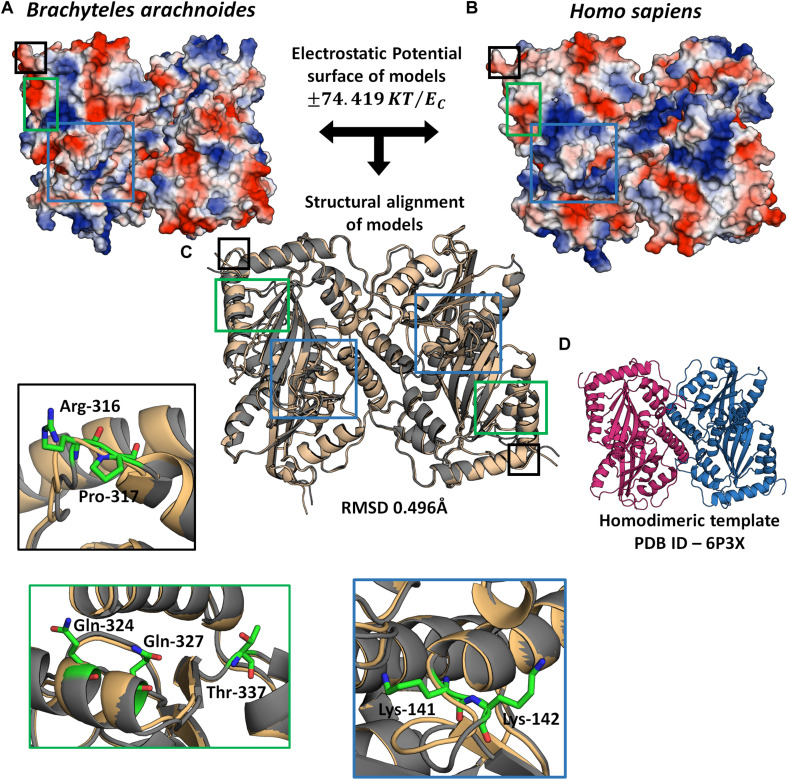
3D Model of APOBEC3G homodimer. Electrostatic profile of *B. arachnoides* **(A)** and *H. sapiens* **(B)** APOBEC3G: the bluest zones on protein surface represent values +74.419 KT/e_*c*_; the reddest zones represent values −74.419 KT/e_*c*_ of the electrostatic properties; white regions mean zero values of the electrostatic potential. **(C)** The structural alignment between APOBEC3G *Homo sapiens* models (gold cartoon) and *B. arachnoides* (gray cartoon). **(D)** Template used for structural modeling, showing the homodimeric interaction between two APOBEC3Gs. Insets show residues (in sticks) under positive selection in muriqui sequence, border color of insets corresponds to each specific highlighted region in C.

In order to evaluate the possible impact of sites under positive selection on APOBEC3G and CEACAM1, we used a 3D modeling approach to assess their structures. The APOBEC3G structures of *H. sapiens*, shown as a ribbon diagram (Figure 5D), and *B. arachnoides* were superposed. The analysis presented a RMSD value of 0.469 (Figure 5C), and both proteins have a similar conformation due to the modeling by satisfaction of spatial restraints (Supplementary Figure 6). However, when we compare the electrostatic potential profile of both models, we can observe a more electrophilic region in the center of human monomers of APOBEC3G (Figure 5B) in comparison to the southern muriqui protein, which has a more neutral profile in the same region (Figure 5A). The superposition of CEACAM1 models of *H. sapiens* and *B. arachnoides* presented a higher RMSD value (3.426) (Supplementary Figure 5) in comparison to APOBEC3G models, and this high value is a consequence of a coil region, located on aminoacid residues ranging from 421 to 455 (Supplementary Figure 5). This same region in the *H. sapiens* model presents a small beta sheet structure, probably a consequence of residues His449 and Phe450. Overall, both CEACAM1 models have only beta sheet folding and a similar structure (Supplementary Figure 6). As it occurred with APOBEC3G, the electrostatic potential profile of CEACAM1 shows some differences in the comparison of the two proteins (Supplementary Figure 5), mainly on residues 77 and 346.

## Discussion

We present herein, for the first time, a high-quality transcriptome assembly report on the critically endangered New World monkey, *B. arachnoides*, providing valuable genetic data of a species whose reference genome is still unavailable, as well as the first transcriptome report of a representative member of the family Atelidae. This work also provides the first transcriptome analysis of both blood cells and fibroblasts of any New World monkey species, assembling and annotating more than 24,000 full-length protein-coding sequences from these cell samples. It is also relevant to highlight the richness and completeness of this dataset, as the blood sample presented here showed 13 out of 22 cell signatures and the comparative analysis showed a high number of inferred orthogroups across all primate lineages studied. The Human Protein Atlas^2^ (June 8th, 2020) examined 19,670 protein coding genes, and presents their expression in many different tissues. In the case of blood, a total of 16,063 genes, corresponding to 81.7% of the total, are detected by RNA-seq. This number agrees with previous publications indicating that blood transcriptome presents a rich source of information in itself (Newman et al., 2015).

A comparison with other *de novo* assemblies of New World monkeys showed that the N50 length of *B. arachnoides* contigs was similar to that in *C. jacchus* assemblies (2,441–2,935 bp) (Maudhoo et al., 2014) but higher than that in *Cebus apella* (1,809 bp) (Boddy et al., 2017). Comparison of transcriptional profiles between cell samples showed a higher number of coding sequences expressed exclusively in fibroblasts than in blood cells. Fibroblast genes were enriched in fewer pathways than blood genes, pointing to the higher transcript complexity resulting from the different cell types present in blood.

Sequencing and *de novo* assembly of *B*. *arachnoides* transcriptome provided information on several genes despite the lack of a reference genome which was useful for testing evolutionary hypotheses and selection. Analyses of 4,528 sets of *B. arachnoides* orthologs using a branch-site model revealed evidence of positive selection in 30 genes, at 132 amino acid sites, several of them within host defensive genes. This probably reflects the selective pressures imposed by pathogen infections on proteins encoded by immune system genes which have been targets of positive selection and rapid evolutionary adaptation (Barreiro and Quintana-Murci, 2010; Daugherty and Malik, 2012; Akkaya and Barclay, 2013; Rausell and Telenti, 2014).

In this study, the *OAS2* gene presented the highest number of sites under positive selection. It encodes a 2′-5′-oligoadenylate synthase 2 whose homodimer may bind to viral double-stranded RNA and catalyze synthesis of 2′-5′ oligoadenylates, leading to activation of RNase L and RNA degradation (Williams and Kerr, 1978; Sarkar et al., 1999). In humans, the *OAS* family comprises four paralogs (*OAS1*, *OAS2*, *OAS3*, and *OASL*) distinguished by the number of OAS units, which is the number of NTase and OAS1-C domains they contain as a result of genomic tandem duplications (*OAS1*- one unit, *OAS2*- two units, and *OAS3*- three units) (Hancks et al., 2015; Mozzi et al., 2015). These authors postulated that these domain duplications and fusions might be adaptive, making *OAS2* and *OAS3* encoded proteins more resistant to pathogen-encoded inhibitors than the one encoded by *OAS1*, making *OAS1* a more frequent target of positive selection than *OAS2*. In this study, however, analysis of the almost complete, assembled sequence of the *OAS1* ortholog in *B. arachnoides* did not show evidence of positive selection.

In humans, several studies have shown associations of single nucleotide polymorphisms located within the *OAS* genes and increased risk of infection by viruses of the genus *Flavivirus*, including West Nile virus, dengue virus and tick-borne encephalitis virus (Lim et al., 2009; Barkhash et al., 2010; Thamizhmani and Vijayachari, 2014). Another relevant zoonotic disease caused by a Flavivirus is the yellow fever. Monath (2012) reviewing the risks and benefits of taking the live, attenuated yellow fever 17D vaccine, reported associations of a vaccine-associated viscerotropic disease and polymorphisms in innate immune genes, including *OAS1* and *OAS2* genes. From 2016 to 2018, Brazil has experienced the worst outbreak of yellow fever in the last 80 years, with high numbers of humans and non-human primates deaths (Possas et al., 2018). Although *Alouatta* is reported to be the most susceptible species to yellow fever virus, as reviewed by Strier et al. (2019) and Silva et al. (2020) reported reductions of 10–26% of two populations of *B. hypoxanthus* in areas with yellow fever outbreaks, both being Atelid species. Our results on positive selection sites identified in *OAS2* suggest that variations within *OAS* genes can be related to a high susceptibility to *Flavivirus*, as seen in humans, and this transcriptome is a valuable resource for future studies aiming to unveil this association.

*CEACAM1*, a gene belonging to the carcinoembryonic antigen (CEA) gene family, was also found to be under positive selection. It encodes the carcinoembryonic antigen-related cell adhesion molecule 1, a glycoprotein involved in cell adhesion, neovascularization, insulin homeostasis, and T-cell regulation, and plays an important role as a target for pathogen interaction and internalization (Hauck et al., 2006; Kuespert et al., 2006). *CEACAM1* has rapidly evolved in vertebrates, including humans, in a host, species-specific manner (Kammerer et al., 2004, 2007; Kammerer and Zimmermann, 2010; Voges et al., 2010; Zimmermann and Kammerer, 2016). One of the protein domains that was identified as harboring sites under positive selection was the immunoglobulin variable-region-like (IgV-like) domain, which was reported to be responsible for functional differences regarding bacterial binding (Voges et al., 2010), correlating with the restricted pathogenicity of the *CEACAM1*-targeting microorganisms.

In addition to our findings on adaptive selection in genes related to the immune response, we identified the *APOBEC3G* gene to be potentially under positive selection. The protein encoded by this gene belongs to the APOBEC3 family of cytidine deaminases which binds to single-stranded DNA and converts cytosine to uracil (Navaratnam and Sarwar, 2006). This protein can inhibit retroviral infection and endogenous retrotransposons, such as *Alu* elements (Hulme et al., 2007; Mbisa et al., 2010; Hultquist et al., 2011) and is classified as a host restriction factor, an important protective component of the innate immune system (Chiu and Greene, 2008). APOBEC3G has been subject to strong positive selection throughout primate evolution, possibly reflecting a long-term evolutionary conflict between retroviruses and their primate hosts (Sawyer et al., 2004; Bulliard et al., 2009; Krupp et al., 2013). In the comparative analysis, we also found several copies of this gene, which is in agreement with a recent published paper that found that NWM genomes, specifically Cebidae family, have accumulated *APOBEC3G* copies derived from retrotransposition, moreover these retrocopies can be transcribed and retain functional domains (Yang L. et al., 2020). Our findings show for the first time an Atelid species with *APOBEC3G* expansion and extend the knowledge that this might have occurred in the common ancestor of NWM.

Though, the seven sites herein identified to be under positive selection in *APOBEC3G* are present inside the cytidine and deoxycytidylate deaminase domain (InterPro ID: IPR002125), they are located downstream of two zinc-coordinating motifs (Navarro et al., 2005) and outside the intermonomeric contact region identified by Bulliard et al. (2009) and these sites did not affect the structure of the molecular model. However, the differences in the electrostatic profile might have a functional impact, though it is speculative. A recent work showed that mutating the residues in the dimeric interface was not sufficient to cancel the HIV-1 restriction (Yang H. et al., 2020). In fact, cloned NWM *APOBEC3G* retrocopies restrict HIV-1 but not LINE-1 (Yang L. et al., 2020), showing that differences in the sequence as consequence of accelerated evolution do not affect the antiviral activity and the positive selection on *APOBEC3G* gene might have occurred through a viral-driven evolutionary pressure. Nevertheless, more functional studies are needed for defining the nature of these underlying evolutionary forces.

Interestingly, several genes under positive selection encoded membrane or extracellular matrix proteins, some of which localized in the plasma membrane. Scarce available information on their evolution and divergence notwithstanding, they were associated with immunological functions, leading us to suggest that they might be under similar evolutionary pressures to the above mentioned genes. Such was the case of C-type lectin domain family 17, member A (*CLEC17A*), also known as Prolactin, a glycan-binding receptor that may be involved in innate immunity as a pattern recognition receptor and whose expression was reported in association with proliferating B cells in germinal centers (Graham et al., 2009; Angulo et al., 2019). Integrin alpha-M (*ITGAM*) was identified as the major cell receptor of neutrophils in the recognition of pathogenic fungi that may evade the complement system (Losse et al., 2010). Solute carrier family 15, member 2 (*SLC15A2*) is responsible for the intake of oligopeptides of 2–4 amino acids, and was implicated in the uptake optimization of bacterial peptides into the cytosol of macrophages, enhancing the production of proinflammatory cytokines (Hu et al., 2018). Extracellular matrix protein 1 (*ECM1*) is a soluble protein described in the promotion of follicular helper cell differentiation and antibody production (He et al., 2018). Our findings corroborate those of Nielsen et al. (2005) who reported a high number of immune-related genes targeted by positive selection in the genomes of humans and chimpanzees as a consequence of strong pathogen-driven evolutionary pressure. Future functional studies of these genes and their encoded proteins may help to elucidate their physiological implications in *B*. *arachnoides*.

## Conclusion

Transcriptome assembly of the southern muriqui (*B. arachnoides*), a critically endangered New World monkey, has proven to be a valuable genetic resource of a species whose reference genome is presently unavailable. It also provides the first transcriptome account of an Atelid primate. The high throughput approach for analyzing transcriptome data identified 30 candidate genes under positive selection with strong statistical evidence, and an important gene expansion event, shedding light on the evolutionary pressures modeling the primate genome, mainly on genes involved in the immune response. Furthermore, we presented a comparative analysis with other primates genomes, extending the knowledge of NWM genomic evolution. This work generated an abundant catalog of complete sequences that may be used to further elucidate several other aspects of genome evolution in primates as well as unveil the evolutionary diversification genes involved in the immune response. Further functional studies are required to provide an understanding of the nature of these underlying evolutionary processes.

## Data Availability Statement

The datasets presented in this study can be found in online repositories. The names of the repository/repositories and accession number(s) can be found below: https://www.ncbi.nlm.nih.gov/, SRR10907267-SRR10907271 and https://www.ncbi.nlm.nih.gov/, GIJV00000000.

## Ethics Statement

The animal study was reviewed and approved by IBAMA (Instituto Brasileiro do Meio Ambiente e dos Recursos Naturais Renováveis, Brazil; permanent license number 111375-1).

## Author Contributions

DM, AL, RS, and NS performed the data analysis. DM wrote the manuscript. DM, AL, AC, MM, HS, and MB contributed with data interpretation and manuscript preparation. CF performed the experiments and Illumina sequencing. HS and MB conceived and designed the work and reviewed the manuscript. All authors read and approved the final manuscript.

## Conflict of Interest

The authors declare that the research was conducted in the absence of any commercial or financial relationships that could be construed as a potential conflict of interest.

## Footnotes

1 http://geneontology.org/

2 https://www.proteinatlas.org/humanproteome/tissue
