# Supplementary figures and images for: Transcriptome of the Southern Muriqui Brachyteles arachnoides (Primates:Platyrrhini), a Critically Endangered New World Monkey: Evidence of Adaptive Evolution
Source: Front Genet. 2020 Jul 31;11:831. doi: 10.3389/fgene.2020.00831 (PMC7412869; doi:10.3389/fgene.2020.00831)

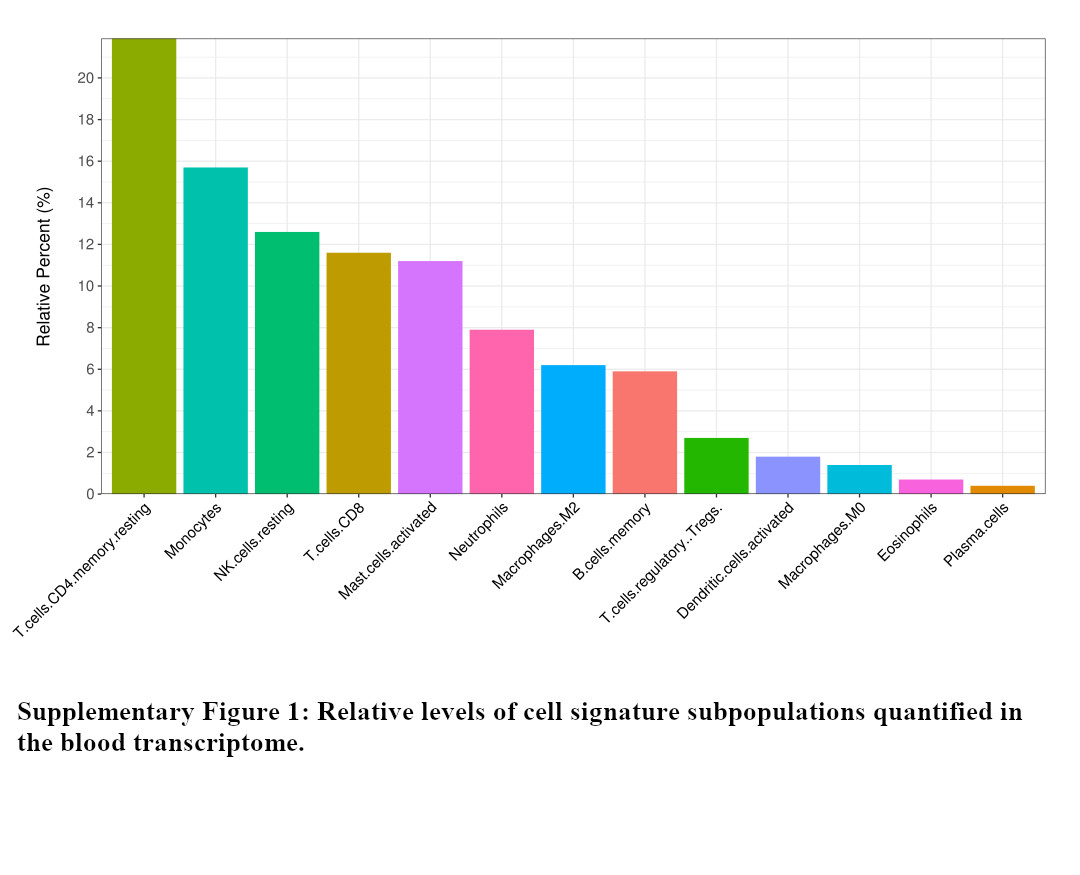

Supplement: Supplementary file 1 [file Image_1.JPEG]

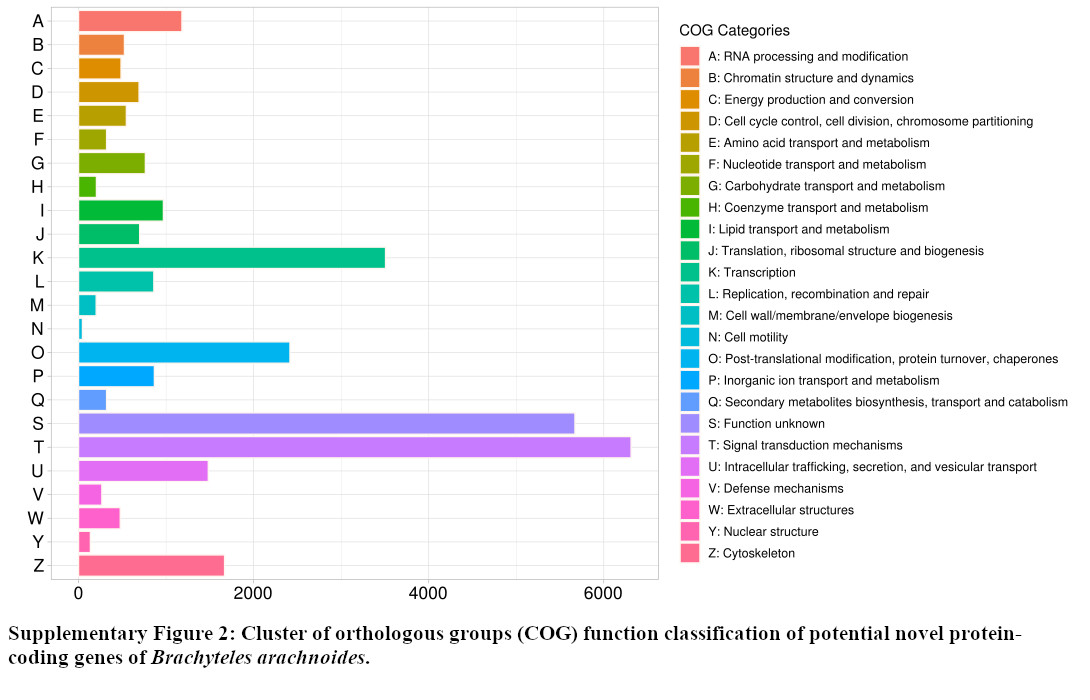

Supplement: Supplementary file 2 [file Image_2.JPEG]
