# Supplementary material for: Transcriptome of the Southern Muriqui Brachyteles arachnoides (Primates:Platyrrhini), a Critically Endangered New World Monkey: Evidence of Adaptive Evolution
Source: Front Genet. 2020 Jul 31;11:831. doi: 10.3389/fgene.2020.00831 (PMC7412869; doi:10.3389/fgene.2020.00831)
Supplement: Supplementary file 3 [file Image_3.pdf]

Supplementary Figure 3: Multi-species alignments of the proteins with positively selected sites identified by CODEML and FEL. To prepare the images, aligned coding sequences were converted to amino acid sequences in SeaView, manually re-numbered and colorized in DNATagger (https://bioinfo.inca.gov.br/DNATagger). Protein domains identified by interproscan in the following databases: Hamap, ProSiteProfiles, ProSitePatterns, Pfam, TIGRFAM, SMART, PRINTS, SFLD, CDD, Gene3D, ProDom, PIRSF, PANTHER, SUPERFAMILY, were manually added to the alignments.

AKAP11

```
                                                              *                     *
Brachyteles_arachnoides   MRNINKPSDISSDPLNQSHHSGMLCVMRVSHTLPRLKIDFIFSLLSKYATGIRYTLDTLL
Callithrix_jacchus        MRNINKSSDISSDPLNQSHHSGMLCVMRVSHTLPRLRIDFIFSLLSKYATGIRYTLDTFL
Aotus_nancymaae           MRNINKPSDISSDPLNQSHHSGMLCVMRVSHTLPRLRIDFIFSLLSKYATGIRYTLDTFL
Cebus_capucinus_imitator  MRNINKPSDISSDPLNQSHRSGMLCVMRVSHTLPRLRIDFIFSLLSKYATGIRYTLDTFL
Saimiri_boliviensis       MRNINKPSDISSDPLNQSHHSGMLCVMRVSHTLPRLRIDFIFSLLSKYATGIRYTLDTFL
Macaca_nemestrina         MKNINKPSDISSDPLNQSHPSGMLCVMRVSPTSPKLRIDFIFSLLSKYATGIRYTLDTFL
Papio_anubis              MKNINKPSDISSDPLNQSHPSGMLCVMRVSPTSPKLRIDFIFSLLSKYATGIRYTLDTFL
Chlorocebus_sabaeus       MKNINKPSDISSDPLNQSHPSGMLCVMRVSPTSPKLRIDFIFSLLSKYATGIRYTLDTFL
Homo_sapiens              MKNINKPLDISSDPLNQSHPSGMLCVMRVSPTSPRLRIDFIFSLLSKYATGIRYTLDTFL
Pongo_abelii              MKNINKPSDISSDPLNQSHPSGMLCVMRVSPTSPRLRIDFIFSLLSQYATGIRYTLDTFL
                          123456789012345678901234567890123456789012345678901234567890
                                   1         2         3         4         5         6

Brachyteles_arachnoides   HQKHQLETTDEDDDDTNQSVSSIEDDFVTAFEHLEEEETSKSYNNGMNITVLRSQCDAAS
Callithrix_jacchus        HQKHQLETTDEDDDDTNQSVSSIEDDFVTAFEHLEEEETSKPYNNGMNITVLRSQCDAAS
Aotus_nancymaae           HQKHQLETTDEDDDDTNQSVSSIEDDFVTAFEHLEEEETSKPYNNGMNITVLRSQCDAAS
Cebus_capucinus_imitator  HQKHQLETTDEDDDDTNQSVSSIEDDFVTAFEHLEEEETSKLYNNGMNITVLRSQCDAAS
Saimiri_boliviensis       HQKHQLETTDEDDDDTNQSVSSIEDDFVTAFEHLEEEETSKPYNNGMNITVLRSQCDAAS
Macaca_nemestrina         HQKHQLETTDEDDDDTNQSVSSIEDDFVTAFEHLEEEETSKPYNDGMNITALRNQCDAAS
Papio_anubis              HQKHQLETTDEDDDDTNQSVSSIEDDFVTAFEHLEEEETSKPYNDGMNVTALRNRCDAAS
Chlorocebus_sabaeus       HQKHQLETTDEDDDDTNQSVSSIEDDFVTAFEHLEEEETSKPYNDGMNITALRNQCDAAS
Homo_sapiens              HQKHQLETTDEDDDDTNQSVSSIEDDFVTAFEHLEEEETSKPYNDGMNITVLRSQCDAAS
Pongo_abelii              HQKHQLETTDEDDDDTNQSVSSIEDDFVTAFEHLEEEETSKPYNDGMNITVLRSQCDAAS
                          123456789012345678901234567890123456789012345678901234567890
                         61        7         8         9       100         1         2

                                          |---------------- Ser-rich -----------------
Brachyteles_arachnoides   QTVTGHHLETHDLKILISSGQQKSLAKPSTSPMNVLRQKELPSVKTSVTTSISEPWTQRS
Callithrix_jacchus        QTVTGHPLETQDLKILISSGQQKSLAKPSTSSMNVLGQKELPSVKTSVTTSVSEPWTQRS
Aotus_nancymaae           QTVTGHHLETHDLKILISSGQQKSLAKPSTSSMNVLGQKELPSVKTSVTTSVSEPWTQRS
Cebus_capucinus_imitator  QTVTGHHLEAHDLKILISSGQQKSLAKSSASSMNVLGQKELPSVKTSVTTSISEPWTQRS
Saimiri_boliviensis       QTVTGHHLETHDLKILIGSGQQKSLAKPSTSSMNVLGQKELPSVKTSVTTSISEPWTQRS
Macaca_nemestrina         QTVTGHHLETHDLKILISSGQQKSLAKPSTSSVNVLGPKELPSVKTSVTTSISEPWTQRS
Papio_anubis              QTVTGHHLETHDLKILISSGQQKSLAKPSTSSVNVLGPKELPSVKTSVTTSISEPWTQRS
Chlorocebus_sabaeus       QTVTGHHLETHDLKILISSGQQKSLAKPSTSSVNVLGPKELPSVKTSVTTSISEPWTQRS
Homo_sapiens              QTVTGHHLETHDLKILISSGQQKSLAKPSTSSVNVLGHKELPSVKTSVTTSISEPWTQRS
Pongo_abelii              QTVTGHHLETHDLKILISSRQQKSLAKPSTSSVDVLGHKELPSVKTSVTTSISEPWTQRS
                          123456789012345678901234567890123456789012345678901234567890
                        121        3         4         5         6         7         8

                          -----------------------------------|
Brachyteles_arachnoides   FYRSSNASDKDSDLQKTFF-SSPAYSSESECSSPSPVIFLDEEGYQKSLKAKLELPKIPV
Callithrix_jacchus        FYRSSNASDKDNDLQKTFV-SSPAYSSESECSSPSPVIFLDEEGYQKSLKAKLELPKIPV
Aotus_nancymaae           FYRSSNASDKDSDLQKTFFSSSPAYSSESECSSPSPVIFLDEEGYQKSLKAKLELPKIPV
Cebus_capucinus_imitator  FYRSSNASDKDSDLQKTFFSPSPAYSSESECSSPSPVIFLDEEGYQKSLKAKLELPKIPV
Saimiri_boliviensis       FYRSSNASDKDIDLQKTFF-SSPAYSSESECSSPSPVIFLDEEGYQKSLKAKLELPKIPV
Macaca_nemestrina         FYRSSNASDKDSDLQKTFCSSSPAYSSESECSSPSPVIFLDEEGYQKSLKAKLELPKIPV
Papio_anubis              FYRSSNASDKESDLQKTFFSSSPAYSSESECSSPSPVIFLDEEGYQKSLKAKLELPKIPV
Chlorocebus_sabaeus       FYRSSNASDKDSDLQKTFFSSSPAYSSESECSSPSPVIFLDEEGYQKSLKAKLELPKIPV
Homo_sapiens              FYRSSNASDKDSDLQKTFF-SSPAYSSESECSSPSPVIFLDEEGYQKSLKAKLELPKIPV
Pongo_abelii              FYRSSNASDKDSDLQKTFFSSSPAYSSESECSSPSPVIFLDEEGYQKSLKAKLELPKIPV
                          1234567890123456789-0123456789012345678901234567890123456789
                        181        9        200         1         2         3

Brachyteles_arachnoides   MKDDIEDSDSEVSEFFDSFDQFDELEQTLETCLFNKDAVIGKSSQKKGHKHGKSCMNPQK
Callithrix_jacchus        MKDDIEDSDSEVSEFFDSFDQFDELEQTLETCLFNKDPVIGKSSQKKGHKHGKSCMNPQK
Aotus_nancymaae           MKDDIEDSDSEVSEFFDSFDQFDELEQTLETCLFNKDPIIRKSSQKKGHKHGKSCMNPQK
Cebus_capucinus_imitator  MKDDIEDSDSEVSEFFDSFDQFDELEQTLETCLFNKDPVIRKSSQKKGHKHGKSCMNPQK
Saimiri_boliviensis       TKDDIEDSDSEVSEFFDSFDQFDELEQTLETCLFNKDPVIRKSSQKKGHKRGKSCMNPQK
Macaca_nemestrina         MKDDIEDSDSEVSEFFDSFDQFDELEQTLETYLFNKDPVIGKSSQKKGHKHGKSCMNPQK
Papio_anubis              MKDDIEDSDSEVSEFFDSFDQFDELEQTLETYLFNKDPVIGKSSQKKGHKHGKSCMNPQK
Chlorocebus_sabaeus       MKDDIEDSDSEVSEFFDSFDQFDELEQTLETYLFNKDPVIGKSSQKKGHKHGKSCMNPQK
Homo_sapiens              MKDDIEDSDSEVSEFFDSFDQFDELEQTLETCLFNKDPVIGKSSQRKGHKHGKSCMNPQK
Pongo_abelii              MKDDIEDSDSEVSEFFDSFDQFDELEQTLETYLFNKDPVIGKSSQKKGHKHGKSCMNPQK
                          012345678901234567890123456789012345678901234567890123456789
                       240          5         6         7         8         9
```

```
Brachyteles_arachnoides     FKFDRPALPANVRKPTPRKPESPYGNLCDAPDSPRPVKASGEDSGLFSPIRSSAFSPLGG
Callithrix_jacchus          FKFDRPALPANVRKPTPRKPESPYGNLCDAPDSPRPVKASGEDSGLFSPIRSSAFSPLGG
Aotus_nancymaae             FKFDRPALPANVRKPTPRKPESPYGNPCDAPDSPRPVKASGEDSGLFSPIRSSAFSPLGG
Cebus_capucinus_imitator    FKFDRPALPANVRKPTPRKPESPYGNLCDAPDSPRPVKASGEDSGLFSPIRSSAFSPLGG
Saimiri_boliviensis         FKFDRPALPANVRKPTPRKPESPYGNLCDAPDSPRPVKASGEDSGLFSPIRSSAFSPLGG
Macaca_nemestrina           FKFDHPALPANVRKPTPRKPESPYGNLCDAPDSPRPVKASGEDSGLFSPIRSSAFSPLGG
Papio_anubis                FKFDRPALPANVRKPTPRKPESPYGNLCDAPDSPRPVKASGEDSGLFSPIRSSAFSPLGG
Chlorocebus_sabaeus         FKFDRPALPANVRKPTPRKPESPYGNLCDAPDSPRPVKASGEDSGLFSPIRSSAFSPLGS
Homo_sapiens                FKFDRPALPANVRKPTPRKPESPYGNLCDAPDSPRPVKASREDSGLFSPIRSSAFSPLGG
Pongo_abelii                FKFDRPALPANVRKPTPRKPESPYGNLCDAPDSPRPVKASREDSGLFSPIRASAFSPLGG
                            012345678901234567890123456789012345678901234567890123456789
                          300         1         2         3         4         5
```

```
Brachyteles_arachnoides     CTPAECFCQTDIGGDRIHENHDSVYYTYEDYANSISCEVLDSVLCTQHTNALSNIDSIKH
Callithrix_jacchus          CTPAECFCQTDIGGDRIHENHDSVYYTYEDYANSISCEVLDSVLRTQHTNVLSNIDSIKH
Aotus_nancymaae             CTPAECFCQTDIGGDRIHENHDSVYYTYEDYANSISCEVLDSVLRTQHTNVLSNIDSINH
Cebus_capucinus_imitator    CTPAECFCQTDIGGDRIPENHDSVYYTYEDYANSISCEVLDSVLCTQHTNALSNIDSIKH
Saimiri_boliviensis         CTPAECFCQTDIGGDRIHENHDSVYYTYEDYANSISCEVLDSVLRTQHTNALSNIDSIKH
Macaca_nemestrina           CTPAECFCQTDIGGDRIHENLDSIYYTYEDYANNVSCEVLGSVLRTQHTNALSNIISIKH
Papio_anubis                CTPAECVCQTDIGGDRIHENLDSIYYTYEDYANNVSCEVLGSVLRTQHTNALSNIISIKH
Chlorocebus_sabaeus         CTPAECFCQTDIGGDRIHENLDSIYYTYEDYANNVSCEVLGSVLRTQHTNALSNIISIKH
Homo_sapiens                CTPAECFCQTDIGGDRIHENHDSVYYTYEDYAKSISCEVLGSVLRTHHTNTLSNINSIKH
Pongo_abelii                CTPAECFCQTDIGGDRIHENHDSVYYTYEDYANSISCEVLGSVLRTQHTNALSNINSIKH
                            012345678901234567890123456789012345678901234567890123456789
                          360         7         8         9        400         1
```

```
Brachyteles_arachnoides     GENKTVTFKHGNLDQKNKSKNKSLIIKDSIQKFAADLLEKSFGSAFKDLQKGVSSCTNAL
Callithrix_jacchus          GENKTVTFKHGNLDQKNRFKNKSLMIKDSIQKFAADLLEKSLGSAFKDLQKGVSSCTNAL
Aotus_nancymaae             GESKTVTFKHGNLDQKNKSKNKSLMIKDSIQKFAADLLEKSFGSAFKDLQKGVSSCTNAL
Cebus_capucinus_imitator    GENKTVTFKHGNLDQKNKSKNKSLVIKDSIQKFAADLLEKSFGSAFKDLQKGVSSCTNAL
Saimiri_boliviensis         GENKTVTFKHGNLDQKNKSKNKSLMIKDSIQKFAADLLEKSFGSAFKDLQKGVSSCTNAL
Macaca_nemestrina           GENKTVTFKHGNLDQKNKSKNKSLMVKDSIQKFAADLVEKSFGSAFKDLQKGVSSCTNAL
Papio_anubis                GENKTVTFKHGNLDQKNKSKNKSLMIKDSIQKFAADLVEKSFGSAFKDLQKGVSSCTNAL
Chlorocebus_sabaeus         GENKTVTFKHGNLDQINKSKNKSIMIKDSIQKFAADLVEKSFGSAFKDLQKGVSSCTNAL
Homo_sapiens                GENKTVTFKHGNLDQKNKSKNKSLMIKDSIQKFAADLVEKSFGSAFKDLQKGVSSCTNAL
Pongo_abelii                GENKTVTFKHGNLEQKNKSKNKSLMIKDSIQKFAADLVEKSFGSAFKDLQKGVSSCTNAL
                            012345678901234567890123456789012345678901234567890123456789
                          420         3         4         5         6         7
```

```
Brachyteles_arachnoides     CHLAIKLTSSVFQMAFNELRRQHTFSLKERAISSLANFLVSEALSNALKDLQYVKKQIFT
Callithrix_jacchus          CHLAIKLTSSVFQMAFNELRRQHAFSLKERAISSLANFLVSEALSNALKDLQYVKKQIFT
Aotus_nancymaae             CHLAIKLTSSVFQMAFNELRRQRAFSLKERAISSLANFLVSEALSNALKDLQYVKKQIFT
Cebus_capucinus_imitator    CHLAIKLTSSVFQMAFNELRMQCAFSLKERAISSLANFLVSEALSNALKDLQYVKKQIFT
Saimiri_boliviensis         CHLAIKLTSSVFQMAFNELKRQRAFSLKERAISSLANFLVSEALSNALKDLQYVKKQIFT
Macaca_nemestrina           CHLAIKLTSSVFQMAFNELRRQRAFSLKERAISGLANFLVSEALSNALKDLQYVKKQIFT
Papio_anubis                CHLAIKLTSSVFQMAFNELRRQRAFSLKERAISGLANFLVSEALSNALKDLQYVKKQIFT
Chlorocebus_sabaeus         CHLAIKLTSSVFQMAFNELRRQRAFSLKERAISGLANFLVSEALSNALKDLQYVKKQIFT
Homo_sapiens                YHLAIKLTSSVLQMAFDELRRQRAFSLKERAISGLANFLVSEALSNALKDLQYVKKQIFT
Pongo_abelii                CHLAIKLTSSVLQMAFDELRRQRAFSLKERAISGLANFLVSEALSNALKDLQYVKKQIFT
                            012345678901234567890123456789012345678901234567890123456789
                          480         9        500        1         2         3
```

```
Brachyteles_arachnoides     NTVARFAADLAEELVFEGIMEVCQFSYPQTPASPQCGSFDFEDKVVKSYAKDLSESVIQE
Callithrix_jacchus          NTVARFAADLAEELVFEGIMEVCQFSYPPTPASPQCGSFDFEDKVVKSYAKDLSESVIQE
Aotus_nancymaae             NTVARFAADLAEELVFEGIMEVCQFSYPQTPASPQCGSFDFEDKVVKSYAKDLSESVIQE
Cebus_capucinus_imitator    NTVARFAADLAEELVFEGIMEVCQFSYPQTPASPQCGSFDFEDKVVKSYAKDLSESVIQE
Saimiri_boliviensis         NTVARFAADLAEELVFEGIMEVCQFSYPQTPASPQCGSFDFEDKVVKSYAKDLSESVIQE
Macaca_nemestrina           NTVARFAADLAEELVFEGIMEVCQFSYPQTPASPRCGSFDFEDKVVKSYAKDLSESVIQE
Papio_anubis                NTVARFAADLAEELVFEGIMEVCQFSYPQTPASPRCGSFDFEDKVVKSYAKDLSESVIQE
Chlorocebus_sabaeus         NTVARFAADLAEELVFEGIMEVCQFSYPQTPASPRCGSFDFEDKVVKSYAKDLSESVIQE
Homo_sapiens                NTVARFAADLAEELVFEGIMEVCQFSYPQTPASPQCGSFDFEDKVVKLYAKDLSESVIQE
Pongo_abelii                NTVARFAADLAEELVFEGIMEVCQFSYPQTPASPQCGSFDFEDKVVKSYAKDLSESVIQE
                            012345678901234567890123456789012345678901234567890123456789
                          540         5         6         7         8         9
```

```
                                                           *
Brachyteles_arachnoides   AFIELSQVDVTFTTKAAVSVSTDNIKYVSAESMVPSTQAVTFSPSFHNQAIMVTKPVQEY
Callithrix_jacchus        AFVELSQVDVTFTTRAAVSVSTANIKDVSAESVVLSTQAVTFSPSFHNQAIMVTKPVQEY
Aotus_nancymaae           AFIELSQVDVTFTTKAAVSVSTDNIKYVSAESVVPSTQAITFSPSFHHQAIMVTKPVQEY
Cebus_capucinus_imitator  AFIELSQVDVTFTTKAAVSVSTDNIKYVSAESVVPSTQAVTFSPSFHNQAIMVTKPVQEY
Saimiri_boliviensis       AFIELSQVDVTFTTKAAVSVSTDNIKYVSAESVVPSTQAVTFSPSFHNQAIMVTKPVQEY
Macaca_nemestrina         AFIELSQVDVTFTTKAAVSVSTDNIKYVSAESVVPSTQAVTFSPSFHNQAIMVTKPVQEY
Papio_anubis              AFIELSQVDVTFTTKAAVSVSTDNIKYVSAESVVPSTQAVTFSPSFHNQAIMVTKPVQEY
Chlorocebus_sabaeus       AFIELSQVDVTFTTKAAVSVSTDNIKYVSAESVVPSTQAVTFSPSFHNQAIMVTKPMQEH
Homo_sapiens              AFIELSQVDVTFTTKAAVSVSTDNIKYVSAESVVPSTQAVTFSPSFHNQAIMVTKPVQEY
Pongo_abelii              AFIELSQVDVTFTTKAAVSVSTDNIKYVSAESVVPSTQAVTFSPSFHNQAIMVTKPVQEY
                          012345678901234567890123456789012345678901234567890123456789
                        600         1         2         3         4         5

Brachyteles_arachnoides   KKEYTVQQALFCTSGIVTSIPVPLAGSALLPYHISSTACQAKALLSSDDSNSNGDSAQVH
Callithrix_jacchus        KKEYTVQQALFCTSGIVTSIPVPLAGSALLPYNISSTACQAKALPSSDDINSNGDSAQVH
Aotus_nancymaae           KKEYTLQQALFCTSGIVTSIPVPLAGSALLPYHISSTACQAKALLSSDDSNSNGDSAQVH
Cebus_capucinus_imitator  KKEFTVQQALFCTSGIVTSIPVPLAGSALLPYHISSTACQAKALLSSGDSNSNGDSTQVH
Saimiri_boliviensis       KKEFTVQQALFCTSGIVTSIPVPLAGSALLPYHISSTACQAKALLSSDDSNSNGDSAQVH
Macaca_nemestrina         KKEYTVQQALFCTSGIVTSIPVPLAGSALLPYHISSTACQAKAHLSSDDSNSSGDSTQVH
Papio_anubis              KKEYTVQQALFCTSGIVTSIPVPLAGSALLPYHISSTACQAKAHLSSDDSNSSGDSTQVH
Chlorocebus_sabaeus       KKEYTVQQALFCTSGIVTSIPVPLAGSALLPYHISSTACQAKAHLSSDDSNSSGDSTQVH
Homo_sapiens              KKEYTVQQALFCTSGIVTSIPVPLAGSALLPYHISSTACQAKAHLSSDDSNSNGDSAQVH
Pongo_abelii              KKEYTVQQALFCTSGIVTSIPVPLAGSALLPYHISSTACQAKAHLSSDDSNSNGDSAQVH
                          012345678901234567890123456789012345678901234567890123456789
                        660         7         8         9       700         1

                                                                   *
Brachyteles_arachnoides   IATKNREEKAACLRNICLPSEHNPGNQNDFKPTNDDIEMQSCSKLPSDPAIISNFSAAMV
Callithrix_jacchus        IATKNREEKAACLRNICLPSEQNPGNQNDFKPTNDDIEMQSSSKLPSDPAVISNFSAAMV
Aotus_nancymaae           IATKNREEKAACLRNICLPSEQNPGNQNDFKPTNDDIEMQSSSKLPSDPAIISNFSAAMV
Cebus_capucinus_imitator  IATKNREEKAACLRNICLPSEHNPGNQNDFKPTNDDIEMQSSSKLRSDPA-ISNFSAAMV
Saimiri_boliviensis       IATKNREEKVACLRNICLPSEHNPGNTNDFKPTNDDIEMQSSSKLPSDPAIISNFSAAMV
Macaca_nemestrina         IATKNREEKAACLRNICLPSEHNPGNQNDFKPTN-DLEMQSSSKLPSDPAIISNFSAAMV
Papio_anubis              IATKNREEKAACLRNICLPSEHNPGNQNDFKPTN-DLEMQSSSKLPSDPAIISNFSAAMV
Chlorocebus_sabaeus       IATKNREEAAACLRNICLPSEHNPGNQNDFKPTNGDLEMQSSSKLPSDPAIISHFSAAMV
Homo_sapiens              IATKNREEKAACLRNICLPSEHNPGNQNDFKPTNDDIEMQSSSKLPNDPAIISNFSAAVV
Pongo_abelii              IATKNREEKAACLRNICLPSEHNPGNQNDFKPTNDDTEMQSSSKLPNDPAVISNFSAAMV
                          0123456789012345678901234567890123-456789012345678-901234567
                        720         3         4         5          6          7

Brachyteles_arachnoides   HTIVNETLESMTSLEVTKMVDEHTDYLTKSVKGKTPPFSHCDQAVLQCSEASSNKNMFAD
Callithrix_jacchus        HTTVNETLESVTSLEATKMVDEHTDYLTKSVKGKTPPFSHCHQAVLQCSEASSNKNMFAD
Aotus_nancymaae           HTIVNETLESKTSLEVTKMVDEHTDYLTKSVKGKTPPFSHCDQAVLQCSEASSNKNMFAD
Cebus_capucinus_imitator  HTIVNETLQSMTSLEVTKMVDEHTDYLTKSVKGKTPSFSHCDQAVLECSEASSNKNMFAD
Saimiri_boliviensis       HTIVNETLESMTSLEVTKMVDEHADYLTKSVKGKTPPFSHCDEAVLECSEASSNKNMFAD
Macaca_nemestrina         HTIVNETLESMTSLEVTKMVDECTDYLTKSIKGKTPPFSHCGQAVLQCSEASSNKDVFAD
Papio_anubis              HTIVNETLESMTSLEVTKMVDERTDYLTKSIKGKTPPFSHCGQAVLQCSEASSNKDMFAD
Chlorocebus_sabaeus       HTIVNETLESMTSSEVTKMVDERTDYLTKSIKGKTPPFSHCGQAVPQCSEASSNKDMFAD
Homo_sapiens              HTIVNETLESMTSLEVTKMVDERTDYLTKSLKEKTPPFSHCDQAVLQCSEASSNKDMFAD
Pongo_abelii              HTIVNETLESMTSLEVTKMVDERTDYLTKSVKEKTPPFSHCDQAVLQCSEASSNKDMFAD
                          890123456789012345678901234567890123456789012345678901234567
                          780         9       800         1         2         3

Brachyteles_arachnoides   QLSKSVIKHSIDKSKSVIPSVDKNAVYKEGLPVLGEESHLTLKKSPKFSESHNHLTHCSL
Callithrix_jacchus        QLSKSIIKHSIDKSKSVIPNMDKNAIYKEGLPVPGEESQWTPEKSPKFSESHNHLTHCSL
Aotus_nancymaae           QLSKSIIKHSIDKSKSVIPNMDKNAVYKEGLPVPGEESKLTQEKSPTFSESHNHLTHCSL
Cebus_capucinus_imitator  QLSKSIIKHSIDKSKSVIPNVDKNAVYKEGLPAPGEESQLTLEKSPKFSESHNHLTHCSL
Saimiri_boliviensis       QLSKSIIKHSIDKSKSVIPNMDKKAVYKETLPVPGEESQLTPEKSPKFSESHSHLTHCSL
Macaca_nemestrina         QLSKSIIKHSIDKSKSMIPNIDKNAVYKESLPVSGEESQLTPEKSPKFPGSHNHLTHCSL
Papio_anubis              QLSKSIIKHSIDKSKSMIPNIDKNAVYKESLPVSGEESQLTPEKSPKFPDSHNHLTHCSL
Chlorocebus_sabaeus       QLSKSIIKHSIDKSKSVIPNIDKNAVYKESLPVSGEESQLTPEKSPKFPGSHNHLTHCSL
Homo_sapiens              RLSKSIIKHSIDKSKSVIPNIDKNAVYKESLPVSGEESQLTPEKSPKFPDSQNQLTHCSL
Pongo_abelii              RLSKSIIKHSIDKSKSVIPNIDKNAVYKESLPVSGEESQLTPEKSPKFPDSQNHLTHCSL
                          890123456789012345678901234567890123456789012345678901234567
                          840         5         6         7         8         9
```

```
Brachyteles_arachnoides   SAGKDCVPECKGSVAHGSSLDTLPSCPAVTGQKPDLKELDKDQPLKKPDLNNTSLEPLSF
Callithrix_jacchus        SAGKDCVPECKGSVAHVSSLESLPSCPAVTGQKPDLKELDKDQSLKKHNLNNTSLEPLSF
Aotus_nancymaae           SAGKDCVPECKGSVAHVSSLETLPSCPAVTGQKPDLKELNKDQPLKKHNLNNTSLEPLSF
Cebus_capucinus_imitator  PAGKDCVTECKGSVAHGSSLETLPSCPAVTGQKPDLKELDKDQTLKKHNLNNTSLEPLSF
Saimiri_boliviensis       SAGKDCVPECKGSVAHGSSLEMLPSCPAVTGQKPDLKELDKDQTLKKQNLNNTSLEPLSF
Macaca_nemestrina         SAGKDCVPECKVSVVHGSSLETLPSCPAVTGQKSDLKEPAKDQPLKKHNLNNTSLESLSF
Papio_anubis              SAGKDCVPECKVSMVHGSSLETLPSCPAVTGQKSDLKEPAKDQPLKKHNLNNTSLESLSF
Chlorocebus_sabaeus       SAGKDCVPECKVSMVHGSSLETLPSCPAVTGQKSDLKEPAKDQPLKKHNLNNTSLESLSF
Homo_sapiens              SAAKDCVPECKVSMVHGSSLETLPSCPAVTGQKSDLKESAKDQPLKKHNLNSTSLEALSF
Pongo_abelii              SAAKDCVPECKVSMVHGSSLETLPSCPAVTGQKSDLKESAKDQPLKKHNLNNTSLEPLSF
                          890123456789012345678901234567890123456789012345678901234567
                          900         1         2         3         4         5

Brachyteles_arachnoides   GQENPFPHSHTFSSTALTCVDGLHVEDKQKVRDRNVIPNTPPSTPLVPSQASSEWDIKKL
Callithrix_jacchus        GQENPFPHSHTFSSTALTCVDGLHVEDKQKVRDRNVIPDTPPSTPLVPSQASSEWDIKKL
Aotus_nancymaae           GQENPFPHSHTFSSTALTCVDGLHVEDKQKVRDRNVIPDTPPSTPLVPSQASSEWDIKKL
Cebus_capucinus_imitator  GQENTFPHSHTFSSTALTCVDGLHVEDKQKVRDRNIIPDTPPSTPLVPSQASSEWDIKKL
Saimiri_boliviensis       GQENPFPHSHTFSSTALTCVDGLHVEDKQKVRDRNIIPDTPPSTPLVPSQASSEWDIKKL
Macaca_nemestrina         GQENPFPHSHTFSSTALTCVDGLHVEDKQKVRDGNVIPDTPPSTPLVPSQASSEWDIKKL
Papio_anubis              GQENPFPHSHTFSSTALTCVDGLHVEDKQKVRDGNVIPDTPPSTPLVPSQASSEWDIKKL
Chlorocebus_sabaeus       GQENPFPHSHTFSSTALTCVDGLHVEDKQKVRDGNVIPDTPPSTPLVPSQASSEWDIKKL
Homo_sapiens              GQENPFPHSHTFSSTALTCVDGLHVEDKQKVRDRNVIPDTPPSTPLVPSRASSEWDIKKL
Pongo_abelii              GQENPFPHSHTFSSTALTCVDGLHVEDKQKVRDRNVIPDTPPSTPLVPSQASSEWDIKKL
                          890123456789012345678901234567890123456789012345678901234567
                          960         7         8         9      1000         1

Brachyteles_arachnoides   TKKLKGELAKEFAPATPPSTPHNSSVGSLSENEQNTIEKEEFMLKLMRSLSEEVESSESG
Callithrix_jacchus        TKKLKGELAKEFAPATPPSTPHNSSVGSLSENEQNTIEKEEFMLKLMRSLSEEVESSESG
Aotus_nancymaae           TKKLKGELAKEFAPATPPSTPHNSSVGSLSENEQNTIEKEEFMLKLMRSLSEEVESSESG
Cebus_capucinus_imitator  TKKLKGELAKEFAPATPPSTPHNSSVGSLSENEQNTIEKEEFMLKLMRSLSEEVESSESG
Saimiri_boliviensis       TKKLKGELAKEFAPATPPSTPHNSSVGSLSENEQNTIEKEEFMLKLMRSLSEEVESNESG
Macaca_nemestrina         TKKLKGELAKEFAPATPPSTPHNSSVGSLSENEQNTIEKEEFMLKLMRSLSEEVESSENG
Papio_anubis              TKKLKGELAKEFAPATPPSTPHNSSVGSLSENEQNTIEKEEFMLKLMRSLSEEVESSENG
Chlorocebus_sabaeus       TKKLKGELAKEFAPATPPSTPHNSSVGSLSENEQNTIEKEEFMLKLMRSLSEEVESSENG
Homo_sapiens              TKKLKGELAKEFAPATPPSTPHNSSVGSLSENEQNTIEKEEFMLKLMRSLSEEVESSESG
Pongo_abelii              TKKLKGELAKEFAPATPPSTPHNSSVGSLSENEQNTIEKEEFMLKLMRSLSEEVESSESG
                          890123456789012345678901234567890123456789012345678901234567
                         1020         3         4         5         6         7

Brachyteles_arachnoides   ELTEVDVKSEHSGKKVQFAEALATHILSLATEMAASHLDSRIIQEPKVKSPCLNVQSQRS
Callithrix_jacchus        ELPEVDVKSEHSGKKVQFAEALATHILSLATEMAASHLDSKIIQEPKVKSPYLNVQSQRN
Aotus_nancymaae           ELPEVDVKSEHSGKKVQFAEALATHILSLATEMAASHLDSKIIQEPKVQSPCLNVQSQRS
Cebus_capucinus_imitator  ELPEVDVKSEHSGKKVQFAEALATHILSLATEMAASHLHSKIIQEPKVKSPCLNVQSQRS
Saimiri_boliviensis       ELPEVDVKSEHSGKKVQFAEALATHILSLATEMAASHLDSKIIQEPKVKSPCLNVQSQRS
Macaca_nemestrina         ELPEVDVKSEHSGKKVQFAEALATHILSLATEMAASHLDNKIIQEPKVKSPCLNVQSQRS
Papio_anubis              ELPEVDVKSEHSGKKVQFAEALATHILSLATEMAASHLDNKIIQEPKVKSPCLNVQSQRS
Chlorocebus_sabaeus       ELPEVDVKSEHSGKKVQFAEALATHILSLATEMAASHLDNKIIQEPKVKSPCLNVQSQRS
Homo_sapiens              ELPEVDVKSEHSGKKVQFAEALATHILSLATEMAASHLDNKIIQEPKVKNPCLNVQSQRS
Pongo_abelii              ELPEVDVKSEHSGKKVQFAEALATHILSLATEMAASHLDNKIIQEPKVKNACLNVQSQRS
                          890123456789012345678901234567890123456789012345678901234567
                         1080         9    1100         1            2         3

                                         *
Brachyteles_arachnoides   VSPTFLNPSDENLKIR-NFAGDMAAEVITEAEKITKVRSCMLFKQKKNSCYVDGDQDYKV
Callithrix_jacchus        VSPTFLNPSHGNLKIL-NFAGDMAAEVITEAEKITKVQSCMLFKQKKNSCYVDGDQDYKV
Aotus_nancymaae           VSPTFLNPSDENLKILCNFAGDMAAEVITEAEKITKVRSCMLFKQKKNSCYVDGDQDYKV
Cebus_capucinus_imitator  ISPTFLNPSDENLKILCNFASDMAAEVITEAEKITKVRSCMLFKQKKNSCYVDGDQDYKV
Saimiri_boliviensis       VSPTFLNHSDENLKIL-NFAGDMAAEVITEAEKITKVRSCMLFKQKKNSCHADGGQDYKV
Macaca_nemestrina         VSPTFLNPSDENLKTLGSFAGDLAAEVITEAEKIAKVRSCMLFKQKKNRCYADGDQDYKV
Papio_anubis              VSPTFLNPSDENLKTLCSFAGDLAAEVITEAEKIAKVRSCMLFKQKKNRCYADGDQDYKV
Chlorocebus_sabaeus       VSPTFLNPSDENLKTLCSFAGDLAAEVITEAEKIAKVRSCMLFKQKKNRCYADGDQDYRV
Homo_sapiens              VSPTFLNPSDENLKTL-NFAGDLAAEVITEAEKIAKVRNCMLFKQKKNSCYADGDEDYKV
Pongo_abelii              VSPTFLNPSDENLKTLCNFAGDLAAEVITEAEKIAKVQNCMLFKQKKNSCYVDGDQDYKV
                          8901234567890123-45678901234567890123456789012345678901234 56
                         1140         5         6         7          8         9
```

```
Brachyteles_arachnoides      EEKLDLEAVAHPREVDSFILSLPPSSCMSGLTYKYPSCESVTDEYAGHVIQILKQEGGNS
Callithrix_jacchus           EEKLDLEAVAHPREVDPFILSLPPSSCMSGLTYKYPSCESVTDEYAGHIIQILKQEGGNS
Aotus_nancymaae              EEKLDLEAVAHPREVDPFILSLPPSSCMSGLTYKYPSCESVTDEYAGHIIQILKQEGGNS
Cebus_capucinus_imitator     EEKLDLEVVAHPREVDPFILSLPPSSCMSGLTYKYPSCESVTDEYAGHIIQILKQEGGNS
Saimiri_boliviensis          EERLDLEAVAHPREVDSFILSLPPSSCMSGLTYRYPSCESVTDEYAGHIIQILKQEGGNS
Macaca_nemestrina            EEKLDIEAVAHPREVDPFILSLPPSSCMSGLTYKYPSCESVTDEYAGHIIQILKQEGGNI
Papio_anubis                 EEKLDIEAVAHPREVDPFILSLPPSSCMSGLTYKYPSCESVTDEYAGHIIQILKQEGGNI
Chlorocebus_sabaeus          EEKLDIEAVAHPREVDPFILSLPPSSCMSGLTYKYPSCESVTDEYAGHIIQILKQEGGNI
Homo_sapiens                 EEKLDIEAVVHPREVDPFILSLPPSSCMSGLMYKYPSCESVTDEYAGHLIQILKQEGGNS
Pongo_abelii                 EEKLDIEAVVHPREVDPFILSLPPSSCMSGLMYKYPSCESVTDEFAGHIIQILKQEGGNS
                             789012345678901234567890123456789012345678901234567890123456
                             1200        1         2         3         4         5

                                                       *           *
Brachyteles_arachnoides      ELIMDQYANRLAYRSVKSGLQEAAKTSKVQCNSRMFPVASSQVKANKELLMFSNKEHHQE
Callithrix_jacchus           ELIMDQYANRLAYRSVKSGLQEAAKTTKVQCNSRMFPVPSSQVKANKELLMFSHKEHHQE
Aotus_nancymaae              ELIMDQYANRLAYRSVKSGLQEAAKTTKVQCNSRMFPVPSSQVKANKELLMFSNKEHHQE
Cebus_capucinus_imitator     ELIMDQYANRLAYRSVKSGLQEAAKTTKVKCNSRMFPVPSSQVKANKELLMFSNKEHHQE
Saimiri_boliviensis          ELIMDQYANRLAYRSVKSGLQEAAKTTKVQCNSRMFPVPSSQVKANKELLMFSNKEHHQE
Macaca_nemestrina            ELIMDQYANRLAYRSVKSGLQEAAKTTKVQCNSRMFPVPSSQVKTN-ELLMFSNKEHHQE
Papio_anubis                 ELIMDQYANRLAYRSVKSGLQEAAKTTKVQCNSRMFPVPSSQVKTN-ELLMFSNKEHHQE
Chlorocebus_sabaeus          ELIMDQYANRLAYRSVKSGLQEAAKTTKVQCNSRMFPVPSSQVKTN-ELLIFSNKEHHQE
Homo_sapiens                 ELIMDQYANRLAYRSVKSGLQEAAKTTKVQCNSRMFPVPSSQVKTNKELLMFSNKEHHQE
Pongo_abelii                 ELIMDQYANRLAYRSVKSGLQEASKTAKVQCNSRMFPVPSSRVKTNKELLMFSNKEHHQE
                             7890123456789012345678901234567890123456789012-3456789012345
                             1260        7         8          9      1300           1

Brachyteles_arachnoides      ADKKRQSKRNEGYFCKNQPFERTQDPCRNECSELYTFSTSLAHSITKDATEELTASLVGL
Callithrix_jacchus           ADKKRQSKRNEGYFCKNQPFERTQDPCRNECSELYTFSTSLAHSITKDATEELTASLVGL
Aotus_nancymaae              ADKK--SQRNEGYFCKNQPFERTQDPCRNECSELYTFSTSLAHSITKDATEELTASLVGL
Cebus_capucinus_imitator     ADKKRQSKRNEGYFCKNQPFERTQDPCRNECSELYTFSTSLAHSITKDATEELTASLVGL
Saimiri_boliviensis          ADKKRQSKRNEGYFCKNQPFERTQDPCRNECSELYTFSTSLAHSITKDATEELTASLVGL
Macaca_nemestrina            ADKKRQSKRNEDYFCKNQTCERTLDPCRNECSELYSFSASFVHSITKDAKEELTASLVGL
Papio_anubis                 ADKKRQSKRNEDYFCKNQTCERTLDPCRNECPELYSFSASFVHSITKDAKEELTASLVGL
Chlorocebus_sabaeus          ADKKRQSKRNEDYFCKNQTCERTLDPYRNECSELCSFSASFVHSITKDAKEELTASLVGL
Homo_sapiens                 ADKKRQSKRNEGYFCKNQTCERTLDPYRNEVSQLYSFSTSLVHSITKDAKEELTASLVGL
Pongo_abelii                 ADKKRQSKRNEGYFCKNQTCERTLDPYRNECSELYGFSTSLVHSITKDAEEELTASLVGL
                             67890--12345678901234567890123456789012345678901234567890123
                              1320            3         4         5         6         7

Brachyteles_arachnoides      PKSLTDSCLFEKSECEGGNECHVKPELPKSLQPPSQNHRFYHSTGSLNGYGCGENVVQAI
Callithrix_jacchus           PKSLTDSCLFEKSGCEGDNECHVKPELPKSLQPPSQNHRFYHSTGSLNGYGCAESVVQAI
Aotus_nancymaae              PKSLTDSCLFEKSGCEGDNKCPVKPELPKSLQPPSQNHRFYHSTGSLNGYGCGENVVQAI
Cebus_capucinus_imitator     PKSLTDSCLFEKSGCEGDNECHVKPELPKSLQPPSRNHRFYHSTGSLNGYGCGENVVQAI
Saimiri_boliviensis          PKSLTDSCLFEKSGCEEDNECHVKPELPKSLQPPSQSHRFYHSAGSLNGYGCGENVVQAI
Macaca_nemestrina            PKSSTDSCLFEKSGCEGDKECHVKPELPKSLQPSSQSHRFYHSTGSLNGYGCGDNIVQAV
Papio_anubis                 PKSSTDSCLFEKSGCEGDKECHVKPELPKSLQPSSPSHRFYHSTGSLNGYGCGDNIVQAV
Chlorocebus_sabaeus          PKSSTDSCLFQKSGCEGDKECHVKPELPKSLQPSSQSHRFYHSTGSLNGYGCGDSIVQAV
Homo_sapiens                 PKSLTDSCLFEKSGYEEDNECHVTPELPKSLQPSSQNHRFYHSTGSLNGYGCGDNVVQAV
Pongo_abelii                 PKSLTDSCLFEKSGCEEDNECHVTPELPKSLQPSSQNHRFYHSTDSLNGYGCGDNVVQAV
                             456789012345678901234567890123456789012345678901234567890123
                                1380          9    1400          1         2         3

Brachyteles_arachnoides      EQYAKKVVDDTLELTLGSTVFQVSETTKSADRVTYAEKLSPLIGQTCRYCDLKELHNCTG
Callithrix_jacchus           EQYAKKVVDDTLELTLGSTAFQVSETTKSADRVTYAEKLSPLIGQTCRYCDLKELHNCTG
Aotus_nancymaae              EQYAKKVVDDTLELTLGSTVFQVSETTKSADRVTYAEKLSPLIGQTCRYCDLKELHNCTG
Cebus_capucinus_imitator     EQYAKKVVDDTLELTLGSTVFQVSETTKSADGVTYAEKLSPLIGQTCRYCDLKELHNCTG
Saimiri_boliviensis          EQYAKKVVDDTLELTLGSTVFQVSETTKSADRVTYAEKLSPLIGQTCRYCDLKELHNCSG
Macaca_nemestrina            EEYAKKVVDDTLELTLGSTVLRVSETTKSADRVTYAEKLSPLTGQACRYCDLKELHNCTG
Papio_anubis                 EQYAKKVVDDTLELTLGSTVLRVSETTKSADRVTYAEKLSPLTSQACRYCDLKELHNCTG
Chlorocebus_sabaeus          EQYAKKVVDDTLELTLGSTVLRVSETTKSADRVTYAEKLSPLTGQACRYCDLKELHNCTG
Homo_sapiens                 EQYAKKVVDDTLELTLGSTVFRVSETTKSADRVTYAEKLSPLTGQACRYCDLKELHNCTG
Pongo_abelii                 EQYAKKVVDDTLELTLGSTVFRVSETTKSADRVTYAEKLSPLTGQACRYCDLKELHNCTG
                             456789012345678901234567890123456789012345678901234567890123
                                1440          5         6         7         8         9
```

```
                                                                                PKA-RII subunit binding
                                                         *                                   |-----------
Brachyteles_arachnoides     NSSQHFFRQGSLPSSKLASNPKFSNHSPKSRIFHLDVPQIHVNLDKKAVLAEKIVAEAIE
Callithrix_jacchus          NSSQHFFRQGSLPGSKLASNPTLSSRSPKSRIFHLDVPQIHVSLDKKAVLAEKIVAEAIE
Aotus_nancymaae             NSSQHFFKQGSLPSSKLASNPKFSSRSPKSRIFHLDVPQIHVNLDKKAVLAEKIVAEAIE
Cebus_capucinus_imitator    NSSQHFFRQGSLPSSKLASNPKFSSCSPKSRIFHLDVPQIHVNLDKKAVLAEKIVAEAIE
Saimiri_boliviensis         NSSQHFFRQGSLPGSKLASNPKFSSRSPKSRIFHLDVPQIHVNLDKKAVLAEKIVAEAIE
Macaca_nemestrina           NSSQNFFRQGSLASSKPASNPKFSSHYQKSRIFHLNVPQIHVNLDKKAVLAEKIVAEAIE
Papio_anubis                NSSQNFFRQGSLASSKPASNPKFSSHYQKSRIFHLSVPQIHVNLDKKAVLAEKIVAEAIE
Chlorocebus_sabaeus         NSSQNFFRQGSLASSKPASNPKFSSHYQKSRIFHLNVPQIHVNLDKKAVLAEKIVAEAIE
Homo_sapiens                NSSQHFFRQGSLASSKPASNPKFSSRYQKSRIFHLSVPQIHVNLDKKAVLAEKIVAEAIE
Pongo_abelii                SSSQHFFRQGSLASSKPASNPKFSSRYQKSRIFHLSVPQIHVNLDKKAVLAEKIVAEAIE
                            456789012345678901234567890123456789012345678901234567890123
                               1500         1         2         3         4         5

                            domain
                            ---|                                        |----------------
Brachyteles_arachnoides     KAERELSSASLAADSGVGQEGANFAESLATEIMTAAVTNVGHAVSSSKEIEDFQLTESFG
Callithrix_jacchus          KAERELSSTSLAADSGIGQEGASFAESLATEIMTAAVTNVGHAVSSSKEIEDFQLTESFG
Aotus_nancymaae             KAERELSSTSLAADSGIGQEGASFAESLATEIMTAAVTNVGHAVSSSKEIEDFQLTESFG
Cebus_capucinus_imitator    KAERELSSTSLAADSGIGQEGASFAESLATEIMTAAVTNVGHAVSSSKEIEDFQLTESFG
Saimiri_boliviensis         KAERELSSTSLAADSGIGQEGTSFAESLATEIMTAAVTNVGHAISSSKEVEDFQLTESFS
Macaca_nemestrina           KAEQEVSSVSLAADSGIGQEGASFAESLATEIMTAAVTNVGHVVNSSKEIEDFQSAESVG
Papio_anubis                KAEQEVSSVSLAADSGIGQEGASFAESLATEIMTAAVTNVGHVVSSSKEIEDFQSAESVG
Chlorocebus_sabaeus         KAEQEVSSVSLAADSGIGQEGASFAESLATEIMTAAVTNVGHAVSSSKEIEDFQSAESVG
Homo_sapiens                KAERELSSTSLAADSGIGQEGASFAESLATETMTAAVTNVGHAVSSSKEIEDFQSTESVS
Pongo_abelii                KAERELSSTSLAADSGIGQEGASFAESLATEIMTAAVTNVGHAVSSSKEIEDFQSTESVG
                            456789012345678901234567890123456789012345678901234567890123
                               1560         7         8         9      1600         1

                            ---------- Ser-rich ----------------|
Brachyteles_arachnoides     SQQMNLSIGDDSTGSWSNLSFEDEHQDESSSFHHLSESDEPDDKDEEHEDEVEGLGQDGK
Callithrix_jacchus          SQQMNLSIGDDSTGSWSNLSFEDEHQDESSSFHHLSESDEPDDKDEEHEDEVEGLGQAGK
Aotus_nancymaae             SQQMNLSIGDDSTGSWSNLSFEDEHQDESSSFHHLSESDDPDDKDEEHEDEVEGLGQDGK
Cebus_capucinus_imitator    SQQMNLSIGDDSTGSWSNLSFEDEHQDESSSFHHLSESDEPDDKDEEHEDEIEGLGQDGK
Saimiri_boliviensis         SQQMNLSIGDDSTGSWSNLSFEDEHQDESSSFHHLSESDEPDDKDEEQEDEVEGLGQDGK
Macaca_nemestrina           SQQMNLSIGDDSTGSWSNLSFEDEHQDESSSFHHLSESDGPDDKDEEHEDEVEGLGQDGK
Papio_anubis                SQQMNLSIGDDSTGSWSNLSFEDEHQDESSSFHHLSESDGPDDKDEEDEDEVEGLGQDGK
Chlorocebus_sabaeus         SQQMNLSIGDDSTGSWSNLSFEDEHQDESSSFHHLSESDGPDDKDEEHEDEVEGLGQDGK
Homo_sapiens                SQQMNLSIGDDSTGSWSNLSFEDEHQDESSSFHHLSESDGPDDKDEEHEDEVEGLGQDGK
Pongo_abelii                SQQMNLSIGDDSTGSWSNLSFEDEHQDESSSFHHLSESDGPDDKDEEHEDEVEGVGQDGK
                            456789012345678901234567890123456789012345678901234567890123
                               1620         3         4         5         6         7

Brachyteles_arachnoides     TLLITNIDMEPCTVDPQLRIILQWLVASEAEVAELYFHDSAKKEFMLLSKRLQEKGWKVG
Callithrix_jacchus          TLLITNIDMEPCTVDPQLRIILQWLVASEAEVAELHFHDSAKKELMLLSKRLQEKGWKVG
Aotus_nancymaae             TMLITNIDMEPCTVDPQLRIILQWLVASEAEVAELYFHDSAKKEFMLLSKRLQEKGWKVG
Cebus_capucinus_imitator    TLLITNIDMEPCTIDPQLRIILQWLVASEAEVTELYFHDSAKKEFMLLSKRLQEKGWKVG
Saimiri_boliviensis         TLLITNIDMEPCTVDPQLRIILQWLVASEAEVAELYFHDSAKKEFILLSKRLQEKGWKVG
Macaca_nemestrina           TLLITNIDMEPCTVDPQLRIILQWLIASEAEVTELYFHDSANKEFMLLSKQLQEKGWKVG
Papio_anubis                TLLITNIDMEPCTVDPQLRIILQWLIASEAEVAELYFHDSANKEFMLLSKQLQEKGWKVG
Chlorocebus_sabaeus         TLLITNIDMEPCTVDPQLRIILQWLIASEAEVAELYFHDSANKEFMLLSKQLQEKGWKVG
Homo_sapiens                TLLITNIDMEPCTVDPQLRIILQWLIASEAEVAELYFHDSANKEFMLLSKQLQEKGWKVG
Pongo_abelii                TLLITNIDMEPCAVDPQLRIILQWLIASEAEVAELYFHDSANKEFMLLSKQLQEKGWKVG
                            456789012345678901234567890123456789012345678901234567890123
                               1680         9      1700         1         2         3

Brachyteles_arachnoides     DLLQAVLQYYEVMENASSEERCKSLFDWLLENA
Callithrix_jacchus          DLLQAVLQYYEVMENASSEERCKSLFDWVLENA
Aotus_nancymaae             DLLQAVLQYYEVMENASSEERCKSLFDWLLENA
Cebus_capucinus_imitator    DLLQAVLQYYEVMENASSEERCKSLFDWLLEN-
Saimiri_boliviensis         DLLQAVLQYYEVMENASSEERCKSLFDWLLENA
Macaca_nemestrina           DLLQAVLQYYEVMEKASSEERCKSLFDWLLENA
Papio_anubis                DLLQAVLQYYEVMEKASSEERCKSLFDWLLENA
Chlorocebus_sabaeus         DLLQGVLQYYEVMEKASSEERCKSLFDWLLEN-
Homo_sapiens                DLLQAVLQYYEVMEKASSEERCKSLFDWLLENA
Pongo_abelii                DLLQAVLQYYEVMEKASSEERCKSLFDWLLE--
                            4567890123456789012345678901234--
                               1740         5         6
```

APOBEC3G

```
                                                      |-------------------------------
Brachyteles_arachnoides   MKPQTRNTVVRMDPDTFFYNFYNRPILSHRNTVWLCYEV--KTNDRSRPPLVANIFQGQV
Callithrix_jacchus        MKPQTRNTVVRMDPDTFFYNFYNRPILLHWNTVRLCYEV--KTNDCSRPPLVAKILQGQV
Aotus_nancymaae           MKPQTRNTVVRMDPDTFFYNFYNRPILSHRNIIWLCYEVKMKTNDLSRPPLVAKTFQGQV
Cebus_capucinus_imitator  -----RNTVVQMDPDTFVDDFYNRPILSDRNTVWLCYEVKMKTNDCSRPLLVANIFEGEV
Saimiri_boliviensis       MKPQTRNTVVRMDPDTFFYNFYNRPILSHRNTVWLCYEV--KTNDRSRPPLVAKIFQGQV
Macaca_nemestrina         MNPQIRNMVEPMDPRTFVSNFNNRPILSGLNTVWLCCEV--KTKDPSGPPLDAKIFQGKV
Papio_anubis              MKPQIRNMVKRMKADIFVSNFNNRPILSGRNTVWLCCEV--NTKDPSGPPLDAKIFRGKV
Chlorocebus_sabaeus       -NPQIRNMVERMKPGIFVYYFNNRPILSGRNIVWLCCEV--KTKDPSGPPLDANIFQGEL
Homo_sapiens              MKPHFRNTVERMYRDTFSYNFYNRPILSRRNTVWLCYEV--KTKGPSRPPLDAKIFRGQV
Pongo_abelii              MNPQFRNMVDGMDPHKFSYNFKNRPILSRRNTVWLCYEV--KTKGPSRPPLDAKIFRGQV
                               1234567890123456789012345678901234--5678901234567890123
                               0        1         2         3           4         5

                          ---------------- CMP/dCMP-type deaminase 1 -----------------
Brachyteles_arachnoides   YSKPQHHPEMRFLHWFRKWK-LRSDQEYEVTWFVSWSPCPVCARNVAEFLAEDRKVTLTI
Callithrix_jacchus        YSMPQHNPEMRFLHWFQKWK-LHSDQEYEVTWFVSWSPCPVCARNVAEFLAEDRKVTLTI
Aotus_nancymaae           YSKPQHHPEMRFLHWFRKWK-LHSDQEYEVTWFVSWSPCPVCARNVAEFLAEDRKVTLTI
Cebus_capucinus_imitator  YLKPQHHPEMRFLHWFRKWK-LHSDQEYEVTWFVSWSPCPMCARNMAKFLAEDRKVTLTI
Saimiri_boliviensis       YSKPQHHPEMRFLHWFRKWK-PHSDQEYEVTWFVSWSPCPVCARNVAEFLAEDGKVTLTI
Macaca_nemestrina         YSKAKYHPEMRFLRWFHKWRQLHHDQEYKVTWYVSWSPCTRCANSVATFLAKDPEVTLTI
Papio_anubis              YSKAKYHPEMRFLHWFRKWRQLHRDQEYEVTWYVSWSPCTGCANSVATFLAEDPKVTLTI
Chlorocebus_sabaeus       YPEAKDHPEMKFLHWFRKWRQLHRDQEYEVTWYVSWSPCTRCANSVATFLAEDPKVTLTI
Homo_sapiens              YSELKYHPEMRFFHWFSKWK-LHRDQEYEVTWYISWSPCTKCTRDMATFLAEDPKVTLTI
Pongo_abelii              YFELKNHPEMRFFHWFSKWRTLHRDQECEVTWYMSWSPCTKCTRNVATFLAEDPKVTLTI
                          45678901234567890123-456789012345678901234567890123456789012
                         54    6         7          8         9       100         1

                          --------------------|  **
Brachyteles_arachnoides   FVARLYYFWDPHYREELRRLCQKKKNPHATMKIMSYGEFQHCWDNFVDNQR-PFEPWNKL
Callithrix_jacchus        FVARLYYFWDLHYQEELHRLCQRRDSPHATMKIMSYGEFQHCWDKFMDNQR-LYKPWNKL
Aotus_nancymaae           FVARLYYFWDPHYQEGLRRLCQRRDSPHATMKIMSYGEFQHCWDKFVDNQR-LYKPGNKL
Cebus_capucinus_imitator  FVARLYYFWDPHYREGLRRLCQKKKNPHATMKIMSYGEFQHCWDNFVENQGEPFMPWNAL
Saimiri_boliviensis       FVARLYYFWDPHYWEELRRLCQRRDSPHATMKIMSYGEFQHCWDKFVDNQR-LYKPWNKL
Macaca_nemestrina         FVARLYYFWKPDYQQAFRILCQKRGGPPATMKIMNYNEFQDCWNKFVDGRGKPFKPWNNL
Papio_anubis              FVARLYYFWKPDYQEALRVLCQKRGSPHATMKIMNYNEFQHCWNKFVRGRREPFEPWENL
Chlorocebus_sabaeus       FVARLYYFWKPHYQEALRILCQKRGGPHATMKIMNYNEFQHCWNEFVDGQGKPFKPRKNL
Homo_sapiens              FVARLYYFWDPDYQEALRSLCQKRDGPRATMKIMNYDEFQHCWSKFVYSQR-LFEPWNNL
Pongo_abelii              FVARLYYFWDPDYQEALRSLCRERDGPRANMKIMNYDEFQHCWNKFVYSQRELFEPWNNL
                          345678901234567890123456789012345678901234567890123-45678901
                        113     2         3         4         5         6          7

                                                            |-------------------------
Brachyteles_arachnoides   PKHYTLLRILLGEVLRHLMDPVTFTYNFTNDPLVLGQHQTYLCYKVEHLRNGTWVPLDQH
Callithrix_jacchus        TKHYTVLHIILGEVLGHLMDPVTFTYNFTNDPSVFGQHQTYLCYEVEHLHNGTWVLLHQH
Aotus_nancymaae           PKHYTLLHITLGEVLRHLMDPDTFTYNFTNDPSVLGRHQTYLCYEVEHLHNGTWVPLHQH
Cebus_capucinus_imitator  PKHYTLLRITLGEVLRHLMDPGTFAYNFTNDPSVLGQHQTYLCYKVEHLNSGTWVPLHQH
Saimiri_boliviensis       PKHYTLLHITLGEVLGHLMDPGTFSYNFTNDPSVLGRHQTYLCYEVEHVHNGTWVPMHQH
Macaca_nemestrina         PKHYTLLQATLGELLRHLMDPGTFTSNFNNKPWVSGQHETYLCYKVERLHNDTWVPLNQH
Papio_anubis              PKHYTLLHATLGELLRHLMDPGTFTSNFYNKPWVSGQHETYLCYKVERLHNGTWVPLNQH
Chlorocebus_sabaeus       PKHYTLLHATLGELLRHVMDPGTFTSNFNNKPWVSGQRETYLCYKVERWHNDTWVLLNQH
Homo_sapiens              PKYYILLHIMLGEILRHSMDPPTFTFNFNNEPWVRGRHETYLCYEVERMHNDTWVLLNQR
Pongo_abelii              PKYYIVLHIILGEILRHSMDPLTFTSNFNNEPCVEGRHETYLCYKVERLHNDTWVLLNQR
                          234567890123456789012345678901234567890123456789012345678901
                        172      8         9       200         1         2         3
```

```
                          -- CMP/dCMP-type deaminase 2 ------------------------------
                                            |-------- zinc-binding ----------------|
Brachyteles_arachnoides   RGFILNEASNNPGLPEGRHAELCLLDLISFWKLDLAQRYRVTCFISWSPCFSCAEKVAEF
Callithrix_jacchus        RGFILNQASNNPGFPEGRHAELCLLDLISFWKLDLAQYYTVTCFISWSPCFSCAEKVAEF
Aotus_nancymaae           RGFILNEASNNPGFPEGRHAELCLLDLISFWKVDLAQHYRVTCFISWSPCFSCAEKVAEF
Cebus_capucinus_imitator  RGFILNEASNNPGFPEARHAELCLLDLISFWKLDPAQRYRVTCFISWSPCFSCARKVAEF
Saimiri_boliviensis       RGFILNEASNSLGFPEGRHAELCLLDLISFWKLDLAQRYRVTCFISWSPCFSCAEKVAEF
Macaca_nemestrina         RGFLRNQAPNIHGFPKGRHAELCFLDLIPFWKLD-GQQYRVTCFTSWSPCFSCAQEMAKF
Papio_anubis              RGFLRNQAPDIHGFPKGRHAELCFLDLIPFWKLD-GQQYRVTCFTSWSPCFSCAQEMAKF
Chlorocebus_sabaeus       RGFLRNQAPDRHGFPKGRHAELCFLDLIPFWKLD-DQQYRVTCFTSWSPCFSCAQKMAKF
Homo_sapiens              RGFLCNQAPHKHGFLEGRHAELCFLDVIPFWKLDLDQDYRVTCFTSWSPCFSCAQEMAKF
Pongo_abelii              RGFLCNQAPAIHGFPEGRHAELCFLDVIPFWKLDGKQRYRVTCFTSWSPCFRCAQEMAKF
                          2345678901234567890123456789012345-6789012345678901234567890
                        232       4         5         6          7         8         9

                          -----------------------------|
                                             **      *  *         *
Brachyteles_arachnoides   LQENPHVNLRIFAARIYDYRPGYEEGLQMLQNAGAQVSIMTSDDFRHCWDTFVDHQGHPF
Callithrix_jacchus        LQENPHVNLHIFAAHIYGYQRGYIKGLCRLNRAGAPISMMKYSEFSYCWDTFVDHQEHPF
Aotus_nancymaae           LQQNPHVNLRIFAARIYNYQRGYKKGLRRLDRAGAPISMMKYSEFRHCWDTFVDHQGHLF
Cebus_capucinus_imitator  LQQNPHVNLRIFAARIYDYHPGYEEGLCRLGWAGAPISMMKYSEFRHCWDTFVDHQGCRF
Saimiri_boliviensis       LQENPHVNLHIFAARIYDCQRGYKKGLRRLDRARAPISMMKYSEFRYCWDTFVDHQGHPF
Macaca_nemestrina         ISNNEHVSLCIFAARIYDDQGRYQEGLRTLHRDGAKIAMMNYSEFEYCWDTFVDRQGRPF
Papio_anubis              ISNNEHVSLCIFAARIYDDQGRCQEGLRTLHRDGAKIAMMNYSEFEYCWDTFVDRQGRPF
Chlorocebus_sabaeus       ISKNKHVSLCIFAARIYDDQGRCQEGLRTLHRDGAKIAVMNYSEFEYCWDTFVDRQGRPF
Homo_sapiens              ISKNKHVSLCIFTARIYDDQGRCQEGLRTLAEAGAKISIMTYSEFKHCWDTFVDHQGCPF
Pongo_abelii              ISNNQHVSLCIFAARIYDDQGRCKEGLRTLDEAEAKISIMTYSEFQHCWDTFVDHQGRPF
                          123456789012345678901234567890123456789012345678901234567890
                        291      300         1         2         3         4         5

Brachyteles_arachnoides   QPWEGLDEHSQALSGRLQAILQGN
Callithrix_jacchus        QPWEGLDEYTQALSGKLQAILQGN
Aotus_nancymaae           KPWEGLNEHSQALSGRLQAILQ--
Cebus_capucinus_imitator  KPWKGLNEHSQALSRRLQAILQ--
Saimiri_boliviensis       QPWEGLDEHSQALSGRLQAILQGN
Macaca_nemestrina         QPWDGLDEHSQALSGRLQAILQ--
Papio_anubis              QPWDGLDEHSQDLSGRLRAILQ--
Chlorocebus_sabaeus       QPWDGLDEHSQALSGRLRAILQ--
Homo_sapiens              QPWDGLDEHSQDLSGRLRAILQEN
Pongo_abelii              QPWDGLEEHSEAWSGKLQAILQ--
                          1234567890123456789012--
                        351        6         7
```

BMS1

```
                              *
Brachyteles_arachnoides   MEAKEQKKHRKKNSGPKAAKKKKRHLQDLQLGDEEDARKRNPKAFAVQSAVRMARSFHRT
Callithrix_jacchus        MEAKDQKKHRKKNSGPKAEKKKKRHLQDLQLGDEEDARKRNPKAFAVQSAVRMARSFHRT
Aotus_nancymaae           MEAKDQKKHRKKNSGPKAEKKKKRHLQALQLGDEEDARKRNPKAFAVQSAVRMARSFHRT
Saimiri_boliviensis       MEAKDQKKHRKKNSGPKAEKKKKRHLQDLQLGDEEDARKRNPKAFAVQSAVRMARSFHRT
Chlorocebus_sabaeus       METKDQKKHRKKNSGPKAEKKKKRHLQDLQLGDEEDARKRNPKAFAVQSAVRMARSFHRT
Homo_sapiens              MEAKDQKKHRKKNSGPKAAKKKKRLLQDLQLGDEEDARKRNPKAFAVQSAVRMARSFHRT
Pongo_abelii              MEAKDQKKHRKKNSGPKAAKKKKRHLQDLQLGDEEDARKRNPKAFAVQSAVRMARSFHRT
Otolemur_garnetti         METKDQKKHRKKNSGPKAEKKKKRHLQDLQLGDEEDARKRNPKAFSVQSAVRMARSFHRT
                          123456789012345678901234567890123456789012345678901234567890
                                   1         2         3         4         5         6

                                             |-----------------------------------------
Brachyteles_arachnoides   QDLKTKKHHIPVVDRTPLEPPPIVVVVMGPPKVGKSTLIQCLIRNFTRQKLTEIRGPVTI
Callithrix_jacchus        QDLKTKKHHIPVVDRTPLEPPPIVVVVMGPPKVGKSTLIQCLIRNFTRQKLTEIRGPVTI
Aotus_nancymaae           QDLKTKKHHIPVVDRTPLEPPPIVVVVMGPPKVGKSTLIQCLIRNFTRQKLTEIRGPVTI
Saimiri_boliviensis       QDLKTKKHHIPVVDRTPLEPPPIVVVVMGPPKVGKSTLIQCLIRNFTRQKLTEIRGPVTI
Chlorocebus_sabaeus       QDLKTKKHHIPVVDRTPLEPPPIVVVVMGPPKVGKSTLIQCLIRNFTRQKLTEIRGPVTI
Homo_sapiens              QDLKTKKHHIPVVDRTPLEPPPIVVVVMGPPKVGKSTLIQCLIRNFTRQKLTEIRGPVTI
Pongo_abelii              QDLKTKKHHIPVVDRTPLEPPPIVVVVMGPPKVGKSTLIQCLIRNFTRQKLTEIRGPVTI
Otolemur_garnetti         QDLKTKKHHIPVVDRTPLEPPPIVVVVMGPPKVGKSTLIQCLIRNFTRQKLTEIRGPVTI
                          123456789012345678901234567890123456789012345678901234567890
                         61        7         8         9        100        1         2

                                 Bms1-type guanine nucleotide-binding (G) domain
                          ------------------------------------------------------------
Brachyteles_arachnoides   VSGKKRRLTIIECGCDINMMIDLAKVADLVLMLIDASFGFEMETFEFLNICQVHGFPKIM
Callithrix_jacchus        VSGKKRRLTIIECGCDINMMIDLAKVADLVLMLIDASFGFEMETFEFLNICQVHGFPKIM
Aotus_nancymaae           VSGKKRRLTIIECGCDINMMIDLAKVADLVLMLIDASFGFEMETFEFLNICQVHGFPKIM
Saimiri_boliviensis       VSGKKRRLTIIECGCDINMMIDLAKVADLVLMLIDASFGFEMETFEFLNICQVHGFPKIM
Chlorocebus_sabaeus       VSGKKRRLTIIECGCDINMMIDLAKVADLVLMLIDASFGFEMETFEFLNICQVHGFPKIM
Homo_sapiens              VSGKKRRLTIIECGCDINMMIDLAKVADLVLMLIDASFGFEMETFEFLNICQVHGFPKIM
Pongo_abelii              VSGKKRRLTIIECGCDINMMIDLAKVADLVLMLIDASFGFEMETFEFLNICQVHGFPKIM
Otolemur_garnetti         VSGKKRRLTIIECGCDINMMIDLAKVADLVLMLIDASFGFEMETFEFLNICQVHGFPKIM
                          123456789012345678901234567890123456789012345678901234567890
                        121        3         4         5         6         7         8

                          ------------------------------------------------------------
                                                                       |----------
Brachyteles_arachnoides   GVLTHLDSFKHNKQLKKTKKRLKHRFWTEVYPGAKLFYLSGMVHGEYQNQEIHNLGRFIT
Callithrix_jacchus        GVLTHLDSFKHNKQLKKTKKRLKHRFWTEVYPGAKLFYLSGMVHGEYQNQEIHNLGRFIT
Aotus_nancymaae           GVLTHLDSFKHNKQLKKTKKRLKHRFWTEVYPGAKLFYLSGMVHGEYQNQEIHNLGRFIT
Saimiri_boliviensis       GVLTHLDSFKHNKQLKKTKKRLKHRFWTEVYPGAKLFYLSGMVHGEYQNQEIHNLGRFIT
Chlorocebus_sabaeus       GVLTHLDSFKHNKQLKKTKKRLKHRFWTEVYPGAKLFYLSGMVHGEYQNQEIHNLGRFIT
Homo_sapiens              GVLTHLDSFKHNKQLKKTKKRLKHRFWTEVYPGAKLFYLSGMVHGEYQNQEIHNLGRFIT
Pongo_abelii              GVLTHLDSFKHNKQLKKTKKRLKHRFWTEVYPGAKLFYLSGMVHGEYQNQEIHNLGRFIT
Otolemur_garnetti         GVLTHLDSFKHNKQLKKTKKRLKHRFWTEVYPGAKLFYLSGMVHGEYQNQEIHNLGRFIT
                          123456789012345678901234567890123456789012345678901234567890
                        181        9        200        1         2         3         4

                          ------|
                          -------------- AARP2CN (NUC121) domain ---------------------
Brachyteles_arachnoides   VMKFRPLTWQTSHPYILADRMEDLTNPEDIRTNIKCDRKVSLYGYLRGAHLKNKSQIHMP
Callithrix_jacchus        VMKFRPLTWQTSHPYILADRMEDLTNPEDIRTNIKCDRKVSLYGYLRGTHLKNKSQIHMP
Aotus_nancymaae           VMKFRPLTWQTSHPYILADRMEDLTNPEDIRTNIKCDRKVSLYGYLRGAHLKNKSQIHMP
Saimiri_boliviensis       VMKFRPLTWQTSHPYILADRMEDLTNPEDIRRNIKCDRKVSLYGYLRGAHLKNKSQIHMP
Chlorocebus_sabaeus       VMKFRPLTWQTSHPYILADRMEDLTNPEDIRTNIKCDRKVSLYGYLRGAHLKNKSQIHMP
Homo_sapiens              VMKFRPLTWQTSHPYILADRMEDLTNPEDIRTNIKCDRKVSLYGYLRGAHLKNKSQIHMP
Pongo_abelii              VMKFRPLTWQTSHPYILADRMEDLTNPEDIRTNIKCDRKVSLYGYLRGAHLKNKSQIHMP
Otolemur_garnetti         VMKFRPLTWQTSHPYILADRMEDLTNPEDIRTNIKCDRKVSLYGYLRGAHLKNKSQIHMP
                          123456789012345678901234567890123456789012345678901234567890
                        241        5         6         7         8         9        300
```

```
                         -----------------|
Brachyteles_arachnoides  GVGDFTVSDVSFLPDPCALPEQQKKRCLNEKEKLVYAPLSGVGGVLYDKDAVYVDLGGSH
Callithrix_jacchus       GIGDFTVSDVSFLPDPCALPEQQKKRCLNEKEKLVYAPLSGVGGVLYDKDAVYVDLGGSH
Aotus_nancymaae          GVGDFTVSDVSFLPDPCALPEQQKKRCLNEKEKLVYAPLSGVGGVLYDKDAVYVDLGGSH
Saimiri_boliviensis      GVGDFTVSDVSFLPDPCALPEQQKKRCLNEKEKLVYAPLSGVGGVLYDKDAVYVDLGGSH
Chlorocebus_sabaeus      GVGDFAVSDISFLPDPCALPEQQKKRCLNEKEKLVYAPLSGVGGVLYDKDAVYVDLGGSH
Homo_sapiens             GVGDFAVSDISFLPDPCALPEQQKKRCLNEKEKLVYAPLSGVGGVLYDKDAVYVDLGGSH
Pongo_abelii             GVGDFAVSDISFLPDPCALPEQQKKRCLNEKEKLVYAPLSGVGGVLYDKDAVYVDLGGSH
Otolemur_garnetti        GVGDFAVSDVSFLPDPCALPEQQKKRCLNEKEKLVYAPLSGVGGVLYDKDAVYVDLGGSH
                         123456789012345678901234567890123456789012345678901234567890
                       301         1         2         3         4         5        6

Brachyteles_arachnoides  GFQ--DEVGSTHELVQSLISTHSTIDAKMASSRVMLFSDSKALGSEDIDNQGLLMPKEEK
Callithrix_jacchus       GFQ--DEVGSTHELVQSLISTHSTIDAKMASSRVMLFSDSKALGSEDIDNQGLLMPKEEK
Aotus_nancymaae          GFQALDEVGSTHELVQSLISTHSTIDAKMASSRVMLFSDSKALGSEDIDNQGLLMLKEEK
Saimiri_boliviensis      GFQ--DEVGSTHELVQSLISTHSTIDAKMASSRVMLFSDSKALGSEDIDNQGLLIPKEEK
Chlorocebus_sabaeus      GFQ--DEVGPTHELVQSLISTHSTIDAKMASSRVTLFSDSKPLGSEDLDNQGLLMPKEEK
Homo_sapiens             VFQ--DEVGPTHELVQSLISTHSTIDAKMASSRVTLFSDSKPLGSEDIDNQGLMMPKEEK
Pongo_abelii             GFQ--DEVGPTHELVQSLISTHSTIDAKMASSRVTLFSDSKPLGSEDIDNQGLMMPKEEK
Otolemur_garnetti        GFQAADEVGPTHELVQSLISTHSTIDAKMASSRVTLFSDSKPLGSEDIDNQGLWMPKEEK
                         123--4567890123456789012345678901234567890123456789012345678
                       361           7         8         9      400          1

Brachyteles_arachnoides  QMDLKTGRMRRKAIFSD-EDGSGDSDDEEDDEMSEDDGLENGSSDEEADEEENAEVTD--
Callithrix_jacchus       QMDLKTGRMRRKAVFGD-EDGSGDSDDEEGDEMSEDDGLENGSSDEEAVEEENAEVTE--
Aotus_nancymaae          QMDLKTGRMRRKAIFGD-EDGSGDSDDEDDDEMSEDDGLEYGSSDEEAEEEENAEVTD--
Saimiri_boliviensis      QMDLKTGRMRRKAIFGD-EDGSGDSDDEEDDEMSEDDGLENGSSDEEAEEEENAEVTD--
Chlorocebus_sabaeus      QMDLKTGRMRRKAIFGD-EDESGDSDDEENDEISEDDLLENGSSDEEAEEEENAEMTD--
Homo_sapiens             QMDLNTGRMRRKAIFGD-EDESGDSDDEEDDEMSEDDGLENGSSDEEAEEEENAEMTD--
Pongo_abelii             QMDLKTGRMRRKAIFGD-EDEPGDSDDEEDDEVSEDDGLENGSSDEEAEEEENAEMTD--
Otolemur_garnetti        QMDVKTGRVRRKAIFGDEEDESGESDDEDDDEMSAGDRSENDSSDDNTEEEEDAKMTDKS
                         90123456789012345-6789012345678901234567890123456789012345--
                        420          3          4         5         6         7

                                                            *
Brachyteles_arachnoides  ECVTAKGVKRRKL-ELEEDSEVDLPAFADSDDDLEESSAEEGEVEGAGESSEEEDRSA-G
Callithrix_jacchus       ECMTAKGVKRRKL-ELEEDSEVDLPAFADSDDDLERSSAEEGEAEEADESSEEEDCSA-G
Aotus_nancymaae          EYMIAKGIKRRKLEELEEDSEVDLPAFADSDDDLEKSSAEEGEVEEADESSEEEDCSS-G
Saimiri_boliviensis      ECMTAKGIKRRKL-ESEEDSEVDLPAFADSDDDLERSSGEEGEVEEADESSEGEDCSA-G
Chlorocebus_sabaeus      QYMAGKGVKRRKLEELEEDSEMDLPAFADSDDDLERSSVEEGEAEEADESSEEEDCTA-G
Homo_sapiens             QYMAVKGIKRRKL-ELEEDSEMDLPAFADSDDDLERSSAEEGEAEEADESSEEEDCTA-G
Pongo_abelii             QYMAIKGVKRRKLEELEEDGEMDLPAFADSDDDLERSSAEEGEAEEADESSEEEDCTA-G
Otolemur_garnetti        KYRTVKGVKRQKLEGLEEDSDLDLPAFADSDDDLERSSGEEGEVEEAEESSEEEDVTTGG
                         6789012345678-90123456789012345678901234567890123456789012-3
                       476    8          9      500          1         2         3

                                       *
Brachyteles_arachnoides  ERGISGSKAVGEGSDAELSPANRQSDRVNLEKSLLMKKAALTTSDSGHCTAEEVFASEDE
Callithrix_jacchus       EKGISGSKAVREGSKAELSPANHQRDRVNLEKSLLMKKAALTTSDSGHCTAEEVFASEDE
Aotus_nancymaae          ERGISGSKAVGEGSKAELSPSNHQSDPVNLEKSLPMKKAALTTSDSGHCTAEEVFASEDD
Saimiri_boliviensis      ERGISGSKAVGEGSKAELSPANHQSDRVNLEKSLPMKKAALTTSDSGHCTAEEAFASEDE
Chlorocebus_sabaeus      EGGISGSKAVGEGSEAGLSPANCRSDRVDLEKSLLMKKAALMTSDSGHCTAEEAFASEDE
Homo_sapiens             EKGISGSKAAGEGSKAGLSPANCQSDRVNLEKSLLMKKAALPTFDSGHCTAEEVFASEDE
Pongo_abelii             EKGISGSKAAGEGSKAGLSPANCQSDRVNLEKSLLMKKAALPTFDSGHCTAEEVFASEDE
Otolemur_garnetti        GRDILELKAVEESGKTGSLPASHWGDSVNLEKSLPLEKAALPTSDSGHCTAEEVFASEDE
                         456789012345678901234567890123456789012345678901234567890123
                       534      4         5         6         7         8         9
```

```
                                                                       *
Brachyteles_arachnoides  SEESSSLSTEEEDSQNQEALRKKLSKPSQVGSGQKLGSRNLTDETSDQEDLLKEEEDYKE
Callithrix_jacchus       SEESSSLSAEEEDSENQEALRKKLLKPSQVGSGQKLGSRNLIDETSDIEDLLKEEEDYKE
Aotus_nancymaae          SEESSSLSIEEENSENQEALRKKLLKPSQVGSGQKLGPRNFIDETSDIEDLLKEEEDYKE
Saimiri_boliviensis      SEESSSLSTEEEDSENQEALRKKLSKPSQVGSGQKRGSRNLIDETSDIEDLLKEEEDYKE
Chlorocebus_sabaeus      SEESSSLGAEEEDSENEEAIRKKLSKPSQVSSGQKLGPRNLIDETSDIEDLLKEEEDYKE
Homo_sapiens             SEESSSLSAEEEDSENEEAIRKKLSKPSQVSSGQKLGPQNFIDETSDIENLLKEEEDYKE
Pongo_abelii             SEESSSLSAEEEDSENEEAIRKKLSKPSQVSSGQKLGPRNLIDETSDIENLLKEEEDYKE
Otolemur_garnetti        SEGSSSLSSEEEDSANEDVIRKKLPKPSQVGSRQKQGSENLINENSDIEDLLKEEEDYKD
                         456789012345678901234567890123456789012345678901234567890123
                       594   600         1         2         3         4         5
```

```
Brachyteles_arachnoides  ERNDPTETSGALKWKEDLSRKAAEAFLRQQQAAPNLRKLIYGTVTEDNEEDD-DTQEELG
Callithrix_jacchus       ERNDPTETSGALKWKEDLSRKAAEAFLRQQQAAPNLRKLIYGTVTEDNEEED-DTQEELG
Aotus_nancymaae          EKNDSTETSGALKWKEDLSRKAAEAFLRQQQAAPNLRKLIYGTVTEDNEEDD-DTQEELG
Saimiri_boliviensis      EKNDPTETSGALKWKEDLSRKAAEAFLRQQQAAPNLRKLIYGTVTEDNEEDD-DTREELG
Chlorocebus_sabaeus      ENNDSKETSGALKWKEDLSRKAAEAFLRQQQAAPNLRKLIYGTVAEDNEEEDGDTREELG
Homo_sapiens             ENNDSKETSGALKWKEDLSRKAAEAFLRQQQAAPNLRKLIYGTVTEDNEEDD-DTLEELG
Pongo_abelii             ENNDSKETSGALKWKEDLSRKAAEAFLRQQQAAPNLRKLIYGTVTEDNEEEDGDTREELG
Otolemur_garnetti        ENNCTTETSGALKWKEDLSRKAAEAFLRQQQTTPNLRKLIYGTVTEDNEEEDGESGEELG
                         45678901234567890123456789012345678901234567890145-6789012
                       654     6         7         8         9       700          1
```

```
                                                                              *
Brachyteles_arachnoides  GLFRVNQPDRGCKHKADSLDCSRFLVEAPHDWDLEEVVNSIRDCFVTGKWEDDTDAAKVL
Callithrix_jacchus       GLFRVTQPDRGCKHKADSLDCSRFPVEAPHDWDLEEVMNSIRDCFVTGKWEDDKDAAKVL
Aotus_nancymaae          GLFRVNQPDRACKHKADSLDCSRFLVEAPHDWDLEEVMNSIRDCFVTGKWEDDKDAAKVL
Saimiri_boliviensis      GLFRVTQPDRECKHKADSLDCSRFLVEAPHDWDLEEVMNSIRDCFVTGKWEDDKDAAKVL
Chlorocebus_sabaeus      GLFRVNQPDRECKHKADSLDCSRFLVEAPHDWDLEEVMNSIRDCFVTGKWEDDKDAAKVL
Homo_sapiens             GLFRVNQPDRECKHKADSLDCSRFLVEAPHDWDLEEVMNSIRDCFVTGKWEDDKDAAKVL
Pongo_abelii             GLFRVNQPDRECKHKADSLDCSRFLVEAPHDWDLEEVMNSIRDCFVTGKWEDDKDAAKVL
Otolemur_garnetti        GLFRVNQPDRECKHKADSLDCSRFPVEAPHDWDLEEVMNSIRDCFVTGKWEDDKDAAKIL
                         345678901234567890123456789012345678901234567890123456789012
                       713      2         3         4         5         6         7
```

```
                                                           **      |-----------------
Brachyteles_arachnoides  AEDEELYGNFEDLETGDVHKGKSGPITQNEDVEKEAQEEIDPSEEESAKKKHLDKKRKLK
Callithrix_jacchus       AEDEELYGDFEDLETGDVHKGKSGPITQNEDVEKEVKEEIDPNEEESAKKKHLDKKRKLK
Aotus_nancymaae          AEDEELYGDFEDLETGDVHKGKSGPITQNEDVEKEVKEEIDPNEEESAKKKHLDKKRKLK
Saimiri_boliviensis      AEDEELYGDFEDLETGDVHKGKSGPITQNEDVEKEVKEEIDPNEEESAKKKHLDKKRKLK
Chlorocebus_sabaeus      AEDEELYGDFEDLETGDVHKGKSGPDTQNEDVEKEVKEEIDPNEEESAKKKHLDKKRKLK
Homo_sapiens             AEDEELYGDFEDLETGDVHKGKSGPNTQNEDIEKEVKEEIDPDEEESAKKKHLDKKRKLK
Pongo_abelii             AEDEELYGDFEDLETGDVHKGKSGPNTQNEDIEKEVKEEIDPDEEESAKKKHLDKKRKLK
Otolemur_garnetti        AEDDELYGDFEDLETGDVHKGKSGQDIEIDDVEKEVKEEIDPDAEESAKKKHLDKKRKLK
                         345678901234567890123456789012345678901234567890123456789012
                       773      8         9       800         1         2         3
```

```
                           40S ribosome biogenesis protein Tsr1 and BMS1 C-terminal
                         ------------------------------------------------------------
                                                                               *
Brachyteles_arachnoides  EMFDAEYDEGESTYFDDLKGEMQKQAQLNRAEFEDQDDEARVQYEGFRPGMYVRIEIENV
Callithrix_jacchus       EMFDAEYDEGESTYFDDLKGEMQKQAQLNHAEFEDQDDEARVQYEGFRPGMYVRVEIENV
Aotus_nancymaae          EMFDAEYDEGESTYFDDLKGEMQKQAQLNRAEFEDQDDEARVQYEGFRPGMYVRVEIENV
Saimiri_boliviensis      EMFDAEYDEGESTYFDDLKGEMQKQAQLNRAEFEDQDDEARVQYEGFRPGMYVRVEIENV
Chlorocebus_sabaeus      EMFDAEYDEGESTYFDDLKGEMQKQAQLNRTEFEDQDDEARVQYEGFRPGMYVRVEIENV
Homo_sapiens             EMFDAEYDEGESTYFDDLKGEMQKQAQLNRAEFEDQDDEARVQYEGFRPGMYVRIEIENV
Pongo_abelii             EMFDAEYDEGESTYFDDLKGEMQKQAQLNRAEFEDQDDEARVQYEGFRPGMYVRVEIENV
Otolemur_garnetti        EMFDAEYDEGESTYFDDLKGEMQKQAQLNRAEFEDQDDEARVQYEGFRPGMYIRIEIENV
                         345678901234567890123456789012345678901234567890123456789012
                       833      4         5         6         7         8         9
```

```
                           40S ribosome biogenesis protein Tsr1 and BMS1 C-terminal
                          ------------------------------------------------------------
Brachyteles_arachnoides   PCEFVQNFDPHYPIILGGLGNSEGNVGYVQMRLKKHRWYKKILKSRDPIIFSVGWRRFQT
Callithrix_jacchus        PCEFVQNFDPHYPIILGGLGNSEGNVGYVQMRLKKHRWYKKILKSRDPIIFSVGWRRFQT
Aotus_nancymaae           PCEFVQNFDPHYPIILGGLGNSEGNVGYVQMRLKKHRWYKKILKSRDPIIFSVGWRRFQT
Saimiri_boliviensis       PCEFVQNFDPHYPIILGGLGNSEGNVGYVQMRLKKHRWYKKILKSRDPIIFSVGWRRFQT
Chlorocebus_sabaeus       PCEFVQNFDPHYPIILGGLGNSEGNVGYVQMRLKKHRWYKKILKSRDPIIFSVGWRRFQT
Homo_sapiens              PCEFVQNFDPHYPIILGGLGNSEGNVGYVQMRLKKHRWYKKILKSRDPIIFSVGWRRFQT
Pongo_abelii              PCEFVQNFDPHYPIILGGLGNSEGNVGYVQMRLKKHRWYKKILKSRDPIIFSVGWRRFQT
Otolemur_garnetti         PCELVLNFDPHYPIILGGLGNSEGNVGYVQMRLKKHRWYKKILKSRDPIIFSVGWRRFQT
                          345678901234567890123456789012345678901234567890123456789012
                        893    900       1         2         3         4         5

                          ----------------------------------------------------|
Brachyteles_arachnoides   IPLYYIEDHNGRQRLLKYTPQHMHCGATFWGPITPQGTGFLAIQSVSGIMPDFRIAATGV
Callithrix_jacchus        IPLYYIEDHNGRQRLLKYTPQHMHCGATFWGPITPQGTGFLAIQSVSGIMPDFRIAATGV
Aotus_nancymaae           IPLYYIEDHNGRQRLLKYTPQHMHCGATFWGPITPQGTGFLAIQSVSGIMPDFRIAATGV
Saimiri_boliviensis       IPLYYIEDHNGRQRLLKYTPQHMHCGATFWGPITPQGTGFLAIQSVSGIMPDFRIAATGV
Chlorocebus_sabaeus       IPLYYIEDHNGRQRLLKYTPQHMHCGAAFWGPITPQGTGFLAIQSVSGIMPDFRIAATGV
Homo_sapiens              IPLYYIEDHNGRQRLLKYTPQHMHCGAAFWGPITPQGTGFLAIQSVSGIMPDFRIAATGV
Pongo_abelii              IPLYYIEDHNGRQRLLKYTPQHMHCGAAFWGPITPQGTGFLAIQSVSGIMPDFRIAATGV
Otolemur_garnetti         IPLYYIEDHNGRQRLLKYTPQHMHCGATFWGPITPQGTGFLAIQSVSGVMPDFRIAATGV
                          345678901234567890123456789012345678901234567890123456789012
                        953      6         7         8         9        1000         1

Brachyteles_arachnoides   ILDLDKSIKIVKKLKLTGFPYKIFKNTSFIKGMFNSALEVAKFEGAMVRTVSGIRGQIKK
Callithrix_jacchus        VLDLDKSIKIVKKLKLTGFPYKIFKNTSFIKGMFNSALEVAKFEGAMIRTVSGIRGQIKK
Aotus_nancymaae           VLDLDKSIKIVKKLKLTGFPYKIFKNTSFIKGMFNSALEMAKFEGAMIRTVSGIRGQIKK
Saimiri_boliviensis       VLDLDKSIKIVKKLKLTGFPYKIFKNTSFIKGMFNSALEVAKFEGAMIRTVSGIRGQIKK
Chlorocebus_sabaeus       VLDLDKSIKIVKKLKLTGFPYKIFKNTSFIKGMFNSALEVAKFEGAVIRTVSGIRGQIKK
Homo_sapiens              VLDLDKSIKIVKKLKLTGFPYKIFKNTSFIKGMFNSALEVAKFEGAVIRTVSGIRGQIKK
Pongo_abelii              VLDLDKSIKIVKKLKLTGFPYKIFKNTSFIKGMFNSALEVAKFEGAVIRTVSGIRGQIKK
Otolemur_garnetti         VLDLDKSIKIVKKLKLTGFPYKIFKNTSFIKGMFNSALEVAKFEGAMIRTVSGIRGQIKK
                          345678901234567890123456789012345678901234567890123456789012
                       1013      2         3         4         5         6         7

Brachyteles_arachnoides   ALRAPEGAFRASFEDKLLMSDIVFMRTWYPVSIPAFYNPVTSLLKPVGEKDTWSGMRTTG
Callithrix_jacchus        ALRAPEGAFRASFEDKLLMSDIVFMRTWYPVSIPAFYNPVTSLLKPVGEKDTWSGMRTTG
Aotus_nancymaae           ALRAPEGAFRASFKDILLMSEIVFMRTWYPVSIPAFYNPVTSLLKPVGEKDTWSGMRTTG
Saimiri_boliviensis       ALRAPEGAFRASFEDKLLMSDIVFMRTWYPVSIPAFYNPVTSLLKPVGEKDTWSGMRTTG
Chlorocebus_sabaeus       ALRAPEGAFRASFEDKLLMSDIVFMRTWYPVSIPAFYNPVTSLLKPVGEKDTWSGMRTTG
Homo_sapiens              ALRAPEGAFRASFEDKLLMSDIVFMRTWYPVSIPAFYNPVTSLLKPVGEKDTWSGMRTTG
Pongo_abelii              ALRAPEGAFRASFEDKLLMSDIVFMRTWYPVSIPAFYNPVTSLLKAVGEKDTWSGMRTTG
Otolemur_garnetti         ALRAPEGAFRATFEDKLLMSDIVFMRTWYPVSIPAFYNPVTSLLKPVGEKDTWSGMQTTG
                          345678901234567890123456789012345678901234567890123456789012
                       1073      8         9       1100        1         2         3

Brachyteles_arachnoides   QLRLAHGIRLKANKDSLYKPILRQKKHFNSLHIPKALQKALPFKNKPKTQAKAGKVPKDR
Callithrix_jacchus        QLRLAHGIRLKANKDSLYKPILRQKKHFNSLHIPKALQKALPFKNKPKTQAKAGKVPKDR
Aotus_nancymaae           QLRLAHGIRLKANKDSLYKPILRQKKHFNSLHIPKALQKALPFKNKPKTQAKAGKVLKDR
Saimiri_boliviensis       QLRLAHGIRLKANKDSLYKPILRQKKHFNSLHIPKALQKALPFKNKPKTQAKAGKVPKDR
Chlorocebus_sabaeus       QLRLAHGVRLKANKDSLYKPILRQKKHFNSLHIPKALQKALPFKNKPKTQAKAGKVPKDR
Homo_sapiens              QLRLAHGVRLKANKDSLYKPILRQKKHFNSLHIPKALQKALPFKNKPKTQAKAGKVPKDR
Pongo_abelii              QLRLAHGVRLKANKDSLYKPILRQKKHFNSLHIPKALQKALPFKNKPKTQAKAGKVPKDR
Otolemur_garnetti         QLRLARGIKLKPNKDSLYKPILRQKKHFNSLHIPKALQKALPFKNKPKNQAKAGKVPKDR
                          345678901234567890123456789012345678901234567890123456789012
                       1133      4         5         6         7         8         9
```

```
Brachyteles_arachnoides    RRPAVVREPHERKILALLDALSTVHSQKMKKAKEQRHLHNKEHFRAKQKEEEEKLKRQKD
Callithrix_jacchus         RRPAVVREPHERKILALLDALSTVHSQKMKKAKEQQHLHNKEHFRARQKEEEEKLKRQKD
Aotus_nancymaae            QRPAVICESHERKILALLDALSTVHSQKMKKAKEQRHLHNKEHFRAKQKEEEEKLKRQKD
Saimiri_boliviensis        RRPAVIREPHERKILALLDALSTVHSQKMKKAKEQRHLHNKEHFRAKQKEEEEKLKRQKD
Chlorocebus_sabaeus        RRPAVIREPHERKILALLDALSTVHSQKMKKAKEQRHLHNKEHFRAKQKEEEEKLKRQKD
Homo_sapiens               RRPAVIREPHERKILALLDALSTVHSQKMKKAKEQRHLHNKEHFRAKQKEEEEKLKRQKD
Pongo_abelii               RRPAVIREPHERKILALLDALSTVHSQKMKKAKEQRHLHNKEHFRAKQKEEEEKLKRQKD
Otolemur_garnetti          QRPAVIREPHERKILALLDALSTVHSQKMKKAKEQRHLHNKEHFKVKQKEEEEKLKRQKD
                           345678901234567890123456789012345678901234567890123456789012
                        1193   1200          1         2         3         4         5

Brachyteles_arachnoides    LRKKLFRIQGQKERRNQKSSLKGAEGQLQ
Callithrix_jacchus         LRKKLFRIQGQKERRNQKSSLKGAEGQLQ
Aotus_nancymaae            LRKKLFRIQGQKERRNQKSSLKGAEGQLQ
Saimiri_boliviensis        LRKKLFRIQGQKERRNQKSSLKGAEGQLQ
Chlorocebus_sabaeus        LRKKLFRIQGQKERRNQKSSLKGAEGQLQ
Homo_sapiens               LRKKLFRIQGQKERRNQKSSLKGAEGQLQ
Pongo_abelii               LRKKLFRIQGQKERRNQKSSLKGAEGQLQ
Otolemur_garnetti          LRKKLFRIQGQKERRNQKSSLKGAE----
                           3456789012345678901234567----
                        1253       6         7
```

CEACAM1

```
                          |------ Signal peptide ----------|------ Ig-like V-type ----
                               *                        *
Brachyteles_arachnoides   MGHLSVQLRRVCVPWQGLLLTASLLTFWNPQTSAQLTIESVPSNAAEGKEVLLLTYNLPE
Callithrix_jacchus        MGHLSARLHRVCVPWQGLLLTASLLTFWNLPTSAQLTTESMPSNAAEGKEVLLLTYNVPQ
Aotus_nancymaae           MGHLSARLHRVCVPWQGLLLTASLLTFWNPPTSAQLTIESMPSNVAEGKEVLLLTHNLPQ
Cebus_capucinus_imitator  MGHLSARLHRVCVPWQGLLLTASLLTFWNPPTSAQLTIESVPSNAAEGKDVLLLTHNLPQ
Saimiri_boliviensis       MGHLSAQLHRVCVPWQGLLLTASLLTFWNPPTSAQLTIESVPSNAAEGKEVLLLARNLPQ
Macaca_nemestrina         MGHLSTPLHRVRVPWQGLLLTASLLTFWSPPTTAQLTIESRPFNVAEGKEVLLLAHNLSQ
Chlorocebus_sabaeus       MGHLSTPLHRVRVPWQGLLLTASLLTFWNPPTTAQLTIESRPFNVAEGKEVLLLAHNLPQ
Homo_sapiens              MGHLSAPLHRVRVPWQGLLLTASLLTFWNPPTTAQLTTESMPFNVAEGKEVLLLVHNLPQ
Pongo_abelii              MGHLSAPLHRVRVPWQGLLLTASLLTFWNPPTTAQLTTESTPFNVAEGKEVLLLVHNLPQ
                          123456789012345678901234567890123456789012345678901234567890
                                   1         2         3         4         5         6

                          ------------------------------------------------------------
                                         *        *
Brachyteles_arachnoides   NTTGFNWYKGERVDGTVRIMGYVIAEQRITPGPAYSSRETIYPNASLLIQNVTLNDTGFY
Callithrix_jacchus        NTNGFNWYKGERVDSTVRIIGYVIASQLTTPGPAYSGREVIYSNASLLIRNVTLNDTGFY
Aotus_nancymaae           NTTGFNWYKGESVDSTRRIMGYVTTTQQTIPGPAHSGRETIYPNASLLIQNVTLNDTGFY
Cebus_capucinus_imitator  NINGFNWYKGQSVDGTRRIIGYVIATQLTTPGPAYSSQETIYPNASLLIQNVTLNDTGFY
Saimiri_boliviensis       DTTGFNWHKGGSVDSTRQITGYVIATQLTTRGPAYSGRETVYPNASLLIQNVTLNDTGFY
Macaca_nemestrina         NLFGFNWYKGERVDAKRLIVAYVIGTQQTTPGPAYSGREIVYSNASLLIQNVTQNDTGSY
Chlorocebus_sabaeus       NTLGYNWHKGERVDAKRLIVAYVIGTQQTTPGPAHSGRERIYSNASLLIQNVTQNDTGSY
Homo_sapiens              QLFGYSWYKGERVDGNRQIVGYAIGTQQATPGPANSGRETIYPNASLLIQNVTQNDTGFY
Pongo_abelii              NPLGYNWYKGEMVDANHRIIGYVISDQLTTPGPAYSSREKIYPNASLLIQNVTQNDTGFY
                          123456789012345678901234567890123456789012345678901234567890
                         61        7         8         9        100         1         2

                          ----------------------|-------- Ig-like C2-type 1 ----------
                                       *
Brachyteles_arachnoides   TLQVIKTDLVNEEVTGQFRVYPELSKPYINSNNSNPVEDEDAVALTCETEAQNTTYLWWV
Callithrix_jacchus        TLQVIKADLVNEEATVQFHVYPELSKPYINSSNSNPVEDEDVVALTCETEAQNTNYLWWV
Aotus_nancymaae           RLQVIMADLVNEEATGQFRVYPELSKPYITSNNYNPVEDEDVVALTCEPETQNTTYLWWV
Cebus_capucinus_imitator  TLQVIKVDLVNEEATGQFRVYPELSKPYINSNNSNPVEDEDAVVLTCETEAQNTTYLWWV
Saimiri_boliviensis       TLQVITANLVNTEATGQFRVYLELSKPYINSSNSNPVEDEDVVALTCETEAQNTTNLWWV
Macaca_nemestrina         TLQVIKGDLVNEEATGQFRVYPELPKPNITINNSNPVEDKDAVTFTCESEAQDTTYLWWV
Chlorocebus_sabaeus       TLQVIKQDLVNEEATGQFRVYPELPKPNITINNSNPVEDKDAVTFTCESEAQDTTYLWWV
Homo_sapiens              TLQVIKSDLVNEEATGQFHVYPELPKPSISSNNSNPVEDKDAVAFTCEPETQDTTYLWWI
Pongo_abelii              TLQVIKSDLVNEEATGQFHVYPETPKPFISSNNSNPVEDNDAVALTCEPGTQDTTYLWWI
                          123456789012345678901234567890123456789012345678901234567890
                        121         3         4         5         6         7         8

                          ---------------------------------------------------|   |----
                               *                                          *
Brachyteles_arachnoides   NNQRLLVSPRLLLSSDNRTLTLLSVTRNDTGPYECEIQNPVSANRSDAVTLNVTYGPDTP
Callithrix_jacchus        NNQSLPVSSRLLLSNDNRTLTLLNVTRNDTGPYECEIQNPVSVNRSDPVTLNVTYGPDTP
Aotus_nancymaae           NNQNLPVSPRLLLSSDNRTLTLLNVTRNDTGPYECEIQNPVSANRSDPVTLNVTYGPDTP
Cebus_capucinus_imitator  NNQSVPVSPRLLLSSDNRTLTLLNVTRNDTGPYECEIQNPVSVNRSDPVTLNVTYGPDTP
Saimiri_boliviensis       NNQSLPVSPRLVLSNDNRTLTLLNITRNDTGPYECEIQNPVSANRSDPVTLNITYGPDTP
Macaca_nemestrina         NNQSLPVSSRLQLSNGNKTLTLLSVLRNDTGPYECEIQNPVSANRSDPVTLNVTYGPDTP
Chlorocebus_sabaeus       NGQSLPVSSRLQLSNGNKTLTLLSVLRNDTGPYECEIQNPVSANRSDPVTLNVTYGPDAP
Homo_sapiens              NNQSLPVSPRLQLSNGNRTLTLLSVTRNDTGPYECEIQNPVSANRSDPVTLNVTYGPDTP
Pongo_abelii              NNQSLLISPRLQLSNGNRTLTLLSVTRNDAGPYECEIQNPVSANRSDPVTLNVTYGPDTP
                          123456789012345678901234567890123456789012345678901234567890
                        181         9        200         1         2         3         4

                          ------------------- Ig-like C2-type 2 ----------------------
Brachyteles_arachnoides   TISPSKTSYYPGANLSLFCDAASNPPAEYSWLINGTSRQ-NTQELFISNITVDHSGSYAC
Callithrix_jacchus        TISPSKTSYYPGANLSLFCDAASNPPAEYSWLINGTSLQ-NTQELFISNITVDDSGSYAC
Aotus_nancymaae           TISPSKTSYYPGANLSLFCDAASNPPAEYSWLINGTSHQKNTQELFISNITVDHSGSYAC
Cebus_capucinus_imitator  TISPSKTSYYSGANLSLFCDAASNPPAEYSWLINGTPCQINTQELFISNITVNRSGSYAC
Saimiri_boliviensis       TISPSKTSYYPGANLSLFCDAASNPPAEYSWLINGTSRQ-NTQELFISNITVNHSGSYAC
Macaca_nemestrina         TISPSDTYYRPGANLSLSCSAASNPPAQYSWLINGTFQQ-STQELFIPNITVNNSGSYTC
Chlorocebus_sabaeus       TISPSDTYYHPGANLSLSCSAASNPPAQYSWLINGTFQQ-STQELFIPNITVNNSGSYTC
Homo_sapiens              TISPSDTYYRPGANLSLSCYAASNPPAQYSWLINGTFQQ-STQELFIPNITVNNSGSYTC
Pongo_abelii              TISPSDTYYRPGANLSLSCYAASNPPAQYSWLINGTFQQ-STQELFIPNITVNNSGSYTC
                          123456789012345678901234567890123456789-01234567890123456789
                        241         5         6         7          8         9
```

```
                          ------------------|   |------------Ig-like C2-type 2 -------
Brachyteles_arachnoides   VALNPVTGRNRTTVKTITVSKLNQ-VARPEVQATSTTVTEDKDSVNLTCSTNDTEISIRW
Callithrix_jacchus        VALNPVTGRNRTTVKTIIVSKLNQ-VAQPEVQATSTTVTEDKDSVNLTCSTNDTEISIKW
Aotus_nancymaae           VALNPVTGRNRTTVKTITVSKLNQ-VARPEVQATSTTVTEDKDSVNLTCSTNDTEISIKW
Cebus_capucinus_imitator  VALNPVTGRNRTTVKTITVSKLNQ-VARPEVQATSTTVTEDKDSVSLTCFTNDTEISIKW
Saimiri_boliviensis       VALNPVTGRNRTTVKTITVSKLNQ-VAQPEVQATSTTVTENKDSVNLTCSTNDTEISIKW
Macaca_nemestrina         HANNSVTGRNRTTVKMIIVSELSPVVAQPQIKASKTTVTEDKDSVNLTCSTNDTGISISW
Chlorocebus_sabaeus       HANNSVTGHNRTTVKMIIVSELSPVVAQPQIKASKTTVTEDKDSVNLTCSTNDTGISISW
Homo_sapiens              HANNSVTGCNRTTVKTIIVTELSP-VAKPQIKASKTTVTGDKDSVNLTCSTNDTGISIRW
Pongo_abelii              HANNSVTGRNRTTVKTIIVTELSP-VVKPQIKASKTTVTEDKDSVNLTCSTNDTGISISW
                          012345678901234567890123-45678901234567890123456789012345678
                        300       1         2          3         4         5

                          ------------------------------------------------------|
                               *
Brachyteles_arachnoides   FFNSQHISSSERIKLFQDNRTLRIDPVKREDAGQYYCEVSNPISVNQSDPIRLTVKYT--
Callithrix_jacchus        FFNNQNILSSERIKLFQDNRTLRIDPVKREDAGQYYCEVSNPISVNQSDPIRLTVKYM--
Aotus_nancymaae           FFNSQNILSSERIKLFQDNRTLRIDPVKREDAGEYYCEVSNPISVNQSDPISLTVKYT--
Cebus_capucinus_imitator  FFNNQSILSSERIKLFQDNRTLRIDPVKRQDAGQYYCEVSNPISVNQSDPIMLTVKYM--
Saimiri_boliviensis       FFNSQNILSSERIKLFQDNRTLRIDPVKREDAGQYYCEVSNPISVNQSDPIMLIVKYT--
Macaca_nemestrina         FFKDQSLPSSERMKLSQDNATLSINPVKREDAGNYSCEVFNLISKNRSDPIMLIVNYNNP
Chlorocebus_sabaeus       FFEDQSLPSSERMKLSQDNATLSINPVKREDAGKYSCEVFNLISKNRSDPIVLIVNYNDP
Homo_sapiens              FFKNQSLPSSERMKLSQGNTTLSINPVKREDAGTYWCEVFNPISKNQSDPIMLNVNYN--
Pongo_abelii              FFENQSLPSSERMKLSQGNTTLSINPVKREDAGKYWCEVFNSISKNQSDPIMLTVKYNYP
                          9012345678901234567890123456789012345678901234567890123456--
                         360         7         8         9       400         1

                                  |---- Transmembrane ----|-- Cytoplasmic
                                                           **
Brachyteles_arachnoides   -EISGLSGGAIAGIVIGVLAGVALIAALMVYFLYVRKTGR
Callithrix_jacchus        -ESSGLSGGAIAGIVIGVLAGVALIAGLVVYFQHSRKTGR
Aotus_nancymaae           -ESSGLSGGAIAGIVIGVLAGVALIAALVVYFLHSRKTGR
Cebus_capucinus_imitator  -ESSGLSRGAIAGIVIGVLAGMALIAALVVYFLHSRKTGR
Saimiri_boliviensis       -ESSGLSGGAIAGIVIGVLAGMALIAALVVYFLHSRKTGR
Macaca_nemestrina         AQENSLPAGAIAGIVIGVLALVVLIVAALACFLHFRKAGR
Chlorocebus_sabaeus       AQENSLTAGAIAGIVIGVLALVVLIAAL-ACFLHFRKTGR
Homo_sapiens              -QENGLSPGAIAGIVIGVVALVALIAVALACFLHFGKTGR
Pongo_abelii              SQENGLSAGAIAGIVIGVLALVALIAVALACFLHFRKTGR
                          7890123456789012345678901234-56789012345
                        417  2         3         4          5
```

CLEC17A

```
                                                                                 *
Brachyteles_arachnoides     MEDEEDEEDDYENMAPPYKDLPPKPGTM-EEEEDD-YENSVPPYKDLPPKPGSVAPPRPP
Callithrix_jacchus          VEEEEDEEDDYENMAPPYKDLPPKPGTM-EEEGDD-YENSAPPYKDLPPKPGSVAPPRPP
Aotus_nancymaae             -EEEDEEDDDYENMAPPYKDLPPKPGTM-EEEEDD-YENSAPPYKDLPPKPGSITPPRPP
Cebus_capucinus_imitator    ---EEEEDDDYENSAPPYKDLPPKPGTM-EEEEDDDYENSAPPYKDLPPKPGSIAPPRPP
Saimiri_boliviensis         VEEEEDEEDDYENMVPPYKDLPPKPGTM-EEEEDD-YENSAPPYKDLPPKPGSIAPPRPP
Chlorocebus_sabaeus         --EEEEEDDDYENSAPAYKDLPPKPGTMKEEEDDDDYENSTPPYKDLPPKPGSSAPPRPP
Homo_sapiens                MEEEEEEEDDYENSTPPYKDLPPKPGTM-EEEDDD-YENSTPPYKDLPPKPGSSAPPRPP
Pongo_abelii                ----EEDDDDYENSTPPYKDLPPKPGTM-EEEDDD-YENSTPPYKDLPPKPGSSAPPRPP
Otolemur_garnetti           ---EEDDADDYENTAPPYKDLPPKPGSM-GEDDDDDYENTAPPYKDLPPKPGLIAPPRPP
                            ----123456789012345678901234-567890-123456789012345678901234
                                         1         2          3          4         5

Brachyteles_arachnoides     RAEKKTEKPPL-CKPRKTTGLDLTAVTCTPPQLAVDLEPSPLQPPLAAAPVPWLSQISGG
Callithrix_jacchus          RAEKKTEKPPL-HKPRQTTGLDLATVTCTPPQLAVDLEPSPLQPPLAAAPVPWLSQTSGG
Aotus_nancymaae             RAEKKTEKPPLPCKPRKMTGLDLTTVTCTPPQLAVDLEPSPLQPPMAAAPAPWLSQTSGG
Cebus_capucinus_imitator    RAEKKTEKPPLPCKPRKTTGLDLAAVTCIPPQLAVDLEPSPLQPPLAAAPARWLSQTSGG
Saimiri_boliviensis         RAEKKTEKPPL-CKPRKMTGLDLNAVTCTPPQLAVDLEPSPLQPPLAAAPAPWPSQTSGG
Chlorocebus_sabaeus         RAKKETEKPPLPCKPRNMTGLDLVSVTCPPPQLAVHLEPSPLQPSLAATPAPWLNQMSEG
Homo_sapiens                RAAKETEKPPL-CKPRNMTGLDLAAVTCPPPQLAVNLEPSPLQPSLAATPVPWLNQRSGG
Pongo_abelii                RTEKETEKPPLPCKPQNMTGLDLAAVTCPPPQLAVNLEPSPLQPSLAATPVPWLNQRSGG
Otolemur_garnetti           RAEKKSENPPLPCKPPKKIGLDLTPVPCVPPQLGIDLEPPPFQPSLAATPVPWLSQKSGS
                            56789012345-678901234567890123456789012345678901234567890123
                           55    6          7         8         9       100         1

                                                      *
Brachyteles_arachnoides     PRCCQKR-LIVCLCLLVVTSLILSCFCLIVTLTKYEELVKELRMLSFQQMMWRANVTGMA
Callithrix_jacchus          PGCCQKR-LIVCLCLLVVTSLILSCFGLIVTLTKYEELVKELRLLSFQQMMWRANVTGMA
Aotus_nancymaae             PGCCQKR-LIVCLCLLVVTSLILSCFGLIVTLTKYEELVKELRMLSFQQMMWRANVTGMA
Cebus_capucinus_imitator    PGCCQKR-LIVCLCLLVATSLILSCFGLILTLTKYEELVKELRMLSFQQMMWRANVTGMA
Saimiri_boliviensis         PGCCQKR-LIVCLCLLVATSLILSCFGLIVTLTKYEELVKELRMLSFQQMMWRANVTGMA
Chlorocebus_sabaeus         PGCCQKR-WMMYLCLMVVTSLFLGCLGLTVTVIKYQKLMEELRMLSFQQMTWRANMTGMS
Homo_sapiens                PGCCQKR-WMVYLCLLVVTSLFLGCLGLTVTLIKYQELMEELRMLSFQQMTWRTNMTGMA
Pongo_abelii                PGCCQKR-WMVYLCLLVVTSLFLGCLGLTVTLIKYQELMEELRMLSFQQMTWRTNMTGMA
Otolemur_garnetti           PGCCQEERLMVYLCVLVVVSLLLGCISLTVTLIKYQEVMEELRMLTFQQMVWQANVTGMA
                            4567890-1234567890123456789012345678901234567890123456789012
                          114      2          3         4         5         6         7

                                                *       **              |----------------
Brachyteles_arachnoides     GLAGLKHDIDRVRADTNQSLLELWGLLDRERVTCPEGWLPFESKCYYFSPTTKSWDGARM
Callithrix_jacchus          GLAGLKHDIDRVRADTNQSLVELWSLLDCSRVTCPEGWLPFESKCYYFSPSTKSWDGARM
Aotus_nancymaae             GLAGLKRDIDRVRADTNQSLVELWGLLDCSRVTCPESWLPFESKCYYFSPTTKSWDGARM
Cebus_capucinus_imitator    GLAGLKHDIDRVRADTNQSLVELWGLLDCSRVTCPEGWLPFESKCYYFSPTTKSWDGARM
Saimiri_boliviensis         GLAGLKHDIDRVRADTNQSLVELWGLLDCSRVTCPEGWLPFESKCYYFSPTTKSWDGARM
Chlorocebus_sabaeus         GLAGLKHDIDRVRADTNQSLVELRGLLGCRRVTCPEGWLPFEGKCYYFSPITKSWDEARM
Homo_sapiens                GLAGLKHDIARVRADTNQSLVELWGLLDCRRITCPEGWLPFEGKCYYFSPSTKSWDEARM
Pongo_abelii                GLAGLKHDIDRVRADTNQSLVELRGLLDCRRITCPEGWLPFEGKCYYFSPSTKSWDEARM
Otolemur_garnetti           GLAGLKKDIDRVRAGTNQSLVELRGLLECTRVTCPEGWVPFEGKCYYFSPSTKSWDEARK
                            345678901234567890123456789012345678901234567890123456789012
                          173       8         9       200         1         2         3

                            ------------ C-type lectin ---------------------------------
                                                               *
Brachyteles_arachnoides     FCQENYSHLVIINSFAEHNFVAKAHGSPRVYWLGLSDREQEGNWRWLDGSPVTLSFWEPE
Callithrix_jacchus          FCQENYSHLVIINSFAEHNFVAKAHGSPRVYWLGLNDREQEGDWRWLDGSPVTLSFWEPE
Aotus_nancymaae             FCQENYSHLVIINSFAEHNFVAKAHGSPRVYWLGLNDREQEGDWRWLDGSPVTLSFWEPE
Cebus_capucinus_imitator    FCQENYSHLVIINSFAEHNFVAKAHGSPRVYWLGLNDREQEGNWRWLDGSPVTLSFWEPE
Saimiri_boliviensis         FCQENYSHLVIINSFAEHNFVAKAHGSPRVYWLGLNDREQEGNWRWLDGSPVTLSFWEPE
Chlorocebus_sabaeus         FCQENYSHLVIINSFAEHNFVAKAHGSPRVYWLGLNDRGQEGDWRWLDGSPVTLSFWEPE
Homo_sapiens                FCQENYSHLVIINSFAEHNFVAKAHGSPRVYWLGLNDRAQEGDWRWLDGSPVTLSFWEPE
Pongo_abelii                FCQENYSHLVIINSFAEHNFVAKAHGSPRVYWLGLNDRAQEGDWRWLDGSPVTLSFWEPE
Otolemur_garnetti           FCQENYSHLVIISNFAEQNFVAKAHRSPRVYWLGLHDKHKEGDWRWLDGSPVTLSFWDPE
                            345678901234567890123456789012345678901234567890123456789012
                          233       4         5         6         7         8         9
```

```
                            -----------------------------------|
Brachyteles_arachnoides     EPNNIQEEDCASMNKGGTWNDLSCHKSTYWICERKCSC
Callithrix_jacchus          EPNNIQEEDCASMNKGGTWNDLSCHKSTYWICERKCSC
Aotus_nancymaae             EPNNIQEEDCASMNKDGTWNDLSCHKSTYWICERKCSC
Cebus_capucinus_imitator    EPNNIQEEDCASMNKDGTWNDLSCHKSTYWICERKCSC
Saimiri_boliviensis         EPNNIQEEDCASMNKDGTWNDLSCYKTTYWICERKCSC
Chlorocebus_sabaeus         EPNNIYNEDCAAMNKDGTWNDLSCYKTTYWICERKCSC
Homo_sapiens                EPNNIHDEDCATMNKGGTWNDLSCYKTTYWICERKCSC
Pongo_abelii                EPNNIHDEDCATMNKGGTWNDLSCYKTTYWICERKCSC
Otolemur_garnetti           EPNNMNEEDCASMNKGGSWNDLSCDKTTYWICERKCSC
                            34567890123456789012345678901234567890
                          293      0         1         2         3
```

DDX52

```
                                                                        *      *
Brachyteles_arachnoides   MDVHDLFRRLGAGAKFDVRRFSADAARFQIGKRKYDFDSSEVLQGLEFFGN-KNVPGV-C
Callithrix_jacchus        MDAHDLFRRLCAGAKFDVRRFSADAARFQIGKRKYDFNSLEMLQELDFFGN-KSVPGV-C
Aotus_nancymaae           MDAHDLFRRLGAGAKFDVRRFSADAARFQIGKRKYDLDSSEVLQGLDFFGNKKCVPGV-C
Cebus_capucinus_imitator  MDAHDLFRRLGAGAKFDVRRFSADAARFQIGKRKYDFDSSEVLQGLDFFGNKKSVPGV-C
Saimiri_boliviensis       MDAHDLFRRLGAGAKFDVRRFSADAARFQIGKRKYDFDSSEVLQGLDFFGN-KSVPGV-C
Macaca_nemestrina         MDVHDLFRRLGAGAKFDTRRFSADAARFQIGKRKYDFDSSEVLQGLDFFGNKKSVPGV-C
Chlorocebus_sabaeus       MDVHDLFRRLGAGAKFDTRRFSADAARFQIGKRKYDFDSSEVLQGLDFFGNKKSVPGV-C
Homo_sapiens              MDVHDLFRRLGAGAKFDTRRFSADAARFQIGKRKYDFDSSEVLQGLDFFGN-KSVPGV-C
Pongo_abelii              MDVHDLFRRLGAGAKFDTRRFSADAARFQIGKRKYDFDSSEVLQGLDFFGNKKSVSGVYS
Otolemur_garnetti         MDIQDLFRRLGTGAKFDVRRFSADAARFQKGKRKYDFDSSEVLQGLDVTGNKKSFSGE-C
                          12345678901234567890123456789012345678901234567890-234567-8
                                   1         2         3         4         5

                                      *
Brachyteles_arachnoides   GASQAHQKPQNEDVKKESLTERKREQSKKKR--MTSEITSQDEGATIQWISSVEAKIEDK
Callithrix_jacchus        GTSQAHQKPQNEEVKKESLTERKREQSKKKR--MTSEITSQDEVATIQWMSSVEAKIEDK
Aotus_nancymaae           GASQAHQKPQSEEVKKESLTERKREQSKKKRKMMTSESTSQDEVATIQWISSVEAKIEDK
Cebus_capucinus_imitator  GASQAHQKPQNEEVKKESLTERKREQSKKKRKTMTSEITSQDEVATIQWISSVEAKIEDK
Saimiri_boliviensis       RASQAHQKPQNEEVKKESLTERKREQSKKKK--MTSEITSQDEVATIQWISSVEAKIEDK
Macaca_nemestrina         GASQTHQKPQNEEKKEESLTERKKEQSKKKRKMMTSEITSQEEGATIQWISSVEAKIEDK
Chlorocebus_sabaeus       GASQTHQKPQNEEKKEESLTERKREQSKRKRKMMTSEITSQEEGASIQWISSVEAKIEDK
Homo_sapiens              GASQTHQKPQNGEKKEESLTERKREQSKKKR--MTSEIASQEEGATIQWMSSVEAKIEDK
Pongo_abelii              GASQTHQKPQNGEKKEESLTERKREQSKKKRKTMTSEITSQEEGATIQWMSSVEAKIEDK
Otolemur_garnetti         GVSQTHQELQNEEKKEESLTERKREQKRQ-REKVTSEISSEGEGTTIQ*MSSVDAKIEEK
                          901234567890123456789012345678901234567-8--90123456789012345678901234
                          60         7         8               9        100       1

                                                                      |-- Q motif ----
Brachyteles_arachnoides   KVQRESKLTSGKLEHLRKEKINFLRNKHKIHVQGTDLPDPIATFQQLDQEYKINSRLLQN
Callithrix_jacchus        KVQRENKLTSGKLERLRKEKINFLRNKHKIHVQGTDLPDPIATFQQLDQEYKINSRLLQN
Aotus_nancymaae           KVQRENKLTSGKLEHLRKEKINFLRNKHKIHVQGTDLPDPIATFQQLDQEYKINSRLLQN
Cebus_capucinus_imitator  KVQRENKLTSGKLEHLRKEKINFLRNKHKIHVQGTDLPDPIATFQQLDQEYKINSRLLQN
Saimiri_boliviensis       KVQRENKLTSGKLEHLRKEKINFLRNKHKIHVQGTDLPDPIATFQQLDQEYKINSRLLQN
Macaca_nemestrina         KVPRESKLTSGKLEHLRKEKINFLRNKHKIHVQGTDLPDPIATFQQLDQEYKINSRLLQN
Chlorocebus_sabaeus       KVPRESKLTSGKLEHLRKEKINFLRNKHKIHVQGTDLPDPIATFQQLDQEYKINSRLLQN
Homo_sapiens              KVQRESKLTSGKLENLRKEKINFLRNKHKIHVQGTDLPDPIATFQQLDQEYKINSRLLQN
Pongo_abelii              KVERESKLTSGKLENLRKEKINFLRNKHKIHVQGTDLPDPIATFQQLDQEYKINSRLLQN
Otolemur_garnetti         KMEGENTLTSEKLEHLRKEKINFFRNKHKIHIQGTDLPDPITKFQQLD*EYKINSQLLQN
                          567890123456789012345678901234567890123456789012-34567890123
                        115    2         3         4         5         6          7

                          --------|      |----- Helicase ATP-binding -----------------
Brachyteles_arachnoides   ILDAGFQMPTPIQMQAIPVMLHGRELLASAPTGSGKTLAFSIPILMQLKQPANKGFRALI
Callithrix_jacchus        ILDAGFQMPTPIQMQAIPVMLHGRELLASAPTGSGKTLAFGIPILMQLKQPANKGFRALI
Aotus_nancymaae           ILDAGFQMPTPIQMQAIPVMLHGRELLASAPTGSGKTLAFSIPILMQLKQPANKGFRALI
Cebus_capucinus_imitator  ILDAGFQMPTPIQMQAIPVMLHGRELLASAPTGSGKTLAFSIPILMQLKQPANKGFRALI
Saimiri_boliviensis       ILDAGFQMPTPIQMQAIPVMLHSRELLASAPTGSGKTLAFSIPILMQLKQPANKGFRALI
Macaca_nemestrina         ILDAGFQMPTPIQMQAIPVMLHGRELLASAPTGSGKTLAFSIPILMQLKQPANKGFRALI
Chlorocebus_sabaeus       ILDAGFQMPTPIQMQAIPVMLHGRELLASAPTGSGKTLAFSIPILMQLKQPANKGFRALI
Homo_sapiens              ILDAGFQMPTPIQMQAIPVMLHGRELLASAPTGSGKTLAFSIPILMQLKQPANKGFRALI
Pongo_abelii              ILDAGFQIPTPIQMQAIPVMLHGRELLASAPTGSGKTLAFSIPILMQLKQPANKGFRALI
Otolemur_garnetti         IVDSGFQTPPPIQM-AIPVMLHGRELLASTPTGSGKTLAFSIPILMQLKQPANKGFRALI
                          45678901234567-890123456789012345678901234567890123456789012
                        174     8          9        200         1         2         3

                          -------------------    Helicase ATP-binding   --------------
Brachyteles_arachnoides   ISPTRELASQIHRELIKISEGTGFRIHMIHKAAVAAKKFGPKSSKKFDILVTTPNRLIYL
Callithrix_jacchus        ISPTRELASQIHRELVKISEGTGFRIHMIHKAVVAAKKFGPKSSKKFDILVTTPNRLIYL
Aotus_nancymaae           ISPTRELASQIHRELIKISEGTGFKIHMIHKAAVAAKKFGPKSSKKFDILVTTPNRLIYL
Cebus_capucinus_imitator  ISPTRELASQIHRELIKISDGTGFRIHMIHKAAVAAKKFGPKSSKKFDILVTTPNRLIYL
Saimiri_boliviensis       ISPTRELASQIHRELIKISEGTGFRIHMIHKAAVAAKKFGPKSSKKFDILVTTPNRLIYL
Macaca_nemestrina         ISPTRELASQIHRELIKISEGTGFRIHMIHKAAVAAKKFGPKSSKKFDILVTTPNRLVYL
Chlorocebus_sabaeus       ISPTRELASQIHRELIKISEGTGFRIHMIHKAAVAAKKFGPKSSKKFDILVTTPNRLIYL
Homo_sapiens              ISPTRELASQIHRELIKISEGTGFRIHMIHKAAVAAKKFGPKSSKKFDILVTTPNRLIYL
Pongo_abelii              ISPTRELASQIHRELIKISEGTGFRIHMIHKAAVAAKKFGPKSSKKFDILVTTPNRLIYL
Otolemur_garnetti         RSPT*ELASQFHRELIKISEGAGFMIHMIYKVAVAAKKSGPKSSKKFDILVTTPNQLIYL
                          3456-7890123456789012345678901234567890123456789012345678901
                        233         4         5         6         7         8         9
```

```
                                             |DEAD| box
                               ---------------------------- Helicase ATP-binding ---------
Brachyteles_arachnoides        LKQDPPGIDLTSVEWLVVDESDKLFEDGKTGFRDQLASIFLACTSHKVRRAMFSATFAYD
Callithrix_jacchus             LKQDPPGIDLTSVEWLVVDESDKLFEDGKTGFRDQLASIFLACTSHKVRRAMFSATFAYD
Aotus_nancymaae                LKQDPPGIDLTSVEWLVVDESDKLFEDGKTGFRDQLASIFLACTSHKVRRAMFSATFAYD
Cebus_capucinus_imitator       LKQDPPGIDLTSVEWLVVDESDKLFEDGKTGFRDQLASIFLACTSHKVRRAMFSATFAYD
Saimiri_boliviensis            LKQDPPGIDLTSVEWLVVDESDKLFEDGKTGFRDQLASIFLACTSHKVRRAMFSATFAYD
Macaca_nemestrina               LKQDPPGIDLTSVEWLVVDESDKLFEDGKTGFRDQLASIFLACTSHKVRRAMFSATFAYD
Chlorocebus_sabaeus            LKQDPPGIDLTSVEWLVVDESDKLFEDGKTGFRDQLASIFLACTSHKVRRAMFSATFAYD
Homo_sapiens                   LKQDPPGIDLASVEWLVVDESDKLFEDGKTGFRDQLASIFLACTSHKVRRAMFSATFAYD
Pongo_abelii                   LKQDPPGIDLTSVEWLVVDESDKLFEDGKTGFRDQLASIFLACTSHKVRRAMFSATFAYD
Otolemur_garnetti              LKQDPPGIDLMSVEWLVVDESDKLFADGKTGFRDQLASVFLACTSPKVRRAMFSATFAYD
                               234567890123456789012345678901234567890123456789012345678901
                             292     300        1         2         3         4         5

                               ---------------|        |----- Helicase C-terminal ---------
Brachyteles_arachnoides        VEQWCKLNLDNVISVSIGARNSAVETVEQELLFVGSETGKLLAMRELVKKGFNPPVLVFV
Callithrix_jacchus             VEQWCKLNLDNVISVSIGARNSAVETVEQELLFVGSETGKLLAMRELVKKGFNPPVLVFV
Aotus_nancymaae                VEQWCKLNLDNVISVSIGARNSAVETVEQELLFVGSETGKLLAMRELVKKGFNPPVLVFV
Cebus_capucinus_imitator       VEQWCKLNLDNVVSVSIGARNSAVETVEQELLFVGSETGKLLAMRELVKKGFNPPVLVFV
Saimiri_boliviensis            VEQWCRLNLDNVISVSIGARNSAVETVEQELLFVGSETGKLLAMRELVKKGFSPPVLVFV
Macaca_nemestrina               VEQWCKLNLDNVISVSIGARNSAVETVEQELLFVGSETGKLLAMRELVKKGFNPPVLVFV
Chlorocebus_sabaeus            VEQWCKLNLDNVISVSIGARNSAVETVEQELLFVGSETGKLLAMRELVKKGFNPPVLVFV
Homo_sapiens                   VEQWCKLNLDNVISVSIGARNSAVETVEQELLFVGSETGKLLAMRELVKKGFNPPVLVFV
Pongo_abelii                   VEQWCKLNLDNVISVSIGARNSAVETVEQELLFVGSETGKLLAMRELVKKGFNPPVLVFV
Otolemur_garnetti              VEQWCKLNLDNVITVSIGARNSAVETVEQELLFVGSETGKLLAVRELVKKGFNPPVLVFG
                               234567890123456789012345678901234567890123456789012345678901
                             352      6         7         8         9        400        1

                               --------------- Helicase C-terminal ------------------------
Brachyteles_arachnoides        QSIERAKELFHELIYEGINVDVIHAERTQQQRDNTVHSFRAGKIWVLICTALLARGIDFK
Callithrix_jacchus             QSIERAKELFHELIYEGINVDVIHAERTQQQRDNTVHSFRAGKIWVLICTALLARGIDFK
Aotus_nancymaae                QSIERAKELFHELIYEGINVDVIHAERTQQQRDNTVHSFRAGKIWVLICTALLARGIDFK
Cebus_capucinus_imitator       QSIERAKELFHELIYEGINVDVIHAERTQQQRDNTVYSFRAGKIWVLICTALLARGIDFK
Saimiri_boliviensis            QSIERAKELFHELIYEGINVDVIHAERTQQQRDNTVYSFRAGKIWVLICTALLARGIDFK
Macaca_nemestrina               QSIERAKELFHELIYEGINVDVIHAERTQQQRDNTVHSFRAGRIWVLICTALLARGIDFK
Chlorocebus_sabaeus            QSIERAKELFHELIYEGINVDVIHAERTQQQRDNTVHSFRAGRIWVLICTALLARGIDFK
Homo_sapiens                   QSIERAKELFHELIYEGINVDVIHAERTQQQRDNTVHSFRAGKIWVLICTALLARGIDFK
Pongo_abelii                   QSIERAKELFHELIYEGINVDVIHAERTQQQRDNTVHSFRAGKIWVLICTALLARGIDFK
Otolemur_garnetti              QSMERAKGLFHELIYEGINVDVIHAERTQQQRDNTVHSFRAGKIWVLICTALLARGIDFK
                               234567890123456789012345678901234567890123456789012345678901
                             412      2         3         4         5         6         7

                               --------------- Helicase C-terminal ------------------------
Brachyteles_arachnoides        GVNLVINYDFPTSSVEYIHRIGRTGRAGNKGKAITFFTEDDKPLLRSVANVIQQAGCPVP
Callithrix_jacchus             GVNLVINYDFPTSSVEYIHRIGRTGRAGNKGKAITFFTEDDKPLLRSVANVIQQAGCPVP
Aotus_nancymaae                GVNLVINYDFPTSSVEYIHRIGRTGRAGNKGKAITFFTEDDKPLLRSVANVIQQAGCPVP
Cebus_capucinus_imitator       GVNLVINYDFPTSSVEYIHRIGRTGRAGNKGKAITFFTEDDKPLLRSVANVIQQAGCPVP
Saimiri_boliviensis            GVNLVINYDFPTSSVEYIHRIGRTGRAGNKGKAITFFTEDDKPLLRSVANVIQQAGCPVP
Macaca_nemestrina               GVNLVINYDFPTSSVEYIHRIGRTGRAGNKGKAITFFTEDDKPLLRSVANVIQQAGCPVP
Chlorocebus_sabaeus            GVNLVINYDFPTSSVEYIHRIGRTGRAGNKGKAITFFTEDDKPLLRSVANVIQQAGCPVP
Homo_sapiens                   GVNLVINYDFPTSSVEYIHRIGRTGRAGNKGKAITFFTEDDKPLLRSVANVIQQAGCPVP
Pongo_abelii                   GVNLVINYDFPTSSVEYIHRIGRTGRAGNKGKAITFFTEDDKPLLRSVANVIQQAGCPVP
Otolemur_garnetti              DVNLVINYDFPTSSVEYIYRTGRTGRAGHKGKAITFSTEDDKPLLRSIASV-QQAGCPAP
                               234567890123456789012345678901234567890123456789012345678901
                             472      8         9        500        1         2         3

                               -------|
                                      |----------------- Lys-rich ------------------|
Brachyteles_arachnoides        EYIKGFQKLLSKQKKKMIKKPLERESISTTPKCFLEKAKDKQKKVTGQNSKKKVALEDKN
Callithrix_jacchus             EYIKGFQKLLSKQKKKMIKKPLERESISTTPKCFLEKAKDKRKKVTGQNSKKKVALEDKN
Aotus_nancymaae                EYIKGFQKLLSKQKKKMIKKPLERESISTTPKCFLEKAKDKQKKVTGQNSKKKVALEDKK
Cebus_capucinus_imitator       EYIKGFQKLLSKQKKKMVKKPLERESISTTPKYFLEKAKDKRKKVTRQNSKKKVALEDKK
Saimiri_boliviensis            EYIKGFQKLLSKQKKKMIKKPLERESISTTPKCFLEKAKDERKKVTGQNSKKKVAFEDKN
Macaca_nemestrina               EYIKGFQKLLSKQKKKMIKKPLERESISTTPKCFLEKAKDKRKKVTGQNSKKKVALEDKS
Chlorocebus_sabaeus            EYIKGFQKLLSKQKKKMIKKPLERESISTTPKCFLEKAKDKRKKVTGQNSKKKVALEDKS
Homo_sapiens                   EYIKGFQKLLSKQKKKMIKKPLERESISTTPKCFLEKAKDKQKKVTGQNSKKKVALEDKS
Pongo_abelii                   EYIKGFQKLLSKQKKKMIKKPLERESISTTPKCFLEKAKDKQKKVTGHNSKKKVALEDKS
Otolemur_garnetti              EYIKGFQKLLSK-KKKMIKKPVERESISATPKYFLEKAKDK-KKVSSQNTKKKVALEE--
                               234567890123-4567890123456789012345678901-2345678901234567--
                             532      4          5         6         7          8
```

DNAJC4

```
                                                    |---------------------------
                                   *                          *
Brachyteles_arachnoides   MPPLLPLRLCRLWFRSPPSRLFGTAAGQRSRPRNYYDVLGVHPGASSEEVKRAFFAKSKE
Callithrix_jacchus        MPPLLPLRLCRLWPRSPPSRLLGTAAGQRSRPRNYYDVLGVHPGASTEEVKRAFFAKSKE
Aotus_nancymaae           MPPLLPLRLCRLWPRSPPSRLHGTAAGQRSSPRNYYDVLGVHPGASTEEVKRAFFAKSKE
Cebus_capucinus_imitator  MPPLLPLRLCRLWPHSPPSRLLGTAAGQRSRPRNYYDVLGVHPGASTEEVKRAFFAKSKE
Saimiri_boliviensis       MLPLLPLRLCRLWPRSPPSRLLETAAGQRSRPRNYYDVLGVHPGASTEEVKRAFFAKSKE
Macaca_nemestrina         MPPLLPLRLCRLWPRSPPSRLLGAAAGQRSSPSNYYELLGVHPGASTEEVKRAFFSKSKE
Papio_anubis              MPPLLPLRLCRLWPRSPPSRLLGAAAGQRSSPSNYYELLGVHPGASTEEVKRAFFSKSKE
Chlorocebus_sabaeus       MPPLLLLRLCRLWPRSPPSRLLGAAAGQRSGPSNYYELLGVHPGASTEEVKRAFFSKSKE
Homo_sapiens              MPPLLPLRLCRLWPRNPPSRLLGAAAGQRSRPSTYYELLGVHPGASTEEVKRAFFSKSKE
Pongo_abelii              MPPLLPLRLCRLWPRSPPSRLLGAAAGQRSRPSTYYELLGVHPGASTEEVKRAFFSKSKE
Otolemur_garnetti         -----PLRLCRLWPRSPPTRPLGAAAGQRSSPSNYYELLGVHPSASTEEVKRAFFTKSKE
                               1234567890123456789012345678901234567890123456789012345
                                        1         2         3         4         5

                          --------- DnaJ domain -----------------|
                                                                              *
Brachyteles_arachnoides   LHPDRDPGNPALHNRFVELSEAYHVLSREQSRRSYDDQLRSPSPPKSPGTTACDKSARQT
Callithrix_jacchus        LHPDRDPGNPALHNRFVELNEAYHVLSHEQSRRSYDDQLRSPGPPKSPGTTAHDKSARQT
Aotus_nancymaae           LHPDRDPGNPALHNRFVELSEAYHVLSREQSRRSYDDQLRSPSPPKSPGTTARDKSAHQT
Cebus_capucinus_imitator  LHPDRDPGNPALHNRFVELSEAYHVLSHEQSRRSYDDQLRSPSPPESPGTTAHDKSTCQT
Saimiri_boliviensis       LHPDRDPGNPALHNRFVELNEAYHVLSHEQSRCRYDDQLRSPRPPKSPGTTARDKSTRQT
Macaca_nemestrina         LHPDRDPGNPSLHSRFVELSEAYRVLSREQSRRRYDAQLRSGGPPKSPQTTAHDKSAGQT
Papio_anubis              LHPDRDPGNPSLHSRFVELSEAYRVLSREQSRRRYDAQLRSGGPPKSPQTTAHDKSAGQT
Chlorocebus_sabaeus       LHPDRDPGNPSLHSRFVELSEAYRVLSREQSRRRYDAQLRSGGPPKSPQTTAHDKSAGQT
Homo_sapiens              LHPDRDPGNPSLHSRFVELSEAYRVLSREQSRRSYDDQLRSGSPPKSPRTTVHDKSAHQT
Pongo_abelii              LHPDRDPGNPSLHSRFVELSEAYRVLSREQSRRSYDDQLRSGSPPKSPRTTAHDKSACQT
Otolemur_garnetti         LHPDRDPGNPTLHSRFVELSEAYRVLSREQSRRSYDHQLRSASPPKSPGNTAHPKSARQT
                          678901234567890123456789012345678901234567890123456789012345
                         56    6         7         8         9        100         1

                                                              |-- Transmembrane ---|
                                 *
Brachyteles_arachnoides   HSSWEHPNAQYWSQFHHARPQGPQSRQQQHKCNQQVLRYCLLLMLAGMGLHYVVFRMVEQ
Callithrix_jacchus        HSSWEHPNAQYWSQFHHVRPQGPQSRQRQHKCNQQVLRYCLLLMLVGMGLHYVVFRMVEQ
Aotus_nancymaae           HSSWEHPNAQYWSQFHHVRPQGPQSRQQQHKCNQQVLRYCLLLMLAGMGLHYVIFRMVEQ
Cebus_capucinus_imitator  HSSREHPNAQYWSQFHHVRPQGPQSRQQQHKCNQQVLRYCLLLMLVGMGLHYVIFRMVEQ
Saimiri_boliviensis       HSSWEHPNAQYWSQFHHVRPQGPQARQQQHKYNQQVLRYCLLLMLAGMGLHYVVFRMVER
Macaca_nemestrina         HSSWAHPNAQYWSQFHSVRPQGPQSRQQQHKQNKQVLRYCLLLMLAGMGLHYIAFRKVKQ
Papio_anubis              HSSWASPNAQYWSQFHRVRPQGPQARQQQHKQNKQVLGYCLLLMLAGMGLHYIAFRKVKQ
Chlorocebus_sabaeus       HSSWAPPNTQYWSQFHSVRPQGPQSRQQQHKQNKQVLGYCLLFMLAGMGLHYIAFRKVKQ
Homo_sapiens              HSSWTPPNAQYWSQFHSVRPQGPQLRQQQHKQNKQVLGYCLLLMLAGMGLHYIAFRKVKQ
Pongo_abelii              HSSWAPPNAQYWSQFHSVRPQGPQLRQQQHKQNKQVLGYCLLLMLAGMGLHYIAFRKVKQ
Otolemur_garnetti         HSSWAPPNAEYWAQFHGMRPQGPKSRQQQYKHNQRVLGYCLLLMLAGMGLHYVAFRKLEQ
                          678901234567890123456789012345678901234567890123456789012345
                       116    2         3         4         5         6         7

Brachyteles_arachnoides   MHLNFMDEKDRIITAMYNEARARAR
Callithrix_jacchus        MHLNFMDEKDRIITAMYNEARARAR
Aotus_nancymaae           MHLNFMDEKDRIITAMYNEARARAR
Cebus_capucinus_imitator  MHLNFMDEKDRIITAMYNDARARAR
Saimiri_boliviensis       MHLNFMDEKDRIITAMYNEARARAR
Macaca_nemestrina         MHLNFMDEKDRIITAMYNEARER--
Papio_anubis              MHLNFMDEKDRIITAMYNEARER--
Chlorocebus_sabaeus       MHLNFMDEKDRIITAMYNEARARAQ
Homo_sapiens              MHLNFMDEKDRIITAFYNEARARAR
Pongo_abelii              MHLNFMDEKDRIITAFYNEARARAR
Otolemur_garnetti         MHRSFMDEKDKIITAIYNDRRAQAR
                          6789012345678901234567890
                       175    8         9       200
```

ECM1

```
Brachyteles_arachnoides   MGTIARAALVLAYLTVASAASEGGFETAGQRQLRPE----HFQEVGYAAPPSPPLSRSLP
Callithrix_jacchus        MGTTARAALVLAYLAVASAASEGGFETAGQGQLRPE----HFQEVGYAAPPSPPLSRSLP
Aotus_nancymaae           MGTTARAALVLAYLAVASAVSEGGFETAGQRQLRPEHIMQHFQEVGYAAPLSPPLSQSLP
Cebus_capucinus_imitator  MGTTARAALVLAYLAVASAASEGGFETAEQRQLRPEHIMQHFQEVGYAAPPSPPLSRSLP
Saimiri_boliviensis       MGTTARAALVLAYLAVASAASEGGFETIEQRQVRPE----HFHEVGYAAPPSPPLSRSLP
Macaca_nemestrina         MGTTVRAALVLAYLAIASAASEGGFKATGQRQLRPE----HFQEVGYAAPPSPPLSRSLL
Papio_anubis              MGTTVRAALVLAYLAIASAASEGGFKATGQRQLRPE----HFQEVGYAAPPSPPLSRSLL
Chlorocebus_sabaeus       MGTTVRAALVLAYLAIASAASEGGFKATGQRQLRPE----HFQEVGYAAPPSPPLSRSLL
Homo_sapiens              MGTTARAALVLTYLAVASAASEGGFTATGQRQLRPE----HFQEVGYAAPPSPPLSRSLP
Pongo_abelii              MGTTARAALVLTYLAVASAASEGGFKATGQRQLRPE----HFQEVGYAAPPSPPLSRSLP
                          123456789012345678901234567890123456----78901234567890123456
                                   1         2         3             4         5

Brachyteles_arachnoides   MDHPDTSQHGPPFEEQKEVQPPPCQDTTPVQQEELPPARFPAEKE---SPPLPQEAVPLQ
Callithrix_jacchus        VDHPDTSQHGPPFEEQKESKPPPCQDTTPVQQEELLPSRFPAEKE---GSPLPQEAVPLQ
Aotus_nancymaae           VDHADTSQHGPPFEEQKEVQHPPCQDTTPVQQEELPPARFPAEKEVQDGPPLPQEAVPLQ
Cebus_capucinus_imitator  TDHSDTSQHGPPFEEQKEVQPPPCQDTTPVQQEELLPARFPAEKEVQDSPPLPQEAVPLQ
Saimiri_boliviensis       LDHPDTSQHGPPFEEQKEVQPPPGQDTIPIQQEELLPAGFPAEKE---SPPLPQEAVPLQ
Macaca_nemestrina         MDHPDSSQHGPPFEGQSQVQPPPSQEATPIQQEELLPAQLPAEKEV--GPPLSQEAVPLQ
Papio_anubis              MDHPDTSQHGPPFEGQSQVQPPPSQEATPIQQEELLPAQLPAEKEV--GPPLSQEAVPLQ
Chlorocebus_sabaeus       MDHPDTSQHGPPFEGQSQVQPPPSQEATPIQQEELLPAQLPAEKEV--GPPLSQEAVPLQ
Homo_sapiens              MDHPDSSQHGPPFEGQSQVQPPPSQEATPLQQEKLLPAQLPAEKE---GPPLPQEAVPLQ
Pongo_abelii              MDHPDTSQHGPPFEGQSQVQPPPSQEATPLQQEELLPAQLPAEKEV--GPPLPQEAVPLQ
                          789012345678901234567890123456789012345678901---234567890123
                         57  6         7         8         9       100         1

Brachyteles_arachnoides   KELPSLQHPSEQKEGTPAPFGDQNHPEPESWNAAQHCQQGRSRGGWGHRLDGFPPGRPSP
Callithrix_jacchus        QEPLSLQHPSEQKEGTPAPFGNRNLPEPESWNAAQHCRQGRSRGGWGHRLDGFPPGRPSP
Aotus_nancymaae           KELPSLQHPSEQKEGTPAPFGNQNHPEPESWNAAQHCQQGRSRGGWGHRLDGFPPGRPSP
Cebus_capucinus_imitator  KEAPSLQHPSEQKEGTPAPFGNQNHPEPESWNAAQHCQQGRSRGGWGHRLDGFPPGRPSP
Saimiri_boliviensis       KEPPSLQHPSEQKEGTPAPFGNQNHPEPESWNAAQHCQQGRSRGGWGHRLDGFPPGRPSP
Macaca_nemestrina         KELPSLQQPSEQKEGTPAPFGDQSHSEPESWNAAQHCQQGRSQGGWGHRLDGFPPGRPSP
Papio_anubis              KELPSLQQPSEQKEGMPAPFGDQSHSEPESWNAAQHCQQGRSQGGWGHRLDGFPPGRPSP
Chlorocebus_sabaeus       KELPSLQQPSEQKEGMPAPFGDQSHSEPESWNAAQHCQQGRSQGGWGHRLDGFPPGRPSP
Homo_sapiens              KELPSLQHPNEQKEGTPAPFGDQSHPEPESWNAAQHCQQDRSQGGWGHRLDGFPPGRPSP
Pongo_abelii              RELPSLQHPNEQKEGMPAPSGDQSHPEPESWNAAQHCQQGRSQGGWGHRLDGFPPGRPSP
                          456789012345678901234567890123456789012345678901234567890123
                        114     2         3         4         5         6         7

                                                               |----------------------
                                                                  *
Brachyteles_arachnoides   DNLNQICLPDRQHVVYGPWNLPQSGYSHLSRQGETLNFLEAGYSRCCHCRSHTNRLECAK
Callithrix_jacchus        DNLNQICLPDRQHVVYGPWNLPQSGYSHLSRQGETLNFLETGYSRCCHCRSHTNRLECAK
Aotus_nancymaae           DNLNQICLPDRQHVVYGPWNLPQSGYSHLSRQGETLNFLETGYSRCCHCRSHTNRLECAK
Cebus_capucinus_imitator  DNLNQICLPDRQHVVYGPWNLPQSGYSHLSRQGETLNFLETGYSRCCHCRSHRNRLECAK
Saimiri_boliviensis       DNLNQICLPGRQHVLYGPWNLPQSGYSHLSRQGETLNFLEAGYSRCCHCRSHTNRLECAR
Macaca_nemestrina         ENLNQICLPDRQHVVYGPWNLPQSGYSHLTRQGETLNFLEIGYSRCCHCRSHTNRLECAK
Papio_anubis              ENLNQICLPDRQHVVYGPWNLPQSGYSHLTRQGETLNFLEIGYSRCCHCRSHTNRLECAK
Chlorocebus_sabaeus       ENLNQICLPDRQHVVYGPWNLPQSGYSHLTRQGETLNFLEIGYSRCCHCRSHTNRLECAK
Homo_sapiens              DNLNQICLPNRQHVVYGPWNLPQSSYSHLTRQGETLNFLEIGYSRCCHCRSHTNRLECAK
Pongo_abelii              DNLNQICLPNRQHVVYGPWNLPQSSYSHLTRQGETLNFLEIGYSRCCHCRSHTNRLECAK
                          456789012345678901234567890123456789012345678901234567890123
                        174     8         9       200         1         2         3

                          ---------- Serum albumin-like -------------------|
Brachyteles_arachnoides   LVWEDTMSRFCEAEFSVKTRPHWCCTRQGEARFSCFQEEAPQPHYQLRACPSHQPDISSG
Callithrix_jacchus        LVWEDTMSRFCEAEFSVKTRPHWCCTRQGEARFSCFQEEAPQPHYQLQPCPSHQPDISSG
Aotus_nancymaae           LVWEDTMSRFCEAEFSVKTRPHWCCTRQGEARFSCFQEEAPQPHYQLQACPSHQPDISSG
Cebus_capucinus_imitator  LVWEDTMSRFCEAEFSVKTRPHWCCTRQGEARFSCFQEKAPQPHYQLQACPSQQPDISSG
Saimiri_boliviensis       LVWEDTMSRFCEAEFSVKTRPHWCCTRQGEARFSCFQEEAPQPHYQLQACPSHQPAMSSG
Macaca_nemestrina         LVWEEAMSRFCEAEFSVKTRPHWCCTQQGEARFSCFQEEAPQPHYQLRACPSHQPDISSG
Papio_anubis              LVWEEAMSRFCEAEFSVKTRPHWCCTQEGEARFSCFQEEAPQPHYQLRACPSHQPDISSG
Chlorocebus_sabaeus       LVWEEAMSRFCEAEFSVKTRPHWCCTQQGEARFSCFKEEAPQPHYQLRACPSHQPDISSG
Homo_sapiens              LVWEEAMSRFCEAEFSVKTRPHWCCTRQGEARFSCFQEEAPQPHYQLRACPSHQPDISSG
Pongo_abelii              LVWEEAMSRFCEAEFSVKTRPHWCCTRQGEVRFSCFQEEAPQPHYQLRACPSHQPDISSG
                          456789012345678901234567890123456789012345678901234567890123
                        234     4         5         6         7         8         9
```

```
                                                                     |----------------
Brachyteles_arachnoides   LELPFPPGAPTLDNIKNICHLRRFSSVPRHLPATDPLQRQLLALTRLEREFQRCCRQGSN
Callithrix_jacchus        LELPFPPGAPTLDNIKNICHLRRFSSVPRHLPATDPLRRQLLALTRLEREFQRCCRQGSN
Aotus_nancymaae           LELPFPPGVPTLDNIKNICRLRRFSSVPRHLPATDPLQRQLLALTRLEREFQRCCRQGSN
Cebus_capucinus_imitator  LELPFPPGEPTLDNIKNICHLRRFSSVPRHLPATDPLQRQLLALTRLEREFQRCCRQGSN
Saimiri_boliviensis       LELPFPPGAPTLDNIKNICRLRRFSSVPRHLPATDPLQRQLLALTRLEREFQRCCRQGSN
Macaca_nemestrina         LELPFPPGAPTLDNIKNICHLRRFRSVPRNLPATDPLQRELLALIRLEREFQRCCRQGNN
Papio_anubis              LELPFPPGAPTLDNIKNICHLRRFRSVPRNLPATDPLQRELLALIRLEREFQRCCRQGNN
Chlorocebus_sabaeus       LELPFPPGAPTLDNIKNICHLRRFRSVPRNLPATEPLQRELLALIRLEREFQRCCRQGNN
Homo_sapiens              LELPFPPGVPTLDNIKNICHLRRFRSVPRNLPATDPLQRELLALIQLEREFQRCCRQGNN
Pongo_abelii              LELPFPPGAPTLDNIKNICHLRRFRSVPRNLPATDPLQRELLALIQLEREFQRCCRQGNN
                          456789012345678901234567890123456789012345678901234567890123
                        294   300         1         2         3         4         5

                          ------ Serum albumin-like -------------------------|
Brachyteles_arachnoides   HTCTWKAWEDTLDKYCEREYAVKTHQHSCCHYPPSPTRDECFASRAPYPNYDRDILTIDI
Callithrix_jacchus        HTCTWKAWEDTLDKYCDQEYAIKTHHHSCCHYPPSPTRDECFASRAPYPNYDRDILTIDI
Aotus_nancymaae           HTCTWKAWEDTLDKYCDQEYAVKTHHHSCCHYPPSPTRDECFARRAPYPNYDRDILTIDI
Cebus_capucinus_imitator  HTCTWKAWEDTLDKYCDQEHAIKTHPHSCCHYPPSPTRDECFASRAPYPNYDRDILTIDI
Saimiri_boliviensis       HTCTWKAWEDTLDKYCDQEHAVKTHHHLCCHYPPSPARDDCFASRAPYPNYDRDILTIDI
Macaca_nemestrina         HTCTWKAWEDTLDKYCDREYTVKTHHHSCCRHPPSPIRDECFARRAPYPNYDRDILAIDI
Papio_anubis              HTCTWKAWEDTLDKYCDREYTVKTHHHSCCRHPPSPIRDECFARRAPYPNYDRDILAIDI
Chlorocebus_sabaeus       HTCTWKAWEDTLDKYCDREYAVKTHHHSCCRHPPSPTRDDCFARRAPYPNYDRDILAIDI
Homo_sapiens              HTCTWKAWEDTLDKYCDREYAVKTHHHLCCRHPPSPTRDECFARRAPYPNYDRDILTIDI
Pongo_abelii              HTCTWKAWEDTLDKYCDREYAVKTHHHSCCHHPPSPTRDECFARRAPYPNYDRDILTIDI
                          456789012345678901234567890123456789012345678901234567890123
                        354     6         7         8         9       400         1

Brachyteles_arachnoides   SQVTPSLMGHLCGNHRVLTKHKHIPGLIHNMTARCCDLLFPEQACCAEEEKLTFINDLCG
Callithrix_jacchus        SQVTPNLMGHLCGNHRVLTKHKHIPGLIHNMTARCCDLPFPEQACCAEEEKLTFINDLCG
Aotus_nancymaae           SQVTPNLMGHLCGNHRVLTKHKHIPGLIHNMTARCCDLPFPEQACCAEEEKLTFINDLCG
Cebus_capucinus_imitator  SQVTPNLMGHLCGNHRVLTKHKHIPGLIHNMTARCCDLPFPEQACCAEEEKLAFINDLCG
Saimiri_boliviensis       SQVTPNLMGHLCGNHRVLAKHKHIPGLIHNMTARCCDLPFPEQACCAEEEKLAFINDLCG
Macaca_nemestrina         SRVTPNLMGHLCGNQRVLTKHKHIPGLIHNMTARCCDLPFPEQACCAEEEKLTFINDLCG
Papio_anubis              SRVTPNLMGHLCGNQRVLTKHKHIPGLIHNMTARCCDLPFPEQACCAEEEKLTFINDLCG
Chlorocebus_sabaeus       SRVTPNLMGHLCGNQRVLTKHKHIPGLIHNMTARCCDLPFPEQACCAEEEKLTFINDLCG
Homo_sapiens              GRVTPNLMGHLCGNQRVLTKHKHIPGLIHNMTARCCDLPFPEQACCAEEEKLTFINDLCG
Pongo_abelii              SRVTPNLMGHLCGNQRVLTKHKHIPGLIHNMTAHCCDLPFPEQACCAEEEKLTFINDLCG
                          456789012345678901234567890123456789012345678901234567890123
                        414     2         3         4         5         6         7

Brachyteles_arachnoides   PRRNFWRDPALCCYLSPGDEQVNCFNVNYLRNVALVAGDTGNSKGQGEQGPTGGTNISST
Callithrix_jacchus        PRRNFWRDPALCCYLSPGDEQVTCFNINYLRNVALVAGDTENSKGQGEQGPTGGTNISST
Aotus_nancymaae           PRRNFWRDPALCCYLSPEDEQVNCFNINYLRNVALVAGDTEYSKGQGERGPTGGTNVSST
Cebus_capucinus_imitator  PRRNFWRDPALCCDLSPGDEQVNCFNINYLRNVALVSGDTGNSKGQGEQGPTGGTNISST
Saimiri_boliviensis       PRRNFWRDPALCCYLSPGDEQVNCFNINYFRNVALVAGDTENSRGEGEQGPTGGTNISST
Macaca_nemestrina         PRRKFWQDPALCCYLSPGDEQVNCFNINYLRNVALVAGDTENTKGQGEQGSIGGTNISST
Papio_anubis              PRRKFWQDPALCCYLSPGDEQVNCFNINYLRNVALVAGDTENTKGQGEQGSIGGTNIGST
Chlorocebus_sabaeus       PRRKFWQDPALCCYLSPGDEQVNCFNINYLRNVALVAGDTENTKGQGEQGSIGGTNIGST
Homo_sapiens              PRRNIWRDPALCCYLSPGDEQVNCFNINYLRNVALVSGDTENAKGQGEQGSTGGTNISST
Pongo_abelii              PRRNFWRDPALCCYLSPGDEQVNCFNINYLRNVALVAGDTENAKGQGEQGSTGGKNISST
                          456789012345678901234567890123456789012345678901234567890123
                        474     8         9       500         1         2         3

Brachyteles_arachnoides   SEPKEE
Callithrix_jacchus        SEPKEE
Aotus_nancymaae           SEPKEE
Cebus_capucinus_imitator  SEPKEE
Saimiri_boliviensis       SEPKEE
Macaca_nemestrina         SEPKEE
Papio_anubis              SEPEEE
Chlorocebus_sabaeus       SEPKEE
Homo_sapiens              SEPKEE
Pongo_abelii              SEPKEE
                          456789
                        534
```

EMCN

```
                             |Signal pep|
Brachyteles_arachnoides       LFLLPSLCSSDN-TGVINA-NTSPIVSTTIKTSVRTPSTESSQNPVITPAIGTTPNGTTN
Callithrix_jacchus            MFFHRTLQSSYF-SDVINA-NTSPIVSTTIKTSVLTPSTESLQNPVITPTIGTTPKGTTS
Aotus_nancymaae               LFLLPSLCSSDVNTDVINASTSLPIVSTTIKTSVLTPSTESLWNPVITPTMGTTPKGTTN
Cebus_capucinus_imitator      LFLLPSLCSSDVDTGLINANISSPSVTTTIKTSAQTPSTESLQNPVTTPTIGTIPTGTTN
Saimiri_boliviensis           LFLLPSLCSSDN-TDVINA-NTSPIVSTAIKTSALPSSTESLSSPVITPIIGTTPKGTTN
Macaca_nemestrina             LFLLPSICSSENSTGVINAATPSSVVVT-TETSVTTPNTGSSQKNVITTTNETTPKGTIT
Chlorocebus_sabaeus           LFLLPSICSSENRTGVINVTTLSSVVVTTTKTSVATPNTGSSQKNVTTTTTETTPKGTIT
Homo_sapiens                  LFLLPSICSSNS-TGVLEA-ANNSLVVTTTKPSITTPNTESLQKNVVTPTTGTTPKGTIT
Pongo_abelii                  LFLLPSICSSNS-TGVLEA-ANNSLVVTTIKTSITTPNTESLQKNVITPTTGTTPKGKIT
Otolemur_garnetti             ----------------------------------------------------TSPKGTTN
                                                                                  12345678

                                                                                        *
Brachyteles_arachnoides       SELLTKSLLSTVASLTTSKEE-LRITTTDGRKNESIISNVAVTNVALPNAVSTLQSSQQK
Callithrix_jacchus            SELLKKSLLSTIASLTTSKEE-LRITSTDGKMNESIISSVAVTNLALPNAVSTSPNSQHK
Aotus_nancymaae               SEL------STVASLTTSKED-LGITTTDGRQNESIISNAAVTYLALPTAVSTLQSSQHK
Cebus_capucinus_imitator      SELLKKSLF-TVASLTTSTEE-LRITTTDGRENESIISNVAVTNLALPNAVSTSQSSQHK
Saimiri_boliviensis           SELLKNPLLSTVASLTTSKEE-LIITTTDGKKNESIISNVAVTNLALPNAVSTLQSYQHK
Macaca_nemestrina             SELPKTSLTTTVTLLITSKDEGLRVTTPDVRENKSIISNVTVTSVTPPNAVSALQSSKPK
Chlorocebus_sabaeus           SELLKTSLTTTVTLLTTSKDEGLRVTTPDVTENKSIISNVTVTSVTPPNAVSTLQSSKPK
Homo_sapiens                  NELLKMSLMSTATFL-TSKDEGLKATTTDVRKNDSIISNVTVTSVTLPNAVSTLQSSKPK
Pongo_abelii                  NELLKMSLMSAVTL--TSKDEGLKVTTTDVRKNESIVSNVTVTIVTLPNAVSTLQSSKPK
Otolemur_garnetti             SESLKTTLVSTVSSLSTVKGQELRTTTNGTTKNESIFTTVTEANIPLSKVVSTSQSFQNK
                              901-------2345--67890-12345678901234567890123456789012345678
                              10                  2          3         4         5

                                                                       *              *
Brachyteles_arachnoides       TETQSTIKTTEIPGSTLQPDASPSKTGTLHSMPVTIPESTLPSQ--GTENGKNASAVATS
Callithrix_jacchus            SNVKLVLASEYFPASTLQPDASPSKTGTLHSMPVTIPEGTLQSQ--GTENGKNASASATS
Aotus_nancymaae               TETQSTIKTMELSGNTLQPDVSPSKTGTLHSTPVTIPESTLQSQ--GTENGKNASASATS
Cebus_capucinus_imitator      TETQSTIKIMKIPGSTLQPDASPSKTGTSHSMPATIPESTLQSQ--GTENGKNSSASAAS
Saimiri_boliviensis           TETQSTIKIMEISGSTRQPDASSSKTGTLHSMPVTIPESTLQSQ--GTENGKNASASAAS
Macaca_nemestrina             TETQSSIKTTKIPGSILQPDASPSKAGTVSSIPVTIPENTSQSQVIGSEGGKNASTSATS
Chlorocebus_sabaeus           TETQSSIKTTKIPGSILQPDASPSKAGTVSSIPVTIPENTSQSQVIGTEGGKNASTSATS
Homo_sapiens                  TETQSSIKTTEIPGSVLQPDASPSKTGTLTSIPVTIPENTSQSQ--GTEGGKNASTSATS
Pongo_abelii                  TETQSSIKTTEIPGSILQPDASPSETGTLSSIPVTIPENTSQSQVIGTEGGKNASTSATS
Otolemur_garnetti             TDNQSLTKTTEIPGKTPQPDISHSKTSTLPSVSTTVTENLLPSQ--GTEDGKNASSPTTS
                              901234567890123456789012345678901234567890123--3456789012345
                              60        7         8         9       100           1

                                  |--- Transmembrane ---|
Brachyteles_arachnoides       PSYSSIILPVVIALIVITLSVFVLVGLYRVCWKSDPG--------QPQSDKESVKLLTVK
Callithrix_jacchus            PSYSSIILPVVIALIVITLSVFVLVGLYRVCWKSDPG--------RPQSDKESVKLLTVK
Aotus_nancymaae               PSYSSIILPVVIALIVITLSVFVLVGLYRVCWKSDPGT-AENGNDQPQSDKESVKLLTVK
Cebus_capucinus_imitator      PSYSSIILPVVIALIVITLSVFVLVGLYRVCWKSDPGT-AENGNDQPQSDKESVKLLTVK
Saimiri_boliviensis           PSYSSIILPVVIALIVITLSVFVLVSLYRVCWKSDPG--------QPQSDKESVKLLTVK
Macaca_nemestrina             RSYSSIILPVVIALIVITLSVFVLVGLYRMCWKADPGT-PENGNDQPQSDKESVKLLTVK
Chlorocebus_sabaeus           RSYSSIILPVVIALIVITLSVFVLVGLYRMCWKADPGT-PENGNDQPQSDKESVKLLTVK
Homo_sapiens                  RSYSSIILPVVIALIVITLSVFVLVGLYRMCWKADPG--------QPQSDKESVKLLTVK
Pongo_abelii                  RSYSSIILPVVIALIVITLSVFVLVGLYRMCWKADPGT-PENGNDQPQSDKESVKLLTVK
Otolemur_garnetti             PSYSSIILPVVIALIVITLSVFVVVGLYRMCWKTDPGTTQENGNDQPQSDKESVKLLTVK
                              7890123456789012345678901234567890123--------456789012345678
                            117  2         3         4         5                 6

Brachyteles_arachnoides       TISHES-EHSAQGKTKN
Callithrix_jacchus            TISHES-ELLLYGRRKN
Aotus_nancymaae               TISHESGEHSAQGKSKN
Cebus_capucinus_imitator      TISHESGEHSAQGKTKN
Saimiri_boliviensis           TISHES-EHSAQGKTKN
Macaca_nemestrina             TISHESGEHSAQGKTKN
Chlorocebus_sabaeus           TLSHESGEHSAQGKTKN
Homo_sapiens                  TISHES-EHSAQGKTKN
Pongo_abelii                  TISHESGEHSAQGKTKN
Otolemur_garnetti             TISHESGEHSAQGKSKN
                              901234-5678901234
                             170          8
```

FLVCR2

```
                                            |------ tandem repeats --------------
                                                                                *
Brachyteles_arachnoides     MVNEGPNEEERDDTPAPETALQADASVSVH------PSVSINPSVSINPSVSVHPSSSAQ
Callithrix_jacchus          MVNEGPNEEERDDTPAPETELQADGSVSVH------PSVSIDPSLSVHPSSSAHPSALAQ
Aotus_nancymaae             MVNEGPNEEERDDTPAPETELQADASVSVH------PSVSVNPSVSVHPSSSAHPSTLAQ
Cebus_capucinus_imitator    MVNEGPNEEERDDTPAPETELQADASVSVH------PSVSINPSVSVHPSSSAHPSALAQ
Saimiri_boliviensis         MVNEGPNEEERDDTPAPETELQADASVSVH------PSVSINPSVSVHPSSSAHPSALAQ
Macaca_nemestrina           MVNEGPNQEERDDTPAPESALQADPSVSVH------PSVSINPSVSVHPSSSAHPSALAQ
Chlorocebus_sabaeus         MVNEGPNQEERDDTPAPESALQADPSVSVH------PSVSINPSVSVHPSSSAHPSALAQ
Homo_sapiens                MVNEGPNQEESDDTPVPESALQADPSVSVH------PSVSINPSVSVHPSSSAHPSALAQ
Pongo_abelii                MVNEGPNKEERDDTPAPESALQADPSVSVHPSVSVHPSVSINPSVSVHPSSSAHPSALAQ
Otolemur_garnetti           ------------------------PSVSAH------PSVSIHPSVSVYPSSSAHPSALAQ
                                                    123456------789012345678901234567890
                                                                   1         2         3

                            ------------|
Brachyteles_arachnoides     PSALAQPSGSGPENLSVIKVSRRRWAVVLVFSCYSMCNAFQWIQYSSINNIFMNFYGVSA
Callithrix_jacchus          PSGLAHPSGSGSENLSVIKVSRRRWAVVLVFSCYSMCNAFQWIQYSSINNIFMHFYGVSA
Aotus_nancymaae             PSGLAHSSGSDPENLSVIKVSRRRWAVVLVFSCYSMCNAFQWIQYSSINNIFMHFYGVSA
Cebus_capucinus_imitator    PSGLARSSGSGPENLSVIKVSRRRWAVVLVFSCYSMCNAFQWIQYSSINNIFMHFYGVSA
Saimiri_boliviensis         PSGLAHSSGSGPENLSVIKVSRRRWAVVLVFSCYSMCNAFQWIQYSSINNIFMHFYGVSA
Macaca_nemestrina           PSGLAHPSSSGPEDLSVIKVSRRRWAVVLVFSCYSMCNAFQWIQYGSINNIFMHFYGVSA
Chlorocebus_sabaeus         PSGLAHPSSSGPEDLSVIKVSRRRWAVVLVFSCYSMCNAFQWIQYGSINNIFMHFYGVSA
Homo_sapiens                PSGLAHPSSSGPEDLSVIKVSRRRWAVVLVFSCYSMCNSFQWIQYGSINNIFMHFYGVSA
Pongo_abelii                PSGLAHPSSSDPEDLSVIKVSRRRWAVVLVFSCYSMCNAFQWIQYGSINNIFMHFYGVSA
Otolemur_garnetti           PSGLTQPSGSGPED-SVIKVSRRRWAVVLVFSCYSMCNAFQWIQYGSINNIFMKFYGVSA
                            12345678901234-567890123456789012345678901234567890123456789
                           31        4          5         6         7         8

Brachyteles_arachnoides     FAIDWLSMCYMLTYIPLLLPVAWLLEKFGLRTIALTGSALNCLGAWVKLGSLRPHLFPVT
Callithrix_jacchus          FAIDWLSMCYMLTYIPLLLPVAWLLEKFGLRTIALTGSALNCLGAWVKLGSLRPHLFPVT
Aotus_nancymaae             FAIDWLSMCYMLTYIPLLLPVAWLLEKFGLRTIALTGSALNCLGAWVKLGSLRPHLFPVT
Cebus_capucinus_imitator    FAIDWLSMCYMLTYIPLLLPVAWLLEKFGLRTIALTGSALNCLGAWVKLGSLRPHLFPVT
Saimiri_boliviensis         FAIDWLSMCYMLTYIPLLLPVAWLLEKFGLRTIALTGSALNCLGAWVKLGSLRPHLFPVT
Macaca_nemestrina           FAIDWLSMCYMLTYIPLLLPVAWLLEKFGLRTIALTGSALNCLGAWVKLGSLKPHLFPVT
Chlorocebus_sabaeus         FAIDWLSMCYMLTYIPLLLPVAWLLEKFGLRTIALTGSALNCLGAWVKLGSLKPHLFPVT
Homo_sapiens                FAIDWLSMCYMLTYIPLLLPVAWLLEKFGLRTIALTGSALNCLGAWVKLGSLKPHLFPVT
Pongo_abelii                FAIDWLSMCYMLTYIPLLLPVAWLLEKFGLRTIALTGSALNCLGAWVKLGSLKPHLFPVT
Otolemur_garnetti           FAIDWLSMCYMITYIPLLFPVAWLLEKFGLRTIAITGSALNCLGAWVKLGSLQPHLFPVT
                            012345678901234567890123456789012345678901234567890123456789
                           90       100         1         2         3         4

Brachyteles_arachnoides     VVGQLICSVAQVFILGMPSRIASVWFGANEVSTACSVAVFGNQLGIAIGFLVPPVLVPNI
Callithrix_jacchus          VVGQLICSVAQVFILGMPSRIASVWFGANEVSTACSVAVFGNQLGIAIGFLVPPVLVPNI
Aotus_nancymaae             VVGQLICSVAQVFILGMPSRIASVWFGANEVSTACSVAVFGNQLGIAIGFLVPPVLVPNI
Cebus_capucinus_imitator    VVGQLICSVAQVFILGMPSRIASVWFGANEVSTACSVAVFGNQLGIAIGFLVPPVLVPNV
Saimiri_boliviensis         VVGQLICSVAQVFILGMPSRIASVWFGANEVSTACSVAVFGNQLGIAIGFLVPPVLVPNI
Macaca_nemestrina           VVGQVICSVAQVFILGMPSRIASVWFGANEVSTACSVAVFGNQLGIAIGFLVPPVLVPNV
Chlorocebus_sabaeus         VVGQVICSVAQVFILGMPSRIASVWFGANEVSTACSVAVFGNQLGIAIGFLVPPVLVPNV
Homo_sapiens                VVGQLICSVAQVFILGMPSRIASVWFGANEVSTACSVAVFGNQLGIAIGFLVPPVLVPNI
Pongo_abelii                VVGQLICSVAQVFILGMPSRIASVWFGANEVSTACSVAVFGNQLGIAIGFLVPPVLVPNI
Otolemur_garnetti           VVGQVICSMAQVFILGMPSRLASVWFGANEVSTACSMAVFGNQLGIALGFLIPPVLVPSI
                            012345678901234567890123456789012345678901234567890123456789
                          150        6         7         8         9        200

                                                                                     *
Brachyteles_arachnoides     EDRDKLAYHISIMFYIIGGVATLLLILVIIVFKEKPKYPPSRAQSLSYALTSPDASYFSS
Callithrix_jacchus          EDQDKLAYHISIMFYIIGGVATLLLILVIIVFKEKPKYPPSRAQSLSYALASPDASYLSS
Aotus_nancymaae             EDRNKLAYHISIMFYIIGGVATLLLILVIIVFKEKPKYPPSRAQSLSYALASPDASYLSS
Cebus_capucinus_imitator    EDRDQLAYHISIMFYIIGGVATLLLILVIIVFKEKPKYPPSRAQSLSYALASPDASYLSS
Saimiri_boliviensis         EDRDKLAYHISIMFYIIGGVATLLLILVIIVFKEKPKYPPSRAQSLSYALASPDASYLSS
Macaca_nemestrina           EDRDKLAYHISIMFYIIGGVATLLLILVFIVFKEKPKYPPSRAQSLSYALTSPDASYLGS
Chlorocebus_sabaeus         EDRDKLAYHISIMFYIIGGVATLLLILVFIVFKEKPKYPPSRAQSLSX-LTSPDASYLGS
Homo_sapiens                EDRDELAYHISIMFYIIGGVATLLLILVIIVFKEKPKYPPSRAQSLSYALTSPDASYLGS
Pongo_abelii                EDRDKLAYHISIMFYIIGGVATLLLILVIIVFKEKPKYPPSRAQSLSYALTSPDASYLGS
Otolemur_garnetti           SDRDKLAYHISIMFYIIAGVATLLFILVVIVFKEKPKHPPSRAQSLSYALASPDASYLSS
                            012345678901234567890123456789012345678901234567-89012345678
                          210        2         3         4         5           6
```

```
Brachyteles_arachnoides     ITRLFKNLNFVLLVITYGLNAGAFYALSTLLNRMVIRHYPGEEVNAGRIGLTIVIAGMLG
Callithrix_jacchus          IVRLFKNLNFVLLVITYGLNAGAFYALSTLLNRMVIRHYPGEEVNAGRIGLTIVIAGMLG
Aotus_nancymaae             IIRLFKNLNFVLLVITYGLNAGAFYALSTLLNRMVIRHYPGEEVNAGRIGLTIVIAGMLG
Cebus_capucinus_imitator    ISRLFKNLNFVLLVITYGLNAGAFYALSTLLNRMVIRHYPGEEVNAGRIGLTIVIAGMLG
Saimiri_boliviensis         ISRLFKNLNFVLLVITYGLNAGAFYALSTLLNRMVIRHYPGEEVNAGRIGLTIVIAGMLG
Macaca_nemestrina           IAQLFKNLNFVLLVITYGLNAGAFYALSTLLNRMVIWHYPGEEVNAGRIGLTIVIAGMLG
Chlorocebus_sabaeus         IAQLFKNLNFVLLVITYGLNAGAFYALSTLLNRMVIWHYPGEEVNAGRIGLTIVIAGMLG
Homo_sapiens                IARLFKNLNFVLLVITYGLNAGAFYALSTLLNRMVIWHYPGEEVNAGRIGLTIVIAGMLG
Pongo_abelii                IARLFKNLNFVLLVITYGLNAGAFYALSTLLNRMVIWHYPGEEVNAGRIGLTIVIAGMLG
Otolemur_garnetti           IVRLFKNLNFVLLIITYGLNAGSFYSLSTLLNRMVISHYPDEEVNAGRIGLTIIIAGMLG
                            901234567890123456789012345678901234567890123456789012345678
                           270        8         9      300         1         2

Brachyteles_arachnoides     AVLSGIWLDRSKTYKETTLVVYIMTLVGMLVYTFTLNLGHLWVVFITAGTMGFFMTGYLP
Callithrix_jacchus          AVLSGIWLDRSKTYKETTLVVYIMTLVGMLVYTFTLNLGYLWVVFITAGTMGFFMTGYLP
Aotus_nancymaae             AVLSGIWLDRSKTYKETTLVVYIMTLVGMLVYTFTLNLGHLWVVFITAGSMGFFMTGYLP
Cebus_capucinus_imitator    AVLSGIWLDRSKTYKETTLVVYIMTLVGMLVYTFTLSLGHLWIVFITAGTMGFFMTGYLP
Saimiri_boliviensis         AVLSGIWLDRSKTYKETTLVVYIMTLVGMLVYTFTLNLGHLWVVFITAGTMGFFMTGYLP
Macaca_nemestrina           AVISGIWLDRSKTYKETTLVVYIMTLLGMVVYTFTLNLGHLWVVFITAGTMGFFMTGYLP
Chlorocebus_sabaeus         AVISGIWLDRSKTYKETTLVVYIMTLVGMVVYTFTLNLGHLWVVFITAGTMGFFMTGYLP
Homo_sapiens                AVISGIWLDRSKTYKETTLVVYIMTLVGMVVYTFTLNLGHLWVVFITAGTMGFFMTGYLP
Pongo_abelii                AVISGIWLDRSKTYKETTLVVYIMTLVGMVVYTFTLNLGHLWVVFITAGTMGFFMTGYLP
Otolemur_garnetti           AVISGIWLDRSKTYKETTLVVYIMTLVGMVVYTLTLSLGYLWVVFITAGTMGFFMTGYLP
                            901234567890123456789012345678901234567890123456789012345678
                           330        4         5         6         7         8

Brachyteles_arachnoides     LGFEFAVELTYPESEGISSGLLNISAQV-GLIF-------------ISAQV-GLIFF-IS
Callithrix_jacchus          LGFEFAVELTYPESEGISSGLLNISAQV-GIIF-------------ISAQV-GIIFF-IS
Aotus_nancymaae             LGFEFAVELTYPESEGISSGLLNISAQVFGIIFX------------ISAQVFGIIFF-IS
Cebus_capucinus_imitator    LGFEFAVELTYPESEGISSGLLNISAQVFGIIFX------------ISAQVFGIIFF-IS
Saimiri_boliviensis         LGFEFAVELTYPESEGISSGLLNISAQV-GIIF-------------ISAQV-GIIFF-IS
Macaca_nemestrina           LGFEFAVELTYPESEGISSGLLNISAQVFGIIF------------FISAQVFGIIFFFIS
Chlorocebus_sabaeus         LGFEFAVELTYPESEGISSGLLNISAQVFGIIF--------------SAQVFGIIFF--S
Homo_sapiens                LGFEFAVELTYPESEGISSGLLNISAQV-GIIF-------------ISAQV-GIIFF-IS
Pongo_abelii                LGFEFAVELTYPESEGISSGLLNISAQVFGIIFQVFGIIFSSGLLNISAQVFGIIFF---
Otolemur_garnetti           LGFEFAVELTYPESEGMSSGLLNVSAQVFGIIF.--------------------------
                            9012345678901234567890123456-7890
                           390       400        1         420

Brachyteles_arachnoides     AQV-GLIF----------------------------
Callithrix_jacchus          AQV-GIIF----------------------------
Aotus_nancymaae             AQVFGII-----------------------------
Cebus_capucinus_imitator    AQVFGII-----------------------------
Saimiri_boliviensis         AQV-GIIF----------------------------
Macaca_nemestrina           AQVFGIIF----------------------------
Chlorocebus_sabaeus         AQVFGIIF----------------------------
Homo_sapiens                AQV-GIIF----------------------------
Pongo_abelii                -QVFGIIFSSGLLNISAQVFGIIF------------
Otolemur_garnetti           ------------------------------------
```

GZMH

```
                                                                   Chymotrypsin (S1) signature
                                                                          |-----------
                                                             *  **    *
Brachyteles_arachnoides    MQPILFLLAFLLLPRADAGEIIGGHEAKPHSHPYMAYLAIWDQKS-KRCGGFLVREDFVL
Callithrix_jacchus         MQPFLLLSAFLLPPGTGTEEIIGGHEAKPHSYHYMAFVQFLDKKS-KGCGGILVRKDFVM
Aotus_nancymaae            MQPFLLLLAFLLPPGTGTEEIIGGHKAKPHSRPYMAFVQFLDKKSKKRCGGILVRKDFVM
Cebus_capucinus_imitator   ------------------EEIIGGHEAEPHSHPYMAFVQFLDKKSKKRCGGILVRKDFVM
Saimiri_boliviensis        MQPFLLLLAFLLPPGTGTEEIIGGHEAKPHSRPYMAFVQFLDKKS-KRCGGILVRKDFVM
Macaca_nemestrina          MQPFLLLLAFLLTPGAGTEEIIGGHEAKPHSRPYMAFVQFLDEKSRKRCGGILVRKDFVL
Chlorocebus_sabaeus        MQPFLLLLAFLLIPGAGTEEIIGGHEAKPHSRPYMAFVQFLDEKGKKRCGGILVRKDFVL
Homo_sapiens               MQPFLLLLAFLLTPGAGTEEIIGGHEAKPHSRPYMAFVQFLQEKS-KRCGGILVRKDFVL
Pongo_abelii               MQPFLLLLAFLLTPGAGTEEIIGGHEAKPHSRPYMAFVQFLHEKSRKRCGGILVRKDFVL
Otolemur_garnetti          MQPFLLLLAFLMPSRAGTEEIIGGNKAKPHSRPYMAFLQFLAKENRKRCGGVLVRNDFVL
                                             123456789012345678901234567-89012345678901
                                                      1         2          3         4

                                                                  Chymotrypsin (S1) signature
                           -----|                                      |---------------|
                                                             *
Brachyteles_arachnoides    TAAHCWGSSINVTLGAHNIKEQEKTQQSIPVRRTFCHPDYNPENFSSDIMLLQLERKAKQ
Callithrix_jacchus         TAAYCWGSSINVTLGAHNIKEQERTQQFIPVRRAFPHPAYNPEIFSSDIMLLQLERKAKQ
Aotus_nancymaae            TX--CWGSSINVTLGAHNIKEQERTQQFIPVRRAIPHPAYNPENFSSDIMLLQLERKAKQ
Cebus_capucinus_imitator   TAAHFWGSSINVTLGAHNIKEQERAQQFIPVRRAIPHPAYNPENFSSDIMLLQLERKAKQ
Saimiri_boliviensis        TAAHCWGSSINVTLGAHNIKEQERTQQFIPVKRAIPHPAYNPENFSSDIMLLKLERKAKQ
Macaca_nemestrina          TAAHCLGSSINVTLGAHNIKEQERTQQIIPVKRAIPHPAYNPENFSNDIMLLQLERKAKR
Chlorocebus_sabaeus        TAAHCLGSSINVTLGAHNIKEQERTQQFIPVKRAIPHPAYNPKNFSNDVMLLQLERKAKR
Homo_sapiens               TAAHCQGSSINVTLGAHNIKEQERTQQFIPVKRPIPHPAYNPKNFSNDIMLLQLERKAKW
Pongo_abelii               TAAHCQGRSINVTLGAHNIKEQERTQQFIPVERAIPHPAYNPKNFSNDIMLLQLERKAKW
Otolemur_garnetti          TAAHCWGSSMNVTLGAHNIKEQEKTQQVIHVRRSIPHEAYNPNTFANDIMLLQLERKARR
                           23--45678901234567890123456789012345678901234567890123456789
                          42         5         6         7         8         9

Brachyteles_arachnoides    TSAVQPLRLPSSKAQVKPGQVCSVAGWGRVSMG-TLATTLQEVLLTVEKDKECEYVFRDY
Callithrix_jacchus         TSAVQTLRQPSSKAQVKPGQVCSVAGWGRVSMG-TLATTLQEVLLTVQKDQECEYVFHAY
Aotus_nancymaae            TSAVQPLRLPSSKAQVKPGQVCSMAGWGRVSMG-TLATTLQEVLLTVQKDQECEYVFHDY
Cebus_capucinus_imitator   TPAVQPLRLPSSKAQVKPGQVCSVAGWGHVSVG-TLATT.QEALLTVQKDQECESVFHDD
Saimiri_boliviensis        TYAVQPLRLPSSKAQVKPGQVCSVAGWGRVSMD-TLATTLQEVLLTVQKDQECEYVFHAY
Macaca_nemestrina          TTAVQPLRLPSSKVQVKPGQVCSVAGWGRVSTG-TLAATLQEVLLTVQKDWECERLFHAN
Chlorocebus_sabaeus        TTAVQPLRLPSSKVQVRPGQVCSVAGWGRVSTG-TLATTLQEVLLTVQKDWECERLFHGN
Homo_sapiens               TTAVRPLRLPSSKAQVKPGQLCSVAGWGYVSMS-TLATTLQEVLLTVQKDCQCERLFHGN
Pongo_abelii               TTAVRPLRLPSSKAQVKPGQVCSVAGWGYVSVS-TLATTLQEVSLTVQKDSECERLFPDN
Otolemur_garnetti          TTAVQPLKLPSSTGGAMPEMVCSMAGWGRTAPGGKLSDTLQEVMLTVQKDQECKSRFPNN
                           012345678901234567890123456789012-345678901234567890123456778
                         100         1         2         3          4         5

Brachyteles_arachnoides    YSRATEICVGDPEKTK
Callithrix_jacchus         YSRATKICVGDPKKTK
Aotus_nancymaae            YSRATEICVGDPKKTK
Cebus_capucinus_imitator   YSRATEICVGDPNMTK
Saimiri_boliviensis        YSRATEICVGDPEKTK
Macaca_nemestrina          YSKATEICVGDPKTTK
Chlorocebus_sabaeus        YSKATEICVGDPKTTK
Homo_sapiens               YSRATEICVGDPKKTQ
Pongo_abelii               YSRATEICVGDPKKTQ
Otolemur_garnetti          YNRITQICVGDPKKK*
                           9012345678901234
                          160         7
```

HLA-DRB1

```
                           |  Signal peptide  |
                                                                           *
Brachyteles_arachnoides     MAALTVTLMVLSSPLALAGDTRPRFLEHVKFECYLFNGTE--RYLVRAIYNQEEYVRFDS
Callithrix_jacchus          VAALTVTLMVLSSPLALAGDTRPRYLEQRKAECHHFNGTE--RLLERYFYNQEEFVRFDS
Aotus_nancymaae             -AALTVTLMVLSSPLALVGDTRPRFLELVKHECHFFNGTERVRYLDRYIHNQEEVVRFDS
Cebus_capucinus_imitator    MAALTVTLMVLSSPLALAGDTRPRFLEQAKSECHFFNGTERVRYLQRYFYNQEEYVRFDS
Saimiri_boliviensis         MAALTVTLMVMSSPLALAGDTRPRFLKQVKHECHHFKGME--RFLFRHIYNQEEYVRFDS
Macaca_nemestrina           MAALTMTLMVLSSPLALAGDTRARFLEQVKHECHFFNGTERVRFLDRYFYNQEEYVRFDS
Chlorocebus_sabaeus         MAALTVTLMVLSSPLALAGDTRPRFLEQVKSECHFFNGTERVRYLDRYFYNQEEYVRFDS
Homo_sapiens                MAALTVTLMVLSSPLALAGDTRPRFLEQVKHECHFFNGTE--RFLDRYFYHQEEYVRFDS
Pongo_abelii                MAALTVTLMVLSSPLALSGDTQTRFLEQVKSECHFFNGTERVRFLERYFYNQEEYVHFDS
                             123456789012345678901234567890123456789--012345678901234567
                                      1         2         3           4         5

                                                                                  |-----
                                         *                 *
Brachyteles_arachnoides     DVGEFRAVTELGRRSAEYYNSQKDFLERMRASVDT-CRHNYGVGESFTVQRRVQPKVTVY
Callithrix_jacchus          DVGEFRAVTELGRPVAESWNSRKDILEQRRAAVDT-CRHNYEGFETFLVPRRVQPKVTVY
Aotus_nancymaae             DVGEYRAVTELGRPDAEYWNSQKDYVEQKRGQVDNYCRHNYGVFESFTVQRRVQPKMTVY
Cebus_capucinus_imitator    DVGEYRAVTELGRPSAEYWNSQKELLEQKRGQVDNYCRHNYRVGESFTVQRRVQPKVTVY
Saimiri_boliviensis         DVGEFRAVTELGRPDAESWNSQEDILEQMRAEVDK-CRFNYGVLERFLVPQRVQPKVTVY
Macaca_nemestrina           DVGEYRAVTELGRPDAEYWNGQKDILEQKRGQVDNYCRHNYGVAESFTVQRRVQAKVTVY
Chlorocebus_sabaeus         DVGEFRAVSELGRPDAENWNSRKDILEDERASVDTYCRHNYGVGESFTVQRRVQPKVTVY
Homo_sapiens                DVGEYRAVTELGRPDAEYWNSQKDLLEQRRAEVDT-CRHNYGVVESFTVQRRVYPEVTVY
Pongo_abelii                DVGEYRAVTELGRPDAEYWNSQKNLLEQKRGQVDNYCRHNYGVGESFTVQRRVQPKVTVY
                            89012345678901234567890123456789012-345678901234567890123456
                           58 6         7         8         9         100        1

                            ------------------- Ig-like C1-type ------------------------
Brachyteles_arachnoides     PAKTQPLQHHNLLVCSVSGFYPGSIEVRWFRNDQEEKAGVVSTGLIQNGDWTFQTLVMLE
Callithrix_jacchus          PAKTQPLQHHNLLVCSVSGFYPGSIEVRWFRNDQEEKAGVVSTGLIQNGDWTFQTLVMLE
Aotus_nancymaae             PAKTQPLQHHNLLVCSVSGFYPGSIEVRWFRNNQEEKAGVVSTGLIQNGDWTFQTLVMLE
Cebus_capucinus_imitator    PAKTQPLQHHSLLVCSVSGFYPGSIEVRWFRNGQEEKAGVVSTGLIQNGDWTFQTLVMLE
Saimiri_boliviensis         PAKTQPLQHHNLLVCSVSGFYPGSIEVRWFRNDQEEKAGVVSTGLIQNGDWTFQTLVMLE
Macaca_nemestrina           PAKTQPLQHHNLLVCSVNGFYPGSIEVRWFRNGQEEKAGVVSTGLIQNGDWTFQTLVMLE
Chlorocebus_sabaeus         PAKTQPLQHHTLLVCSVNGFYPGSIEVRWFRNGQEEKAGVVSTGLIQNGDWTFQTLVMLE
Homo_sapiens                PAKTQPLQHHNLLVCSVNGFYPGSIEVRWFRNGQEEKTGVVSTGLIQNGDWTFQTLVMLE
Pongo_abelii                PSKTQPLQHHNLLVCSVNGFYPGSIEVRWFRNGQEEKTGVVSTGLIQNGDWTFQTLVMLE
                            789012345678901234567890123456789012345678901234567890123456
                          117  2         3         4         5         6         7

                            ----------------------|
Brachyteles_arachnoides     TVPQSGEVYTCQVEHPSVMSPITVQWRARSESAQSKMLSGVGGFVLGLLFLGAGLFIYFR
Callithrix_jacchus          TVPQSGEVYICQVEHPSVTSPLTVQWRAQSESAQSKMLSGVGGFVLGLLFLGAGLFIYFR
Aotus_nancymaae             TVPQSGEVYTCQVEHPSVTTPITVQWRAQSESAQSKMLSGVGGFVLGLLFLGAGLFIYFR
Cebus_capucinus_imitator    TVPQSGEVYTCQVEHPSVMSPLTVQWRAQSESAQSKMLSGVGGFVLGLLFLGAGLFIYFR
Saimiri_boliviensis         TVPQSGEVYTCQVEHPSVMSPITVQWRAQSESAQRKMLSGVGGFVLGLLFLGAGLFIYFR
Macaca_nemestrina           TVPRSGEVYTCQVEHPSVTSPLTVEWRAQSESAQSKMLSGVGGFVLGLLFLGAGLFIYFR
Chlorocebus_sabaeus         TVPRSGEVYTCQVEHPSVTSPLTVEWRARSESAQSKMLSGVGGFVLGLLFLGAGLFIYFR
Homo_sapiens                TVPRSGEVYTCQVEHPSLTSPLTVEWRARSESAQSKMLSGVGGFVLGLLFLGAGLFIYFR
Pongo_abelii                TVPRSGEVYTCQVEHPSVTSPLTVEWRARSESAQSKMLSGVGGFVLGLLFLAAGLFIYFR
                            789012345678901234567890123456789012345678901234567890123456
                          177  8         9        200        1         2         3

Brachyteles_arachnoides     NQKGGSGLQPT
Callithrix_jacchus          NQKGRSGLQPT
Aotus_nancymaae             NQKGGSGLQPT
Cebus_capucinus_imitator    NQKGRSGLQPT
Saimiri_boliviensis         NQKGHSGLQPT
Macaca_nemestrina           NQKGHSGLQPT
Chlorocebus_sabaeus         NQKGHSGLQPT
Homo_sapiens                NQKGHSGLQPT
Pongo_abelii                NQKGHSGLPPT
                            78901234567
                          237  4
```

MEF2D

```
                             |------- Transcription factor MADS-box -----------------|
Brachyteles_arachnoides      MGRKKIQIQRITDERNRQVTFTKRKFGLMKKAYELSVLCDCEIALIIFNHSNKLFQYAST
Callithrix_jacchus           MGRKKIQIQRITDERNRQVTFTKRKFGLMKKAYELSVLCDCEIALIIFNHSNKLFQYAST
Aotus_nancymaae              MGRKKIQIQRITDERNRQVTFTKRKFGLMKKAYELSVLCDCEIALIIFNHSNKLFQYAST
Cebus_capucinus_imitator     MGRKKIQIQRITDERNRQVTFTKRKFGLMKKAYELSVLCDCEIALIIFNHSNKLFQYAST
Saimiri_boliviensis          MGRKKIQIQRITDERNRQVTFTKRKFGLMKKAYELSVLCDCEIALIIFNHSNKLFQYAST
Macaca_nemestrina            MGRKKIQIQRITDERNRQVTFTKRKFGLMKKAYELSVLCDCEIALIIFNHSNKLFQYAST
Papio_anubis                 MGRKKIQIQRITDERNRQVTFTKRKFGLMKKAYELSVLCDCEIALIIFNHSNKLFQYAST
Chlorocebus_sabaeus          MGRKKIQIQRITDERNRQVTFTKRKFGLMKKAYELSVLCDCEIALIIFNHSNKLFQYAST
Homo_sapiens                 MGRKKIQIQRITDERNRQVTFTKRKFGLMKKAYELSVLCDCEIALIIFNHSNKLFQYAST
Pongo_abelii                 MGRKKIQIQRITDERNRQVTFTKRKFGLMKKAYELSVLCDCEIALIIFNHSNKLFQYAST
                             012345678901234567890123456789012345678901234567890123456789
                                       1         2         3         4         5

                                                         |-------------------------------
Brachyteles_arachnoides      DMDKVLLKYTEYNEPHESRTNADIIETLRKKGFNGCDSPEPDGEDSLEQSPLLEDKYRRA
Callithrix_jacchus           DMDKVLLKYTEYNEPHESRTNADIIETLRKKGFNGCDSPEPDGEDSLEQSPLLEDKYRRA
Aotus_nancymaae              DMDKVLLKYTEYNEPHESRTNADIIETLRKKGFNGCDSPEPDGEDSLEQSPLLEDKYRRA
Cebus_capucinus_imitator     DMDKVLLKYTEYNEPHESRTNADIIETLRKKGFNGCDSPEPDGEDSLEQSPLLEDKYRRA
Saimiri_boliviensis          DMDKVLLKYTEYNEPHESRTNADIIETLRKKGFNGCDSPEPDGEDSLEQSPLLEDKYRRA
Macaca_nemestrina            DMDKVLLKYTEYNEPHESRTNADIIETLRKKGFNGCDSPEPDGEDSLEQSPLLEDKYRRA
Papio_anubis                 DMDKVLLKYTEYNEPHESRTNADIIETLRKKGFNGCDSPEPDGEDSLEQSPLLEDKYRRA
Chlorocebus_sabaeus          DMDKVLLKYTEYNEPHESRTNADIIETLRKKGFNGCDSPEPDGEDSLEQSPLLEDKYRRA
Homo_sapiens                 DMDKVLLKYTEYNEPHESRTNADIIETLRKKGFNGCDSPEPDGEDSLEQSPLLEDKYRRA
Pongo_abelii                 DMDKVLLKYTEYNEPHESRTNADIIETLRKKGFNGCDSPEPDGEDSLEQSPLLEDKYRRA
                             012345678901234567890123456789012345678901234567890123456789
                            60         7         8         9        100        1

                             Holliday junction regulator
                             ---------------------------------|
Brachyteles_arachnoides      SEELDGLFRRYGSAVPAPNFAMPVTVPVSNQSSLQFSNPSGSLVTPSLVTSSLTDPRLLS
Callithrix_jacchus           SEELDGLFRRYGSAVPAPNFAMPVTVPVSNQSSLQFSNPSGSLVTPSLVTSSLTDPRLLS
Aotus_nancymaae              SEELDGLFRRYGSAVPAPNFAMPVTVPVSNQSSLQFSNPSGSLVTPSLVTSSLTDPRLLS
Cebus_capucinus_imitator     SEELDGLFRRYGSAVPAPNFAMPVTVPVSNQSSLQFSNPSGSLVTPSLVTSSLTDPRLLS
Saimiri_boliviensis          SEELDGLFRRYGSAVPAPNFAMPVTVPVSNQSSLQFSNPSGSLVTPSLVTSSLTDPRLLS
Macaca_nemestrina            SEELDGLFRRYGSAVPAPNFAMPVTVPVSNQSSLQFSNPSGSLVTPSLVTSSLTDPRLLS
Papio_anubis                 SEELDGLFRRYGSAVPAPNFAMPVTVPVSNQSSLQFSNPSGSLVTPSLVTSSLTDPRLLS
Chlorocebus_sabaeus          SEELDGLFRRYGSAVPAPNFAMPVTVPVSNQSSLQFSNPSGSLVTPSLVTSSLTDPRLLS
Homo_sapiens                 SEELDGLFRRYGSTVPAPNFAMPVTVPVSNQSSLQFSNPSGSLVTPSLVTSSLTDPRLLS
Pongo_abelii                 SEELDGLFRRYGSAVPAPNFAMPVTVPVSNQSSLQFSNPSGSLVTPSLVTSSLTDPRLLS
                             012345678901234567890123456789012345678901234567890123456789
                           120         3         4         5         6         7

Brachyteles_arachnoides      PQQPALQRNSVSPGLPQRPASAGAMLGGDLNSANGACPSPVGNGYVSARASPGLLPVANG
Callithrix_jacchus           PQQPALQRNSVSPGLPQRPASAGAMLGGDLNSANGACPSPVGNGYVSARASPGLLPVANG
Aotus_nancymaae              PQQPALQRNSVSPGLPQRPASAGAMLGGDLNSANGACPSPVGNGYVSARASPGLLPVANG
Cebus_capucinus_imitator     PQQPALQRNSVSPGLPQRPASAGAMLGGDLNSANGACPSPVGNGYVSARASPGLLPVANG
Saimiri_boliviensis          PQQPALQRNSVSPGLPQRPASAGAMLGGDLNSANGACPSPVGNGYVSARASPGLLPVANG
Macaca_nemestrina            PQQPALQRNSVSPGLPQRPASAGAMLGGDLNSANGACPSPVGNGYVSARASPGLLPVANG
Papio_anubis                 PQQPALQRNSVSPGLPQRPASAGAMLGGDLNSANGACPSPVGNGYVSARASPGLLPVANG
Chlorocebus_sabaeus          PQQPALQRNSVSPGLPQRPASAGAMLGGDLNSANGACPSPVGNGYVSARASPGLLPVANG
Homo_sapiens                 PQQPALQRNSVSPGLPQRPASAGAMLGGDLNSANGACPSPVGNGYVSARASPGLLPVANG
Pongo_abelii                 PQQPALQRNSVSPGLPQRPASAGAMLGGDLNSANGACPSPVGNGYVSARASPGLLPVANG
                             012345678901234567890123456789012345678901234567890123456789
                           180         9        200        1         2         3

Brachyteles_arachnoides      NSLNKVIPAKSPPPPTHSTQLGAPSRKPDLRVITSQAGKGLMHHLNNAQRLGVSQSTHSL
Callithrix_jacchus           NSLNKVIPAKSPPPPTHSTQLGAPSRKPDLRVITSQAGKGLMHHLNNAQRLGVSQSTHSL
Aotus_nancymaae              NSLNKVIPAKSPPPPTHSTQLGAPSRKPDLRVITSQAGKGLMHHLNNAQRLGVSQSTHSL
Cebus_capucinus_imitator     NSLNKVIPAKSPPPPTHSTQLGAPSRKPDLRVITSQAGKGLMHHLNNAQRLGVSQSTHSL
Saimiri_boliviensis          NSLNKVIPAKSPPPPTHSTQLGAPSRKPDLRVITSQAGKGLMHHLNNAQRLGVSQSTHSL
Macaca_nemestrina            NSLNKVIPAKSPPPPTHGTQLGAPSRKPDLRVITSQAGKGLMHHLNNAQRLGVSQSTHSL
Papio_anubis                 NSLNKVIPAKSPPPPTHGTQLGAPSRKPDLRVITSQAGKGLMHHLNNAQRLGVSQSTHSL
Chlorocebus_sabaeus          NSLNKVIPAKSPPPPTHGTQLGAPSRKPDLRVITSQAGKGLMHHLNNAQRLGVSQSTHSL
Homo_sapiens                 NSLNKVIPAKSPPPPTHSTQLGAPSRKPDLRVITSQAGKGLMHHLNNAQRLGVSQSTHSL
Pongo_abelii                 NSLNKVIPAKSPPPPTHSTQLGAPSRKPDLRVITSQAGKGLMHHLNNVQRLGVSQSTHSL
                             012345678901234567890123456789012345678901234567890123456789
                           240         5         6         7         8         9
```

```
                                                                                   |--
Brachyteles_arachnoides   TTPVVSVATPSLLSQGLPFSSMPTAYNTDYQLTSAELSSLPAFSSPGGLSLGNVTAWQQP
Callithrix_jacchus        TTPVVSVATPSLLSQGLPFSSMPTAYNTDYQLTSAELSSLPAFSSPGGLSLGNVTAWQQP
Aotus_nancymaae           TTPVVSVATPSLLSQGLPFSSMPTAYNTDYQLTSAELSSLPAFSSPGGLSLGNVTAWQQP
Cebus_capucinus_imitator  TTPVVSVATPSLLSQGLPFSSMPTAYNTDYQLTSAELSSLPAFSSPGGLSLGNVTAWQQP
Saimiri_boliviensis       TTPVVSVATPSLLSQGLPFSSMPTAYNTDYQLTSAELSSLPAFSSPGGLSLGNVTAWQQP
Macaca_nemestrina         TTPVVSVATPSLLSQGLPFSSMPTAYNTDYQLTSAELSSLPAFSSPGGLSLGNVTAWQQP
Papio_anubis              TTPVVSVATPSLLSQGLPFSSMPTAYNTDYQLTSAELSSLPAFSSPGGLSLGNVTAWQQP
Chlorocebus_sabaeus       TTPVVSVATPSLLSQGLPFSSMPTAYNTDYQLTSAELSSLPAFSSPGGLSLGNVTAWQQP
Homo_sapiens              TTPVVSVATPSLLSQGLPFSSMPTAYNTDYQLTSAELSSLPAFSSPGGLSLGNVTAWQQP
Pongo_abelii              TTPVVSVATPSLLSQGLPFSSMPTAYNTDYQLTSAELSSLPAFSSPGGLSLGNVTAWQQP
                          012345678901234567890123456789012345678901234567890123456789
                        300         1         2         3         4         5

                          -- Gln/Pro-rich --------------------|
                                    *        *
Brachyteles_arachnoides   QQPQQPQQPQQPQ---PQQPQPQPQQPQQPQQPPQPQSHLVPVSLSNLIPGSPLPHVGAA
Callithrix_jacchus        QQPQQPQQPQPPQ---PQPQQPQPQQPQQPQQPPQPQSHLVPVSLSNLIPGSPLPHVGAA
Aotus_nancymaae           QQPQQPQQPQPPQQQPPQPQQPQPQQPQQPQQPPQPQSHLVPVSLSNLIPGSPLPHVGAA
Cebus_capucinus_imitator  QQPQQPQQPQPPQQQPPQPQQPQPQQPQQPQQPPQPQSHLVPVSLSNLIPGSPLPHVGAA
Saimiri_boliviensis       QQPQQPQQPQPPQ---PQPQQPQPQQPQQPQQPPQPQSHLVPVSLSNLIPGSPLPHVGAA
Macaca_nemestrina         QQPQQPQQPQPPQQQPPQPQQPQPQQPQQPQQPPQQQSHLVPVSLSNLIPGSPLPHVGAA
Papio_anubis              QQPQQPQQPQPPQQQPPQPQQPQPQQPQQPQQPPQQQSHLVPVSLSNLIPGSPLPHVGAA
Chlorocebus_sabaeus       QQPQQPQQPQPPQQQPPQPQQPQPQQPQQPQQPPQQQSHLVPVSLSNLIPGSPLPHVGAA
Homo_sapiens              QQPQQPQQPQPPQ---PQPQQPQPQQPQQPQQPPQQQSHLVPVSLSNLIPGSPLPHVGAA
Pongo_abelii              QQPQQPQQPQPPQQQPPQPQQPQPQQPQQPQQPPQQQSHLVPVSLSNLIPGSPLPHVGAA
                          0123456789012---345678901234567890123456789012345678901234
                        360         7            8         9       400         1

Brachyteles_arachnoides   LTVTTHPHISIKSEPVSPSRERSPAPPPPAVFPAARPEPGDGLSSPAGGSYETGDRDDGR
Callithrix_jacchus        LTVTTHPHISIKSEPVSPSRERSPAPPPPAVFPAARPEPGDGLSSPAGGSYETGDRDDGR
Aotus_nancymaae           LTVTTHPHISIKSEPVSPSRERSPAPPPPAVFPAARPEPGDGLSSPAGGSYETGDRDDGR
Cebus_capucinus_imitator  LTVTTHPHISIKSEPVSPSRERSPAPPPPAVFPAARPEPGDGLSSPAGGSYETGDRDDGR
Saimiri_boliviensis       LTVTTHPHISIKSEPVSPSRERSPAPPPPAVFPAARPEPGDGLSSPAGGSYETGDRDDGR
Macaca_nemestrina         LTVTTHPHISIKSEPVSPSRERSPAPPPPAVFPAARPEPGDGLSSPAGGSYETGDRDDGR
Papio_anubis              LTVTTHPHISIKSEPVSPSRERSPAPPPPAVFPAARPEPGDGLSSPAGGSYETGDRDDGR
Chlorocebus_sabaeus       LTVTTHPHISIKSEPVSPSRERSPAPPPPAVFPAARPEPGDGLSSPAGGSYETGDRDDGR
Homo_sapiens              LTVTTHPHISIKSEPVSPSRERSPAPPPPAVFPAARPEPGDGLSSPAGGSYETGDRDDGR
Pongo_abelii              LTVTTHPHISIKSEPVSPSRERSPAPPPPAVFPAARPEPGDGLSSPAGGSYETGDRDDGR
                          789012345678901234567890123456789012345678901234567890123456
                        417  2         3         4         5         6         7

Brachyteles_arachnoides   GDFGPTLGLLRPAAEPEAEGSAVKRMRLDTWTLK
Callithrix_jacchus        GDFGPTLGLLRPAAEPEAEGSAVKRMRLDTWTLK
Aotus_nancymaae           GDFGPTLGLLRPAAEPEAEGSAVKRMRLDTWTLK
Cebus_capucinus_imitator  GDFGPTLGLLRPAAEPEAEGSAVKRMRLDTWTLK
Saimiri_boliviensis       GDFGPTLGLLRPAAEPEAEGSAVKRMRLDTWTLK
Macaca_nemestrina         GDFGPTLGLLRPAPEPEAEGSAVKRMRLDTWTLK
Papio_anubis              GDFGPTLGLLRPAPEPEAEGSAVKRMRLDTWTLK
Chlorocebus_sabaeus       GDFGPTLGLLRPAPEPEAEGSAVKRMRLDTWTLK
Homo_sapiens              GDFGPTLGLLRPAPEPEAEGSAVKRMRLDTWTLK
Pongo_abelii              GDFGPTLGLLRPAPEPEAEGSAVKRMRLDTWTLK
                          7890123456789012345678901234567890
                        477  8         9       500         1
```

MINDY2

```
                                         *
Brachyteles_arachnoides    MESSPESLQPLEHGVLAGPASGIGSSQEGLQETRLAAGDGPGVWAAESGGGNGQGAAAAR
Callithrix_jacchus         MESSPESLQPLEHGVAARPASGIGSSREGLQETRLAAGDGPGVWAAESGGGNGQGAAAAR
Aotus_nancymaae            MESSPESLQPLEHGVAAGPASGIGSSQEGLQETRLAAEDGPGVWAAESSGGNGQGAAAAR
Cebus_capucinus_imitator   MESSPESLQPLEHGVAAGPASGIGSSQEGLHETRLAAGDGPGVWAAESGGGNGQGAAAAG
Saimiri_boliviensis        MESSPESLQPLEHGVAAGPASRIGSSQEGLQETRLAAGDGPGVWAAESGGGNGQGAAAAR
Macaca_nemestrina          MESSPESLQPLEHGVAAGPASGTGSSQEGLQETRLAAGDGPGVWAAESSGGNGLGAAAAS
Papio_anubis               MESSPESLQPLEHGVAAGPASGTGSSQEGLQETRLAAGDGPGVWAAESSGGNGLGAAAAS
Chlorocebus_sabaeus        MESSPESLQPLEHGVAAGPASGTGSSQEGLQETRLAAGDGAGVWAAESSGGNGLGAAAAR
Homo_sapiens               MESSPESLQPLEHGVAAGPASGTGSSQEGLQETRLAAGDGPGVWAAETSGGNGLGAAAAR
Pongo_abelii               MESSPESLQPLEHGVAAGPASGTGSSQEGLQETRLAAGDGPGVWAAENSGGNGLGAAAAR
Otolemur_garnetti          MESGPESLQPLEDRVAAGPAPGTGSLQEGQPETRLAAGDGPGVWAAESGGGNGQGAAAAE
                           123456789012345678901234567890123456789012345678901234567890
                                    1         2         3         4         5         6

                                *                                       *
Brachyteles_arachnoides    RSLPDAASPAGSPEVPGPCSSAPGLDLKDSALESPAAAEVPLRGQCKVTASPETAEAGVG
Callithrix_jacchus         RSLPDSASPAGSPEVPGPCSSAPGLDLKDSALESPAAAEVPPRGQYKVTASPETAEAGVG
Aotus_nancymaae            RSLPDSASPAGSPEAPGPYSSAPGLDLKDGALESPAAAEVPLRGQYKVTASPETAEAGVG
Cebus_capucinus_imitator   RSLPDSASSAGCPEVSGPCSSAPGLDLKNSALESLAAAEVPLRGQYKVTASPKTAEAGVG
Saimiri_boliviensis        RSLPDSASPAGCPEAPGPCSSAPGLDLKDSALECPAAAEVPLRGQYKVTASPETAEARVG
Macaca_nemestrina          RSLPDSASPAGSPEVPGPCSSSPGLDLKDSGVESPAAAEAPLRGQYKVTASPETAVAGVG
Papio_anubis               RSLPDSASPAGSPEVPGPCSSSPGLDLKDSGVESPAAAEAPLRGQYKVTASPETAVAGVG
Chlorocebus_sabaeus        RSLPDSSSPAGSPEVPRPCSSSPGLDLKDSGLESPAAAEAPLRGQYKVTASPETAVAGVG
Homo_sapiens               RSLPDSASPAGSPEVPGPCSSSAGLDLKDSGLESPAAAEAPLRGQYKVTASPETAVAGVG
Pongo_abelii               RSLPDSASPAGSLEVPGPCSSSPGLDLKDSGLESPAAAEAPLRGQYKVTASPETAVAGVG
Otolemur_garnetti          RSLPDSASPAGSLQVPGPCSFFADLGVQESALESLAAARPPLRGQHKVTASPETAEAGVG
                           123456789012345678901234567890123456789012345678901234567890
                          61        7         8         9        100        1         2

                                                    *
Brachyteles_arachnoides    HELGPAGDAGVRPDLASTCQAELTATGSEEPSSAGGLSSSCSDPSPPGESPSLDSLESFS
Callithrix_jacchus         HGLGPAGDAGVRPDLASTCQAEVTAAGSEEPSSAGGLSSSCSDPSPPGESPSLDSLESFS
Aotus_nancymaae            HELGPAGDAGVRPDLASTCQAELTAAGSEEPSSAGGLSSSCSDPSPPGESPSLDSLESFS
Cebus_capucinus_imitator   HELGPAGDAGVRPDLASTCQAELTAAGSEEPSSAGGLSSSCSDPSPPGESPSLDSLESFS
Saimiri_boliviensis        HELGPAGDAGVRPDLASTCQTELTAAGSEEPSSAGGLSSSCSDPSPPGESPSLDSLESFS
Macaca_nemestrina          HELGPAGDAGDRPDLAGTCQAELTAAGSEEPSSAGGLSSSCSDPTPPGESPSLDSLESFS
Papio_anubis               HELGPAGDAGDRPDLAGTCQAELTAAGSEEPSSAGGLSSSCSDPTPPGESPSLDSLESFS
Chlorocebus_sabaeus        HELGPAGDAGDRPDLAGTCQAELTAAGSEEPSSAGGLSSSCSDPTPPGESPSLDSLESFS
Homo_sapiens               HELGTAGDAGARPDLAGTCQAELTAAGSEEPSSAGGLSSSCSDPSPPGESPSLDSLESFS
Pongo_abelii               HELGPAGDAGARPDLAGTCQAELTAAGSEEPSSAGGLSSSCSDPSPPGESPSLDSLESFS
Otolemur_garnetti          HGLGPAGDAGARPDPAGTCRTELATSGSEDPSSAGGLSSSCSDPSPPGESPSLDSLESFS
                           123456789012345678901234567890123456789012345678901234567890
                         121          3         4         5         6         7         8

Brachyteles_arachnoides    NLHSFPSSCEFNSE--AENRVP---EEEEGAAVLPGAVPLCK------EEEETAQVLAAS
Callithrix_jacchus         NLHSFPSSCEFNSE--AEHRVP---EEEEGAAVLPGAVPLCK------EEEETAQVLAAS
Aotus_nancymaae            NLHSFPSSCEFNSEEGAENRVP--EEEEEGAAVLPGAVPLCK---EEEEEEETAQVLAAS
Cebus_capucinus_imitator   NLHSFPSSCEFNSEEGAENRVPEEEEEEEGAAVLPGAVPLCKEEEEEEEEEETAQVLAAS
Saimiri_boliviensis        NLHSFPSSCEFNSE--AENRVP---EEEEGAAVLPGAVPLCK------EEEENAQVLAAS
Macaca_nemestrina          NLHSFPSSCEFNSEEGAENRVP---EEEEGAAVLPGAVPLCK---GEEEEEETAQVLAAS
Papio_anubis               NLHSFPSSCEFNSEEGAENRVP---EEEEGAAVLPGAVPLCK---GEEEEEETAQVLAAS
Chlorocebus_sabaeus        NLHSFPSSCEFNSEEGAENRVP---EEEEGAAVLPGAVPLCK---GEEEEEETAQVLAAS
Homo_sapiens               NLHSFPSSCEFNSE--AENRVP---EEEEGAAVLPGAVPLCK------EGEETAQVLAAS
Pongo_abelii               NLHSFPSSCEFNSEEGAENRVP---EEEEGAAVLPGAVPLCK----EEEGEETAQVLAAS
Otolemur_garnetti          NLHSFPSSSEFNSEEGAENRVP--EEEECAAAAVPGAVPLCQ-----EEEAETAQVLAAS
                           12345678901234--567890---1234567890123456------7890123456789
                         181        9        200             1                2
```

```
                                                                  |----------------------
Brachyteles_arachnoides     KERFPGQSVYHIKWIQWKEENTPIITQNENGPCPLLAILNVLLLAWKVKLPPMMEIITAE
Callithrix_jacchus          KERFPGQSVYHIKWIQWKEENTPIITQNENGPCPLLAILNVLLLAWKVKLPPMMEIITAE
Aotus_nancymaae             KERFPGQSVYHIKWIQWKEENTPIITQNENGPCPLLAILNVLLLAWKVKLPPMMEIITAE
Cebus_capucinus_imitator    KERFPGQSVYHIKWIQWKEENTPIITQNENGPCPLLAILNVLLLAWKVKLPPMMEIITAE
Saimiri_boliviensis         KERFPGQSVYHIKWIQWKEENTPIITQNENGPCPLLAILNVLLLAWKVKLPPMMEIITAE
Macaca_nemestrina           KERFPGQSVYHIKWIQWKEENTPIITQNENGPCPLLAILNVLLLAWKVKLPPMMEIITAE
Papio_anubis                KERFPGQSVYHIKWIQWKEENTPIITQNENGPCPLLAILNVLLLAWKVKLPPMMEIITAE
Chlorocebus_sabaeus         KERFPGQSVYHIKWIQWKEENTPIITQNENGPCPLLAILNVLLLAWKVKLPPMMEIITAE
Homo_sapiens                KERFPGQSVYHIKWIQWKEENTPIITQNENGPCPLLAILNVLLLAWKVKLPPMMEIITAE
Pongo_abelii                KERFPGQSVYHIKWIQWKEENTPIITQNENGPCPLLAILNVLLLAWKVKLPPMMEIITAE
Otolemur_garnetti           KERFPGQSVYHIKWIQWKEENTPIITQNENGPCPLLAILNVLLLAWKVKLPPMMEIITAE
                            012345678901234567890123456789012345678901234567890123456789
                          230         4         5         6         7         8

                            ------------- MINDY deubiquitinase domain ------------------
Brachyteles_arachnoides     QLMEYLGDYMLDAKPKEISEIQRLNYEQNMSDAMAILHKLQTGLDVNVRFTGVRVFEYTP
Callithrix_jacchus          QLMEYLGDYMLDAKPKEISEIQRLNYEQNMSDAMAILHKLQTGLDVNVRFTGVRVFEYTP
Aotus_nancymaae             QLMEYLGDYMLDAKPKEISEIQR*NYEQNMSDAMAILHKLQTGLDVNVRFTGVRVFEYTP
Cebus_capucinus_imitator    QLMEYLGDYMLDAKPKEISEIQRLNYEQNMSDAMAILHKLQTGLDVNVRFTGVRVFEYTP
Saimiri_boliviensis         QLMEYLGDYMLDAKPKEISEIQRLNYEQNMSDAMAILHKLQTGLDVNVRFTGVRVFEYTP
Macaca_nemestrina           QLMEYLGDYMLDAKPKEISEIQRLNYEQNMSDAMAILHKLQTGLDVNVRFTGVRVFEYTP
Papio_anubis                QLMEYLGDYMLDAKPKEISEIQRLNYEQNMSDAMAILHKLQTGLDVNVRFTGVRVFEYTP
Chlorocebus_sabaeus         QLMEYLGDYMLDAKPKEISEIQRLNYEQNMSDAMAILHKLQTGLDVNVRFTGVRVFEYTP
Homo_sapiens                QLMEYLGDYMLDAKPKEISEIQRLNYEQNMSDAMAILHKLQTGLDVNVRFTGVRVFEYTP
Pongo_abelii                QLMEYLGDYMLDAKPKEISEIQRLNYEQNMSDAMAILHKLQTGLDVNVRFTGVRVFEYTP
Otolemur_garnetti           QLMEYLGDYMLEAKPKEISEIQRLNYEQNMSDAMAVLHKLQTGLDVNVKFTGVRVFEYTP
                            01234567890123456789012-34567890123456789012345678901234567 8
                          290       300         1          2         3         4

                            -------------------------------------------|
Brachyteles_arachnoides     ECIVFDLLDIPLYHGWLVDPQIDDIVKAVGNCSYNQLVEKIISCKQSDNSELVSEGFVAE
Callithrix_jacchus          ECIVFDLLDIPLYHGWLVDPQIDDIVKAVGNCSYNQLVEKIISCKQSDNSELLSEGFVAE
Aotus_nancymaae             ECIVFDLLDIPLYHGWLVDPQIDDIVKAVGNCSYNQLVEKIISCKQSDNSELVSEGFVAE
Cebus_capucinus_imitator    ECIVFDLLDIPLYHGWLVDPQIDDIVKAVGNCSYNQLVEKIISCKQSDNSELVSEGFVAE
Saimiri_boliviensis         ECIVFDLLDIPLYHGWLVDPQIDDIVKAVGNCSYNQLVEKIISCKQSDNSELVSEGFVAE
Macaca_nemestrina           ECIVFDLLDIPLYHGWLVDPQIDDIVKAVGNCSYNQLVEKIISCKQSDNSELVSEGFVAE
Papio_anubis                ECIVFDLLDIPLYHGWLVDPQIDDIVKAVGNCSYNQLVEKIISCKQSDNSELVSEGFVAE
Chlorocebus_sabaeus         ECIVFDLLDIPLYHGWLVDPQIDDIVKAVGNCSYNQLVEKIISCKQSDNSELVSEGFVAE
Homo_sapiens                ECIVFDLLDIPLYHGWLVDPQIDDIVKAVGNCSYNQLVEKIISCKQSDNSELVSEGFVAE
Pongo_abelii                ECIVFDLLDIPLYHGWLVDPQIDDIVKAVGNCSYNQLVEKIISCKQSDNSELVSEGFVAE
Otolemur_garnetti           ECIVFDLLDIPLYHGWLVDPQIDDIVKAVGNCSYNQLVEKIISCKQSDSSELVSEGFVAE
                            901234567890123456789012345678901234567890123456789012345678
                            350        6         7         8         9       400

Brachyteles_arachnoides     QFLNNTATQLTYHGLCELTSTVQEGELCVFFRNNHFSTMTKYKGQLYLLVTDQGFLTEEK
Callithrix_jacchus          QFLNNTATQLTYHGLCELTSTVQEGELCVFFRNNHFSTMTKYKGQLYLLVTDQGFLTEEK
Aotus_nancymaae             QFLNNTATQLTYHGLCELTSTVQEGELCVFFRNNHFSTMTKYKGQLYLLVTDQGFLTEEK
Cebus_capucinus_imitator    QFLNNTATQLTYHGLCELTSTVQEGELCVFFRNNHFSTMTKYKGQLYLLVTDQGFLTEEK
Saimiri_boliviensis         QFLNNTATQLTYHGLCELTSTVQEGELCVFFRNNHFSTMTKYKGQLYLLVTDQGFLTEEK
Macaca_nemestrina           QFLNNTATQLTYHGLCELTSTVQEGELCVFFRNNHFSTMTKYKGQLYLLVTDQGFLTEEK
Papio_anubis                QFLNNTATQLTYHGLCELTSTVQEGELCVFFRNNHFSTMTKYK.----------------
Chlorocebus_sabaeus         QFLNNTATQLTYHGLCELTSTVQEGELCVFFRNNHFSTMTKYKGQLYLLVTDQGFLTEEK
Homo_sapiens                QFLNNTATQLTYHGLCELTSTVQEGELCVFFRNNHFSTMTKYKGQLYLLVTDQGFLTEEK
Pongo_abelii                QFLNNTATQLTYHGLCELTSTVQEGELCVFFRNNHFSTMTKYKGQLYLLVTDQGFLTEEK
Otolemur_garnetti           QFLNNTATQLTYHGLCELTSTVQEGELCVFFRNNHFSTMTKYKGQLYLLVTDQGFLTEEK
                            901234567890123456789012345678901234567890012----------------
                            410        2         3         4         5
```

```
Brachyteles_arachnoides      IVWESLHNVDGDGNFCDSEFHLRPPSDPETVYKGQQDQIDQDYLMALSLQQEQQSQEINW
Callithrix_jacchus           VVWESLHNVDGDGNFCDSEFHLRPPSDPETVYKGQQDQIDQDYLMALSLQQEQQSQEINW
Aotus_nancymaae              VVWESLHNVDGDGNFCDSEFHLRPPSDPETVYKGQQDQIDQDYLMALSLQQEQQSQEINW
Cebus_capucinus_imitator     VVWESLHNVDGDGNFCDSEFHLRPPSDPETVYKGQQDQIDQDYLMALSLQQEQQSQEINW
Saimiri_boliviensis          VVWESLHNVDGDGNFCDSEFHLRPPSDPETVYKGQQDQIDQDYLMALSLQQEQQSQEINW
Macaca_nemestrina            VVWESLHNVDGDGNFCDSEFRLRPPSDPETVYKGQQDQIDQAGPASTSLS-------IKW
Papio_anubis                 ------------------------------------------------------------
Chlorocebus_sabaeus          VVWESLHNVDGDGNFCDSEFRLRPPSDPETVYKGQQDQIDQDYLMALSLQQEQQSQEINW
Homo_sapiens                 VVWESLHNVDGDGNFCDSEFHLRPPSDPETVYKGQQDQIDQDYLMALSLQQEQQSQEINW
Pongo_abelii                 VVWESLHNVDGDGNFCDSEFHLRPPSDPETVYKGQQDQIDQDYLMALSLQQEQQSQEINW
Otolemur_garnetti            VVWESLHNVDGDGNFCDSEFHLRPPSDPETVYKGQQDQIDQDYLMALSLQQEQQSQEI--


Brachyteles_arachnoides      EQIPEGISDLELAKKLQEEEDRRASQYYQEQEQ-AAAAAAASTQAQQGQPTQASPSSGRQ
Callithrix_jacchus           EQIPEGISDLELAKKLQEEEDRRASQYYQEQEQ-AAAAAAASTQAQQGQPTQASPSSGRQ
Aotus_nancymaae              EQIPEGISDLELAKKLQEEEDRRASQYYQEQEQAAAAAAAASTQAQQGQPTQASPSSGRQ
Cebus_capucinus_imitator     EQIPEGISDLELAKKLQEEEDRRASQYYQEQEQAAAAAAAASTQAPQGQPTQASPSSGRQ
Saimiri_boliviensis          EQIPEGISDLELAKKLQEEEDRRASQYYQEQEQ-AASAAAASTQAQQGQPTQASPSSGRQ
Macaca_nemestrina            KTIWE**T*TE-------------------------------------------------
Papio_anubis                 ------------------------------------------------------------
Chlorocebus_sabaeus          EQIPEGISDLELAKKLQEEEDRRASQYYQEQEQAAAAAAAASTQVQQGQPAQASPSSGRQ
Homo_sapiens                 EQIPEGISDLELAKKLQEEEDRRASQYYQEQEQ-AAAAAAASTQAQQGQPAQASPSSGRQ
Pongo_abelii                 EQIPEGISDLELAKKLQEEEDRRASQYYQEQEQAAAAAAAASTQAQQGQPAQASPSSGRQ
Otolemur_garnetti            ------------------------------------------------------------


Brachyteles_arachnoides      SGNSERKRKEPREKDKEKEKEKNSCVIL
Callithrix_jacchus           SGNSERKRKEPREKDKEKEKEKNSCVIL
Aotus_nancymaae              SGNSERKRKEPREKDKEKEKEKNSCVIL
Cebus_capucinus_imitator     SGNSERKRKEPREKDKEKEKEKNSCVIL
Saimiri_boliviensis          SGSSERKRKEPREKDKEKEKEKNSCVIL
Macaca_nemestrina            --------.EPREKDKEKEKEKNSCVIL
Papio_anubis                 ----------------------------
Chlorocebus_sabaeus          SGNSERKRKEPREKDKEKEKEKNSCVIL
Homo_sapiens                 SGNSERKRKEPREKDKEKEKEKNSCVIL
Pongo_abelii                 SGNSERKRKEPREKDKEKEKEKNSCVIL
Otolemur_garnetti            ----------------------------
```

MRPS14

```
                              | Signal Pept |
                                *    *
Brachyteles_arachnoides       MAGSTLGFVLRTFKQMVPSSASGQVRGYYVDWRMWRDVKRRKMAYEYADERLRVNSLRKN
Callithrix_jacchus            MAASVLGSVLRTFKQMVPSSASGQVRGYYVDWRMWRDVKRRKMAYEYADERLRVNSLRKN
Aotus_nancymaae               MAVSVLGSVLRMFKQMVPSSASGQVRGYYVDWRMWRDVKRRKMAYEYADERLRVNSLRKN
Cebus_capucinus_imitator      MAASVLGSVLRTFKQMVPSSASGQVRGYYVDWRMWRDVKRRKMAYEYADERLRVNSLRKN
Saimiri_boliviensis           MAASVLGSVLRAFKQMVPSSASGQVRGYYVDWRMWRDVKRRKMAYEYADERLRVNSLRKN
Macaca_nemestrina             MAAFMLGSLLRTFKQMVPSSASGQVRSHYVDWRMWRDVKRRKMAYEYADERLRINSLRKN
Papio_anubis                  MAAFMLGSLLRTFKQMVPSSASGQVRSHYVDWRMWRDVKRRKMAYEYADERLRINSLRKN
Chlorocebus_sabaeus           MAAFMLGSLLRMFKQMVPSSASGQVRSHYVDWRMWRDVKRRKMAYEYADERLRINSLRKN
Homo_sapiens                  MAAFMLGSLLRTFKQMVPSSASGQVRSHYVDWRMWRDVKRRKMAYEYADERLRINSLRKN
Pongo_abelii                  MAAFMLCSLLRKFKQMVPSSASGQVRSHYVDWRMWRDVKKRKMAYEYADERLRINSLRKN
Otolemur_garnetti             MAAYVLGSLLRTFRQVGPASASGQVRGYYVDWRMLRDVKRRKMAYEYADERLRINALRKN
                              123456789012345678901234567890123456789012345678901234567890
                                       1         2         3         4         5         6

                                 |-------------- Ribosomal protein S14/S29 ---------------
Brachyteles_arachnoides       TILPKLLQDVADEEIAAFPRDSCPVRIRNRCVMTSRPRGVKRRWRLSRIVFRHLADHGQL
Callithrix_jacchus            TILPKLLQDVADEEIAAFPRDSCPVRIRNRCVMTSRPRGVKRRWRLSRIVFRHLADHGQL
Aotus_nancymaae               TILPKLLQDVADEEIAAFPRDSCPVRIRNRCVLTSRPRGVKRRWRLSRIVFRHLADHGQL
Cebus_capucinus_imitator      TILPKLLQDVADEEIAAFPRDSCPVRIRNRCVMTSRPRGVKRRWRLSRIVFRHLADHGQL
Saimiri_boliviensis           TILPKLLQDVADEEIAAFPRDSCPVRIRNRCVMTSRPRGVKRRWRLSRIVFRHLADHGQL
Macaca_nemestrina             TXLPKILQDVADEEIAALPRDSCPVRNQESVCYDVRPRGVKRRWRLSRIVFRHLADN-QL
Papio_anubis                  TILPKILQDVADEEIAALPRDSCPVRIRNRCVMTSRPRGVKRRWRLSRIVFRHLADHGQL
Chlorocebus_sabaeus           TILPKILQDVADEEIAALPRDSCPVRIRNRCVMTSRPRGVKRRWRLSRIVFRHLADHGQL
Homo_sapiens                  TILPKILQDVADEEIAALPRDSCPVRIRNRCVMTSRPRGVKRRWRLSRIVFRHLADHGQL
Pongo_abelii                  TILPKILQDVADEEIAALPRDSCPVRIRNRCVMTSRPRGVKRRWRLSRIVFRHLADHGQL
Otolemur_garnetti             TILPKDIQEVADEEIAALPRDSCPVRIRNRCVMTSRPRGVKRRWRLSRIVFRHLADHGQL
                              12345678901234567890123456789012345678901234567890123456-789
                             61        7         8         9        100        1

                              ------|
Brachyteles_arachnoides       SGVQRAM
Callithrix_jacchus            SGVQRAM
Aotus_nancymaae               SGVQRAI
Cebus_capucinus_imitator      SGVQRA-
Saimiri_boliviensis           SGVQRAM
Macaca_nemestrina             SGIQRX-
Papio_anubis                  SGIQRX-
Chlorocebus_sabaeus           SGIQRX-
Homo_sapiens                  SGIQRAT
Pongo_abelii                  SGIQRA-
Otolemur_garnetti             SGVQRX-
                              012345
                            120
```

OAS2

```
                                      |-----------------------------------------------
                                                     *   *                   *
Brachyteles_arachnoides    MGNGESQLSSVPAQKLDLFIQKHLKPYKECQLLIDEMVTTICEVLQDPEQFHLVQGVAIG
Callithrix_jacchus         MGNGESQLPSVPAQKLDWFIQKRLKPYEECQRLIDRIVTTICEALQDPEQFPLVQGVAIG
Aotus_nancymaae            MGNRESQLSSVPAQKLDWFIQEHLKPYEECQTLIDRMVTTICEALQDPEQFPLVQGVAIG
Cebus_capucinus_imitator   MGNGESQLSSVPAQKLDSFIHEHLKPYEACQMLIDGLVTTICEALQDPKQFPLVQGVAIG
Saimiri_boliviensis        MGNGESQLSSVPAQKLDSFIHKHLKPYEECQKPINWMVTTICAALQDPVRFPLVQGVAIG
Macaca_nemestrina          MGNGGSQLSSVPAQKLGGFIQEYLKPYEECQTLIDDMVNTICDVLQAPNQFPLVQGVAIG
Chlorocebus_sabaeus        MGNGGSQLSSVPAQKLGWFIQEYLKPYEECQTLIDDMVNTICDVLQAPKQFPLVQGVAIG
Homo_sapiens               MGNGESQLSSVPAQKLGWFIQEYLKPYEECQTLIDEMVNTICDVLQEPEQFPLVQGVAIG
Pongo_abelii               MGNGESQLSSVPAQKLGWFIQEYLKPYKECQTLIDETVNTICDVLQEPEQFPLVQGVAIG
                           123456789012345678901234567890123456789012345678901234567890
                          0         1         2         3         4         5         6

                           ---- 2-5OAS/ClassI-CCAase, nucleotidyltransferase domain ----
                                                                                *
Brachyteles_arachnoides    GSYGRETVLRGNSDGTLVLFFSDLKQFQDQKRSQHDSLVKTGHKLNLCLFTKNMKDNFEI
Callithrix_jacchus         GSYGRETFLRGNCDGTLVLFFGDLKQFQDQKGSQRDKLVKTGHKLNSCQFVEWIKDNFEI
Aotus_nancymaae            GSYGRETVLRGNSDGTLVLFFGDLKQFQDQKRSQHDNLVKTGHKLNLYLFTEWMKDSVEI
Cebus_capucinus_imitator   GSYGRETVLRGNSDGTLVLFFGDLKQFQDQKRSQRDNLVKTGHKLNLCLFTEWMKDRFEI
Saimiri_boliviensis        GSYGRETVLRGDSDGTLVLFFSDLKQFQDQKRSQRDNLIKIGHQLNVASFTELMKDNFEI
Macaca_nemestrina          GSYGRKTVLRGNSDGTLVLFFSNLKQFQDQKKSQHDILDKTGHKLEFCLYTKWMKDSFEI
Chlorocebus_sabaeus        GSYGRKTVLRGNSDGTLVLFFSDLKQFQDQKKSQHDILDKTGHKLEFCLYEKWMKDSFEI
Homo_sapiens               GSYGRKTVLRGNSDGTLVLFFSDLKQFQDQKRSQRDILDKTGDKLKFCLFTKWLKNNFEI
Pongo_abelii               GSYGRKTVLRGNSDGTLVLFFSDLKQFQDQKRSQHDILDKTGDKLKFYLFTKLMNNNFEI
                           123456789012345678901234567890123456789012345678901234567890
                          61        7         8         9        100        1         2

                           -----------------------------------|-----------------------
                              *         *                 *              *
Brachyteles_arachnoides    QMSDDGFTIQVFTSNQRISFEVLAAFNALGLDDNPSPWIYRELKRSMDKTNARPGEFAVC
Callithrix_jacchus         QMSCDGFTIQVFTKDQRISFEVLAAFNALGLNDSPSPWIYRELKRSLDKTNARPGEFAVC
Aotus_nancymaae            QMSHDGFSIQVFTKDQRISFEVLAAFNALGLNDNPSPWIYRELKRSLDKTNAHPGEFAVC
Cebus_capucinus_imitator   QMSDDGFTIQVFTKDQRISFEVLAAFNALGLNDNPSPWIYRDLKRSLDKTNARPGEFAVC
Saimiri_boliviensis        QMSHDGFTIQVFIKDQRISFEVLAAFNALGLNDNPSPWIYRELKRSLDKTNARPGEFAVC
Macaca_nemestrina          QKSHDGFTIQLFTKNQRVSFEVLAAFNALSLNDNPSPWIYRELKRSLDKTSASPGEFAVC
Chlorocebus_sabaeus        QKSHDGFTIQLFTKNQRVSFEVLAAFNALSLNDNPSPWIYRELKRSLDKTSASPGEFAVC
Homo_sapiens               QKSLDGFTIQVFTKNQRISFEVLAAFNALSLNDNPSPWIYRELKRSLDKTNASPGEFAVC
Pongo_abelii               QKSHDGFTIQVFTRNQRISFEVLAAFNALSLNDNPSPWIYRELKRSLDKTNASPGEFAVC
                           123456789012345678901234567890123456789012345678901234567890
                         121        3         4         5         6         7         8

                           -- 2'-5'-oligoadenylate synthetase 1, domain 2/C-terminal --
                                        *
Brachyteles_arachnoides    FTELQQKFFNNCPSKLKDLILLIKHWHQQCQKKMSGLPLLSPYALELLTVYAWEQGCRKD
Callithrix_jacchus         FTELQQKFFNNCPRKLKDLILLIKHWHQQCQEKMNGLPLLSPYALELLTVYAWEQGCRQD
Aotus_nancymaae            FTELQQRFFNNCPRKLKDLILLIKHWHQQCQKKMNGXPLLSPYALELLTVYAWEQGCRKD
Cebus_capucinus_imitator   FTELQQKFFNNRPRKLKDLILLIKHWHQQCQKKMNGLPLLSPYALELLTVYAWEQGCRKD
Saimiri_boliviensis        FTELQQKFFNNCPRKLKDLILLIKHWHQQCQKKMNGLPLPSPYALELLTVYAWEQGCRKD
Macaca_nemestrina          FTELQQKFFDNRPRKLKDLILLIKHWHQQCEKKMKDLPLLSPYALELLTVYAWEQGCRRD
Chlorocebus_sabaeus        FTELQQKFFDNRPRKLKDLILLIKHWHQQCQKKMKDLPLLSPYALELLTVYAWEQGCRKD
Homo_sapiens               FTELQQKFFDNRPGKLKDLILLIKHWHQQCQKKIKDLPSLSPYALELLTVYAWEQGCRKD
Pongo_abelii               FTELQQKFFDNRPRKLKDLILLIKHWHLQCQKKNKDLPSLSPYALELLTVYAWEQGCRKD
                           123456789012345678901234567890123456789012345678901234567890
                         181        9        200        1         2         3         4

                           ------------------------------------------------------------
Brachyteles_arachnoides    NFDIAEGVRTVLGLIKCHEQLCIYWMVNYNFEDETVRNVLLHQLQSERPVILDPTDPTNN
Callithrix_jacchus         DFDIAEGVRTVLGLIKCQEQLCIYWMVNYNFEDETVRNVLLHQFQSERPVILDPTDPTNN
Aotus_nancymaae            DFDIAEGVRTVLGLIKCEEQLCIYWMVNYNFENETVRNVLLHQLQSERPVILDPTDPTNN
Cebus_capucinus_imitator   NFDIAEGVRTVLGLIKCQEQLCIYWMVNYNFEDETVRNVLLHQLQSVRPVILDPTDPTNN
Saimiri_boliviensis        DFDIAEGVRTVLGLIKCQEQLCIYWMVNYNFEDETVRNVLLHQLQSKRPVILDPTDPTNN
Macaca_nemestrina          NFDIAEGVRTILELIKCHEQLCVYWMVNYNFEDETIRNILLPQLQSVRPVILDPTDPTNN
Chlorocebus_sabaeus        NFGIAEGVRTILELIKCHEQLCVYWTVNYNFEDETIRNILLPQLQSARPVILDPTDPTNN
Homo_sapiens               NFDIAEGVRTVLELIKCQEKLCIYWMVNYNFEDETIRNILLHQLQSARPVILDPVDPTNN
Pongo_abelii               NFDIAEGVRTVLELIKRQEQLCIYWMVNYNFEDETIRNILLHQLQSARPVILDPADPTNN
                           123456789012345678901234567890123456789012345678901234567890
                         241        5         6         7         8         9        300
```

```
                             ----------------------------------|
                              *  *
Brachyteles_arachnoides      VRGDYRCWQRLKQEAQTWLTSPNLDNELPAPSWNVLPAPLFTTPGHRLDKFIKDFLQPDK
Callithrix_jacchus           VSGDYRCWQRLKQEAQTWLTSPNLDNELPAPSWNVLPAPLFTTLGHHLDKFIKDFLQPDK
Aotus_nancymaae              VSGDNRCWQRLKQEAQTWLTSPSLDNELPAPSWNVLPAPLFTTPGHRLDKFIKDFLQPDK
Cebus_capucinus_imitator     VSGDNRCWQRLKQEAQTWLTSPQLDNELPAPSWNVLPAPLFTTPGHCLDKFIKDFLQPDK
Saimiri_boliviensis          VSGDNRCWQRLKQEAQTWLTSPNLDNELPAPSWNVLPAPLFTTPGHCLDKFIKDFLQPDK
Macaca_nemestrina            VSGDKRCWQWLKKEAQTWLTSPNLDNELPAPSWNVLPAPLFTTPGHLLDKFIKEFLQPNK
Chlorocebus_sabaeus          VSGDKRCWQWLKKEAQTWLTSPNLDNELPAPSWNVLPAPLFTTPGHLLDKFIKEFLQPNK
Homo_sapiens                 VSGDKICWQWLKKEAQTWLTSPNLDNELPAPSWNVLPAPLFTTPGHLLDKFIKEFLQPNK
Pongo_abelii                 VSGDKICWQRLKKEAQTWLTSPNLDNELPAPSWNVLPAPLFTTPGHLLDKFIKEFLQPNK
                             123456789012345678901234567890123456789012345678901234567890
                           301         1         2         3         4         5         6

                              |--- 2-5OAS/ClassI-CCAase, nucleotidyltransferase domain --
Brachyteles_arachnoides      YFLEQIDSAVNIICTFLKENCFRQSTAKIQTVRGGSTAKGTALKTGSDADLVVFHNSLKS
Callithrix_jacchus           YFLEQVDSAVNIICKFLEENCFRRSTAKIQTVQGGSTAKGTALKTGSDSDLVVFHNSLKS
Aotus_nancymaae              YFLEQVDIAINIICKFLEENCFRQSTAKIQTVQGGSTAKGTALKTGSDADLVVFHNSLKS
Cebus_capucinus_imitator     YFLEQVDSAVNIICKFLEENCFRRSIAKIQTVQGGSTAKGTALKTGSDGNLVVFHNSLKS
Saimiri_boliviensis          YFLEQVNGAVNIICKFLEENCFPQSTAKIKTVQGGSTAKGTALKTGSDADLVVFHNSLRS
Macaca_nemestrina            FFLEQIDSAVDIICTFLKENCFRQSTAKIQIVQGGSTAKGTALKTGSDANLVVFHNSLKS
Chlorocebus_sabaeus          FFLEQIDSAVDMICTFLKENCFRQSTAKIQIVQGGSTAKGTALKTGSDVDLVVFHNSLKS
Homo_sapiens                 CFLEQIDSAVNIIRTFLKENCFRQSTAKIQIVRGGSTAKGTALKTGSDADLVVFHNSLKS
Pongo_abelii                 CFLEQIDSAVNIIRTFLKENCFRQSTAKIQIVRGGSTAKGTALKTGSDADLVVFHNSLKS
                             123456789012345678901234567890123456789012345678901234567890
                           361         7         8         9        400        1         2

                             ------------------------------------------------------------
                              **
Brachyteles_arachnoides      YICQKNERHKIIKEIHKQLEAFQREKKEELEVRFEISKWKAPRVLSFSLKSKVLNESVNF
Callithrix_jacchus           YTFQKNEQHKIIKEIHQQLEAFQQEKKEELEVRFEISRWKAPRVLSFSLKSKVLNESVNF
Aotus_nancymaae              YTSPKNERHKIFKEIHQQLEAFQRQKKEELEVRFEISKWKAPRVLSFSLKSKVLNQSVNF
Cebus_capucinus_imitator     YTSQKNERHKIIKEIHQQLEAFQQEKKEELEVRFEISKWEAPRVLSFSLKSKVLNESVNF
Saimiri_boliviensis          YTSQKYTGHNIMKEIHQQLEAFQREMEEEIEVRFEISKWKAPRVLSFSLKSKVLNESVSF
Macaca_nemestrina            YTSQKNERYRIIKEIHEQLETFWREKKEELEVSFEPPMWKAPRVLSFSLKSKVLNESVSF
Chlorocebus_sabaeus          YTSQKNERYRIIKEIQEQLETFWREKKEELEVSFELPMWKAPRVLSFSLKSKVLNESVSF
Homo_sapiens                 YTSQKNERHKIVKEIHEQLKAFWREKEEELEVSFEPPKWKAPRVLSFSLKSKVLNESVSF
Pongo_abelii                 YTSQKNERHKIVKEIHEQLEAFWRENKEELEVSFEPPKWKAPRVLSFSLKSKVLNESVSF
                             123456789012345678901234567890123456789012345678901234567890
                           421         3         4         5         6         7         8

                             -----------------|------------------------------------------
Brachyteles_arachnoides      DVLPAFNALGQLSSGSTPSPEVYAGLIDLYKSSDLPGGEFSTCFTVLQRNFIRTQPTKLK
Callithrix_jacchus           DVLPAFNALGQLSSGSTSSPEVYAGLIDLYKSSDLPGGEFSTCFTALQRNFISSQHTKLK
Aotus_nancymaae              DVLPAFNALGQLSSGSTSSPEVYAGLIDLYKSSDLPGGEFSTCFTALQRNFIRSQDTKLK
Cebus_capucinus_imitator     DVLPAFNALGQLSSGSTSSPEVYAGLIDLYKSSDLPGGEFSTCFTALQRNFIHSRHTKLK
Saimiri_boliviensis          NVLPAFNALGQLSSGSTSSPEVYAELIDLYKSLDLPGGEFSTCFTVLQRNFIHSRPTKLK
Macaca_nemestrina            DVLPAFNALGQLSSGSTPSPEVYAGLLDLYKSSDFPGGEFSTCFTVLQRNFICSRPTKLK
Chlorocebus_sabaeus          DVLPAFNALGQLSSGSTPTPEVYAGLLDLYKSSDFPGGEFSTCFTVLQRDFIRSRPTKLK
Homo_sapiens                 DVLPAFNALGQLSSGSTPSPEVYAGLIDLYKSSDLPGGEFSTCFTVLQRNFIRSRPTKLK
Pongo_abelii                 DVLPAFNALGQRSSGSTPSPEVYAGLIDLYKSSDLPGGEFSTCFTVLQRNFIRSRPTKLK
                             123456789012345678901234567890123456789012345678901234567890
                           481         9        500        1         2         3         4

                             -- 2'-5'-oligoadenylate synthetase 1, domain 2/C-terminal --
Brachyteles_arachnoides      DLIRLVKHWYKECERKLKPKGSLPPKYALELLTVYAWEQGSGVLDFDTAEGFRTVLGLVT
Callithrix_jacchus           DLIRLVKHWYKECERKLKPKGSLPPKYALELLTVYAWEQGSGQPDFDTAEGFRTVLELVT
Aotus_nancymaae              DLIRLVKHWYKECERKLKPKGSLPPKYALELLTVYAWEQGSGVPDFDTAEGFRTVLELVT
Cebus_capucinus_imitator     DLIRLVKHWYQECERKLKPKGSLPPKYALELLTVYAWEQGSGMPDFDTAEGFRTVLELVT
Saimiri_boliviensis          DLIRLVKHWYKECERKLKPKGSLPPKYALELLTVYAWEQGSGLPDFDTAEGFRTVLELVT
Macaca_nemestrina            DLIRLVKHWYKECKRKLKPKGSLPPKYALELLTVYAWEQGSGAPDFDTAEGFRTVLELVT
Chlorocebus_sabaeus          DLIRLVKHWYKECERKLKPKGSLPPKYALELLTVYAWEQGSGAPDFDTAEGFRTVLELVT
Homo_sapiens                 DLIRLVKHWYKECERKLKPKGSLPPKYALELLTIYAWEQGSGVPDFDTAEGFRTVLELVT
Pongo_abelii                 DLIRLVKHWYKECERKLKPKGSLPPKYALELLTIYAWEQGSGVPDFDTAEGFRTVLELVT
                             123456789012345678901234567890123456789012345678901234567890
                           541         5         6         7         8         9        600
```

```
                           -- 2'-5'-oligoadenylate synthetase 1, domain 2/C-terminal --
Brachyteles_arachnoides    QYQQLCIFWKVNYNFEDETVRKFLLSQLQKTRPVILDPAEPTGDVGGGDRWCWHLLAKEA
Callithrix_jacchus         QYQQLCIFWKVNYNFEDETVRKFLLSQLQKTRPVILDPAEPTGDVGGGDRWCWHLLAKEA
Aotus_nancymaae            QYQQLCIFWKINYNFEDETVRKFLLSQLQKTRPVILDPAEPTGDVGGGDRWCWHLLAKEA
Cebus_capucinus_imitator   QYQQLCIFWKVNYNFEDETVRKFLLSQLQKARPVILDPAEPTGDVGGGDRWCWHLLAKEA
Saimiri_boliviensis        QYQQLCIFWKVNYNFEDETVRKFLLSQLQKTRPVILDPAEPTGDVGGGDRWCWHLLAKEA
Macaca_nemestrina          QYRQLCIFWKVNYNFEDETMRKFLLSQLQKTRPVILDPAEPTGDVGGGDRWCWHLLAKEA
Chlorocebus_sabaeus        QYRQLCIFWKVNYNFEDETMRKFLLSQLQKTRPVILDPAEPTGDVGGGDRWCWHLLAKEA
Homo_sapiens               QYQQLCIFWKVNYNFEDETVRKFLLSQLQKTRPVILDPAEPTGDVGGGDRWCWHLLAKEA
Pongo_abelii               QYQQLCIFWKVNYNFEDETIRKFLLSQLQKTRPVILDPAEPTGDLGGGDRWCWHLLAKEA
                           123456789012345678901234567890123456789012345678901234567890
                         601         1         2         3         4         5         6

                           -----------------------|
Brachyteles_arachnoides    KEWLCSLCFKDGSGNPISPWKVPVKVI
Callithrix_jacchus         KEWFCSLCFKDGSGNPIPPWKVPVKVI
Aotus_nancymaae            KEWLCSLCFKDGSGNPIPPWKVPVKVI
Cebus_capucinus_imitator   KEWLYSLCFKDGSGNPIAPWKVPVKVI
Saimiri_boliviensis        KEWLYSLCFKGGSGNPISPWKVPVKVI
Macaca_nemestrina          KEWSYSLCFKDETGNPISPWKVPVKVI
Chlorocebus_sabaeus        KEWLYSLCFKDGTGNPISPWKVAVKVI
Homo_sapiens               KEWLSSPCFKDGTGNPIPPWKVPVKVI
Pongo_abelii               KEWLSSLCFKDGTGNPIPPWKVPX---
                           123456789012345678901234
                         661         7         8
```

PPEF1

```
                                 |--------- IQ motif ---------|
                                                   *
Brachyteles_arachnoides     MGCNTSSTAVKSSQTSLRSVLIIQNWYRGYRARLKARKQYALTIFQSIEYADEQDQIQLC
Callithrix_jacchus          MGCNTSSTRIKNSQISLRAVLIIQNWYRGYRARLKARRHFALTIFHSLEYADEQDQIQLC
Aotus_nancymaae             MGCNTSSTRIENSQTSLRAVLIIQNWYRGYRARLKARKQYALTIFQSIEYADEQDQIQLC
Cebus_capucinus_imitator    MGCNTSSTRIKNSQTSLRAVLIIQNWFRGYRARLKARQQYALTIFQSIEYADEQDQIQLC
Saimiri_boliviensis         MGCNTSSTRIKNSQTSLRAVLIIQNWYRGYKARLKARQQYALTIFQSIEYADEQDQIQLC
Macaca_nemestrina           MGCSSSSTKTRKSETSLKAALIIQNWYRGYKARLKARQHYALTIFQSIEYADEQGQMQLS
Chlorocebus_sabaeus         MGCSSSSTKTRKSETSLKAALIIQNWYRGYKARLKARQHYALTIFQSIEYADEQGQMQLS
Homo_sapiens                MGCSSSSTKTRRSDTSLRAALIIQNWYRGYKARLKARQHYALTIFQSIEYADEQGQMQLS
Pongo_abelii                MGCSSSSTKTRRSETSLRAALIIQNWYRGYKARLKARQHYALTIFQSIEYADEQGQMQLS
Otolemur_garnetti           ----------------LRAVLIIQNWYRGYRARLRARQHYALTIFQSIEYADEQGQMQLS
                                            12345678901234567890123456789012345678901234
                                                     1         2         3         4


                                                 *  *
Brachyteles_arachnoides     NFFSFMLENYTHIRKEEIEL-YQLGEIKLDMWDKQDYVDL-DVPDSYQGPRLQFPLTCAD
Callithrix_jacchus          NFFSFMLENYTHIRKEEIGL-NQLADSKLDMRDKQDYVDL-DVPDSYQGPRLQFPLTCAD
Aotus_nancymaae             NFFSFMLENYTRIRKEEIGL-NQLAENELDMRDKQDYVDLIDVPDSYKGPRLQFPLTCAD
Cebus_capucinus_imitator    NFFTFMLENYKHIRKEEIEL-SQLTESKLDMMDKQDYVDLIDVPDSYQGPRLQFPLTCAD
Saimiri_boliviensis         NFFSFMLENYTHIRKEEIEL-SQLTESKQDMRDKQDYIHL-DVPDSYQGPRLQFPLTCAD
Macaca_nemestrina           TFFSFMFENYAHVHKEELELRNQSLESEQDMRDRHDYVDLIDVPDSYNGPRLQFPLTCTD
Chlorocebus_sabaeus         TFFSFMLEHYAHVHKEELELRNQSLESEQDMQDRHDYVDLIDVPDSYNGPRLQFPLTCTD
Homo_sapiens                TFFSFMLENYTHIHKEELEL-NQSLESEQDMRDRWDYVDS-DVPDSYNGPRLQFPLTCTD
Pongo_abelii                TFFSFMLENYTHIHKEELELRNQSLESQQDMRDRRDYVDLIDVPDSYNGPRLQFPLTCTD
Otolemur_garnetti           NFFSFMLDNYTNMGEDDPELVHRFIASQQEIKSRQEYVELIDVPDSYLGPRLQFPLTFTD
                            56789012345678901234-5678901234567890123-4567890123456789012
                           45    5         6          7         8          9       100


                            |--- Serine/threonine-specific protein phosphatase ---------
                                      *
Brachyteles_arachnoides     IDLLLEAFKGDQILHARYVLEVLFETKKLLKQMPNFTQVQTSPSKEVTVCGDLHGKLDDL
Callithrix_jacchus          IDLLLEAFKGKQILHAHYVLEVLFETMKLLKQMPNFTQVQTSPSKEITVCGDLHGNLDDL
Aotus_nancymaae             IDLLLEAFKRKQILHARYVLEVLFETKKLLKQMPNFTQVQTSPSKEVTVCGDLHGKLDDL
Cebus_capucinus_imitator    IDLLLEAFKGKQILHARYVLEVLFETKKLLKQMPNFTQIQTSPSKEVTVCGDLHGKLDDL
Saimiri_boliviensis         IDLLLEAFKGKQILHARYVLEVLFETQKLLKQMPNFTQIQTSPSKEVTVCGDLHGKLDDL
Macaca_nemestrina           IDLLLEAFKEQQILHAHYVLEVLFETKKVLKQMPNFTHVQTSPSKELTICGDLHGKLDDL
Chlorocebus_sabaeus         IDLLLEAFKEQQILHAHYVLEVLFETKKVLKQMPNFTHVQTSPSKELTICGDLHGKLDDL
Homo_sapiens                IDLLLEAFKEQQILHAHYVLEVLFETKKVLKQMPNFTHIQTSPSKEVTICGDLHGKLDDL
Pongo_abelii                IDLLLEAFKEQQILHAHYVLEVLFETKKVLKQMPNFTHIQTSPSKEVTICGDLHGKLDDL
Otolemur_garnetti           IDLLLEAFKQQQILHAHYVLEVLFETKKILKQMPNFTHIKTSPSREITICGDLHGKLDDL
                            345678901234567890123456789012345678901234567890123456789012
                          103       1         2         3         4         5         6


                            ------------------------------------------------------------
                                       *                                  *
Brachyteles_arachnoides     FLIFYKNGLPSRSNPYVFNGDFVDRGSNSTEVLMILCVSFLVYPNDVHLNRGNHEDYMMN
Callithrix_jacchus          FLIFYKNGLPSGSNPYVFNGDFVDRGRNSIEVLMILCVSFLVYPNDLHLNRGNHEDYMMN
Aotus_nancymaae             FLIFYKNGLPSGSNPYVFNGDFVDRGRNSIEVLMILCVSFLVYPNDLHLNRGNHEDYMMN
Cebus_capucinus_imitator    FLIFYKNGLPSGSNPYVFNGDFVDRGRNSMEVLMILCVSFLVYPNDLHLNRGNHEDYMMN
Saimiri_boliviensis         FLIFYKNGLPSGSNPYVFNGDFVDRGRNSIEVLMILCVSFLVYPNDLHLNRGNHEDYMMN
Macaca_nemestrina           FMIFYKNGLPSESNPYVFNGDFVDRGKNSIEILIILCVSFLVYPNDLHLNRGNHEDFMMN
Chlorocebus_sabaeus         FMIFYKNGLPSESNPYVFNGDFVDRGKNSIEILIILCVSFLVYPNDLHLNRGNHEDFMMN
Homo_sapiens                FLIFYKNGLPSERNPYVFNGDFVDRGKNSIEILMILCVSFLVYPNDLHLNRGNHEDFMMN
Pongo_abelii                FLIFYKNGLPSERNPYVFNGDFVDRGRNSIEILMILCVSFLVYPNDLHLNRGNHEDFMMN
Otolemur_garnetti           FLIFYKNGLPSENNPYVFNGDFVDRGKNSMEILMILLVSFLVYPNDVHLNRGNHEDFMMN
                            345678901234567890123456789012345678901234567890123456789012
                          163       7         8         9        200        1         2
```

```
                              ------------------------------------------------------------
Brachyteles_arachnoides       LRYGFTKEILCKY-KIHGKKILQSLEELYTWLPIGTIIDNEILVVHGGISETTDLNLLHR
Callithrix_jacchus            LRYGFTKEILHKY-KIHGKKILQTLEEFYTWLPIGTIIDNEILVVHGGISETTDLNLLHR
Aotus_nancymaae               LRYGFTKEILHKY-KIHGKKILQTLEEFYTWLPIGTIIDNEILVVHGGISETTDLNLLHR
Cebus_capucinus_imitator      LRYQKNKTYSISYLQIHGKKILQTLEELYTWLPIGTIIDNEILVVHGGISETTDLNLLHR
Saimiri_boliviensis           LRYGFTKEILHKY-KVHGKKILQTLEELYTWLPIGTIIDNEILVVHGGISETTDLNLLHR
Macaca_nemestrina             LRYGFTKEILHKY-KLHGKRILQILEELYAWLPIGTIVDNEILVIHGGISETTDLNLLHR
Chlorocebus_sabaeus           LRYGFTKEILHKY-KLHGKRILQILEELYAWLPIGTIVDNEILVIHGGISETTDLNLLHC
Homo_sapiens                  LRYGFTKEILHKY-KLHGKRILQILEEFYAWLPIGTIVDNEILVIHGGISETTDLNLLHR
Pongo_abelii                  LRYGFTKEILHKY-KLHGKRILQILEELYAWLPIGTIVDNEILVIHGGISETTDLNLLHR
Otolemur_garnetti             LRYGFTKEILHKY-KLHGKKILEILEELYTWLPIGTIIDNEILVTHGGISESTDLNVLHR
                              3456789012345-67890123456789012345678901234567890123456789012345678901
                            223       3           4         5         6         7         8

                              ------------------------------------------------------------
                              *                        *
Brachyteles_arachnoides       IERNKMKSVLMPPREINKDHNTNSQHSKVGVTISAHGRIKTSGFPSEHLPKQEWEQVIDI
Callithrix_jacchus            VERNKMKSVLMPPREINKDHSSDSKHGNVGVTIGAHGRIKASGCPSEHLPKHEWEQVIDI
Aotus_nancymaae               VERNKMKSVLMPPREIYKDYNTDSKHSKVDVTISAHGRIKTKGFLSEHLPKHEREQVIDI
Cebus_capucinus_imitator      LERNKMKSVLMPPKEINKDYNTDSKHSKLGVTISAHGRIKASGLSSEHLPKHEWEQVIDI
Saimiri_boliviensis           VERNKMKSVLMPPKEINKDCNNDSKPSKVGVTISAHGRIKASGCPSEHLPKHEWEQVVDI
Macaca_nemestrina             VERNKMKSVLIPPTETNRDNDTDSKHNKVGVTFNAHGRIKTNGSPIEHLPKHEWEQIIDI
Chlorocebus_sabaeus           IERNKMKSVLIPPMETDRDYDTDSKHNKVGVTFNAHGRIKTNRSSIEHLPKHEWEQIIDI
Homo_sapiens                  VERNKMKSVLIPPTETNRDHDTDSKHNKVGVTFNAHGRIKTNGSPTEHLTEHEWEQIIDI
Pongo_abelii                  VERNKMKSVLIPPTETNRDHDTDWKHNKVGVTFNAHGRIKTNGSPIEHLTKHEWEQIIDI
Otolemur_garnetti             LERNKMKSVLIPPMDVSRDQAVEFEQSRMGISVSASEIFK-QGETSEHLSRHEWEQVIDI
                              2345678901234567890123456789012345678901-2345678901234567890
                            282       9        300        1         2         3         4

                              ------------------------------------------------------------
                                                                            *
Brachyteles_arachnoides       LWSDPRGKKGCFPNTGRGGGCYFGPDITSKILNKFQLKMIIRSHQCKPEGYEICHDGKVV
Callithrix_jacchus            LWSDPRGKKGCFPNTGRGGGCYFGPDITSKILNKFQLKMIIRSHECKPEGYEICHGGKVV
Aotus_nancymaae               LWSDPRGKKGCFPNTGRGGGCYFGPDITSKILNKFQLKMIIRSHECKPEGYEICHGGKVV
Cebus_capucinus_imitator      LWSDPRGKKGCFPNTGRGGGCYFGPDITSKILNKFQLKMIIRSHECKPEGYEICHGGKVV
Saimiri_boliviensis           LWSDPRGKKGCFPNTGRGGGCYFGPDITSKILNKFQLKMIIRSHECKPEGYEICHGGKVV
Macaca_nemestrina             LWSDPRGKNGCFPNTGRGGGCYFGPDVTSKILNKYQLKMLIRSHECKPEGYEICHDGKVV
Chlorocebus_sabaeus           LWSDPRGKNGCFPNTGRGGGCYFGPDVTSKILNKYQLKMLIRSHECKPEGYEICHDGKVV
Homo_sapiens                  LWSDPRGKNGCFPNTCRGGGCYFGPDVTSKILNKYQLKMLIRSHECKPEGYEICHDGKVV
Pongo_abelii                  LWSDPRGKNGCFPNTGRGGGCYFGPDVTSKILNKYQLKMLIRSHECKPEGYEICHDGKVV
Otolemur_garnetti             LWSDPKGRKGCYPNTCRGGGCYFGPDVTSKILSKFQLKMLIRSHECKPEGYEICHDGKVI
                              123456789012345678901234567890123456789012345678901234567890
                            341         5         6         7         8         9       400

                              ------------------------------------|
                                                          *
Brachyteles_arachnoides       TIFSASNYYEEDSNRGAYVKLCSGTNPQFFQYQVTDATCFHPLHQRVSTIENSAIKILRE
Callithrix_jacchus            TIFSASNYYEEDSNRGAYVKLCSGKNPLFFQYQVTDATCFHTLHQRVSTVENSAIKILRE
Aotus_nancymaae               TIFSASNYYEEDSNRGAYVKLCSGTNPRFFQYQVTDATCFHPFHQRVSTMENSAIKILKE
Cebus_capucinus_imitator      TIFSASNYYEEDSNRGAYVKLCSGTNPRFFQYQVTDATCFQPLHQRVSTMENSAIKILRE
Saimiri_boliviensis           TIFSASNYYEEDSNRGAYVKLCSGANPRFFQYQVTDATCFQPLHQRVSTMENSAIKILRE
Macaca_nemestrina             TIFSASNYYEEGSNRGAYIKLCSGTAPRFFQYQVTKATCFQPLRQRVYTMESSAIKILKE
Chlorocebus_sabaeus           TIFSASNYYEEGSNRGAYIKLCSGTAPRFFQYQVTKATCFQPLRQRVYTMESSAIKILKE
Homo_sapiens                  TIFSASNYYEEGSNRGAYIKLCSGTTPRFFQYQVTKATCFQPLRQRVDTMENSAIKILRE
Pongo_abelii                  TIFSASNYYEEGSNRGAYIKLCSGTTPRFFQYQVTKATCFQPLRQRVHTMENSAIKILRE
Otolemur_garnetti             TVFSASNYYEEGSNRGAYIRLCSGMNPRFFQYQVTKATCLKPLHQRVNIMEISAIRALKE
                              123456789012345678901234567890123456789012345678901234567890
                            401         1         2         3         4         5         6
```

```
                             EF-hand calcium-binding domain 1
                           |-------------------------------------|
                             *                               *
Brachyteles_arachnoides     RMIARKTDLIRAFQHQDRRKSGKLPVSQWAFCMESVLGLNLPWRSLSSNLVIIDQNGNVE
Callithrix_jacchus          RMISRKTDLICAFQHQDRRKSGKLPVSQWAFCMESILGLNLPWRSLSSNLVIIDQNGNVE
Aotus_nancymaae             RMISRKTDLIRAFLYQDRRKSGKLPVSKWAFCMETILGLNLPWRSLSSNLVIIDQNGNVD
Cebus_capucinus_imitator    RMISRKSDLIHAFQHQDRRKSGKLPVSQWAFCMESILGLNLPWRSLSSNLVIIDQNGNVE
Saimiri_boliviensis         RMISRKTDLIHAFQHQDRRKSGKLPVSQWAFCMESILGLHLPWRSLSSNLATIDENGNIE
Macaca_nemestrina           RVISRKSDLIRAFQLQDHRKSGKLSLSQWAFCMENILGLNLPWRSLSSNLVNIDKNGNVE
Chlorocebus_sabaeus         RVISRKSDLTRAFQLQDHRKSGKLSLSQWAFCMENILGLNLPWRSLSSNLVNIDKNGNVE
Homo_sapiens                RVISRKSDLTRAFQLQDHRKSGKLSVSQWAFCMENILGLNLPWRSLSSNLVNIDQNGNVE
Pongo_abelii                RVISRKSDLIRAFQLQDHRKSGKLSVSQWAFCMENILGLNLPWRSLSSNLVNIDQNGNVE
Otolemur_garnetti           RMISRKTDLTRAFQLQDPRKSGKVSLGQWALSMENILGLQLPWRTLSSHLVTADKDGNIM
                            123456789012345678901234567890123456789012345678901234567890
                          461       7         8         9         500       1         2


                                                   EF-hand calcium-binding domain 2
                                                 |-----------------------------------
                                                                                   *
Brachyteles_arachnoides     YMSSFRNIHIEKPIKEANSALVETLYRYRSDLEIIFNAIDTDHSGLISMEEFRAMWKLFI
Callithrix_jacchus          YMSSFQNIQIEKPIQEANSALVETLYRYRSDLEIIFNAIDTDHSGLISMEEFRTMWKLFR
Aotus_nancymaae             YMSSFRNIHIEKPVKEANSALVETLCRYRSDLEIIFNAIDTDHSGLISMEEFRAMWKLFS
Cebus_capucinus_imitator    YMSSFRNIHIEKPIKEANSALVETLYRYRSDLEIIFNAIDTDHSGLISMEEFHAMWKLFS
Saimiri_boliviensis         YMSSFRNIHIEKPIKEANSALVEILYRYRSDLEIIFNAIDTDHSGLISMEEFRTMWKLFS
Macaca_nemestrina           YMSSFQNIHIEKPVQEAHSTLVETLYRYRSDLEIIFNAIDTDHSGLISIEEFRAMWKLFS
Chlorocebus_sabaeus         YMSSFQNIHIEKPVQEAHSTLVETLYRYRSDLEIIFNAIDTDHSGLISMEEFRAMWKLFS
Homo_sapiens                YMSSFQNIRIEKPVQEAHSTLVETLYRYRSDLEIIFNAIDTDHSGLISVEEFRAMWKLFS
Pongo_abelii                YMSSFHNIHIEKPVQEAHSTLVETLYRYRSDLEIIFNAIDTDHSGLISMEEFRAMWKLFS
Otolemur_garnetti           YMSSFQDIHIEKPVQQAQSTIVETVYRYRSDLEIIYNMIDSDHSGLISMEEFRAMWKLFS
                            123456789012345678901234567890123456789012345678901234567890
                          521       3         4         5         6         7         8


                                    EF-hand calcium-binding domain 3
                            --|  |------------------------------------|
Brachyteles_arachnoides     SHYNVHIDDSHVNKIASTMDLNRDGNIDFTEFLKAFYVVHKYETMMDPEATNLS
Callithrix_jacchus          SHYNVHIDDSQVNKIANIMDLNRDGSIDFTEFLKAFYVVHKYETTMKPEDTNLD
Aotus_nancymaae             YHYNVHIDDSQVNKIASIMDLNRDGSIDFTEFLKAFYVVHKYETMMKPEATNLG
Cebus_capucinus_imitator    SHYNLHIDNSQVNKIANMMDLNRDGSIDFTEFLKAFYVVHKYETMVKPEATNLX
Saimiri_boliviensis         SHYNLHIDDSQVNKIANIMDLNRDGSIDFTEFLKAFYVVHKYEAMIKPEATNLG
Macaca_nemestrina           AHYHVLIDDSQVNKLANIMDLNKDGSIDFNEFLKAFYVVHRYEDLMKPGVTNLX
Chlorocebus_sabaeus         AHYNVLIDDSQVNKLANLMDLNKDGSIDFNEFLKAFYVVHRYEDLMKPNVTNLX
Homo_sapiens                SHYNVHIDDSQVNKLANIMDLNKDGSIDFNEFLKAFYVVHRYEDLMKPDVTNLG
Pongo_abelii                SHYNVLIDDSQVNKLANIMDLNKDGSIDFNEFLKAFYVVHRYEDLMKPDVTNLX
Otolemur_garnetti           SHYNVPIDESQVDKLANTMDLNKDGSIDFNEFLKAFYVVHKYX-----------
                            1234567890123456789012345678901234567890123
                          581       9       600         1         2
```

PRPS1

```
                          N-terminal domain of ribose phosphate pyrophosphokinase
                         |-----------------------------------------------------------
                                                                *
Brachyteles_arachnoides  MPNIKIFSGSSHQDLSQKIADRLGLELGKVVTKKFSNQETCVEIGESVRGEDVYITQNGC
Callithrix_jacchus       MPNIKIFSGSSHQDLSQKIADRLGLELGKVVTKKFSNQETCVEIGESVRGEDVYIVQSGC
Aotus_nancymaae          MPNIKIFSGSSHQDLSQKIADRLGLELGKVVTKKFSNQETCVEIGESVRGEDVYIVQSGC
Saimiri_boliviensis      MPNIKIFSGSSHQDLSQKIADRLGLELGKVVTKKFSNQETCVEIGESVRGEDVYIVQSGC
Macaca_nemestrina        MPNIKIFSGSSHQDLSQKIADRLGLELGKVVTKKFSNQETCVEIGESVRGEDVYIVQSGC
Chlorocebus_sabaeus      MPNIKIFSGSSHQDLSQKIADRLGLELGKVVTKKFSNQETCVEIGESVRGEDVYIVQSGC
Homo_sapiens             MPNIKIFSGSSHQDLSQKIADRLGLELGKVVTKKFSNQETCVEIGESVRGEDVYIVQSGC
Pongo_abelii             MPNIKIFSGSSHQDLSQKIADRLGLELGKVVTKKFSNQETCVEIGESVRGEDVYIVQSGC
Otolemur_garnetti        MPNIKIFSGSSHQDLSQKIADRLDLELGKVVTKKFSNQETCVEIGESVRGEDVYIVQSGC
                         123456789012345678901234567890123456789012345678901234567890
                                  1         2         3         4         5         6

                         -----------------------------------------------------------|
                                  *
Brachyteles_arachnoides  GEINDNLMEALIMINACKIASASRVTAVIPCFPYARQDKKDKSRAPISAKLVANMLSVAG
Callithrix_jacchus       GEINDNLMELLIMINACKIASASRVTAVIPCFPYARQDKKDKSRAPISAKLVANMLSVAG
Aotus_nancymaae          GEINDNLMELLIMINACKIASASRVTAVIPCFPYARQDKKDKSRAPISAKLVANMLSVAG
Saimiri_boliviensis      GEINDNLMELLIMINACKIASASRVTAVIPCFPYARQDKKDKSRAPISAKLVANMLSVAG
Macaca_nemestrina        GEINDNLMELLIMINACKIASASRVTAVIPCFPYARQDKKDKSRAPISAKLVANMLSVAG
Chlorocebus_sabaeus      GEINDNLMELLIMINACKIASASRVTAVIPCFPYARQDKKDKSRAPISAKLVANMLSVAG
Homo_sapiens             GEINDNLMELLIMINACKIASASRVTAVIPCFPYARQDKKDKSRAPISAKLVANMLSVAG
Pongo_abelii             GEINDNLMELLIMINACKIASASRVTAVIPCFPYARQDKKDKSRAPISAKLVANMLSVAG
Otolemur_garnetti        GEINDGLMELLIMINACKIASASRVTAVIPCFPYARQDKKDKSRAPISAKLVANMLSIAG
                         123456789012345678901234567890123456789012345678901234567890
                        61        7         8         9        100        1         2

                                                    Phosphoribosyltransferase domain
                                                  |----------------------------------
Brachyteles_arachnoides  ADHIITMDLHASQIQGFFDIPVDNLYAEPAVLKWIRENISEWRNCTIVSPDAGGAKRVTS
Callithrix_jacchus       ADHIITMDLHASQIQGFFDIPVDNLYAEPAVLKWIRENISEWRNCTIVSPDAGGAKRVTS
Aotus_nancymaae          ADHIITMDLHASQIQGFFDIPVDNLYAEPAVLKWIRENISEWRNCTIVSPDAGGAKRVTS
Saimiri_boliviensis      ADHIITMDLHASQIQGFFDIPVDNLYAEPAVLKWIRENISEWRNCTIVSPDAGGAKRVTS
Macaca_nemestrina        ADHIITMDLHASQIQGFFDIPVDNLYAEPAVLKWIRENISEWRNCTIVSPDAGGAKRVTS
Chlorocebus_sabaeus      ADHIITMDLHASQIQGFFDIPVDNLYAEPAVLKWIRENISEWRNCTIVSPDAGGAKRVTS
Homo_sapiens             ADHIITMDLHASQIQGFFDIPVDNLYAEPAVLKWIRENISEWRNCTIVSPDAGGAKRVTS
Pongo_abelii             ADHIITMDLHASQIQGFFDIPVDNLYAEPAVLKWIRENISEWRNCTIVSPDAGGAKRVTS
Otolemur_garnetti        ADHIITMDLHASQIQGFFDIPVDNLYAEPAVLKWIRECISEWRNCIIVSPDAGGAKRVVS
                         123456789012345678901234567890123456789012345678901234567890
                       121        3         4         5         6         7         8

                         ------------------------------------------------------------
                                                   |---------------------------------
Brachyteles_arachnoides  IADRLNVDFALIHKERKKANEVDRMVLVGDVKDRVAILVDDMADTCGTICHAADKLLSAG
Callithrix_jacchus       IADRLNVDFALIHKERKKANEVDRMVLVGDVKDRVAILVDDMADTCGTICHAADKLLSAG
Aotus_nancymaae          IADRLNVDFALIHKERKKANEVDRMVLVGDVKDRVAILVDDMADTCGTICHAADKLLSAG
Saimiri_boliviensis      IADRLNVDFALIHKERKKANEVDRMVLVGDVKDRVAILVDDMADTCGTICHAADKLLSAG
Macaca_nemestrina        IADRLNVDFALIHKERKKANEVDRMVLVGDVKDRVAILVDDMADTCGTICHAADKLLSAG
Chlorocebus_sabaeus      IADRLNVDFALIHKERKKANEVDRMVLVGDVKDRVAILVDDMADTCGTICHAADKLLSAG
Homo_sapiens             IADRLNVDFALIHKERKKANEVDRMVLVGDVKDRVAILVDDMADTCGTICHAADKLLSAG
Pongo_abelii             IADRLNVDFALIHKERKKANEVDRMVLVGDVKDRVAILVDDMADTCGTICHAADKLLSAG
Otolemur_garnetti        IADQLNVDFALIHKERKKANEVDRMVLVGDVKDRVAILVDDMADTCGTICLAADKLFSAG
                         123456789012345678901234567890123456789012345678901234567890
                      181         9        200        1         2         3         4

                              Phosphoribosyl synthetase-associated domain
                         ------------------------------------------------------------
Brachyteles_arachnoides  ATRVYAVLTHGIFSGPAISRINNACFEAVVVTNTIPQEDKMKHCSKIQVIDISMILAEAI
Callithrix_jacchus       ATRVYAILTHGIFSGPAISRINNACFEAVVVTNTIPQEDKMKHCSKIQVIDISMILAEAI
Aotus_nancymaae          ATRVYAILTHGIFSGPAISRINNACFEAVVVTNTIPQEDKMKHCSKIQVIDISMILAEAI
Saimiri_boliviensis      ATRVYAILTHGIFSGPAISRINNACFEAVVVTNTIPQEDKMKHCSKIQVIDISMILAEAI
Macaca_nemestrina        ATRVYAILTHGIFSGPAISRINNACFEAVVVTNTIPQEDKMKHCSKIQVIDISMILAEAI
Chlorocebus_sabaeus      ATRVYAILTHGIFSGPAISRINNACFEAVVVTNTIPQEDKMKHCSKIQVIDISMILAEAI
Homo_sapiens             ATRVYAILTHGIFSGPAISRINNACFEAVVVTNTIPQEDKMKHCSKIQVIDISMILAEAI
Pongo_abelii             ATRVYAILTHGIFSGPAISRINNACFEAVVVTNTIPQEDKMKHCSKIQVIDISMILAEAI
Otolemur_garnetti        ATRVYAILTHGIFSGPAISRISSAYFEAIVVTNTIPQEDNMKQCSKIQVIDISMILAEAI
                         123456789012345678901234567890123456789012345678901234567890
                       241        5         6         7         8         9       300
```

```
                         -------------|
Brachyteles_arachnoides  RRTHNGESVSYLFSHVPL
Callithrix_jacchus       RRTHNGESVSYLFSHVPL
Aotus_nancymaae          RRTHNGESVSYLFSHVPL
Saimiri_boliviensis      RRTHNGESVSYLFSHVPL
Macaca_nemestrina        RRTHNGESVSYLFSHVPL
Chlorocebus_sabaeus      RRTHNGESVSYLFSHVPL
Homo_sapiens             RRTHNGESVSYLFSHVPL
Pongo_abelii             RRTHNGESVSYLFSHVPL
Otolemur_garnetti        RRTHNGESVSYLFSHVPL
                         123456789012345678
                       301        1
```

RABEP2

```
                          |--------------- Rabaptin coiled-coil domain ----------------
Brachyteles_arachnoides    METMKAVAEVSESTKAEAVAAVQRQCQEEVASLQAILKDSISSYEAQITALKQERQQQLQ
Callithrix_jacchus         METMKAVAEVSESTKAEAVAAVQRQCQEEVASLQAILKDSISSYEAQITALKQERQQQQQ
Aotus_nancymaae            METMKAVAEVSESTKAEAVAAVQRQCQEEVASLQAILKDSISSYEAQITALKQERQQQQQ
Cebus_capucinus_imitator   METMKAVAEVSESTKAEAVAAVQRQCQEEVASLQAILKDSISSYEAQISALKQERQQQQQ
Saimiri_boliviensis        METMKAVAEVSESTKAEAVAAVQRQCQEEVASLQAILKDSISSYEAQITALKQERQQKQQ
Chlorocebus_sabaeus        METMKAVAEVSESTKAEAVAAVQRQCQEEVASLQAILKDSISSYEAQIAALKQERQQQQQ
Homo_sapiens               METMKAVAEVSESTKAEAVAAVQRQCQEEVASLQAILKDSISSYEAQITALKQERQQQQQ
Pongo_abelii               METMKAVAEVSESTKAEAVAAVQRQCQEEVASLQAILKDSISSYEAQITALKQERQQQQQ
Otolemur_garnetti          METMKAVAEVSESTKAEAVAAVQRQCQEEVASLQAILKDSISSYEAQIASLKQERQQQQQ
                           123456789012345678901234567890123456789012345678901234567890
                                    1         2         3         4         5         6

                           ------------------------------------------------------------
Brachyteles_arachnoides    DCEEKERELGRLKQLLSRAHPLDSLEKQMEKAHEDSEKLREIVLPMEQEIEELKAKLLRA
Callithrix_jacchus         DCEEKERELGRLKQLLSRAHPLDSLEKQMEKAHEDSEKLREIILPMEQEIEELKAKLLRA
Aotus_nancymaae            DCEEKERELGRLKQLLSRAHPLDSLEKQMEKAHEDSEKLREIVLPMEQEIEELKAKLLRA
Cebus_capucinus_imitator   DCEEKERELGRLKQLLSRAHPLDSLEKQMEKAHEDSEKLREIILPMEQEIEELKAKLLRA
Saimiri_boliviensis        DCEEKERELGRLKQLLSRAHPLDSLEKQMEKAHEDSEKLREIILPMEQEIEELKAKLLRA
Chlorocebus_sabaeus        HCEEKERELGRLKQLLSRAHPLDSLEKQMEKAHEDSEKLREIVLPMEKEIEELKEKLLRA
Homo_sapiens               DCEEKERELGRLKQLLSRAYPLDSLEKQMEKAHEDSEKLREIVLPMEKEIEELKAKLLRA
Pongo_abelii               DCEEKERELGRLKQLLSRAHPLDSLEKQMEKAHEDSEKLREIVLPMEKEIEELKAKLLRA
Otolemur_garnetti          DCEEKDRELVRLKQLLSRAHPLDSLEKQMEKAHEDSEKLREIVLPMEQEIEELKVKLLRA
                           123456789012345678901234567890123456789012345678901234567890
                          61        7         8         9        100        1         2

                           -------|
                                                                       *
Brachyteles_arachnoides    EELIQEIQRRPQHAPSLHGSTELLPLSRDPSPPLEPLEELSGDGGPAAEVFAHNCDDSAS
Callithrix_jacchus         EELIQEIQRRPQHAPSLHGSTELLPLSRDPSPPLEPLEELSGDGGPAAEAFAHNCDDSAS
Aotus_nancymaae            EELIQEIQRRPQHAPSLHGSTELLPLSRDPSPPLEPLEELSGDGGPAAEAFAHNCDDSAS
Cebus_capucinus_imitator   EELIQEIQRRPQHAPSLHGSTELLPLSRDPSPPLEPLEELSGDGGPAAEAFAHNCDDSAS
Saimiri_boliviensis        EELIQEIQRRPQHAPSLHGSTELLPLSRDPSPPLEPPEELSGDGGPAAEAFAHNCDDSAS
Chlorocebus_sabaeus        EELIQEIQRRPRHAPSLHDSTELLPLSRDPSPPLEPLEELSGDGGPAAEAFAHNCDDSAS
Homo_sapiens               EELIQEIQRRPRHAPSLHGSTELLPLSRDPSPPLEPLEELSGDGGPAAEAFAHNCDDSAS
Pongo_abelii               EELIQEIQRRPQHVPSLHGSTELLPLSRDPSPPLEPLEELSGDGGPAAEAFAHNCDDSAS
Otolemur_garnetti          EELIQEMQRRPQQPPSLHGSTELLPLSRDPSPPLEPLEELSGDGGAAAEAFAHNCDDSAS
                           123456789012345678901234567890123456789012345678901234567890
                         121         3         4         5         6         7         8

                                                                       |------------
                                              *                                   *
Brachyteles_arachnoides    ISSFSLGGGAGSSASLPRHRQGLSPEQEETASLVSTGTLVPEGIYLPPPGYQLVRDTQWE
Callithrix_jacchus         ISSFSLGGGAGSSASLPRSRQGLSPEQEETASLVSTGTLVPEGIYLPPPGYQLVPDTQWE
Aotus_nancymaae            ISSFSLGGGAGSSASLPRSRQGLSPEQEETASLVSTGTLVPEGIYLPPPGYQLVPDTQWE
Cebus_capucinus_imitator   ISSFSLGGGAGSSASLPRSRQGLSPEQEETASLVSTGTLVPEGIYLPPPGYQLVPDTQWE
Saimiri_boliviensis        ISSFSLGGGAGSSASLPRSRQGLSPEQEETASLVSTGTLVPEGIYLPPPGYQLVPDTQWE
Chlorocebus_sabaeus        ISSFSLGGGVGSSSSLPRSRQGLSPEQEETASLVSTGTLVPEGIYLPPPGYQLVPDTQWE
Homo_sapiens               ISSFSLGGGVGSSSSLPQSRQGLSPEQEETASLVSTGTLVPEGIYLPPPGYQLVPDTQWE
Pongo_abelii               ISSFSLGGGVGSSSSLPRSRQGLSPEQEETASLVSTGTLVPEGIYLPPPGYQLVPDTQWE
Otolemur_garnetti          ISSFSLGGGA-SSTSLPRSRQGLSPEQEETASLVSTGTLVPEGIYLPPPGYQLVPDTQWE
                           1234567890-12345678901234567890123456789012345678901234567890123456789
                         181         9        200        1         2         3

                           ------------------------------------------------------------
Brachyteles_arachnoides    QLQMEGRQLQKDLECVSRERDELQEGLRRSNEDCAKQMQVLLAQVQNSEQLLRTLQGTVS
Callithrix_jacchus         QLQMEGRQLQKDLESISRERDVLQEGLRRSNEDCAKQMQVLLAQVQNSEQLLRTLQGTVS
Aotus_nancymaae            QLQMEGRQLQKDLESVSRERDELQEGLRRSNEDCAKQMQVLLAQVQNSEQLLRTLQGTVS
Cebus_capucinus_imitator   QLQMEGRQLQKDLESVSRERDELQEGLRRSNEDCAKQMQVLLAQVQNSEQLLQTLQGTLS
Saimiri_boliviensis        QLQMEGRQLQKDLESISRERDELQEGLRRSNEDCAKQMQVLLSQVQNSEQLLQTLQGTVS
Chlorocebus_sabaeus        QLQMEGRQLQKDLESVSRERDELQEGLRRSNEDCAKQMQVLLAQVQNSEQLLQTLQGTVS
Homo_sapiens               QLQTEGRQLQKDLESVSRERDELQEGLRRSNEDCAKQMQVLLAQVQNSEQLLRTLQGTVS
Pongo_abelii               QLQMEGRQLQKDLESVSRERDELQEGLRRSNEDCAKQMQVLLAQVQNAEQLLRTLQGTVS
Otolemur_garnetti          QLQVEEW-----------------------------------------------------
                           0123456-----------------------------------------------------
                         240
```

```
                                     Rabaptin, GTPase-Rab5 binding domain
                          ------------------------------------------------------------
Brachyteles_arachnoides   QAQERVQLQMAELVTTHKCLHNEVKRLNEENQGLRAEQPPSSAPQGLQQEQGEEESLPSS
Callithrix_jacchus        QAQERVQLQMAELVTTHKCLHNEVKRLNEENQGLRAEQPPSSAPQGLQQERGEEESLPSS
Aotus_nancymaae           QAQERVQLQMAELVTTHKCLHNEVKRLNEENRGLQAEQPPSSAPQGLQQEQGEEESLPSS
Cebus_capucinus_imitator  QAQERVQLQMAELVTTHKCLHNEVKRLNEENRGLRAEQLPSSAPQGLQQEQGEEESLPSS
Saimiri_boliviensis       QAQERVQLQMAELVTTHKCLHNEVKRLNEENRGLRAEQLPSSAPQGPQQEQGEEESLPSS
Chlorocebus_sabaeus       QAQERVQLQMAELVTTHKCLHHEVKRLNEENQGLRAEQLPSSAPQGLQQEQGEEESLPSS
Homo_sapiens              QAQERVQLQMAELVTTHKCLHHEVKRLNEENQGLRAEQLPSSAPQGSQQEQGEEESLPSS
Pongo_abelii              QAQERVQLQMAELVTTHKCLHHEVKRLNEENQGLRAEQLPSSAPQGPQQEQGEEESLPSS
Otolemur_garnetti         ---EQLQVE-AELVTSHKCLRHEVKRLNEENQGLRAEQPPSSAPQGLEQDEGEEESLPTV
                          ---789012-34567890123456789012345678901234567890123456789012
                           247  5          6         7         8         9       300

                          ------------------------------------------------------------
Brachyteles_arachnoides   VPELQQLLRHTQQEARARLQAQEHGAERLRIEVVTLREALEEETAARASLEGQLRVQREE
Callithrix_jacchus        VPELQQLLRYTQQEAQARLQAQEHGAERLRIEIVTLREALEEETAARASLEGQLRVQREE
Aotus_nancymaae           VSELQQLLRHTQQEARARLQAQEHGAERLRIEIVTLREALEEETAARASLEGQLRVQREE
Cebus_capucinus_imitator  VLELQQLLRHTQQEAQAQLQAQEHGAERLRIEIVTLREALEEETAARASLEGQLRVQREE
Saimiri_boliviensis       VPELQQLLRHTQQEAQARLQAQEHGAERLRIEIVTLREALEEETAARASLEGQLRVQREE
Chlorocebus_sabaeus       VPELQQLLRRTRQEARARLQAQEHGAERLRIEIVTLREALEEETAARASLEGQLRVQRED
Homo_sapiens              VPELQQLLCCTRQEARARLQAQEHGAERLRIEIVTLREALEEETVARASLEGQLRVQREE
Pongo_abelii              VPELQQLLCRTRQEARAQLQAQEHGAERLRIEIVTLREALEEETAARASLEGQLRVQREE
Otolemur_garnetti         VPELQQLVRRTRQEARARQQAQEHAAERLRIEIVTLREVLEEEKAARASLDGQLRVQREE
                          345678901234567890123456789012345678901234567890123456789012
                        303      1         2         3         4         5         6

                          ------------------------|
Brachyteles_arachnoides   TEVLEASLCSLRTEVERVQQEQSKAQLPD-LSEQRAKVLRLQAELETSEQVQRDFVRLSQ
Callithrix_jacchus        TEVLEASLCSLRTEVERVQQEQSKAQLPD-LSEQRAKVLRLQAELETSEQVQRDFVRLSQ
Aotus_nancymaae           TEVLEASLCSLRTEVERVQQEQSKAQLPD-LSEQRAKVLRLQAELETSEQVQRDFVRLSQ
Cebus_capucinus_imitator  TEVLEASLCSLRTEVERVQQEQSKAQLPD-LSEQRAKVLRLQAELETSEQVQRDFVRLSQ
Saimiri_boliviensis       TEVLEASLCSLRTEVERVQQEQSKAQLPD-LSEQRAKVLRLQAELETSEQVQRDFVRLSQ
Chlorocebus_sabaeus       TEVLEASLCSLRTEMERVQQEESKAQLPD-LSEQRAKVLRLQAELETSEQVQRDFVRLSQ
Homo_sapiens              TEVLEASLCSLRTEMERVQQEQSKAQLPD-LSEQRAKVLRLQAELETSEQVQRDFVRLSQ
Pongo_abelii              TEVLEASLCSLRTEMERVQQEQSKPQLPDLLSEQRAKVLRLQAELETSEQVQRDFVRLSQ
Otolemur_garnetti         TEVLEASLCSLRTEMERMQQEQSKAQLPDLLSEQRAKVLRLQAELETSEQVQRDFVRLSQ
                          34567890123456789012345678901-234567890123456789012345678901
                        363      7         8         9        400         1         2

Brachyteles_arachnoides   ALQVRLERIRQAETLEQVRSIMDEAPLTDIRDIKDT
Callithrix_jacchus        ALQVRLERIRQAETLEQVRSIMDEAPLTDIRDIKDT
Aotus_nancymaae           ALQVRLERIRQAETLEQVRSIMDEAPLTDIRDIKDT
Cebus_capucinus_imitator  ALQVRLEQIRQAETLDQVRSIMDEAPLTDIRDIKDT
Saimiri_boliviensis       ALQVRLERIRQAETLEQVRNIMDEAPLTDIRDIKDT
Chlorocebus_sabaeus       ALQVRLERIRQAETLEQVRSIMDEAPLTDIRDIKDT
Homo_sapiens              ALQVRLERIRQAETLEQVRSIMDEAPLTDVRDIKDT
Pongo_abelii              ALQVRLERIRQAETLEQVHSIMDEAPLTDVRDIKDT
Otolemur_garnetti         ALQVRLERIRQAETLEQVRSILDEAPPRDVRDIRDT
                          234567890123456789012345678901234567
                        422       3         4         5
```

SLC15A2

```
                                            *
Brachyteles_arachnoides   MNTFQKNESKETLFSPVSVEEVPRRPPSPPKKPSTKICGSNYPLSIAFIVVNEFCERFSY
Callithrix_jacchus        MNTFQKNESKETLFSPVSIEEVPRRPPSPPKKPSTKICGSNYPLSIAFIVVNEFCERFSY
Aotus_nancymaae           MNTFQKNESKETLFSPVSIEEVPHRPPSPPKKPSTKICGSNYPLSIAFIVVNEFCERFSY
Cebus_capucinus_imitator  MNTFQKNESKETLFSPVSIEEVPRRPPSPPKKPSTKICGSNYPLSIAFIVVNEFCERFSY
Saimiri_boliviensis       MNTFQKNESKETLFSPVSIEEVPRRPPSPPKKPSTKICGSNYPLSIAFIVVNEFCERFSY
Macaca_nemestrina         MNPFQKNESKETLFSPVSIEDVPPRPPSPPKKPSPKICGSNYPLSIAFIVVNEFCERFSY
Chlorocebus_sabaeus       MNPFQKNESKETLFSPVSIEDVPPRPPNPPKKPSPKICGSNYPLSIAFIVVNEFCERFSY
Homo_sapiens              MNPFQKNESKETLFSPVSIEEVPPRPPSPPKKPSPTICGSNYPLSIAFIVVNEFCERFSY
Pongo_abelii              MNPFQKNESKETLFSPVSIEEVPPRPPSPPKKPSPTICGSNYPLSIAFIVVNEFCERFSY
Otolemur_garnetti         MNPFQKNESKETFFSPVSTEEVPHRPPGAPKKPPPKICGSNYPLSIAFIVVNEFCERFSY
                          123456789012345678901234567890123456789012345678901234567890
                                   1         2         3         4         5         6

                                                                 |------ PTR2_1 -------
Brachyteles_arachnoides   YGMKAVLTLYFLYFLHWNEDTSTSIYHAFSSLCYFTPILGAAIADSWLGKFKTIIYLSLV
Callithrix_jacchus        YGMKAVLTLYFLYFLHWNEDTSTSIYHAFSSLCYFTPILGAAIADSWLGKFKTIIYLSLV
Aotus_nancymaae           YGMKAVLTLYFLYFLHWNEDTSTSIYHAFSSLCYFTPILGAAIADSWLGKFKTIVYLSLV
Cebus_capucinus_imitator  YGMKAVLTLYFLYFLHWNEDTSTSIYHAFSSLCYFTPILGAAIADSWLGKFKTIIYLSLV
Saimiri_boliviensis       YGMKAVLTLYFLYFLHWNEDTSTSIYHAFSSLCYFTPILGAAIADSWLGKFKTIIYLSLV
Macaca_nemestrina         YGMKAVLILYFLYFLHWNEDTSTSIYHAFSSLCYFTPILGAAIADSWLGKFKTIIYLSLV
Chlorocebus_sabaeus       YGMKAVLILYFLYFLHWNEDTSTSIYHAFSSLCYFTPILGAAIADSWLGKFKTIIYLSLV
Homo_sapiens              YGMKAVLILYFLYFLHWNEDTSTSIYHAFSSLCYFTPILGAAIADSWLGKFKTIIYLSLV
Pongo_abelii              YGMKAVLILYFLYFLHWNEDTSTSIYHAFSSLCYFTPILGAAIADSWLGKFKTIIYLSLV
Otolemur_garnetti         YGMKAVLTLYFLYFLHWSEDTSTSIYHAFSSLCYFTPILGAAIADSWLGKFKTIIYLSLV
                          123456789012345678901234567890123456789012345678901234567890
                         61        7         8         9        100        1         2

                          ----|
Brachyteles_arachnoides   YVLGHVIKSLGALPILGGQVVHTVLSLVGLSLIALGTGGIKPCVAAFGGDQFEEKHAEER
Callithrix_jacchus        YVLGHVIKSLGALPILGGQVVHTVLSLVGLSLIALGTGGIKPCVAAFGGDQFEETHAEER
Aotus_nancymaae           YVLGHVIKSLGALPILGGQVVHTVLSLVGLSLIALGTGGIKPCVAAFGGDQFEEKHAEER
Cebus_capucinus_imitator  YVLGHVIKSLGALPILGGQVVHTVLSLVGLSLIALGTGGIKPCVAAFGGDQFEEKHAEER
Saimiri_boliviensis       YVLGHVIKSLGALPILGGQVVHTVLSLVGLSLIALGTGGIKPCVAAFGGDQFEENHAEER
Macaca_nemestrina         YVLGHVIKSLGALPILGGQVVHTVLSLVGLSLIALGTGGIKPCVAAFGGDQFEEKHAEER
Chlorocebus_sabaeus       YVLGHVIKSLGALPILGGQVVHTVLSLVGLSLIALGTGGIKPCVAAFGGDQFEEKHAEER
Homo_sapiens              YVLGHVIKSLGALPILGGQVVHTVLSLIGLSLIALGTGGIKPCVAAFGGDQFEEKHAEER
Pongo_abelii              YVLGHVIKSLGALPILGGQVVHTVLSLIGLSLIALGTGGIKPCVAAFGGDQFEEKHAEER
Otolemur_garnetti         YVLGHVVKSLGALPILGGPMVHTVLSLVGLTLIALGTGGIKPCVAAFGGDQFEEKDAEER
                          123456789012345678901234567890123456789012345678901234567890
                        121        3         4         5         6         7         8

                            |-- PTR2_2 --|
Brachyteles_arachnoides   SRYFSVFYLAINAGSLISTFITPMLRGDVQCFGEDCYALAFGVPGLLMVIALVVFAMGSK
Callithrix_jacchus        SRYFSVFYLAINAGSLISTFITPMLRGDVQCFGEDCYALAFGVPGLLMVIALVVFAMGSK
Aotus_nancymaae           SRYFSVFYLAINAGSLISTFITPMLRGDVQCFGEDCYALAFGVPGLLMVIALVVFAMGSK
Cebus_capucinus_imitator  SRYFSVFYLAINAGSLISTFITPMLRGDVQCFGEDCYALAFGVPGLLMVIALVVFAMGSK
Saimiri_boliviensis       SRYFSVFYLAINAGSLISTFITPMLRGDVQCFGEDCYALAFGVPGLLMVIALVVFAMGSK
Macaca_nemestrina         TRYFSVFYLSINAGSLISTFITPMLRGDVQCFGEDCYALAFGVPGLLMVIALVVFAMGSK
Chlorocebus_sabaeus       TRYFSVFYLSINAGSLISTFITPMLRGDVQCFGEDCYALAFGVPGLLMVIALVVFAMGSK
Homo_sapiens              TRYFSVFYLSINAGSLISTFITPMLRGDVQCFGEDCYALAFGVPGLLMVIALVVFAMGSK
Pongo_abelii              TRYFSVFYLSINAGSLISTFITPMLRGDVQCFGEDCYALAFGVPGLLMVIALVVFAMGSK
Otolemur_garnetti         TRYFSVFYLSINAGSLISTFITPMLRGDVQCFGEDCYALAFGVPGLLMVIALVVFALGNK
                          123456789012345678901234567890123456789012345678901234567890
                        181        9        200        1         2         3         4

                                                         *
Brachyteles_arachnoides   MYNKPPPEGNIVAQVLKCIWFAISNRFKNRSLDIPKRQHWLDWAAEKYPKQLIMDVKALT
Callithrix_jacchus        MYNKPPPEGNIVAQVLKCIWFALSNRFKNRSGDIPKRQHWLDWAAEKYPKQLIMDVKALT
Aotus_nancymaae           MYNKPPPEGNIVAQVLKCIWFAISNRFKNRSGDIPKRQHWLDWAAEKYPKQLIMDVKALT
Cebus_capucinus_imitator  MYNKPPPEGNIVAQVLKCIWFAISNRFKNRSGDIPKRQHWLDWAAEKYPKQLIMDVKALT
Saimiri_boliviensis       MYNKPPPEGNIVAQVLKCIWFAISNRFKNRSGDIPKRQHWLDWAAEKYPKRLIMDVKALT
Macaca_nemestrina         IYNKPPPEGNIVAQVFKCIWFAISNRFKNRSGDIPKQQHWLDWAAEKYPKQLIMDVKALT
Chlorocebus_sabaeus       IYNKPPPEGNIVAQVFKCIWFAISNRFKNRSGDIPKRQHWLDWAAEKYPKQLIMDVKALT
Homo_sapiens              IYNKPPPEGNIVAQVFKCIWFAISNRFKNRSGDIPKRQHWLDWAAEKYPKQLIMDVKALT
Pongo_abelii              IYNKPPPEGNIVAQVFKCIWFAISNRFKNRSGDISKRQHWLDWAAEKYPKQLIMDVKALT
Otolemur_garnetti         MYKKPPPEGNIVAQVFKCIWFALSNRFKNRSGDIPKRQHWLDWAVEKYPTQLIMDVKALT
                          123456789012345678901234567890123456789012345678901234567890
                        241        5         6         7         8         9        300
```

```
Brachyteles_arachnoides      RILFLYIPLPMFWALLDQQGSRWTLQATRMNRNLGFFVLQPDQMQVLNPLLVLIFIPLFD
Callithrix_jacchus           RILFLYIPLPMFWALLDQQGSRWTLQATRMNRNLGFFVLQPDQMQVLNPLLVLIFIPLFD
Aotus_nancymaae              RVLFLYIPLPMFWALLDQQGSRWTLQATRMNRNLGFFVLQPDQMQVLNPLLVLIFIPLFD
Cebus_capucinus_imitator     RILFLYIPLPMFWALLDQQGSRWTLQATRMNRNLGFFVLQPDQMQVLNPLLVLIFIPLFD
Saimiri_boliviensis          RVLFLYIPLPMFWALLDQQGSRWTLQATRMNSNLGFFVLQPDQMQVLNPLLVLIFIPLFD
Macaca_nemestrina            RVLFLYIPLPMFWALLDQQGSRWTLQAIRMNGNLGFFVLQPDQMQVLNPLLVLIFIPLFD
Chlorocebus_sabaeus          RVLFLYIPLPMFWALLDQQGSRWTLQAIRMNGNLGFFVLQPDQMQVLNPLLVLIFIPLFD
Homo_sapiens                 RVLFLYIPLPMFWALLDQQGSRWTLQAIRMNRNLGFFVLQPDQMQVLNPLLVLIFIPLFD
Pongo_abelii                 RVLFLYIPLPMFWALLDQQGSRWTLQAIRMNGNLGFFVLQPDQMQVLNPLLVLIFIPLFD
Otolemur_garnetti            RVLFLYIPLPMFWALLDQQGSRWTLQATKMNGNLGFFVLQPDQMQVLNPFLVLIFIPLFD
                             123456789012345678901234567890123456789012345678901234567890
                           301         1         2         3         4         5         6

Brachyteles_arachnoides      LVIYRLVSKCGINFSSLRKMAVGMILACLAFAVAAAVEIKINEMAPAQPGPQEVFLQVLN
Callithrix_jacchus           LVIYRLVSKCGIHFSSLRKMAVGMILACLAFAVAAAVEIKINEMAPAQPGSQEVFLQVLN
Aotus_nancymaae              LVIYRLVSKCGINFSSLRKMAVGMILACLAFAVAAAVEIKINEMAPAQPGSQEVFLQVLN
Cebus_capucinus_imitator     LVIYRLVSKCGINFSSLRKMAVGMILACLAFAVAAAVEIKINEMAPAQPGPQEVFLQVLN
Saimiri_boliviensis          LVIYRLVSKCGINFSSLRKMAVGMILACLAFAVAAAVEIKINEMAPAQPGPQEVFLQVLN
Macaca_nemestrina            FVIYRLVSKCGINFSSLRKMAVGMILACLAFAVAAAVEIKINEMTPAQPGPQEVFLQVLN
Chlorocebus_sabaeus          FVIYRLVSKCGINFSSLRKMAVGMILACLAFAVAAAVEIKINEMTPAQPGPQEVFLQVLN
Homo_sapiens                 FVIYRLVSKCGINFSSLRKMAVGMILACLAFAVAAAVEIKINEMAPAQPGPQEVFLQVLN
Pongo_abelii                 FVIYRLVSKCGINFSSLRKMAVGMILVCLAFAVAAAVEIKINEMAPAQSGPQEVFLQVLN
Otolemur_garnetti            LVIYRLISKCGINFSSLRKMAVGMILACLAFAVAATVEIKINGMAPPQPGPQEIFLQVLN
                             123456789012345678901234567890123456789012345678901234567890
                           361         7         8         9        400        1         2

Brachyteles_arachnoides      LADDEMKVTVLGNENNSLLTESIKSFQKTPHYSKLYLKTKSQDFHFHLKYHNLSVYTEHS
Callithrix_jacchus           LADDEMKVTMLGNENNSLLIESIKSFQKTPHYSKLYLKTKSQDFHFHLKYHNLSVYTEHS
Aotus_nancymaae              LADDEMKVTMLGNENNSLLIESIKSFQKTPHYSKLYLKTKSQDFHFHLKYHNLSVYTEHS
Cebus_capucinus_imitator     LADDEMKVTILENENNSLLIESIKSFQKTPHYSKLYLKTKSQDFHFHLKYHNLSVYTEHS
Saimiri_boliviensis          LADDEMKVTMQGNENNSLLIESIKSFQKTPHYSKLYLKTKSQDFHFHLKYHNLSVNTEHS
Macaca_nemestrina            LADDEVKVTVVGNENDSLLIESIKSFQKTPQYSKLHLKTKSQNFHFHLKYHNLSLYTEHS
Chlorocebus_sabaeus          LADDEVKVTVVGNENNSLLIESIKSFQKTPQYSKLHLKTKSQNFHFHLKYHNLSLYTEHS
Homo_sapiens                 LADDEVKVTVVGNENNSLLIESIKSFQKTPHYSKLHLKTKSQDFHFHLKYHNLSLYTEHS
Pongo_abelii                 LADDEVKVTVVGNENNSLLIESIKSFQKTPHYSKLHLKTKSQDFHFHLKYHNLSLYTEHS
Otolemur_garnetti            LADDDVKVTVVGNENHSLLVESIKSFQKTPHYFRLHLKTKSQDFHFHLKYHNLSVYTEHS
                             123456789012345678901234567890123456789012345678901234567890
                           421         3         4         5         6         7         8

                                        *     *
Brachyteles_arachnoides      VQEQNWYSLFIHEDGNSVSSMMVKDTESKTTNGMTAVRFVNTLHKDVNISLSTDISLNVG
Callithrix_jacchus           VQEQNWYSLFIRKDGNSISSMMVKDTESKTTNGMTAVRFVNTLHKDVNISLSTDISLSVG
Aotus_nancymaae              VQEQNWYSLFIREDGNSISSMMVKDTESKTTNGMTAVRFVNTLHKDVNISLSTDISLNVG
Cebus_capucinus_imitator     VQEQNWYSLFIREDGNSISSMMVKDTESKTTNGMTAVRFVNTLHKDVNITLSTDMSFNVG
Saimiri_boliviensis          VQEQNWYSLFIHENGNSISSTMVKDTESKTTNGMTAVRFVNTLHKVVNISLSTDTSLNVG
Macaca_nemestrina            VQEKNWYSLVIREDGNSISSMMVKDTESKTTNGMTTVRFVNTLHKGVNISLSKDTSLNVG
Chlorocebus_sabaeus          VQEKNWYSLVIREDGNSISSMMVKDTESKTTNGMTTVRFVNTLHKGVNISLSKDTSLNVG
Homo_sapiens                 VQEKNWYSLVIREDGNSISSMMVKDTESRTTNGMTTVRFVNTLHKDVNISLSTDTSLNVG
Pongo_abelii                 VQEKNWYSLVIREDGNSISSMMVKDTESKTTNGMTTVRFVNTLHKDVNISLSTDTSLNVG
Otolemur_garnetti            VEEKNGYSLIIREDGKNISSMMVKDGEGKTTKGMATMRFINTLHKDVNITLGTDISFSVG
                             123456789012345678901234567890123456789012345678901234567890
                           481         9        500        1         2         3         4

Brachyteles_arachnoides      EDYGVSAYRIVQRGEYPAVHCRTEDENFSLNLGLLEFGAAYLFVITNSTNQGLQAWKIED
Callithrix_jacchus           EDYGVSAYRIVQRGEYPAVHCRTEDENFSLNLGLLEFGAAYLFVITNSTNQGLQAWKIED
Aotus_nancymaae              EDYGVSAYRIVQRGEYPAVHCRTEDENFSLNLGLLEFGAAYLFVITNSTNQGLQAWKIED
Cebus_capucinus_imitator     EDYGVSAYRIVQRGEYPAVHCRTEDENFSLNLGLLEFGAAYLFVITNSTNQGLQAWKIAD
Saimiri_boliviensis          EDYGVSAYRIVQRGEYPAVHCRTEDETFSLNLGLLEFGAAYLFVITNSTNQGLQAWKIED
Macaca_nemestrina            EDYGVSAYRTVQRGEYPAVHCRTEDKNFSLNLGLLDFGAAYLFVITNNTNQGLQAWKTED
Chlorocebus_sabaeus          EDYGVSAYRTVQRGEYPAVHCRTEDKNFSLNLGLLDFGAAYLFVITNNTNQGLQAWKTED
Homo_sapiens                 EDYGVSAYRTVQRGEYPAVHCRTEDKNFSLNLGLLDFGAAYLFVITNNTNQGLQAWKIED
Pongo_abelii                 EDYGVSAYRTVQRGEYPSVHCRTEDKNFSLNLGLLDFGAAYLFVITNNTNQGLQAWKIED
Otolemur_garnetti            EDYGVSAYKTVQRGEYPAVHCRTENKDFSLNLGLLDFGAAYLFVITN-TSQGLQAWKMED
                             1234567890123456789012345678901234567-890123456789012345678 9
                           541         5         6         7         8         9
```

```
Brachyteles_arachnoides    IPANKMSIAWQLPQYALVTAAEVMFSVTGLEFSYSQAPSSMKSVLQAAWLLTVAVGNIIV
Callithrix_jacchus         IPANKMSIAWQLPQYALVTAAEVMFSVTGLEFSYSQAPSSMKSVLQAAWLLTVAAGNIIV
Aotus_nancymaae            IPANKMSIAWQLPQYALVTAAEVMFSVTGLEFSYSQAPSSMKSVLQAAWLLTVAVGNIIV
Cebus_capucinus_imitator   IPANKMSIAWQLPQYALVTAAEVMFSVTGLEFSYSQAPSSMKSVLQAAWLLTVAVGNIIV
Saimiri_boliviensis        IPANKMSIAWQLPQYALVTAAEVMFSVTGLEFSYSQAPSSMKSVLQAAWLLTVAVGNIIV
Macaca_nemestrina          IPANKMSIAWQLPQYALVTAGEVMFSVTGLEFSYSQAPSSMKSVLQAAWLLTIAVGNIIV
Chlorocebus_sabaeus        IPANRMSIAWQLPQYALVTAGEVMFSVTGLEFSYSQAPSSMKSVLQAAWLLTIAVGNIIV
Homo_sapiens               IPANKMSIAWQLPQYALVTAGEVMFSVTGLEFSYSQAPSSMKSVLQAAWLLTIAVGNIIV
Pongo_abelii               IPANKMSIAWQLPQYALVTAGEVMFSVTGLEFSYSQAPSSMKSVLQAAWLLTIAVGNIIV
Otolemur_garnetti          IPANKMSIAWQLPQYALVTAGEVMFSVTGLEFSYSQAPSSMKSVLQAAWLLTVAVGNIIV
                           012345678901234567890123456789012345678901234567890123456789
                         600         1         2         3         4         5

Brachyteles_arachnoides    LVVAQFSGLVQWAEFILFSCLLLVVCLIFSIMGYNYVPVKTEDRQGPADKQIPHIQGNMI
Callithrix_jacchus         LVVAQFSGLVQWAEFILFSCLLLVVCLLFSIMGYNYVPVKTEDMQDPADKQIPHIQGNVI
Aotus_nancymaae            LVVAQFSGLVQWAEFILFSCLLLVVCLIFSIMGYNYVPVKTEDMQGPADKQIPHIQGNMI
Cebus_capucinus_imitator   LVVAQFSGLVQWAEFILFSCLLLVVCLIFSIMGYNYVPVKTEDMQGPADKQIPHIQGNMI
Saimiri_boliviensis        LVVAQFSGLVQWAEFILFSCLLLVVCLIFSIMGYNYVPVKTEDMQGPADKQIPHIQGNMI
Macaca_nemestrina          LVVAQFSGLVQWAEFILFSCLLLVICLIFSIMGYYYVPVKTEDMQGPGDKQISHIQGNMI
Chlorocebus_sabaeus        LVVAQFSGLVQWAEFILFSCLLLVICLIFSIMGYYYVPVKTEDMQGPGDKQIPHIQGNMI
Homo_sapiens               LVVAQFSGLVQWAEFILFSCLLLVICLIFSIMGYYYVPVKTEDMRGPADKHIPHIQGNMI
Pongo_abelii               LVVAQFSGLVQWAEFILFSCLLLVICLIFSIMGYYYVPVKTEDMRGPADKQIPHIQGNMI
Otolemur_garnetti          LVVAQFSGLVQWAEFILFSCLLLVVCLIFSIMGYYYVPVKPEDMQGPADKQFPHTQGDMI
                           012345678901234567890123456789012345678901234567890123456789
                         660         7         8         9       700         1

Brachyteles_arachnoides    NLETKKTK
Callithrix_jacchus         NLETKKTK
Aotus_nancymaae            NLETKKT.
Cebus_capucinus_imitator   NLETKKTK
Saimiri_boliviensis        NLETKKTK
Macaca_nemestrina          NLETKKTK
Chlorocebus_sabaeus        NLETKKTK
Homo_sapiens               KLETKKTK
Pongo_abelii               NLETKKTK
Otolemur_garnetti          NLETKKT.
                           01234567
                         720
```

SLC39A13

```
                             |-- Transmembrane -|---------- Cytoplasmic ---------------|-
                                                *
Brachyteles_arachnoides      MAGPRLLFLTALAVELLGRASGSQPALQSRGTATACRLDNKESESWGALLSGERLDTWIC
Callithrix_jacchus           MAGPRLLFLTALAVELLGRAGGSQSALQSRGTATACRLDNKESESWGALLSGERLDTWIC
Aotus_nancymaae              MAGPRLLFLTALVVELLGRAGGSQPALQSRGTATACRLDNKESESWGALLSGERLDTWIC
Cebus_capucinus_imitator     MAGPRLLFVTTLVVELLGRAGGSQPALQSRGTATACRLDNKESESWGALLSGERLDTWIC
Saimiri_boliviensis          MVGPRLFFLTALAVELLGRAGGSQPALQSRGTATACRLDNKESESWGALLSGERLDTWIC
Macaca_nemestrina            MAGPRLLFLTALALELLGRAGGSQPALRSRGTATACRLDNKESESWGALLSGERLDTWIC
Papio_anubis                 MAGPRLLFLTALALELLGRAGGSQPALRSRGTATACRLDNKESESWGALLSGERLDTWIC
Chlorocebus_sabaeus          MAGPRLLFLTALALELLGRAGGSQPALRSRGTATACRLDNKESESWGALLSGERLDTWIC
Homo_sapiens                 MAGPRLLFLTALALELLERAGGSQPALRSRGTATACRLDNKESESWGALLSGERLDTWIC
Pongo_abelii                 MAGPRLLFLTALALELLGRAGGSQPALRSRGTATACRLDNKESESWGALLSGERLDTWIC
Otolemur_garnetti            MAGQRLLFLTALALELLGMAGGSQPALRSWGAAAACRLDNKESDSWGALLSGERLDTWIC
                             123456789012345678901234567890123456789012345678901234567890
                                      1         2         3         4         5         6

                             -- Transmembrane --|---- Lumenal ----|--- Transmembrane --|-
                                               *
Brachyteles_arachnoides      SLLGSLMVGLSGVFPLLLIPLEMGTMLRSEAGAWRLKQLLSFALGGLLGNVFLHLLPEAW
Callithrix_jacchus           SLLGSLMVGLSGVFPLLVIPLEMGTMLCSEAGAWRLKQLLSFALGGLLGNVFLHLLPEAW
Aotus_nancymaae              SLLGSLMVGLSGVFPLLVIPLEMGTMLRSEAGAWRLKQLLSFALGGLLGNVFLHLLPEAW
Cebus_capucinus_imitator     SLLGSLMVGLSGVFPLLVIPLEMGTMLRSEAGARRLKQLLSFALGGLLGNVFLHLLPEAW
Saimiri_boliviensis          SLLGSLMVGLSGVFPLLVIPLEMGTMLRSEAGAWRLKQLLSFALGGLLGNVFLHLLPEAW
Macaca_nemestrina            SLLGSLMVGLSGVFPLLVIPLEMGTMLRSEAGAWRLKQLLSFALGGLLGNVFLHLLPEAW
Papio_anubis                 SLLGSLMVGLSGVFPLLVIPLEMGTMLRSEAGAWRLKQLLSFALGGLLGNVFLHLLPEAW
Chlorocebus_sabaeus          SLLGSLMVGLSGVFPLLVIPLEMGTMLRSEAGAWRLKQLLSFALGGLLGNVFLHLLPEAW
Homo_sapiens                 SLLGSLMVGLSGVFPLLVIPLEMGTMLRSEAGAWRLKQLLSFALGGLLGNVFLHLLPEAW
Pongo_abelii                 SLLGSLMVGLSGVFPLLVIPLEMGTMLRSEAGAWHLKQLLSFALGGLLGNVFLHLLPEAW
Otolemur_garnetti            SLLGSLMVGLSGVFPLLVIPLEMGTMLRSEAGARRLKQLLSFALGGLLGNVFLHLLPEAW
                             123456789012345678901234567890123456789012345678901234567890
                            61        7         8         9        100         1         2

                             --- Cytoplasmic ---|-- Transmembrane --|------ Lumenal -----
Brachyteles_arachnoides      AYTCSTGPGGEGQSLQQQQQLGLWVIAGILTFLALE-MFLDSKE-EGTSQAPSKDPTAAA
Callithrix_jacchus           AYTCSTGPGGEGQSLQQQQQLGLWVIAGILTFLALE-MFLDSKE-EGTSQAPSKDPTAAA
Aotus_nancymaae              AYTCSTGPDGEGQSLQQQQQLGLWVIAGILTFLVLEKMFLDSKE-EGASQAPSKDPTAAA
Cebus_capucinus_imitator     AYTCSTGPGGEGQSLQQQQQLGLWVIAGILTFLVLEKMFLDSKE-EGTSQAPSKDPTAAA
Saimiri_boliviensis          AYTCSTGPGGEGQSLQQQQRLGLWVIAGILTFLALE-MFLDSKE-EGTSQAPSKDPTAAA
Macaca_nemestrina            AYTCSAGPGGEGQSLQQQQQLGLWVIAGILTFLALEKMFLDSKA-ERTSQAPNKDPTAAA
Papio_anubis                 AYTCSAGPGGEGQSLQQQQQLGLWVIAGILTFLALEKMFLDSKA-ERTSQAPNKDPTAAA
Chlorocebus_sabaeus          AYTCSAGPGGEGQSLQQQQQLGLWVIAGILTFLALEKMFLDSKA-EGTSQAPNKDPTAAA
Homo_sapiens                 AYTCSASPGGEGQSLQQQQQLGLWVIAGILTFLALE-MFLDSKE-EGTSQAPNKDPTAAA
Pongo_abelii                 AYTCSASPGGEGQSLQQQQQLGLWVIAGILTFLALEKMFLDSKE-EGTSQAPNKDPTAAA
Otolemur_garnetti            AYTCSASPGGEGQSLQQQQQLGLWVIAGILSFLALEKMFLDNKERQGTTQAPSKDPTTAA
                             123456789012345678901234567890123456-7890123-45678901234567 8
                           121         3         4         5          6          7

                             ------------ Lumenal -----------------------|-Transmembrane
Brachyteles_arachnoides      AALNGGHCLAQPAAEPGLGAVVRSIKVSGYLNLLANTIDNFTHGLAVAASFLVSKKIGLL
Callithrix_jacchus           VTLNGGHCLAQPAAEPGLGAVVRSIKVSGYLNLLANTIDNFTHGLAVAASFLVSKKIGLL
Aotus_nancymaae              AALNGGHCLAQPTAEPGLGAVVRSIKVSGYLNLLANTIDNFTHGLAVAASFLVSKKIGLL
Cebus_capucinus_imitator     TTLNGGHCLAQPAAEPGLGAVVRSIKVSGYLNLLANTIDNFTHGLAVAASFLVSKKIGLL
Saimiri_boliviensis          ATLNGGHCLAQPAAEPGLGAVVRSIKVSGYLNLLANTIDNFTHGLAVAASFLVSKKIGLL
Macaca_nemestrina            AALNGGHCLAQPAAEPGLGAVVRSIKVSGYLNLLANTIDNFTHGLAVAASFLVSKKIGLL
Papio_anubis                 AALNGGHCLAQPAAEPGLGAVVRSIKVSGYLNLLANTIDNFTHGLAVAASFLVSKKIGLL
Chlorocebus_sabaeus          AALNGGHCLAQPAAEPGLGAVVRSIKVSGYLNLLANTIDNFTHGLAVAASFLVSKKIGLL
Homo_sapiens                 AALNGGHCLAQPAAEPGLGAVVRSIKVSGYLNLLANTIDNFTHGLAVAASFLVSKKIGLL
Pongo_abelii                 AALNGGHCLAQPAAEPGLGAVVRSIKVSGYLNLLANTIDNFTHGLAVAASFLVSKKIGLL
Otolemur_garnetti            ATLNGGRCLAQPVAEPGLSVVVRSIKVRGYLNLLANTIDNFTHGLAVAASFLVSKKIGFL
                             901234567890123456789012345678901234567890123456789012345678
                            180        9       200         1         2         3
```

```
                          ------|---- Cytoplasmic ----|-- Transmembrane ---| Lumenal -
Brachyteles_arachnoides   TTMAILLHEIPHEVGDFAILLRAGFDRWSAAKLQLSTALGGLLGAGFAICTQSPKGVEET
Callithrix_jacchus        TTMAILLHEIPHEVGDFAILLRAGFDRWSAAKLQLSTALGGLLGAGFAICTQSPKGVEET
Aotus_nancymaae           TTMAILLHEIPHEVGDFAILLRAGFDRWSAAKLQLSTALGGLLGAGFAICTQSPKGVEET
Cebus_capucinus_imitator  TTMAILLHEIPHEVGDFAILLRAGFDRWSAAKLQLSTALGGLLGAGFAICTQSPKGVEET
Saimiri_boliviensis       TTMAILLHEIPHEVGDFAILLRAGFDRWSAAKLQLSTALGGLLGAGFAICTQSPKGVEET
Macaca_nemestrina         TTMAILLHEIPHEVGDFAILLRAGFDRWSAAKLQLSTALGGLLGAGFAICTQSPKGVEET
Papio_anubis              TTMAILLHEIPHEVGDFAILLRAGFDRWSAAKLQLSTALGGLLGAGFAICTQSPKGVEET
Chlorocebus_sabaeus       TTMAILLHEIPHEVGDFAILLRAGFDRWSAAKLQLSTALGGLLGAGFAICTQSPKGVEET
Homo_sapiens              TTMAILLHEIPHEVGDFAILLRAGFDRWSAAKLQLSTALGGLLGAGFAICTQSPKGVEET
Pongo_abelii              TTMAILLHEIPHEVGDFAILLRAGFDRWSAAKLQLSTALGGLLGAGFAICTQSPKGVEET
Otolemur_garnetti         TTMAILLHEIPHEVGDFAILLRAGFDRWSAAKLQLSTALGGLLGACFAICTQSPKGVEET
                          901234567890123456789012345678901234567890123456789012345678
                         240         5         6         7         8         9

                          |-- Transmembrane --|Cytoplasmic|-- Transmembrane ---|
Brachyteles_arachnoides   AAWVLPFTSGGFLYIALVNVLPDLLEEEDPWRSLQQLLLLCTGIMVMVLFSLFVD
Callithrix_jacchus        AAWVLPFTSGGFLYIALVNVLPDLLEEEDPWRSLQQLLLLCAGIMVMVLFSLFVD
Aotus_nancymaae           AAWVLPFTSGGFLYIALVNVLPDLLEEEDPWRSLQQLLLLCAGIMVMVLFSLFVD
Cebus_capucinus_imitator  VAWVLPFTSGGFLYIALVNVLPDLLEEEDPWRSLQQLLLLCAGIMVMVLFSLFVD
Saimiri_boliviensis       AAWVLPFTSGGFLYIALVNVLPDLLEEEDPWRSLQQLLLLCAGIMVMVLFSLFVD
Macaca_nemestrina         AAWVLPFTSGGFLYIALVNVLPDLLEEEDPWRSLQQLLLLCAGIVVMVLFSIFVD
Papio_anubis              AAWVLPFTSGGFLYIALVNVLPDLLEEEDPWRSLQQLLLLCAGIVVMVLFSIFVD
Chlorocebus_sabaeus       AAWVLPFTSGGFLYIALVNVLPDLLEEEDPWRSLQQLLLLCAGIVVMVLFSIFVD
Homo_sapiens              AAWVLPFTSGGFLYIALVNVLPDLLEEEDPWRSLQQLLLLCAGIVVMVLFSLFVD
Pongo_abelii              AAWVLPFTSGGFLYIALVNVLPDLLEEEDPWRSLQQLLLLCAGIVVMVLFSLFVD
Otolemur_garnetti         VAWILPFTSGGFLYIALVNVLPDLLEEDDPWHSLQQVILLCTGIMVMVLFSLFV-
                          901234567890123456789012345678901234567890123456789012
                         500         1         2         3         4         5
```

SOAT1

```
                                                    **
Brachyteles_arachnoides    MVGEEKMSLRNRLSKSRENPEEDEDGTNPAKESLETPSNGRIDIKQLIAKKIQLTAEAEE
Callithrix_jacchus         MVGEEKMSLRNRLSKSRENPEEDEDRRSPVKESLETPSNGRIDIKQLIAKKIQLTAEAEE
Cebus_capucinus_imitator   MVGEEKMSLRNRLSKSRENPEEDEDQRNPAKESLETPSNGRIDIKQLIAKKIQLTAEAEE
Saimiri_boliviensis        MVGEENMSLRNRLSKSRENPEEDEDQRNPAKESLETPSNGRIDIKQLIAKKIQLTAEAEE
Macaca_nemestrina          MVGEEKMSLRNRLSKSRENPEEDEDQRKPAKESLEAPSNGRIDIKQLIAKKIKLTAEAEE
Papio_anubis               MVGEEKMSLRNRLSKSRENPEEDEDQRKPAKESLEAPSNGRIDIKQLIAKKIKLTAEAEE
Chlorocebus_sabaeus        MVGEEKMSLRNRLSKSRENPEEDEDQRKPAKESLEAPSNGRIDIKQLIAKKIKLTAEAEE
Homo_sapiens               MVGEEKMSLRNRLSKSRENPEEDEDQRNPAKESLETPSNGRIDIKQLIAKKIKLTAEAEE
Pongo_abelii               MVGEEKMSLRNRLSKSRENPEEDEDQRNPAKESLETPSNGRIDIKQLIAKKIKLTAEAEE
Otolemur_garnetti          MVGEEKMSLRNRLSKSCENPEEDEDQRNPTEESLQRPSNGRIDIKQLIAKKIQLTAEAEE
                           123456789012345678901234567890123456789012345678901234567890
                                    1         2         3         4         5         6
```

```
Brachyteles_arachnoides    LKPFFMKEVGSHFDDFVTNLIEKSASLDNGGCALTTFSVLEGEKNNHRAKDLRAPPEQGK
Callithrix_jacchus         LKPFFMKEVGSHFDDFVTNLIEKSASLDNGGCALTTFSVLEGEKTNHRAKDLRAPPEQGK
Cebus_capucinus_imitator   LKPFFMKEVGSHFDDFVTNLIEKSASLDNGGCALTTFSVLEGEKNNHRAKDLRAPPEQGK
Saimiri_boliviensis        LKPFFMKEVGSHFDDFVTNLIEKSASLDNGGCALTTFSVLEGEKNNHRAKDLRAPPEQGK
Macaca_nemestrina          LKPFFMKEVGSHFDDFVTNLIEKSASLDNGGCALTTFSILEGEKNNHRAKDLRAPPEQGK
Papio_anubis               LKPFFMKEVGSHFDDFVTNLIEKSASLDNGGCALTTFSILEGEKNNHRAKDLRAPPEQGK
Chlorocebus_sabaeus        LKPFFMKEVGSHFDDFVTNLIEKSASLDNGGCALTTFSILEGEKNNHRAKDLRAPPEQGK
Homo_sapiens               LKPFFMKEVGSHFDDFVTNLIEKSASLDNGGCALTTFSVLEGEKNNHRAKDLRAPPEQGK
Pongo_abelii               LKPFFMKEVGSHFDDFVTNLIEKSASLDNGGCALTTFSVLEGEKNNHRVKDLRAPPEQGK
Otolemur_garnetti          LKPFFMKEIGNHFDDFVTNLIEKSASLDNGGCALTTFSVLEGEKNNHRTKDLRASPDQGK
                           123456789012345678901234567890123456789012345678901234567890
                          61        7         8         9        100        1         2
```

```
                                                                          |---------
Brachyteles_arachnoides    IFIARRSLLDELLEVDHIRTIYHMFIALLILFILSTLVVDYIDEGRLVLEFSLLSYAFGK
Callithrix_jacchus         IFIARRSLLDELLEVDHIRTIYHMFIALLILFILSTLVVDYIDEGRLVLEFSLLSYAFGK
Cebus_capucinus_imitator   IFIARRSLLDELLEVDHIRTIYHMFIALLILFILSTLVVDYIDEGRLVLEFSLLSYAFGK
Saimiri_boliviensis        IFIARRSLLDELLEVDHIRTIYHMFIALLILFILSTLVVDYIDEGRLVLEFSLLSYAFGK
Macaca_nemestrina          IFIARRSLLDELLEVDHIRTIYHMFIALLILFILSTLVVDYIDEGRLVLEFSLLSYAFGK
Papio_anubis               IFIARRSLLDELLEVDHIRTIYHMFIALLILFILSTLVVDYIDEGRLVLEFSLLSYAFGK
Chlorocebus_sabaeus        IFIARRSLLDELLEVDHIRTIYHMFIALLILFILSTLVVDYIDEGRLVLEFSLLSYAFGK
Homo_sapiens               IFIARRSLLDELLEVDHIRTIYHMFIALLILFILSTLVVDYIDEGRLVLEFSLLSYAFGK
Pongo_abelii               IFIARRSLLDELLEVDHIRTIYHMFIALLILFILSTLVVDYIDEGRLVLEFSLLSYAFGK
Otolemur_garnetti          IFITRRSLLDELLEVDHIRTIYHMFIALLILFILSTLVVDYIDEGRLVLEFSLLSYAFGK
                           123456789012345678901234567890123456789012345678901234567890
                         121         3         4         5         6         7         8
```

```
                           --------- MBOAT, membrane-bound O-acyltransferase ----------
Brachyteles_arachnoides    FPTVVWTWWIMFLSTFSVPYFLFQCWATGYSRSSHPLIHSLFHGFLFMVFQIGVLGFGPT
Callithrix_jacchus         FPTVIWTWWIMFLSTFSVPYFLFQCWATGYSRSSHPLIHSLFHGFLFMVFQIGVLGFGPT
Cebus_capucinus_imitator   FPTVVWTWWIMFLSTFSVPYFLFQCWATGYSRSSHPLIHSFFHGFLFMVFQIGVLGFGPT
Saimiri_boliviensis        FPTVVWTWWIMFLSTFSVPYFLFQCWATGYSRSSHPLIHSLFHGFLFMVFQIGVLGFGPT
Macaca_nemestrina          FPTVVWTWWIMFLSTFSVPYFLFQRWATGYSKSSHPLINSLFHGFLFMVFQIGILGFGPT
Papio_anubis               FPTVVWTWWIMFLSTFSVPYFLFQRWATGYSKSSHPLINSLFHGFLFMVFQIGILGFGPT
Chlorocebus_sabaeus        FPTVVWTWWIMFLSTFSVPYFLFQRWATGYSKSSHPLINSLFHGFLFMVFQIGILGFGPT
Homo_sapiens               FPTVVWTWWIMFLSTFSVPYFLFQHWATGYSKSSHPLIRSLFHGFLFMIFQIGVLGFGPT
Pongo_abelii               FPTVVWTWWIMFLSTLSVPYFLFQHWATGYSKSSHPLIRSLFHGFLFMIFQIGVLGFGPT
Otolemur_garnetti          FPTVIWTWWIMFLSTLAVPYFLFDRWASGYSKSSHPLIHSLFHGLLFMIFQIGVLGFVPV
                           123456789012345678901234567890123456789012345678901234567890
                         181         9       200         1         2         3         4
```

```
                           ------------------------------------------------------------
Brachyteles_arachnoides    YVVLAYTLPPASRFIIIFEQIRFVMKAHSFVRENVPRVLNSAKEKSSTIPIPTVNQYLYF
Callithrix_jacchus         YVVLAYTLPPASRFIVIFEQIRFIMKAHSFVRENVPRVLNSAKEKSSIIPIPTVNQYLYF
Cebus_capucinus_imitator   YVVLAYTLPPASRFIVIFEQIRFVMKAHSFVRENVPRVLNSAKEKSSTIPIPTVNQYLYF
Saimiri_boliviensis        YVVLAYTLPPASRFIVIFEQIRFVMKAHSFVRENVPRVLNSAKEKSSTIPIPTVNQYLYF
Macaca_nemestrina          YVVLAYTLPPASRFIIIFEQIRFVMKAHSFVRENVPRVLNSAKEKSSTVPIPTVNQYLYF
Papio_anubis               YVVLAYTLPPASRFIIIFEQIRFVMKAHSFVRENVPRVLNSAKEKSSTVPIPTVNQYLYF
Chlorocebus_sabaeus        YVVLAYTLPPASRFIIIFEQIRFVMKAHSFVRENVPRVLNSAKEKSSTVPIPTVNQYLYF
Homo_sapiens               YVVLAYTLPPASRFIIIFEQIRFVMKAHSFVRENVPRVLNSAKEKSSTVPIPTVNQYLYF
Pongo_abelii               YVVLAYTLPPASRFIIIFEQIRFVMKAHSFVRENVPRVLNSSKEKSSTVPIPTVNQYLYF
Otolemur_garnetti          YVVLAYTLPPASRFIVILEQIRLIMKAYSFVRENVPRVLNSAKEKSSAVPIPTVNQYLYF
                           123456789012345678901234567890123456789012345678901234567890
                         241         5         6         7         8         9       300
```

```
                           ------------------------------------------------------------
Brachyteles_arachnoides    LFAPTLIYRDSYPRTPTVRWGYVAMQFAQVFGCFFYVYYIFERLCAPLFRNIKQEPFSAR
Callithrix_jacchus         LFAPTLIYRDSYPRTPTVRWGYVAMQFAQVFGCFFYVYYIFERLCAPLFRNIKQEPFSAR
Cebus_capucinus_imitator   LFAPTLIYRDSYPRTPTVRWGYVAMQFAQ-------------------------------
Saimiri_boliviensis        LFAPTLIYRDSYPRTPTVRWGYVAMQFAQVFGCFFYVYYIFERLCAPLFRNIKQEPFSAR
Macaca_nemestrina          LFAPTLIYRDSYPRNPTVRWGYVAMQFAQVFGCFFYVYYIFERLCAPLFRNIKQEPFSAR
Papio_anubis               LFAPTLIYRDSYPRNPTVRWGYVAMQFAQVFGCFFYVYYIFERLCAPLFRNIKQEPFSAR
Chlorocebus_sabaeus        LFAPTLIYRDSYPRNPTVRWGYVAMQFAQVFGCFFYVYYIFERLCAPLFRNIKQEPFSAR
Homo_sapiens               LFAPTLIYRDSYPRNPTVRWGYVAMKFAQVFGCFFYVYYIFERLCAPLFRNIKQEPFSAR
Pongo_abelii               LFAPTLIYRDSYPRNPTVRWGYVAMKFAQVFGCFFYVYYIFERLCAPLFRNIKQEPFSAR
Otolemur_garnetti          LFAPTLIYRDSYPRTPTVRWGYVTMQFAQVFGCFCYVYYIFERLCAPLFRNIKQEPFSAR
                           12345678901234567890123456789-------------------------------
                         301         1         2

                           ------------------------------------------------------------
Brachyteles_arachnoides    VLVLCVFNSILPGVLILFLTFFAFLHCWLNAFAEMLRFGDRMFYKDWWNSTSYSNYYRTW
Callithrix_jacchus         VLVLCVFNSILPGVLILFLTFFAFLHCWLNAFAEMLRFGDRMFYKDWWNSTSYSNYYRTW
Cebus_capucinus_imitator   -----------.GVLILFLTFFAFLHCWLNAFAEMLRFGDRMFYKDWWNSTSYSNYYRTW
Saimiri_boliviensis        VLVLCVFNSILPGVLILFLTFFAFLHCWLNAFAEMLRFGDRMFYKDWWNSTSYSNYYRTW
Macaca_nemestrina          VLVLCVFNSILPGVLILFLTFFAFLHCWLNAFAEMLRFGDRMFYKDWWNSTSYSNYYRTW
Papio_anubis               VLVLCVFNSILPGVLILFLTFFAFLHCWLNAFAEMLRFGDRMFYKDWWNSTSYSNYYRTW
Chlorocebus_sabaeus        VLVLCVFNSILPGVLILFLTFFAFLHCWLNAFAEMLRFGDRMFYKDWWNSTSYSNYYRTW
Homo_sapiens               VLVLCVFNSILPGVLILFLTFFAFLHCWLNAFAEMLRFGDRMFYKDWWNSTSYSNYYRTW
Pongo_abelii               VLVLCVFNSILPGVLILFLTFFAFLHCWLNAFAEMLRFGDRMFYKDWWNSTSYSNYYRTW
Otolemur_garnetti          VLVLCVFNSILPGVLILLLSFFAFLHCWLNAFAEMLRFGDRMFYKDWWNSTSYSNYYRTW
                           ------------012345678901234567890123456789012345678901234567
                                       330       4         5         6         7

                           ------------------------------------------------------------
                                                                  [H] active site
Brachyteles_arachnoides    NVVVHDWLYYYAYKDFLWFFSKRFKSAAMLAVFAVSAVVHEYALAVCLSFFYPVLFVLFM
Callithrix_jacchus         NVVVHDWLYYYAYKDFLWFFSKRFKSAAMLAVFAVSAVVHEYALAVCLSFFYPVLFVLFM
Cebus_capucinus_imitator   NVVVHDWLYYYAYKDFLWFFSKRFKSAAMLAVFAVSAVVHEYALAVCLSFFYPVLFVLFM
Saimiri_boliviensis        NVVVHDWLYYYAYKDFLWFFSKRFKSAAMLAVFAVSAVVHEYALAVCLSFFYPVLFVLFM
Macaca_nemestrina          NVVVHDWLYYYAYKDFLWFFSKRFKSAAMLAVFAVSAVVHEYALAVCLSFFYPVLFVLFM
Papio_anubis               NVVVHDWLYYYAYKDFLWFFSKRFKSAAMLAVFAVSAVVHEYALAVCLSFFYPVLFVLFM
Chlorocebus_sabaeus        NVVVHDWLYYYAYKDFLWFFSKRFKSAAMLAVFAVSAVVHEYALAVCLSFFYPVLFVLFM
Homo_sapiens               NVVVHDWLYYYAYKDFLWFFSKRFKSAAMLAVFAVSAVVHEYALAVCLSFFYPVLFVLFM
Pongo_abelii               NVVVHDWLYYYAYKDFLWFFSKRFKSAAMLAVFAVSAVVHEYALAVCLSFFYPVLFVLFM
Otolemur_garnetti          NVVVHDWLYYYAYKDFLWLFSKRFKSAAMLAVFAVSAVVHEYALAVCLSFFYPVLFVLFM
                           890123456789012345678901234567890123456789012345678901234567
                           380       9         400       1         2         3

                           ------------------------------------------------------------
Brachyteles_arachnoides    FFGMAFNFIVNDSRKKPIWNVLMWTSLFLGNGVILCFYSQEWYARQHCPLKNPTFLDYVR
Callithrix_jacchus         FFGMAFNFIVNDSRKKPIWNVLMWTSLFLGNGVLLCFYSQEWYARQHCPLKNPTFLDYVR
Cebus_capucinus_imitator   FFGMAFNFIVNDSRKKPIWNVLMWTSLFLGNGVILCFYSQEWYARQHCPLKNPTFLDYVR
Saimiri_boliviensis        FFGMAFNFIVNDSRKKPIWNVLMWTSLFLGNGVILCFYSQEWYARQHCPLKNPTFLDYVR
Macaca_nemestrina          FFGMAFNFIVNDSRKKPIWNVMMWTSLFLGNGVLLCFYSQEWYARQHCPLKNPTFLDYVR
Papio_anubis               FFGMAFNFIVNDSRKKPIWNVMMWTSLFLGNGVLLCFYSQEWYARQHCPLKNPTFLDYVR
Chlorocebus_sabaeus        FFGMAFNFIVNDSRKKPIWNVMMWTSLFLGNGVLLCFYSQEWYARQHCPLKNPTFLDYVR
Homo_sapiens               FFGMAFNFIVNDSRKKPIWNVLMWTSLFLGNGVLLCFYSQEWYARQHCPLKNPTFLDYVR
Pongo_abelii               FFGMAFNFIVNDSRKKPIWNVLMWTSLFLGNGVLLCFYSQEWYARQHCPLKNPTFLDYVR
Otolemur_garnetti          FFGMAFNFIVNDRRKRPIWNVMMWTSLFLGNGVMLCFYSQEWYARQHCPLKNPTFLDYIQ
                           890123456789012345678901234567890123456789012345678901234567
                           440       5         6         7         8         9

                           --------|
                             *
Brachyteles_arachnoides    PRTWTCRY.-
Callithrix_jacchus         PRSWTCRY.-
Cebus_capucinus_imitator   PRSWTCRYVF
Saimiri_boliviensis        PRSWTCRY.-
Macaca_nemestrina          PRSWTCRY.-
Papio_anubis               PRSWTCRY.-
Chlorocebus_sabaeus        PRSWTCRY.-
Homo_sapiens               PRSWTCRY.-
Pongo_abelii               PRSWTCRY.-
Otolemur_garnetti          PRSWTCQY.-
                           89012345--
                           500
```

TAF9B

```
                              |---------------------------------------------------
Brachyteles_arachnoides       MESGKMAPPKNAPRDALVMAQILKDMGITEYEPRVINQMLEFAFRYVTTILDDAKIYSSH
Callithrix_jacchus            MESGKMAPPKNAPRDALVMAQILKDMGITEYEPRVINQMLEFAFRYVTTILDDAKIYSSH
Aotus_nancymaae               MESGKMAPLKNAPRDALVMAQILKDMGITEYEPRVINQMLEFAFRYVTTILDDAKMYSSH
Cebus_capucinus_imitator      MESGKMAPPKNAPRDALVMAQILKDMGITEYEPRVINQMLEFAFRYVTTILDDAKIYSSH
Saimiri_boliviensis           MESGKMAPPKNAPRDALVMAQILKDMGITEYEPRVINQMLEFAFRYVTTILDDAKIYSSH
Macaca_nemestrina             MESGKMAPPKNAPRDALVMAQILKDMGITEYEPRVINQMLEFAFRYVTTILDDAKIYSSH
Papio_anubis                  MESGKMASPKSMPKDAQMMAQILKDMGITEYEPRVINQMLEFAFRYVTTILDDAKIYSSH
Chlorocebus_sabaeus           MESGKMAPPKNAPRDALVMAQILKDMGITEYEPRVINQMLEFAFRYVTTILDDAKIYSSH
Homo_sapiens                  MESGKMAPPKNAPRDALVMAQILKDMGITEYEPRVINQMLEFAFRYVTTILDDAKIYSSH
Pongo_abelii                  MESGKMAPPKNAPRDALVMAQILKDMGITEYEPRVINQMLEFAFRYVTTILDDAKIYSSH
Otolemur_garnetti             MESGKMAPPKNAPRDALVMAQILKDMGITEYEPRVINQMLEFAFRYVTTILDDAKIYSSH
                              123456789012345678901234567890123456789012345678901234567890
                                       1         2         3         4         5         6

                              ---- Transcription initiation factor IID, 31kD subunit -----
Brachyteles_arachnoides       AKKPNVDADDVRLAIQCRADQSFTSPPPRDFLLDIARQKNQTPLPLIKPYAGPRLPPDRY
Callithrix_jacchus            AKKPNVDADDVRLAIQCRADQSFTSPPPRDFLLDIARQKNQTPLPLIKPYAGPRLPPDRY
Aotus_nancymaae               AKKPNVDADDVRLAIQCRADQSFTSPPPRDFLLDIARQKNQTPLPLIKPYAGPRLPPDRY
Cebus_capucinus_imitator      AKKPNVDADDVRLAIQCRADQSFTSPPPRDFLLDIARQKNQTPLPLIKPYAGPRLPPDRY
Saimiri_boliviensis           AKKPNVDADDVRLAIQCRADQSFTSPPPRDFLLDIARQKNQTPLPLIKPYAGPRLPPDRY
Macaca_nemestrina             AKKPNVDADDVRLAIQCRADQSFTSPPPRDFLLDIARQKNQTPLPLIKPYAGPRLPPDRY
Papio_anubis                  AKKATVDADDVRLAIQCRADQSFTSPPPRDFLLDIARQRNQTPLPLIKPYSGPRLPPDRY
Chlorocebus_sabaeus           AKKPNVDADDVRLAIQCRADQSFTSPPPRDFLLDIARQKNQTPLPLIKPYAGPRLPPDRY
Homo_sapiens                  AKKPNVDADDVRLAIQCRADQSFTSPPPRDFLLDIARQKNQTPLPLIKPYAGPRLPPDRY
Pongo_abelii                  AKKPNVDADDVRLAIQCRADQSFTSPPPRDFLLDIARQKNQTPLPLIKPYAGPRLPPDRY
Otolemur_garnetti             AKKPNVDADDVRLAIQCRADQSFTSPPPRDFLLDIARQKNQTPLPLIKPYAGPRLPPDRY
                              123456789012345678901234567890123456789012345678901234567890
                             61        7         8         9        100        1         2

                              ----------|
                                                                                       *
Brachyteles_arachnoides       CLTAPNYRLKSLIKKGP-NQGRL-VPRLSVGAVSSKPTTPTIA--TPQTVSVPNKVAAPM
Callithrix_jacchus            CLTAPNYRLKSLIKKGP-NQGRL-VPRLSVGAVSSKPTTSTIA--TPQTVSVPNKVATPM
Aotus_nancymaae               CLTAPNYRLKSLIKKGP-NQGRL-VPRLSVGAVSSKPTTPTIA--TPQTVSVPNKVATPM
Cebus_capucinus_imitator      CLTAPNYRLKSLIKKGP-NQGRL-VPRLSVGAVSSKPTTPTVA--TPQTVSVPNKVATPM
Saimiri_boliviensis           CLTAPNYRLKSLIKKGP-NQGRL-VPRLSVGAVSSKPTTPTIA--TPQTVSVANKVATPM
Macaca_nemestrina             CLTAPNYRLKSLIKKGP-NQGRL-VPRLSVGAVSSKPTTPTIA--TPQTVSVPNKVATPM
Papio_anubis                  CLTAPNYRLKSLQKKASTSAGRITVPRLSVGSVTSRPSTPTLGTPTPQTMSVSTKVGTPM
Chlorocebus_sabaeus           CLTAPNYRLKSLIKKGP-NQGRL-VPRLSVGAVSSKPTTPTIA--TPQTVSVPNKVATPM
Homo_sapiens                  CLTAPNYRLKSLIKKGP-NQGRL-VPRLSVGAVSSKPTTPTIA--TPQTVSVPNKVATPM
Pongo_abelii                  CLTAPNYRLKSLIKKGP-NQGRL-VPRLSVGAVSSKPTTPTIA--TPQTVSVPNKVATPM
Otolemur_garnetti             CLTAPNYRLKSLIKKGP-NQGRV-VPRLSVGAVSSRPTTPTIA--TPQTVSIPNKVATPV
                              12345678901234567-89012-3456789012345678901--234567890123456
                            121        3          4          5         6           7

                                                                                   **
Brachyteles_arachnoides       SVTSQRFTVQIPPSQSTPVK-PVPATTAVQNVLINPSMIGPKNILITTNMVSSQNILN
Callithrix_jacchus            SVTSQRFTVQIPPSQSTPVK-PVPATTAVQNVLINPSMIGPKNILITTNMVSSQNTAN
Aotus_nancymaae               SVTSQRFTVQIPPSQSTPVK-PVPATTAVQNVLINPSMIGPKNILITTNMVSSQNTAN
Cebus_capucinus_imitator      SVTSQRFTVQIPPSQSTPVK-PVPATTAVQNVLINPSMIGPKNILITTNMVSSQNTAN
Saimiri_boliviensis           SVTSQRFTVQIPPSQSTPVK-PGPATTAVQNVLINPSMIGPKNILITTNMVSSQNTAN
Macaca_nemestrina             SVTSQRFTVQIPPSQSTPVK-PVPATTAVQNVLINPSMIGPKNILITTNMVSSQNTAN
Papio_anubis                  SLTGQRFTVQMPTSQSPAVKASIPATSAVQNVLINPSLIGSKNILITTNMVSSQNAAN
Chlorocebus_sabaeus           SVTSQRFTVQIPPSQSTPVK-PVPATTAVQNVLINPSMIGPKNILITTNMVSSQNTAN
Homo_sapiens                  SVTSQRFTVQIPPSQSTPVK-PVPATTAVQNVLINPSMIGPKNILITTNMVSSQNTAN
Pongo_abelii                  SVTSQRFTVQIPPSQSTPVK-PVPATTAVQNVLINPSMIGPKNILITTNMVSSQNTAN
Otolemur_garnetti             SVASQRFTMQIPPSQTAPVK-PVPATTAVQNVLINPSMIGPKNILITTNMVSSQNMAN
                              7890123456789012345 6-7890123456789012345678901234567890123
                               180        9         200        1         2         3
```

UBXN2B

```
                                                                     *
Brachyteles_arachnoides   MAEGGGPEPGEKERRSSGPRPPSARDLQLALAELYEDEVKLKSSKSDRPKAAVFKSPRTP
Callithrix_jacchus        MAEGGGSEPGEQERRSSGPRPPSARDLQLALAELYEDEVKCKSSKPDRPKAAVFKSPRTP
Cebus_capucinus           MAEGGGPEPGEQERRSSGPRPPSARDLQLALAELYEDEVKCKSSKSDRPKAAVFKSPRTP
Saimiri_boliviensis       MAEGGGPEPGEQERRSSGPRPPSARDLQLALAELYEDEVKCKSSKSDRPKAAVFKSPRTP
Macaca_nemestrina         MAEGGGPDPSEKERRSSGPRPPSARDLQLALAELYEDEVKCKSSKSNRPKAAVFKSPRTP
Chlorocebus_sabaeus       MAEGGGPDPGEKERRSSGPRPPSARDLQLALAELYEDEVKCKSSKSNRPKAAVFKSPRTP
Homo_sapiens              MAEGGGPEPGEQERRSSGPRPPSARDLQLALAELYEDEVKCKSSKSNRPKATVFKSPRTP
Pongo_abelii              MAEGGGPEPGEQERRSSGPRPPSARDLQLALAELYEDEVKCKSSKSNRPKATVFKSPRTP
                          123456789012345678901234567890123456789012345678901234567890
                                   1         2         3         4         5         6

Brachyteles_arachnoides   PQRFYSSEHEYSGLHIVRPSTGKIVNELFKEAREHGAVPLNEATRASGDDKSKSFTGGGY
Callithrix_jacchus        PQRFYSSEHEYSGLHIVRPSTGKIVNELFKEAREHGAVPLNEATRASGDDKSKSFTGGGY
Cebus_capucinus           PQRFYSSEHEYSGLHIVRPSTGKIVNELFKEAREHGAVPLNEATRASGDDKSKSFTGGGY
Saimiri_boliviensis       PQRFYSREHEYSGLHIVRPSTGKIVNELFKEAREHGAVPLSEATRASGDDKSKSFTGGGY
Macaca_nemestrina         PQRFYSSEHEYSGLNIVRPSTGKIVNELFREAREHGAVPLNEATRASGDDKSKSFTGGGY
Chlorocebus_sabaeus       PQRFYSSEHEYSGLNIVRPSTGKIVNELFREAREHGAVPLNEATRASGDDKSKSFTGGGY
Homo_sapiens              PQRFYSSEHEYSGLNIVRPSTGKIVNELFKEAREHGAVPLNEATRASGDDKSKSFTGGGY
Pongo_abelii              PQRFYSSEHEYSGLNIVRPSTGKIVNELFKEAREHGAVPLNEATRASGDDKSKSFTGGGY
                          123456789012345678901234567890123456789012345678901234567890
                         61        7         8         9        100         1         2

                                              |--------------- SEP domain ------------
Brachyteles_arachnoides   RLGNSFCKRSEYIYGENQLQDVQILLKLWSNGFSLDDGELRPYNDPTNAQFLESVKRGEI
Callithrix_jacchus        RLGNSFCKRSEYIYGENQLQDVQVLLKLWSNGFSLDDGELRPYNDPTNAQFLESVKRGEI
Cebus_capucinus           RLGNSFCKRSEYIYGENQLQDVQILLKLWSNGFSLDDGELRPYNDPTNAQFLESVKRGEI
Saimiri_boliviensis       RLGNSFCKRSEYIYGENQLQDVQILLKLWSNGFSLDDGELRPYNDPTNAQFLESVKRGEI
Macaca_nemestrina         RLGNSFCKRSEYIYGENQLQDVQILLKLWSNGFSLDDGELRPYNEPTNAQFLESVKRGEI
Chlorocebus_sabaeus       RLGNSFCKRSEYIYGENQLQDVQILLKLWSNGFSLDDGELRPYNEPTNAQFLESVKRGEI
Homo_sapiens              RLGSSFCKRSEYIYGENQLQDVQILLKLWSNGFSLDDGELRPYNEPTNAQFLESVKRGEI
Pongo_abelii              RLGSSFCKRSEYIYGENQLQDVQILLKLWSNGFSLDDGELRPYNEPTNAQFLESVKRGEI
                          123456789012345678901234567890123456789012345678901234567890
                        121        3         4         5         6         7         8

                          ---------------------------------------|
Brachyteles_arachnoides   PLELQRLVHGGQVNLDMEDHQDQEYIKPRLRFKAFSGEGQKLGSLTPEIVSTPSSPEEED
Callithrix_jacchus        PLELQRLVHGGQVNLDMEDHQDQEYIKPRLRFKAFSGEGQKLGSLTPEIVSTPSSPEEED
Cebus_capucinus           PLELQRLVHGGQVNLDMEDHQDQEYIKPRLRFKAFSGEGQKLGSLTPEIVSTPSSPEEED
Saimiri_boliviensis       PLELQRLVHGGQVNLDMEDHQDQEYIKPRLRFKAFSGEGQKLGSLTPEIVSTPSSPEEED
Macaca_nemestrina         PLELQRLVHGGQVNLDMEDHQDQEYIKPRLRFKAFSGEGQKLGSLTPEIVSTPSSPEEED
Chlorocebus_sabaeus       PLELQRLVHGGQVNLDMEDHQDQEYIKPRLRFKAFSGEGQKLGSLTPEIVSTPSSPEEED
Homo_sapiens              PLELQRLVHGGQVNLDMEDHQDQEYIKPRLRFKAFSGEGQKLGSLTPEIVSTPSSPEEED
Pongo_abelii              PLELQRLVHGGQVNLDMEDHQDQEYIKPRLRFKAFSGEGQKLGSLTPEIVSTPSSPEEED
                          123456789012345678901234567890123456789012345678901234567890
                        181        9        200        1         2         3         4

                                    |----------------- UBX domain ------------------
Brachyteles_arachnoides   KSILNAVVLIDDSVPTTKIQIRLADGSRLIQRFNSTHRILDVRNFIIQSRPEFATLDFIL
Callithrix_jacchus        KSILNAVVLIDDSVPTTKIQIRLADGSRLIQRFNSTHRILDVRNFIVQSRPEFATLDFIL
Cebus_capucinus           KSILNAVVLIDDSIPTTKIQIRLADGSRLIQRFNSTHRILDVRNFIVQSRPEFATLDFIL
Saimiri_boliviensis       KSILNAVVLIDDSVPTTKIQIRLADGSRLIQRFNSTHRILDVRNFIVQSRPEFATLDFIL
Macaca_nemestrina         KSILNAVVLIDDSVPTTKIQIRLADGSRLIQRFNSTHRILDVRNFIVQSRPEFAALDFIL
Chlorocebus_sabaeus       KSILNAVVLIDDSVPTTKIQIRLADGSRLIQRFNSTHRILDVRNFIVQSRPEFAALDFIL
Homo_sapiens              KSILNAVVLIDDSVPTTKIQIRLADGSRLIQRFNSTHRILDVRNFIVQSRPEFAALDFIL
Pongo_abelii              KSILNAVVLIDDSVPTTKIQIRLADGSRLIQRFNSTHRILDVRNFIVQSRPEFAALDFIL
                          123456789012345678901234567890123456789012345678901234567890
                        241        5         6         7         8         9        300

                          -----------------------------|
Brachyteles_arachnoides   VTSFPNKELTDESLTLQEADILNTVLLQQLK
Callithrix_jacchus        VTSFPNKELTDESKTLQEADILNTVLVQQLK
Cebus_capucinus           VTSFPNKELTDESLTLQEADILNTVLLQQLK
Saimiri_boliviensis       VTSFPNKELTDESLTLQEADILNTVLLQQLK
Macaca_nemestrina         VTSFPNKELTDESLTLLEADILNTVLLQQLK
Chlorocebus_sabaeus       VTSFPNKELTDESLTLQEADILNTVLLQQLK
Homo_sapiens              VTSFPNKELTDESLTLLEADILNTVLLQQLK
Pongo_abelii              VTSFPNKELTDESLTLLEADILNTVLLQQLK
                          1234567890123456789012345678901
                        301        1         2         3
```

UHRF1BP1L

```
                          |-------- N-terminal region of Chorein or VPS13 ----------
Brachyteles_arachnoides   MAGIIKKQILKHLSRFTKNLSPDKINLSTLKGEGELKNLELDEEVLQNMLDLPTWLAINK
Callithrix_jacchus        MAGIIKKQILKHLSRFTKNLSPDKINLSTLKGEGELKNLELDEEVLQNMLDLPTWLAINK
Aotus_nancymaae           MAGIIKKQILKHLSRFTKNLSPDKINLSTLKGEGELKNLELDEEVLQNMLDLPTWLAINK
Saimiri_boliviensis       MAGIIKKQILKHLSRFTKNLSPDKINLSTLKGEGELKNLELDEEVLQNMLDLPTWLAINK
Macaca_nemestrina          MAGIIKKQILKHLSRFTKNLSPDKINLSTLKGEGELKNLELDEEVLQNMLDLPTWLAINK
Chlorocebus_sabaeus       MAGIIKKQILKHLSRFTKNLSPDKINLSTLKGEGELKNLELDEEVLQNMLDLPTWLAINK
Homo_sapiens              MAGIIKKQILKHLSRFTKNLSPDKINLSTLKGEGELKNLELDEEVLQNMLDLPTWLAINK
Pongo_abelii              MAGIIKKQILKHLSRFTKNLSPDKINLSTLKGEGELKNLELDEEVLQNMLDLPTWLAINK
Otolemur_garnetti         MAGIIKKQILKHLSRFTKNLSPDKINLSTLKGEGELKNLELDEEVLQNMLDLPTWLAINK
                          123456789012345678901234567890123456789012345678901234567890
                                   1         2         3         4         5         6

                          ---------------------------------|
Brachyteles_arachnoides   VFCNKASIRIPWTKLKTHPICLSLDKVIMEMSTCEEPRSPNGPSPIATASGQSEYGFAEK
Callithrix_jacchus        VFCNKASIRIPWTKLKTHPICLSLDKVIMEMSTCEEPRIPNGPSPIATASGQSEYGFAEK
Aotus_nancymaae           VFCNKASIRIPWTKLKTHPICLSLDKVIMEMSTCEEPRSPNGPSPIATASGQSEYGFAEK
Saimiri_boliviensis       VFCNKASIRIPWTKLKTHPICLSLDKVIMEMSTCEEPRSPNGPSPIATASGQSEYGFAEK
Macaca_nemestrina         VFCNKASIRIPWTKLKTHPICLSLDKVIMEMSTCEEPRSPNGPSPIATASGQSEYSFAEK
Chlorocebus_sabaeus       VFCNKASIRIPWTKLKTHPICLSLDKVIMEMSTCEEPRSPNGPSPIATASGQSEYSFAEK
Homo_sapiens              VFCNKASIRIPWTKLKTHPICLSLDKVIMEMSTCEEPRSPNGPSPIATASGQSEYGFAEK
Pongo_abelii              VFCNKASIRIPWTKLKTHPICLSLDKVIMEMSTCEEPRSPNGPSPIATASGQSEYGFAEK
Otolemur_garnetti         VFCNKASIRIPWTKLKTQPICLSLDKVIMEMSTCEEPRPPNGPSPIATASGQSEYGFAEK
                          123456789012345678901234567890123456789012345678901234567890
                         61        7         8         9        100        1         2

Brachyteles_arachnoides   VVEGITVSVNSIVIRIGAKAFNASFELSQLRIYSVNAHWEHGDLRFTRIQDPQRGEVLTF
Callithrix_jacchus        VVEGITVSVNSIVIRIGAKAFNASFELSQLRIYSVNAHWEHGDLRFTRIQDPQRGEVLTF
Aotus_nancymaae           VVEGITVSVNSIVIRIGAKAFNASFELSQLRIYSVNAHWEHGDLRFTRIQDPQRGEVLTF
Saimiri_boliviensis       VVEGITVSVNSIVIRIGAKAFNASFELSQLRIYSVNAHWEHGDLRFTRIQDPQRGEVLTF
Macaca_nemestrina         VVEGITVSVNSIVIRIGAKAFNASFELSQLRIYSVNAHWEHGDLRFTRIQDPQRGEVLTF
Chlorocebus_sabaeus       VVEGITVSVNSIVIRIGAKAFNASFELSQLRIYSVNAHWEHGDLRFTRIQDPQRGEVLTF
Homo_sapiens              VVEGISVSVNSIVIRIGAKAFNASFELSQLRIYSVNAHWEHGDLRFTRIQDPQRGEVLTF
Pongo_abelii              VVEGISVSVNSIVIRIGAKAFNASFELSQLRIYSVNAHWEHGDLRFTRIQDPQRGEVLTF
Otolemur_garnetti         VVEGITVSVNSIVIRIGAKAFNASFELSQLRIYSVNAKWEHGDLRFTRIQDPQRGEVLTF
                          123456789012345678901234567890123456789012345678901234567890
                        121        3         4         5         6         7         8

Brachyteles_arachnoides   KEINWQMIRIEADATQSSHLEIMCAPVRLITNQSKIRVTLKRRLKDCNVIATKLVLILDD
Callithrix_jacchus        KEINWQMIRIEADATQSSHLEIMCAPVRLITNQSKIRVTLKRRLKDCNVIATKLVLILDD
Aotus_nancymaae           KEINWQMIRIEADATQSSHLEIMCAPVRLITNQSKIRVTLKRRLKDCNVIATKLVLILDD
Saimiri_boliviensis       KEINWQMIRIEADATQSSHLEIMCAPVRLITNQSKIRVTLKRRLKDCNVIATKLVLILDD
Macaca_nemestrina         KEINWQMIRIEADATQSSHLEIMCAPVRLITNQSKIRVTLKRRLKDCNVIATKLVLILDD
Chlorocebus_sabaeus       KEINWQMIRIEADATQSSHLEIMCAPVRLITNQSKIRVTLKRRLKDCNVIATKLVLILDD
Homo_sapiens              KEINWQMIRIEADATQSSHLEIMCAPVRLITNQSKIRVTLKRRLKDCNVIATKLVLILDD
Pongo_abelii              KEINWQMIRIEADATQSSHLEIMCAPVRLITNQSKIRVTLKRRLKDCNVIATKLVLILDD
Otolemur_garnetti         KEINWQMIRIEADATQSSHLEIMCAPVRLITNQSKIRVTLKRRLKDCNVIATKLVLTLDD
                          123456789012345678901234567890123456789012345678901234567890
                        181        9        200        1         2         3         4

Brachyteles_arachnoides   LLWVLTDSQLKAMVQYAKSLSEAIEKSTEQRKSMAPEPTQSSTVVTSAQQVKTTQTSNAP
Callithrix_jacchus        LLWVLTDSQLKAMVQYAKSLSEAIEKSTEQRKSMAPEPTQSSTVVASAQQVKTTQTSNAP
Aotus_nancymaae           LLWVLTDSQLKAMVQYAKSLSEAIEKSTEQRKSMAPEPTQSSTVVASAQEVKTTQTSNAP
Saimiri_boliviensis       LLWVLTDSQLKAMVQYAKSLSEAIEKSTEQRKSMAPEPTQSSTVVASAQQVKTTQTSNAP
Macaca_nemestrina         LLWVLTDSQLKAMVQYAKSLSEAIEKSTEQRKSMAPEPTQSSTVVASAQQVKTTQTSNAP
Chlorocebus_sabaeus       LLWVLTDSQLKAMVQYAKSLSEAIEKSTEQRKSMAPEPTQSSTVVASAQQVKTTQTSNAP
Homo_sapiens              LLWVLTDSQLKAMVQYAKSLSEAIEKSTEQRKSMAPEPTQSSTVVASAQQVKTTQTSNAP
Pongo_abelii              LLWVLTDSQLKAMVQYAKSLSEAIEKSTEQRKSMAPEPTQSSTVVASAQQVKTTQTSNAP
Otolemur_garnetti         LLWVLTDSQLKAMVQYAKSLSEAIEKSTEQRKSMAPEPTQSSTVVPSTQQVKTPQTSNVP
                          123456789012345678901234567890123456789012345678901234567890
                        241        5         6         7         8         9       300
```

```
Brachyteles_arachnoides   DVNDAIVKLFNDFDVKETSHHLVISHLDLHICDDIHAKEKESNRRITGGAMQLSFTQLTI
Callithrix_jacchus        DVNDAIVKLFNDFDVKETSHHLVISHLDLHICDDIHAKEKESNRRITGGAMQLSFTQLTI
Aotus_nancymaae           DVNDAIVKLFNDFDVKETSHHLVISHLDLHICDDIHAKEKESNRRITGGAMQLSFAQLTI
Saimiri_boliviensis       DVNDAIVKLFNDFDVKETSHHLVISHLDLHICDDIHAKEKESNRRITGGAMQLSFTQLTI
Macaca_nemestrina         DVNDAIVKLFNDFDVKETSHHLVISHLDLHICDDIHAKEKESNRRITGGAMQLSFTQLTI
Chlorocebus_sabaeus       DVNDAIVKLFNDFDVKETSHHLVISHLDLHICDDIHAKEKESNRRITGGAMQLSFTQLTI
Homo_sapiens              DVNDAIVKLFNDFDVKETSHHLVISHLDLHICDDIHAKEKESNRRITGGAMQLSFTQLTI
Pongo_abelii              DVNDAIVKLFNDFDVKETSHHLVISHLDLHICDDIHAKEKESNRRITGGAMQLSFTQLTI
Otolemur_garnetti         DLSDAIVKLFSDFDVKETSHHLVISHLDLHICDDIHNKEKESNRRITGGAMQLSFTQLTI
                          123456789012345678901234567890123456789012345678901234567890
                        301         1         2         3         4         5         6

Brachyteles_arachnoides   DYYPYHKAGDSCKHWMYFSDATKTKNGWANELLHEFECNVEMLKQAVKDHNVGSPPKSPT
Callithrix_jacchus        DYYPYHKAGDSCNHWMYFSDATKTKNGWANELLHEFECNVEMLKQAVKDHNVGSPPKSPT
Aotus_nancymaae           DYYPYHKAGDSCNHWMYFSDATKAKNGWANELLHEFECNVEMLKQAVKDHNVGSPPKSPT
Saimiri_boliviensis       DYYPYHKAGDSCNHWMYFSDATKTKNGWANELLHEFECNVEMLKQAVKDHNVGSPPKSPT
Macaca_nemestrina         DYYPYHKAGDSCNHWMYFSDATKTKNGWANELLHEFECNVEMLKQAVKDHNVGSPPKSPT
Chlorocebus_sabaeus       DYYPYHKAGDSCNHWMYFSDATKTKNGWANELLHEFECNVEMLKQAVKDHNVGSPPKSPT
Homo_sapiens              DYYPYHKAGDSCNHWMYFSDATKTKNGWANELLHEFECNVEMLKQAVKDHNVGSPPKSPT
Pongo_abelii              DYYPYHKAGDSCNHWMYFSDATKTKNGWANELLHEFECNVEMLKQAVKDHNVGSPPKSPT
Otolemur_garnetti         DYYPYHKAGDSCNHWMYFSDATKTKNGWANELLHEFEYNVEMLKQAVKDHNVGSPPTSPT
                          123456789012345678901234567890123456789012345678901234567890
                        361         7         8         9        400        1         2

Brachyteles_arachnoides   HASPQHTQTEKDYTLKGTCRTPSVLSQQSKAKLMSSSVVVRLADFNIYQVSTAEQCRSSP
Callithrix_jacchus        HASPQHTQTEKDYTLKGTCRTPSVLSQQSKAKLMSSSVVVRLADFNIYQVSTAEQCRSSP
Aotus_nancymaae           HASPQHTQTEKDYTLKGTCRTPSVLSQQSKAKLMSSSVVVRLADFNIYQVSTAEQCRSSP
Saimiri_boliviensis       HASPQHTQTEKDYTLKGTCRTPSVLSQQSKAKLMSSSVVVRLADFNIYQVSTAEQCRSSP
Macaca_nemestrina         HASPQHTQTEKDYPLKGTCRTPSVLSQQSKAKLMSSSVVVRLADFNIYQVSTAEQCRSSP
Chlorocebus_sabaeus       HASPQHTQTEKDYPLKGTCRTPSVLSQQSKAKLMSSSVVVRLADFNIYQVSTAEQCRSSP
Homo_sapiens              HASPQHTQTEKDYPLKGTCRTPSVLSQQSKAKLMSSSVVVRLADFNIYQVSTAEQCRSSP
Pongo_abelii              HASPQHTQTEKDYPLKGTCRTPSVLSQQSKAKLMSSSVVVRLADFNIYQVSTAEQCRSSP
Otolemur_garnetti         HASPQHTQTEKEPPLKGSSRTPSVLSEQSKARLMSSSVVVRLADFNIYQVSTAEQCRSSP
                          123456789012345678901234567890123456789012345678901234567890
                        421         3         4         5         6         7         8

Brachyteles_arachnoides   KSMICCNKKSLYLPQEMSAVYIEFTEYYYPDGKDFPIPSPNLYSQLNALQFTVDERSILW
Callithrix_jacchus        KSMICCNKKSLYLPQEMSAVYIEFTEYYYPDGKDFPIPSPNLYSQLNALQFTVDERSILW
Aotus_nancymaae           KSMICCNKKSLYLPQEMSAVYIEFTEYYYPDGKDFPIPSPNLYSQLNALQFTVDERSILW
Saimiri_boliviensis       KSMICCNKKSLYLPQEMSAVYIEFTEYYYPDGKDFPIPSPNLYSQLNALQFTVDERSILW
Macaca_nemestrina         KSMICCNKKSLYLPQEMSAVYIEFTEYYYPDGKDFPIPSPNLYSQLNALQFTVDERSILW
Chlorocebus_sabaeus       KSMICCNKKSLYLPQEMSAVYIEFTEYYYPDGKDFPIPSPNLYSQLNALQFTVDERSILW
Homo_sapiens              KSMICCNKKSLYLPQEMSAVYIEFTEYYYPDGKDFPIPSPNLYSQLNALQFTVDERSILW
Pongo_abelii              KSMICCNKKSLYLPQEMSAVYIEFTEYYYPDGKDFPIPSPNLYSQLNALQFTVDERSILW
Otolemur_garnetti         KSMISCNKKTLFLPQEMSAVYIEFTEYYYPDGKDFPIPSSNLYSQLNALQFTVDERSILW
                          123456789012345678901234567890123456789012345678901234567890
                        481         9        500        1         2         3         4

Brachyteles_arachnoides   LNQFLLDLKQSLNQFMAVYKLNDNSKSDEHVDVRVDGLMLKFVIPSEMKSECHQDQPRAI
Callithrix_jacchus        LNQFLLDLKQSLNQFMAVYKLNDNSKSDEHVDVRVDGLMLKFVIPSEMKSECHQDQPRAI
Aotus_nancymaae           LNQFLLDLKQSLNQFMAVYKLNDNSKSDEHVDVRVDGLMLKFVIPSEMKSECHQDQPRAI
Saimiri_boliviensis       LNQFLLDLKQSLNQFMAVYKLNDNSKSDEHVDVRVDGLMLKFVIPSEMKSECHQDQPRAI
Macaca_nemestrina         LNQFLLDLKQSLNQFMAVYKLNDNSKSDEHVDVRVDGLMLKFVIPSEMKSECHQDQPHAI
Chlorocebus_sabaeus       LNQFLLDLKQSLNQFMAVYKLNDNSKSDEHVDVRVDGLMLKFVIPSEMKSECHQDQPHAI
Homo_sapiens              LNQFLLDLKQSLNQFMAVYKLNDNSKSDEHVDVRVDGLMLKFVIPSEVKSECHQDQPRAI
Pongo_abelii              LNQFLLDLKQSLNQFMAVYKLNDNSKSDEHVDVRVDGLMLKFVIPSEVKSECHQDQPRAI
Otolemur_garnetti         LNQFLLDLKQSLNQFMTVYKLNENSKSDEHVDVRVDGLMLKLVIPSEMKSECHGDQPRAI
                          123456789012345678901234567890123456789012345678901234567890
                        541         5         6         7         8         9       600
```

```
Brachyteles_arachnoides   SIQSSEMIATNTRHCPNCRHSDLEALFQDFKDCDFFSKTYTSFPKSCDNFNLLHPIFQRH
Callithrix_jacchus        SIQSSEMIATNTRHCPHCRHSDLEALFQDFKDCDFFSKTYTSFPKSCDSFNLLHPIFQRH
Aotus_nancymaae           SIQSSEMIATNTRHCPNCRHSDLEALFQDFKDCDFFSKTYTSFPKSCDSFNLLHPIFQRH
Saimiri_boliviensis       SIQSSEMIATNTRHCPNCRHSDLEALFQDFKDCDFFSKTYTSFPKSCDSFNLLHPIFQRH
Macaca_nemestrina         SIQSSEMIATNTRHCPNCRHSDLEALFQDFKDCDFFSKTYTSFPKSCDNFNLLHPIFQRH
Chlorocebus_sabaeus       SIQSSEMIATNTRHCPNCRHSDLEALFQDFKDCDFFSKTYTSFPKSCDNFNLLHPIFQRH
Homo_sapiens              SIQSSEMIATNTRHCPNCRHSDLEALFQDFKDCDFFSKTYTSFPKSCDNFNLLHPIFQRH
Pongo_abelii              SIQSSEMIATNTRHCPNCRHSDLEALFQDFKDCDFFSKTYTSFPKSCDNFNLLHPIFQRH
Otolemur_garnetti         SIQSSEMIATNTRHCPNCRHSDLEALFQDFKDCDFFSKTYTSFPKSRDNFNLLHPIFQRH
                          123456789012345678901234567890123456789012345678901234567890
                        601         1         2         3         4         5         6

                                                          *
Brachyteles_arachnoides   AHEQDTKMHEVYKGNITPQLNKNTLKTSAAMDIWAVYFSQFWIDYEGMKSGKGRPISFVD
Callithrix_jacchus        AHEQDTKMHEVYKGNITPQLNKNTLKTSAAMDVWAVYFSQFWIDYEGMKSGKGRPISFID
Aotus_nancymaae           AHEQDTKMHEVYKGNITPQLSKNTLKTSAAMDVWAVYFSQFWIDYEGMKSGKGRPISFVD
Saimiri_boliviensis       AHEQDTKMHEVYKGNITPQLNKNTLKTSAAMDVWAVYFSQFWIDYEGMKSGKGRPISFVD
Macaca_nemestrina         AHEQDTKMHEIYKGNITPQLNKNTLKTSAAMDVWAVYFSQFWIDYEGMKSGKGRPISFVD
Chlorocebus_sabaeus       AHEQDTKMHEIYKGNITPQLNKNTLKTSAATDVWAVYFSQFWIDYEGMKSGKGRPISFVD
Homo_sapiens              AHEQDTKMHEIYKGNITPQLNKNTLKTSAATDVWAVYFSQFWIDYEGMKSGKGRPISFVD
Pongo_abelii              AHEQDTKMHEIYKGNITPQLNKNTLKTSAATDVWAVYFSQFWIDYEGMKSGKGRPISFVD
Otolemur_garnetti         AHEQDTKMHEVYKGNIIPKLNKNTLKTSAAMDVWAVYFSQFWIDYEGMKSGKERPINFVD
                          123456789012345678901234567890123456789012345678901234567890
                        661         7         8         9        700        1         2

Brachyteles_arachnoides   SFPLSIWICQPTRYAESQKEPQTWSQVSLNTSQSESSDLAGRLKRKKLLKEYYSTESEPL
Callithrix_jacchus        SFPLSIWICQPTRYAESQKEPQSCNQVSLNTSQSESSDLAGRLKRKKLLKEYYSTESEPL
Aotus_nancymaae           SFPLSIWICQPTRYAESQKEPQTCSQLSLNTSQSESSDLAGRLKRKKLLKEYYSTESEPL
Saimiri_boliviensis       SFPLSIWICQPTRYAESQKEPQTCSQVSLNTSHSESSDLAGRLKRKKLLKEYYSTESEPL
Macaca_nemestrina         SFPLSIWICQPTRYAESQKEPQSCNQVSLNTSQSESSDLAGRLKRKKLLKEYYSTESEPL
Chlorocebus_sabaeus       SFPLSIWICQPTRYAESQKEPQSCNQVSLNTSQSESSDLAGRLKRKKLLKEYYSTESEPL
Homo_sapiens              SFPLSIWICQPTRYAESQKEPQTCNQVSLNTSQSESSDLAGRLKRKKLLKEYYSTESEPL
Pongo_abelii              SFPLSIWICQPTRYAESQKEPQTCNQVSLNTSQSESSDLAGRLKRKKLLKEYYSTESEPL
Otolemur_garnetti         SFPLSIWICQPTRYTESQKELQTCDQVSLNTSQSESSDLAGRLKRKKLLKEYYSTESEPL
                          123456789012345678901234567890123456789012345678901234567890
                        721         3         4         5         6         7         8

                                        *                                  *
Brachyteles_arachnoides   TNGGQKP-SSDTFFTFSSSSSEADIHVLVRVHKHVSMQINHYQYLLLLFVHESLVLLSEN
Callithrix_jacchus        TNGGQKP-LSDTFFRFSSSSSEADIHVLVHVHKHVSIQINHYQYLLLLFLHESLILLSEN
Aotus_nancymaae           TNGGQKP-SSDTFFRLSSSSSEADIHVLVHVHKHVSMQINHYQYLLLLFLHESLILLSEN
Saimiri_boliviensis       TNGGQKP-SSDTFFRFSPSSSEADIHVLVHVHKHVSMQINHYQYLLLIFLHESLILLSEN
Macaca_nemestrina         ANGGQKPSSSDTFFRFSSSSSEADIHVLVHVHKHVSMQINHYQYLLLLLLHESLILLSEN
Chlorocebus_sabaeus       ANGGQKP-SSDKFFRFSSSSSEADIHVLVHVHKHVSMQINHYQYLLLLLLHESLILLSEN
Homo_sapiens              TNGGQKP-SSDTFFRFSPSSSEADIHLLVHVHKHVSMQINHYQYLLLLFLHESLILLSEN
Pongo_abelii              TNGGQKPSSSDTFFRFSSSSSEADIHVLVHVHKHVSMQINHYQYLLLLFLHESLILLSEN
Otolemur_garnetti         TNGGQNP-LADTFLRFSSSSSEADIHVLVHVHKHVSLQINHYQYLLLLFLHDSLVLLAEN
                          1234567-89012345678901234567890123456789012345678901234567890
                        781          9        800          1         2         3

Brachyteles_arachnoides   LRKDVEAVTGSPASQTSICIGILLRSAELALLLHPVDQANTLKSPVSESVSPVVPDYLPT
Callithrix_jacchus        LRKDVEAVTGSPASQTSICIGILLRSAELALLLHPVDQANTLKSPVSESVSPVVPDYLPT
Aotus_nancymaae           LRKDVEAVTGSPASQTSICIGILLRSAELALLLHPVDQANTLKSPVSESVSPVVPDYLST
Saimiri_boliviensis       LRKDVEAVTGSPASQTSICIGILLRSAELALLLHPVDQANTLKSPVSESVSPVVLDYLST
Macaca_nemestrina         LRKDVEAVTGSPASQTSICIGILLRSAELALLLHPVDQANTLKSPVSESVSPVVPDYLPT
Chlorocebus_sabaeus       LRKDVEAVTGSPASQTSICIGILLRSAELALLLHPVDQANTLKSPVSESVSPVVPDYLPT
Homo_sapiens              LRKDVEAVTGSPASQTSICIGILLRSAELALLLHPVDQANTLKSPVSESVSPVVPDYLPT
Pongo_abelii              LRKDVEAVTGSPASQTSICIGILLRSAELALLLHPVDQANTFKSPVSESVSPVVPDYMPT
Otolemur_garnetti         LRNDVEAVTGNPANQTSVCIGFLLRSAEVALLLHPVDQPNRIKSPVSESVSPVVPDYLPT
                          012345678901234567890123456789012345678901234567890123456789
                        840          5         6         7         8         9
```

```
                                                 *
Brachyteles_arachnoides  ENGDFLSSK---ISRDVN--RSVTANHMSDNRSMSVDLSHVPLKDPLLFKSASDTNLQKG
Callithrix_jacchus       ENGDFLSSK---TSRDVN--RSITVNYMSDNRSMSVDLSHVSLKDPLLFKSASDTNLQKG
Aotus_nancymaae          ENGDFLSSKTKQISRDVNRIRSVTVNHMSDNRSMSVDLSHVPLKDPLLFKSASDTNLQKG
Saimiri_boliviensis      ENGDFLSSK---ISRDIN--RSVTVNHMSDNRSMSVDLSHVPLKDPLLFKSASDTNLQKG
Macaca_nemestrina        ENGDFLSSKRKQISRDINRIRSVTVNHMSDNRSMSVDLSHVPLKDPLLFKSASDTNLQKG
Chlorocebus_sabaeus      EDGDFLSSKRKQISRDINRIRSVTVNHMSDNRSMSVDLSHVPLKDTLLFKSASDTNLQKG
Homo_sapiens             ENGDFLSSK---ISRDIN--RSVTVNHMSDNRSMSVDLSHIPLKDPLLFKSASDTNLQKG
Pongo_abelii             ENGDFLSSKRKQISRDINRIRSVTVNHMSDNRSMSVDLSHVPLKDPLLFKSASDTNLQKG
Otolemur_garnetti        KNGEVLSSERKEVS-DTNRIRSETVNHMSDNRSMSVDLSHVPLKDPLLFKSASDTNLQKG
                         012345678---90-123--4567890123456789012345678901234567890123
                       900            1            2         3         4         5
```

```
Brachyteles_arachnoides  ISFMDYLSDKHLGKISEDESSGLVYKSGSGEMGSETSDKKDSFYTD-SSILNYREDSNML
Callithrix_jacchus       ISFMDYLSDKHLGKISEDESSGLVYKSSSGEMGSEISDKKDSFYTD-SSILNYRDDSNML
Aotus_nancymaae          ISFMDYLSDKHLGKISEDESSGLVYKSGSGEMGSETSDKKDSFYTDSSSILNYREDSNML
Saimiri_boliviensis      ISFMDYLSDKHLGKISEDESSGLVYKSGSGEMGSETSDKKDSFYTD-SSILNYREDLNML
Macaca_nemestrina        ISFMDYLSDKHLGKISEDESSGLVYKSGSGEIGSETSDKKDSFYTDSSSILNYREDSNML
Chlorocebus_sabaeus      VSFMDYLSDKHLGKISEDESSGLVYKSGSGEIGSETSDKKDSFYTDSSSILNYREDSNML
Homo_sapiens             ISFMDYLSDKHLGKISEDESSGLVYKSGSGEIGSETSDKKDSFYTD-SSILNYREDSNIL
Pongo_abelii             ISFMDYLSDKHLGKISEDESSGLVYKSGSGEIGSETSDKKDSFYTDSSSILNYREDSNML
Otolemur_garnetti        ISFLDYLSDKHLGKISEDESSGLVYKSGSGEIGAGVSDKKNS*------------DSNIP
                         456789012345678901234567890123456789012345-------------67890
                       954     6         7         8         9                   1000
```

```
                                   *            *
Brachyteles_arachnoides  SFDSDGNQNIISNSLTSKGNETIQSIFKVEDLLPEAASLSENLEISKEETHAVRTLKSQS
Callithrix_jacchus       SFDNDGNQNILSNSLTSKGNETIESIFKAEDLLPETASLSENVEISKEETPAVRTLKSQL
Aotus_nancymaae          SFDSDGNQNILSNSLTGKGNETIELIFKAEDLLTEAASLSENLEISKEETPAVRTLKSQS
Saimiri_boliviensis      SFDSDGNQNILSNSLTSKGNETIESIFKAEDLLPEAASLSENLEISKEETPAVRTFKSQS
Macaca_nemestrina        SFDSDGNQNILSSSLTSKGNETIESIFKAEDLLSEAASLSENLDISKEETPTVRTLKSQS
Chlorocebus_sabaeus      SFDSDGNQNILSSSLTSKGNETIESIFKAEDLLPEAASLSENLDISKEETPTVRTLKSQS
Homo_sapiens             SFDSDGNQNILSSTLTSKGNETIESIFKAEDLLPEAASLSENLDISKEETPPVRTLKSQS
Pongo_abelii             SFDSDGNQNILSSSLTSKGNETIESIFKAEDLLPEAASLSENLDISKEETPPVRTLKSQS
Otolemur_garnetti        SVGNDGNQNILPSSLTSKGNEAIESIFKAEDLLPEAALVSENLETSKEEAPTLRTLKSQS
                         123456789012345678901234567890123456789012345678901234567890
                      1001        1         2         3         4         5         6
```

```
                                                                       *
Brachyteles_arachnoides  SLSGKPKERYPPNVAPLCVSYKNMKRSSSQMSLDTISLDSMILEEQVLESDGSDSHMFLE
Callithrix_jacchus       SLSGKPRERYPPNVAPLCVSYKNMKRSSSQMSLDTISLDSMILEEQLLESDGSDSHMFLE
Aotus_nancymaae          SLSGKPKERYPPNVAPLCVSYKNMKRSSSQMSLDTISLDSMILEEQLLESDGSDSHMFLE
Saimiri_boliviensis      SLSGKPKERYPPNVAPLCVSYKNMKRSSSQMSLDTISLDSMILEEQLLESDGSDSHMFLE
Macaca_nemestrina        SLSGKPKERCPPNLAPLCVSYKNMKRSSSQMSLDTISLDSMILEEQLLESDGSDSHMFLE
Chlorocebus_sabaeus      SLSGKPKERCPPNLAPLCVSYKNMKRSSSQMSLDTISLDSMILEEQLLESDGSDSHTFLE
Homo_sapiens             SLSGKPKERCPPNLAPLCVSYKNMKRSSSQMSLDTISLDSMILEEQLLESDGSDSHMFLE
Pongo_abelii             SLSGKPKERCPPNLAPLCVSYKNMKRSSSQMSLDTISLDSMILEEQLLESDGSDSHMFLE
Otolemur_garnetti        SLSGKPKERCPPNQAPPCISYKTMKRSSSQISLDTVSLDSMMLEEMLLESDGSDSHVFLE
                         123456789012345678901234567890123456789012345678901234567890
                      1061        7         8         9      1100         1         2
```

```
Brachyteles_arachnoides  KGNKKNSTTNYQSTAESVNASANLQNYGETSPDAISTNSEGAQENHDDLMSVVVFKITGV
Callithrix_jacchus       KGNKKNSTTNYQSTAESVNASANLQNYGETSPDAISTNSEGAQENHDDLMSVVVFKITGV
Aotus_nancymaae          KGNKKNSTTNYQSAAESVNASANLQNYGETSPDAISTNSEGAQENHDDLMSVVVFKITGV
Saimiri_boliviensis      KGNKKNSTTNYQSTAESVTAGANLQNYGETSPDAISTNSEGAQENHDDLMSVVVFKITGV
Macaca_nemestrina        KGNKKNSTTNYRGTAESVNTGANLQNYGETSPDAISTNSEGVQENHDDLMSVVVFKITGV
Chlorocebus_sabaeus      KGNKKNSTTNYRGTAESVNTGANLQNYGETSPDAISTNSEGVQENHDDLMSVVVFKITGV
Homo_sapiens             KGNKKNSTTNYRGTAESVNAGANLQNYGETSPDAISTNSEGAQENHDDLMSVVVFKITGV
Pongo_abelii             KGNKKNSTTNYRGTAESVNAGANLQNYGETSPDAISTNSEGAQENHDDLMSVVVFKITGV
Otolemur_garnetti        KGNKKNSTANYQSLAESENASTSLQNYGETSPDAISTNSEGAQENHDDLMSVVVFKITGV
                         123456789012345678901234567890123456789012345678901234567890
                      1121        3         4         5         6         7         8
```

```
                           *
Brachyteles_arachnoides  NGAIDIRGEDTEICLQVNQVTPDQLGNISLRHYLCNRPVGSDQKAVIHSKSSPEISLRFE
Callithrix_jacchus       NGEIDIRGEDTEICLQVNQVTPDQLGNISLRHYLCNRPVGSDQKAVIHSKSSPEISLRFE
Aotus_nancymaae          NGEIDIRGEDTEICLQVNQVTPDQLGNISLRHYLCNRPVGSDQKAVIHSKSSPEISLRFE
Saimiri_boliviensis      NGEIDIRGEDTEICLQVNQVTPDQLGNISLRHYLCNRPVGSDQKAVIHSKSSPEISLRFE
Macaca_nemestrina        NGEIDIRGEDTEICLQVNQVTPDQLGNISLRHYLCNRPVGSDQKAVIHSKSSPEISLRFE
Chlorocebus_sabaeus      NGEIDIRGEDTEICLQVNQVTPDQLGNISLRHYLCNRPVGSDQKAVIHSKSSPEISLRFE
Homo_sapiens             NGEIDIRGEDTEICLQVNQVTPDQLGNISLRHYLCNRPVGSDQKAVIHSKSSPEISLRFE
Pongo_abelii             NGEIDIRGEDTEICLQVNQVTPDQLGNISLRHYLCNRPVGSDQKAVIHSKSSPEISLRFE
Otolemur_garnetti        NGEIDIRGEDTEICLQVNQVTPDQLGNISLRHYLCNRPVGSDQKVAIHYKSSPEISLRFV
                         123456789012345678901234567890123456789012345678901234567890
                      1181        9      1200         1         2         3         4

                                                                             * *
Brachyteles_arachnoides  SGPGAVIHSLLAEKNGFLQCHIENFSTEFLTSSLLNIQHFLEDETVATVMPMQIEVSNTK
Callithrix_jacchus       SGPGAVIHSLLAEKNGFLQCHIENFSTEFLTSSLLNIQHFLEDETVATVMPMKIQVSNTK
Aotus_nancymaae          SGPGAVIHSLLAEKNGFLQCHIENFSTEFLTSSLLNIQHFLEDETVATVMPMKIQVSNTK
Saimiri_boliviensis      SGPGAVIHSLLAEKNGFLQCHIENFSTEFLTSSLLNIQHFLEDETVATVMPMKIQVSNTK
Macaca_nemestrina        SGPGAVIHSLLAEKNGFLQCHIENFSTEFLTSSLMNIQHFLEDETVATVMPMKIQVSNTK
Chlorocebus_sabaeus      SGPGAVIHSLLAEKNGFLQCHIENFSTEFLTSSLMNIQHFLEDETVATVMPMKIQVSNTK
Homo_sapiens             SGPGAVIHSLLAEKNGFLQCHIENFSTEFLTSSLMNIQHFLEDETVATVMPMKIQVSNTK
Pongo_abelii             SGPGAVIHSLLAEKNGFLQCHIENFSTEFLTSSLMNIQHFLEDETVATVMPMKIQVSNTK
Otolemur_garnetti        SGPGAVIHSLLAEKNGFLQCHVENFSTEFLISSLMNIQHFLEDETVATVMPMKIQVSNMK
                         123456789012345678901234567890123456789012345678901234567890
                      1241        5         6         7         8         9      1300

                                             *
Brachyteles_arachnoides  INLKDDSPRSSTVSLEPTPVAVHIDHLVVERNDDGSFHIR
Callithrix_jacchus       INLKDDSPRSSTVSLEPTPVTVHIDHLVVERNDDGSFHIR
Aotus_nancymaae          INLKDDSPRSSTVSLEPTPVTVHIDHLVVERNDDGSFHIR
Saimiri_boliviensis      INLKDDSPRSSTVSLEPTPITIHIDHLVVERNDDGSFHIR
Macaca_nemestrina        INLKDDSPRSSTISLEPAPVTVHIDHLVVERSDDGSFHIR
Chlorocebus_sabaeus      INLKDDSPRSSTISLEPAPVTVHIDHLVVERSDDGSFHIR
Homo_sapiens             INLKDDSPRSSTVSLEPAPVTVHIDHLVVERSDDGSFHIR
Pongo_abelii             INLKDDSPRSSTVSLEPAPVTVHIDHLVVERSDDGSFHIR
Otolemur_garnetti        INLKDDSPRSSAVSHEPTPVTIHIDHLVVERIDDGSFHIR
                         1234567890123456789012345678901234567890
                      1301        1         2         3         4
```

ZBTB44

```
                             |------ BTB/POZ domain --------
Brachyteles_arachnoides    MGVKTFTHSSSSHSQEMLGKLNMLRNDGHFCDITIRVQDKIFRAHKVVLAACSDFFRTKL
Aotus_nancymaae            MGVKTFTHSSSSHSQEMLGKLNMLRNDGHFCDITIRVQDKIFRAHKVVLAACSDFFRTKL
Cebus_capucinus            MGVKTFTHSSSSHSQEMLGKLNMLRNDGHFCDITIRVQDKIFRAHKVVLAACSDFFRTKL
Saimiri_boliviensis        MGVKTFTHSSSSHSQEMLGKLNMLRNDGHFCDITIRVQDKIFRAHKVVLAACSDFFRTKL
Macaca_nemestrina          MGVKTFTHSSSSHSQEMLGKLNMLRNDGHFCDITIRVQDKIFRAHKVVLAACSDFFRTKL
Papio_anubis               MGVKTFTHSSSSHSQEMLGKLNMLRNDGHFCDITIRVQDKIFRAHKVVLAACSDFFRTKL
Homo_sapiens               MGVKTFTHSSSSHSQEMLGKLNMLRNDGHFCDITIRVQDKIFRAHKVVLAACSDFFRTKL
Pongo_abelii               MGVKTFTHSSSSHSQEMLGKLNMLRNDGHFCDITIRVQDKIFRAHKVVLAACSDFFRTKL
Otolemur_garnetti          MGVKTFTHSSSSHSQEMLGKLNMLRNDGHFCDITIRVQDKIFRAHKVVLAACSDFFRTKL
                           123456789012345678901234567890123456789012345678901234567890
                                    1         2         3         4         5         6

                           --------------------------------------|
Brachyteles_arachnoides    VGQAEDENKNVLDLHHVTVTGFIPLLEYAYTATLSINTENIIDVLAAASYMQMFSVASTC
Aotus_nancymaae            VGQAEDENKNVLDLHHVTVTGFIPLLEYAYTATLSINTENIIDVLAAASYMQMFSVASTC
Cebus_capucinus            VGQAEDENKNVLDLHHVTVTGFIPLLEYAYTATLSINTENIIDVLAAASYMQMFSVASTC
Saimiri_boliviensis        VGQAEDENKNVLDLHHVTVTGFIPLLEYAYTATLSINTENIIDVLAAASYMQMFSVASTC
Macaca_nemestrina          VGQAEDENKNVLDLHHVTVTGFIPLLEYAYTATLSINTENIIDVLAAASYMQMFSVASTC
Papio_anubis               VGQAEDENKNVLDLHHVTVTGFIPLLEYAYTATLSINTENIIDVLAAASYMQMFSVASTC
Homo_sapiens               VGQAEDENKNVLDLHHVTVTGFIPLLEYAYTATLSINTENIIDVLAAASYMQMFSVASTC
Pongo_abelii               VGQAEDENKNVLDLHHVTVTGFIPLLEYAYTATLSINTENIIDVLAAASYMQMFSVASTC
Otolemur_garnetti          VGQAEDENKNVLDLHHVTVTGFIPLLEYAYTATLSINTENIIDVLAAASYMQMFSVASTC
                           123456789012345678901234567890123456789012345678901234567890
                         61         7         8         9        100         1         2

Brachyteles_arachnoides    SEFMKSSILWNTPNSQPEKGLDAGQENNSNCNFTSRDGSISPVSSECSVVERTIPVCRES
Aotus_nancymaae            SEFMKSSILWNTPNSQPEKGLDAGQENNSNCNFTSRDGSISPVSSECSVVERTIPVCRES
Cebus_capucinus            SEFMKSSILWNTPNSQPEKGLDAGQENNSNCNFTSRDGSISPVSSECSVVERTIPVCRES
Saimiri_boliviensis        SEFMKSSILWNTPNSQPEKGLDAGQENNSNCNFTSRDGSISPVSSECSVVERTIPVCRES
Macaca_nemestrina          SEFMKSSILWNTPNSQPEKGLDAGQENNSNCNFTSRDGSISPVSSECSVVERTIPVCRES
Papio_anubis               SEFMKSSILWNTPNSQPEKGLDAGQENNSNCNFTSRDGSISPVSSECSVVERTIPVCRES
Homo_sapiens               SEFMKSSILWNTPNSQPEKGLDAGQENNSNCNFTSRDGSISPVSSECSVVERTIPVCRES
Pongo_abelii               SEFMKSSILWNTPNSQPEKGLDAGPENNSNCNFTSRDGSISPVSSECSVVERTIPVCRES
Otolemur_garnetti          SEFMKSSILWNTPNSQPEKGLDAGQENNSNCNFTSRDGSISPVSSECSVVERAIPVCRES
                           123456789012345678901234567890123456789012345678901234567890
                        121         3         4         5         6         7         8

Brachyteles_arachnoides    RRKRKSYIVMSPESPVKCSTQTSSPQVLNSSASYSENRNQPVDSSLAFPWTFPFGIDRRI
Aotus_nancymaae            RRKRKSYIVMSPESPVKCSTQTSSPQVLNSSASYSENRNQPVDSSLAFPWTFPFGIDRRI
Cebus_capucinus            RRKRKSYIVMSPESPVKCSTQTSSPQVLNSSASYSENRNQPVDSSLAFPWTFPFGIDRRI
Saimiri_boliviensis        RRKRKSYIVMSPESPVKCSTQTSSPQVLNSSASYSENRNQQVDSSLAFPWTFPFGIDRRI
Macaca_nemestrina          RRKRKSYIVMSPESPVKCSTQTSSPQVLNSSASYSENRNQPVDSSLAFPWTFPFGIDRRI
Papio_anubis               RRKRKSYIVMSPESPVKCSTQTSSPQVLNSSASYSENRNQPVDSSLAFPWTFPFGIDRRI
Homo_sapiens               RRKRKSYIVMSPESPVKCGTQTSSPQVLNSSASYSENRNQPVDSSLAFPWTFPFGIDRRI
Pongo_abelii               RRKRKSYIVMSPESPVKCGTQTSSPQVLNSSASYSENRNQPVDSSLAFPWTFPFGIDRRI
Otolemur_garnetti          RRKRKSYIVMSPESPVKCSTQTNSPQVLNSSASYSENRNQPVDSSLAFPWTFPFGIDRRI
                           123456789012345678901234567890123456789012345678901234567890
                        181         9        200         1         2         3         4

Brachyteles_arachnoides    QPEKVKQGENTRTLELPGPSETGRRMTDYVTCESTKTTLPLGTEEDVRVKVERLSDEEVH
Aotus_nancymaae            QPEKVKQGENTRTLELPGPSETGRRMTDYVTCESTKTTLPLGTEEDVRVKVERLSDEEVH
Cebus_capucinus            QPEKVKQGENTRTLELPGPSETGRRMTDYVTCESTKTTLPLGSEEDVRVKVERLSDEEVH
Saimiri_boliviensis        QPEKVKQGENTRTLELPGPSETGRRMTDYVTCESTKTTLPLGTEEDVRVKVERLSDEEVH
Macaca_nemestrina          QPEKVKQAENTRTLELPGPSETGRRMADYVTCESTKTTLPLGTEEDVRVKVERLSDEEVH
Papio_anubis               QPEKVKQAENTRTLELPGPSETGRRMADYVTCESTKTTLPLGTEEDVRVKVERLSDEEVH
Homo_sapiens               QPEKVKQAENTRTLELPGPSETGRRMADYVTCESTKTTLPLGTEEDVRVKVERLSDEEVH
Pongo_abelii               QPEKVKQAENTRTLELPGPSEASRRMADYVTCESTKTTLPLGTEEDVRVKVERLSDEEVH
Otolemur_garnetti          QPEKVKQAESTRTLELPGPSETGRRIADYVTCESTKTTLPLGTEEDVRVKVERLSDEEVH
                           123456789012345678901234567890123456789012345678901234567890
                        241         5         6         7         8         9        300

Brachyteles_arachnoides    EEVSQPVSASQSSLSDQQTVPGSEQVQEDLLISPQSSSIGSVDEGVTEGLPTLQSTSSTN
Aotus_nancymaae            EEVSQPVSASQSSLSDQQTVPGSEQVQEDLLISPQSSSIGSVDEGVTEGLPTLQSTSSTN
Cebus_capucinus            EEVSQPVSASQSSLSDQQTVPGSEQVQEDLLISPQSSSIGSVDEGVTEGLPTLQSTSSTN
Saimiri_boliviensis        EEVSQPVSASQSSLSDQQTVPGSEQVQEDLLISPQSSSIGSVDEGVTEGLPTLQSTSSTN
Macaca_nemestrina          EEVSQPVSASQSSLSDQQTVPGSEQVQEDLLISPQSSSIGSVDEGVSEGLPTLQSSSSTA
Papio_anubis               EEVSQPVSASQSSLSDQQTVPGSEQVQEDLLISPQSSSIGSVDEGVSEGLPTLQSSSSTA
Homo_sapiens               EEVSQPVSASQSSLSDQQTVPGSEQVQEDLLISPQSSSIGSVDEGVSEGLPTLQSTSSTN
Pongo_abelii               EEVSQPVSASQSSLSDQQTVPGSEQVQEDLLISPQSSSIGSVDEGVSEGLPTLQSTSSTN
Otolemur_garnetti          EEVSQPVSASQSSLSDQQTVPGSEQVQEDLLISPQSSSIGSVDEGVTEGLPTLQSTSSTN
                           123456789012345678901234567890123456789012345678901234567890
                        301         1         2         3         4         5         6
```

```
                                                              | Zinc finger C2H2-type
                                               * *
Brachyteles_arachnoides  APADDDDRLENVQYPYQLYIAPATYSTERPSPNGPDRPFQCPTCGVRFTRIQNLKQHMLI
Aotus_nancymaae          APPDDDDRLENVQYPYQLYIAPSTSSTERPSPNGPDRPFQCPTCGVRFTRIQNLKQHMLI
Cebus_capucinus          APADDDDRLENVQYPYQLYIAPSTSSTERPSPNGPDRPFQCPTCGVRFTRIQNLKQHMLI
Saimiri_boliviensis      APADDDDRLENVQYPYQLYIAPSTSSTERPSPNGPDRPFQCPTCGVRFTRIQNLKQHMLI
Macaca_nemestrina        APPDDDDRLENVQYPYQLYIAPSTSSTERPSPNGPDRPFQCPTCGVRFTRIQNLKQHMLI
Papio_anubis             APPDDDDRLENVQYPYQLYIAPSTSSTERPSPNGPDRPFQCPTCGVRFTRIQNLKQHMLI
Homo_sapiens             APPDDDDRLENVQYPYQLYIAPSTSSTERPSPNGPDRPFQCPTCGVRFTRIQNLKQHMLI
Pongo_abelii             APPDDDDRLENVQYPYQLYIAPSTSSTERPSPNGPDRPFQCPTCGVRFTRIQNLKQHMLI
Otolemur_garnetti        PHADDDDRLESVQYPYQLYIAPSTSSTERPSPNGPDRPFQCPTCGVRFTRIQNLKQHMLI
                         123456789012345678901234567890123456789012345678901234567890
                       361        7         8         9       400         1         2

                         -|   | Zinc finger C2H2-type |   |- Zinc finger C2H2-type -|
Brachyteles_arachnoides  HSGIKPFQCDRCGKKFTRAYSLKMHRLKHEGKRCFRCQICSATFTSFGEYKHHMRVSRHI
Aotus_nancymaae          HSGIKPFQCDRCGKKFTRAYSLKMHRLKHEGKRCFRCQICSATFTSFGEYKHHMRVSRHI
Cebus_capucinus          HSGIKPFQCDRCGKKFTRAYSLKMHRLKHEGKRCFRCQICSATFTSFGEYKHHMRVSRHI
Saimiri_boliviensis      HSGIKPFQCDRCGKKFTRAYSLKMHRLKHEGKRCFRCQICSATFTSFGEYKHHMRVSRHI
Macaca_nemestrina        HSGIKPFQCDRCGKKFTRAYSLKMHRLKHEGKRCFRCQICSATFTSFGEYKHHMRVSRHI
Papio_anubis             HSGIKPFQCDRCGKKFTRAYSLKMHRLKHEGKRCFRCQICSATFTSFGEYKHHMRVSRHI
Homo_sapiens             HSGIKPFQCDRCGKKFTRAYSLKMHRLKHEGKRCFRCQICSATFTSFGEYKHHMRVSRHI
Pongo_abelii             HSGIKPFQCDRCGKKFTRAYSLKMHRLKHEGKRCFRCQICSATFTSFGEYKHHMRVSRHI
Otolemur_garnetti        HSGIKPFQCDRCGKKFTRAYSLKMHRLKHEGKRCFRCQICSATFTSFGEYKHHMRVSRHI
                         123456789012345678901234567890123456789012345678901234567890
                       421        3         4         5         6         7         8

                              |- Zinc finger C2H2-type -|
Brachyteles_arachnoides  IRKPRIYECKTCGAMFTNSGNLIVHLRSLNHEASELANYFQSSDFLVPDYLNQEQEETLV
Aotus_nancymaae          IRKPRIYECKTCGAMFTNSGNLIVHLRSLNHEASELANYFQSSDFLVPDYLNQEQEETLV
Cebus_capucinus          IRKPRIYECKTCGAMFTNSGNLIVHLRSLNHEASELANYFQSSDFLVPDYLNQEQEETLV
Saimiri_boliviensis      IRKPRIYECKTCGAMFTNSGNLIVHLRSLNHEASELANYFQSSDFLVPDYLNQEQEETLV
Macaca_nemestrina        IRKPRIYECKTCGAMFTNSGNLIVHLRSLNHEASELANYFQSSDFLVPDYLNQEQEETLV
Papio_anubis             IRKPRIYECKTCGAMFTNSGNLIVHLRSLNHEASELANYFQSSDFLVPDYLNQEQEETLV
Homo_sapiens             IRKPRIYECKTCGAMFTNSGNLIVHLRSLNHEASELANYFQSSDFLVPDYLNQEQEETLV
Pongo_abelii             IRKPRIYECKTCGAMFTNSGNLIVHLRSLNHEASELANYFQSSDFLVPDYLNQEQEETLV
Otolemur_garnetti        IRKPRIYECKTCGAMFTNSGNLIVHLRSLNHEASELANYFQSSDFLVPDYLNQEQEETLV
                         123456789012345678901234567890123456789012345678901234567890
                       481        9       500         1         2         3         4

Brachyteles_arachnoides  QYDLGEHGFESNSSVQMPVISQVSSTQNCESTFPLGSLGGLAEKEEEVPEQPKTSACAEA
Aotus_nancymaae          QYDLGEHGFESNSSVQMPVISQVSSTQNCESTFPLGSLGGLAEKEEEVPEQPKTSACAEA
Cebus_capucinus          QYDLGEHGFESNSSVQMPVISQVSSTQNCESTFPLGSLGGLAEKEEEVPEQPKTSACAEA
Saimiri_boliviensis      QYDLGEHGFESNSSVQMPVISQVSSTQNCESTFPLGSLGGLAEKEEEVPEQPKTSACAEA
Macaca_nemestrina        QYDLGEHGFESNSSVQMPVISQVSSTQNCESTFPLGSLGGLAEKEEEVPEQPKTSACAEA
Papio_anubis             QYDLGEHGFESNSSVQMPVISQVSSTQNCESTFPLGSLGGLAEKEEEVPEQPKTSACAEA
Homo_sapiens             QYDLGEHGFESNSSVQMPVISQVSSTQNCESTFPLGSLGGLAEKEEEVPEQPKSSACAEA
Pongo_abelii             QYDLGEHGFESNSSVQMPVISQVSSTQNCESTFPLGSLGGLAEKEEEVPEQPKTSACAEA
Otolemur_garnetti        QYDLGEHGFESNSSVQMPVISQVSSTQNCESTFPLGSLGGLTEKEEEVPEQPKTSACAEA
                         123456789012345678901234567890123456789012345678901234567890
                       541        5         6         7         8         9       600

Brachyteles_arachnoides  TRDDPPKSELSSITIE
Aotus_nancymaae          TRDDPPKSELSSITIE
Cebus_capucinus          TRDDPPKSELSSITIE
Saimiri_boliviensis      TRDDPPKSELSSITIE
Macaca_nemestrina        TRDDPPKSELSSITI-
Papio_anubis             TRDDPPKSELSSITI-
Homo_sapiens             TRDDPPKSELSSITIE
Pongo_abelii             TRDDPPKSELSSITIE
Otolemur_garnetti        TRDDPPKSELSSVTIE
                         123456789012345-
                       601        1
```

ZSWIM8

```
Brachyteles_arachnoides      MELMFAEWEDGERFSFEDSDRFEEDSLCSFISEAESLCQNWRGWRKQSAGPNSPTGGGGG
Callithrix_jacchus           MELMFAEWEDGERFSFEDSDRFEEDSLCSFISEAESLCQNWRGWRKQSAGPNSPTGGGGG
Aotus_nancymaae              MELMFAEWEDGERFSFEDSDRFEEDSLCSFISEAESLCQNWRGWRKQSAGPNSPTGGGGG
Cebus_capucinus_imitator     MELMFAEWEDGERFSFEDSDRFEEDSLCSFISEAESLCQNWRGWRKQSAGPNSPTGGGGG
Saimiri_boliviensis          MELMFAEWEDGERFSFEDSDRFEEDSLCSFISEAESLCQNWRGWRKQSAGPNSPTGGGGG
Macaca_nemestrina            MELMFAEWEDGERFSFEDSDRFEEDSLCSFISEAESLCQNWRGWRKQSAGPNSPTGGGGG
Papio_anubis                 MELMFAEWEDGERFSFEDSDRFEEDSLCSFISEAESLCQNWRGWRKQSAGPNSPTGGGGG
Chlorocebus_sabaeus          MELMFAEWEDGERFSFEDSDRFEEDSLCSFISEAESLCQNWRGWRKQSAGPNSPTGGGGG
Homo_sapiens                 MELMFAEWEDGERFSFEDSDRFEEDSLCSFISEAESLCQNWRGWRKQSAGPNSPTGGGGG
Pongo_abelii                 MELMFAEWEDGERFSFEDSDRFEEDSLCSFISEAESLCQNWRGWRKQSAGPNSPTGGGGG
Otolemur_garnetti            MELMFAEWEDGERFSFEDSDRFEEDSLCSFISEAESLCQNWRGWRKQSAGPNSPTGGGGG
                             123456789012345678901234567890123456789012345678901234567890
                                      1         2         3         4         5         6
```

```
Brachyteles_arachnoides      GGSGGTRMRDGLVIPLVELSAKQVAFHIPFEVVEKVYPPVPEQLQLRIAFWSFPENEEDI
Callithrix_jacchus           GGSGGTRMRDGLVIPLVELSAKQVAFHIPFEVVEKVYPPVPEQLQLRIAFWSFPENEEDI
Aotus_nancymaae              GGSGGTRMRDGLVIPLVELSAKQVAFHIPFEVVEKVYPPVPEQLQLRIAFWSFPENEEDI
Cebus_capucinus_imitator     GGSGGTRMRDGLVIPLVELSAKQVAFHIPFEVVEKVYPPVPEQLQLRIAFWSFPENEEDI
Saimiri_boliviensis          GGSGGTRMRDGLVIPLVELSAKQVAFHIPFEVVEKVYPPVPEQLQLRIAFWSFPENEEDI
Macaca_nemestrina            GGSGGTRMRDGLVIPLVELSAKQVAFHIPFEVVEKVYPPVPEQLQLRIAFWSFPENEEDI
Papio_anubis                 GGSGGTRMRDGLVIPLVELSAKQVAFHIPFEVVEKVYPPVPEQLQLRIAFWSFPENEEDI
Chlorocebus_sabaeus          GGSGGTRMRDGLVIPLVELSAKQVAFHIPFEVVEKVYPPVPEQLQLRIAFWSFPENEEDI
Homo_sapiens                 GGSGGTRMRDGLVIPLVELSAKQVAFHIPFEVVEKVYPPVPEQLQLRIAFWSFPENEEDI
Pongo_abelii                 GGSGGTRMRDGLVIPLVELSAKQVAFHIPFEVVEKVYPPVPEQLQLRIAFWSFPENEEDI
Otolemur_garnetti            GGGGGTRMRDGLVIPLVELSAKQVAFHIPFEVVEKVYPPVPEQLQLRIAFWSFPENEEDI
                             123456789012345678901234567890123456789012345678901234567890
                            61        7         8         9        100        1         2
```

```
                                                                                |---------
Brachyteles_arachnoides      RLYSCLANGSADEFQRGDQLFRMRAVKDPLQIGFHLSATVVPPQMVPPKGAYNVAVMFDR
Callithrix_jacchus           RLYSCLANGSADEFQRGDQLFRMRAVKDPLQIGFHLSATVVPPQMVPPKGAYNVAVMFDR
Aotus_nancymaae              RLYSCLANGSADEFQRGDQLFRMRAVKDPLQIGFHLSATVVPPQMVPPKGAYNVAVMFDR
Cebus_capucinus_imitator     RLYSCLANGSADEFQRGDQLFRMRAVKDPLQIGFHLSATVVPPQMVPPKGAYNVAVMFDR
Saimiri_boliviensis          RLYSCLANGSADEFQRGDQLFRMRAVKDPLQIGFHLSATVVPPQMVPPKGAYNVAVMFDR
Macaca_nemestrina            RLYSCLANGSADEFQRGDQLFRMRAVKDPLQIGFHLSATVVPPQMVPPKGAYNVAVMFDR
Papio_anubis                 RLYSCLANGSADEFQRGDQLFRMRAVKDPLQIGFHLSATVVPPQMVPPKGAYNVAVMFDR
Chlorocebus_sabaeus          RLYSCLANGSADEFQRGDQLFRMRAVKDPLQIGFHLSATVVPPQMVPPKGAYNVAVMFDR
Homo_sapiens                 RLYSCLANGSADEFQRGDQLFRMRAVKDPLQIGFHLSATVVPPQMVPPKGAYNVAVMFDR
Pongo_abelii                 RLYSCLANGSADEFQRGDQLFRMRAVKDPLQIGFHLSATVVPPQMVPPKGAYNVAVMFDR
Otolemur_garnetti            RLYSCLANGSADEFQRGDQLFRMRAVKDPLQIGFHLSATVVPPQMVPPKGAYNVAVMFDR
                             123456789012345678901234567890123456789012345678901234567890
                           121        3         4         5         6         7         8
```

```
                             --- Zinc finger SWIM-type --|
Brachyteles_arachnoides      CRVTSCSCTCGAGAKWCTHVVALCLFRIHNASAVCLRAPVSESLSRLQRDQLQKFAQYLI
Callithrix_jacchus           CRVTSCSCTCGAGAKWCTHVVALCLFRIHNASAVCLRAPVSESLSRLQRDQLQKFAQYLI
Aotus_nancymaae              CRVTSCSCTCGAGAKWCTHVVALCLFRIHNASAVCLRAPVSESLSRLQRDQLQKFAQYLI
Cebus_capucinus_imitator     CRVTSCSCTCGAGAKWCTHVVALCLFRIHNASAVCLRAPVSESLSRLQRDQLQKFAQYLI
Saimiri_boliviensis          CRVTSCSCTCGAGAKWCTHVVALCLFRIHNASAVCLRAPVSESLSRLQRDQLQKFAQYLI
Macaca_nemestrina            CRVTSCSCTCGAGAKWCTHVVALCLFRIHNASAVCLRAPVSESLSRLQRDQLQKFAQYLI
Papio_anubis                 CRVTSCSCTCGAGAKWCTHVVALCLFRIHNASAVCLRAPVSESLSRLQRDQLQKFAQYLI
Chlorocebus_sabaeus          CRVTSCSCTCGAGAKWCTHVVALCLFRIHNASAVCLRAPVSESLSRLQRDQLQKFAQYLI
Homo_sapiens                 CRVTSCSCTCGAGAKWCTHVVALCLFRIHNASAVCLRAPVSESLSRLQRDQLQKFAQYLI
Pongo_abelii                 CRVTSCSCTCGAGAKWCTHVVALCLFRIHNASAVCLRAPVSESLSRLQRDQLQKFAQYLI
Otolemur_garnetti            CRVTSCSCTCGAGAKWCTHVVALCLFRIHNASAVCLRAPVSESLSRLQRDQLQKFAQYLI
                             123456789012345678901234567890123456789012345678901234567890
                           181        9        200        1         2         3         4
```

```
Brachyteles_arachnoides      SELPQQILPTAQRLLDELLSSQSTAINTVCGAPDPTAGPSASDQSTWYLDESTLTDNIKK
Callithrix_jacchus           SELPQQILPTAQRLLDELLSSQSTAINTVCGAPDPTAGPSASDQSTWYLDESTLTDNIKK
Aotus_nancymaae              SELPQQILPTAQRLLDELLSSQSTAINTVCGAPDPTAGPSASDQSTWYLDESTLTDNIKK
Cebus_capucinus_imitator     SELPQQILPTAQRLLDELLSSQSTAINTVCGAPDPTAGPSASDQSTWYLDESTLTDNIKK
Saimiri_boliviensis          SELPQQILPTAQRLLDELLSSQSTAINTVCGAPDPTAGPSASDQSTWYLDESTLTDNIKK
Macaca_nemestrina            SELPQQILPTAQRLLDELLSSQSTAINTVCGAPDPTAGPSASDQSTWYLDESTLTDNIKK
Papio_anubis                 SELPQQILPTAQRLLDELLSSQSTAINTVCGAPDPTAGPSASDQSTWYLDESTLTDNIKK
Chlorocebus_sabaeus          SELPQQILPTAQRLLDELLSSQSTAINTVCGAPDPTAGPSASDQSTWYLDESTLTDNIKK
Homo_sapiens                 SELPQQILPTAQRLLDELLSSQSTAINTVCGAPDPTAGPSASDQSTWYLDESTLTDNIKK
Pongo_abelii                 SELPQQILPTAQRLLDELLSSQSTAINTVCGAPDPTAGPSASDQSTWYLDESTLTDNIKK
Otolemur_garnetti            SELPQQILPTAQRLLDELLSSQSTAINTVCGAPDPTAGPSASDQSTWYLDESTLTDNIKK
                             123456789012345678901234567890123456789012345678901234567890
                           241        5         6         7         8         9       300
```

```
Brachyteles_arachnoides      TLHKFCGPSPVVFSDVNSMYLSSTEPPAAAEWACLLRPLRGREPEGVWNLLSIVREMFKR
Callithrix_jacchus           TLHKFCGPSPVVFSDVNSMYLSSTEPPAAAEWACLLRPLRGREPEGVWNLLSIVREMFKR
Aotus_nancymaae              TLHKFCGPSPVVFSDVNSMYLSSTEPPAAAEWACLLRPLRGREPEGVWNLLSIVREMFKR
Cebus_capucinus_imitator     TLHKFCGPSPVVFSDVNSMYLSSTEPPAAAEWACLLRPLRGREPEGVWNLLSIVREMFKR
Saimiri_boliviensis          TLHKFCGPSPVVFSDVNSMYLSSTEPPAAAEWACLLRPLRGREPEGVWNLLSIVREMFKR
Macaca_nemestrina             TLHKFCGPSPVVFSDVNSMYLSSTEPPAAAEWACLLRPLRGREPEGVWNLLSIVREMFKR
Papio_anubis                 TLHKFCGPSPVVFSDVNSMYLSSTEPPAAAEWACLLRPLRGREPEGVWNLLSIVREMFKR
Chlorocebus_sabaeus          TLHKFCGPSPVVFSDVNSMYLSSTEPPAAAEWACLLRPLRGREPEGVWNLLSIVREMFKR
Homo_sapiens                 TLHKFCGPSPVVFSDVNSMYLSSTEPPAAAEWACLLRPLRGREPEGVWNLLSIVREMFKR
Pongo_abelii                 TLHKFCGPSPVVFSDVNSMYLSSTEPPAAAEWACLLRPLRGREPEGVWNLLSIVREMFKR
Otolemur_garnetti            TLHKFCGPSPVVFSDVNSMYLSSTEPPAAAEWACLLRPLRGREPEGVWNLLSIVREMFKR
                             123456789012345678901234567890123456789012345678901234567890
                           301         1         2         3         4         5         6

Brachyteles_arachnoides      RDSNAAPLLEILTDQCLTYEQITGWWYSVRTSASHSSASGHTGRSNGQSEVAAHACASMC
Callithrix_jacchus           RDSNAAPLLEILTDQCLTYEQITGWWYSVRTSASHSSASGHTGRSNGQSEVAAHACASMC
Aotus_nancymaae              RDSNAAPLLEILTDQCLTYEQITGWWYSVRTSASHSSASGHTGRSNGQSEVAAHACASMC
Cebus_capucinus_imitator     RDSNAAPLLEILTDQCLTYEQITGWWYSVRTSASHSSASGHTGRSNGQSEVAAHACASMC
Saimiri_boliviensis          RDSNAAPLLEILTDQCLTYEQITGWWYSVRTSASHSSASGHTGRSNGQSEVAAHACASMC
Macaca_nemestrina             RDSNAAPLLEILTDQCLTYEQITGWWYSVRTSASHSSASGHTGRSNGQSEVAAHACASMC
Papio_anubis                 RDSNAAPLLEILTDQCLTYEQITGWWYSVRTSASHSSASGHTGRSNGQSEVAAHACASMC
Chlorocebus_sabaeus          RDSNAAPLLEILTDQCLTYEQITGWWYSVRTSASHSSASGHTGRSNGQSEVAAHACASMC
Homo_sapiens                 RDSNAAPLLEILTDQCLTYEQITGWWYSVRTSASHSSASGHTGRSNGQSEVAAHACASMC
Pongo_abelii                 RDSNAAPLLEILTDQCLTYEQITGWWYSVRTSASHSSASGHTGRSNGQSEVAAHACASMC
Otolemur_garnetti            RDSNAAPLLEILTDQCLTYEQITGWWYSVRTSASHSSASGHTGRSNGQSEVAAHACASMC
                             123456789012345678901234567890123456789012345678901234567890
                           361         7         8         9        400        1         2

Brachyteles_arachnoides      DEMVTLWRLAVLDPALSPQRRRELCAQLRQWQLKVIENVKRGQHKKTLERLFPGFRPAVE
Callithrix_jacchus           DEMVTLWRLAVLDPALSPQRRRELCAQLRQWQLKVIENVKRGQHKKTLERLFPGFRPAVE
Aotus_nancymaae              DEMVTLWRLAVLDPALSPQRRRELCAQLRQWQLKVIENVKRGQHKKTLERLFPGFRPAVE
Cebus_capucinus_imitator     DEMVTLWRLAVLDPALSPQRRRELCAQLRQWQLKVIENVKRGQHKKTLERLFPGFRPAVE
Saimiri_boliviensis          DEMVTLWRLAVLDPALSPQRRRELCSQLRQWQLKVIENVKRGQHKKTLERLFPGFRPAVE
Macaca_nemestrina             DEMVTLWRLAVLDPALSPQRRRELCTQLRQWQLKVIENVKRGQHKKTLERLFPGFRPAVE
Papio_anubis                 DEMVTLWRLAVLDPALSPQRRRELCTQLRQWQLKVIENVKRGQHKKTLERLFPGFRPAVE
Chlorocebus_sabaeus          DEMVTLWRLAVLDPALSPQRRRELCAQLRQWQLKVIENVKRGQHKKTLERLFPGFRPAVE
Homo_sapiens                 DEMVTLWRLAVLDPALSPQRRRELCTQLRQWQLKVIENVKRGQHKKTLERLFPGFRPAVE
Pongo_abelii                 DEMVTLWRLAVLDPALSPQRRRELCTQLRQWQLKVIENVKRGQHKKTLERLFPGFRPAVE
Otolemur_garnetti            DEMVTLWRLAVLDPALSPQRRRELCAQLRQWQLKVIENVKRGQHKKTLERLFPGFRPAVE
                             123456789012345678901234567890123456789012345678901234567890
                           421         3         4         5         6         7         8

                                                                     *
Brachyteles_arachnoides      ACYFNWEEAYPLPGVTYSGTDRKLALCWARALPSRPGVSRPGGLEESRDRPRPLPAEPAV
Callithrix_jacchus           ACYFNWEEAYPLPGVTYSGTDRKLALCWARALPSRPGVSRSGGLEESRDRPRPLPAEPAV
Aotus_nancymaae              ACYFNWEEAYPLPGVTYSGTDRKLALCWARALPSRPGVSRSGGLEESRDRPRPLPAEPAV
Cebus_capucinus_imitator     ACYFNWEEAYPLPGVTYSGTDRKLALCWARALPSRPGVSRSGALEESRDRPRPLPAEPAV
Saimiri_boliviensis          ACYFNWEEAYPLPGVTYSGTDRKLALCWARALPSRPGVSRSGGLEESRDRPRPLPAEPAV
Macaca_nemestrina             ACYFNWEEAYPLPGVTYSGTDRKLALCWARALPSRPGASRSGGLEESRDRPRPLPAEPAV
Papio_anubis                 ACYFNWEEAYPLPGVTYSGTDRKLALCWARALPSRPGASRSGGLEESRDRPRPLPAEPAV
Chlorocebus_sabaeus          ACYFNWEEAYPLPGVTYSGTDRKLALCWARALPSRPGASRSGGLEESRDRPRPLPAEPAV
Homo_sapiens                 ACYFNWEEAYPLPGVTYSGTDRKLALCWARALPSRPGASRSGGLEESRDRPRPLPTEPAV
Pongo_abelii                 ACYFNWEEAYPLPGVTYSGTDRKLALCWARALPSRPGASRSGGLEESRDRPRPLPAEPAV
Otolemur_garnetti            ACYFNWEEAYPLPGVTYSGTDRKLALCWARALPSRPGACRSGGLEESRDRPRSLPTEPAV
                             123456789012345678901234567890123456789012345678901234567890
                           481         9        500        1         2         3         4

Brachyteles_arachnoides      RPKEPGTKRKGLGDGVPSSQRGPRRLSAEGGDKALHKMGPGGGKAKALGGAGSGSKGSAG
Callithrix_jacchus           RPKEPGTKRKGLGDGVSSSQRGPRRLSAEGGDKALHKMGPGGGKAKALGGAGSGSKGSAG
Aotus_nancymaae              RPKEPGTKRKGLGDGVPSSQRGPRRLSAEGGDKALHKMGPGGGKAKALGGAGSGSKGSAG
Cebus_capucinus_imitator     RPKEPGNKRKGLGDGVPSSQRGPRRLSAEGGDKALHKMGPGGGKAKALGGAGSGSKGSA-
Saimiri_boliviensis          RPKEPGTKRKGLGDGVPSSQRGPRRLSAEGGDKALHKMGPGGGKAKALGGAGSGSKGSAG
Macaca_nemestrina             RPKEPGTKRKGLGEGVPSSQRGPRRLSAEGGDKALHKMGPGGGKAKALGGAGSGSKGSAG
Papio_anubis                 RPKEPGTKRKGLGEGVPSSQRGPRRLSAEGGDKALHKMGPGGGKAKALGGAGSGSKGSAG
Chlorocebus_sabaeus          RPKEPGTKRKGLGEGVPSSQRGPRRLSAEGGDKALHKMGPGGGKAKALGGAGSGSKGSAG
Homo_sapiens                 RPKEPGTKRKGLGEGVPSSQRGPRRLSAEGGDKALHKMGPGGGKAKALGGAGSGSKGSAG
Pongo_abelii                 RPKEPGTKRKGLGDGVPSSQRGPRRLSAEGGDKALHKMGPGGGKAKALGGAGSGSQGSAG
Otolemur_garnetti            RPKEPGAKRKGLGEGIPSSQRGPRRLSAEGGDKALHKLGPGGGKAKALGGAGSGSKGSA-
                             12345678901234567890123456789012345678901234567890123456789-
                           541         5         6         7         8         9
```

```
                         *                                      *
Brachyteles_arachnoides  AGSKRRLSSEDSSLEPDLAEMSLDDSSLALGAEASTFGNFPES-PPPCPPHGGSRGPSTF
Callithrix_jacchus       GGSKRRLSSEDSSLEPDLAEMSLDDSSLALGAEASTFGGFPES-PPPCPPHGGSRGPSTF
Aotus_nancymaae          GGSKRRLSSEDSSLEPDLAEMSLDDSSLALGAEASTFGGFPES-PPPCPPHGGSRGPSTF
Cebus_capucinus_imitator GGSKRRLSSEDSSLEPDLAEMSLDDSSLALGAEASTFGGFPES-PPPCPPHGGSRVPSTF
Saimiri_boliviensis      GGSKRRLSSEDSSLEPDLAEMSLDDSSLALGAEASTFGGFPES-PPPCPPHGGSRGPSTF
Macaca_nemestrina        GGSKRRLSSEDSSLEPDLAEMSLDDSSLALGAEASTFGGFPES-PPPCPLHGGSRGPSAF
Papio_anubis             GGSKRRLSSEDSSLEPDLAEMSLDDSSLALGAEASTFGGFPES-PPPCPLHGGSRGPSTF
Chlorocebus_sabaeus      GGSKRRLSSEDSSLEPDLAEMSLDDSSLALGAEASTFGGFPES-PPPCPLHGGSRGPSTF
Homo_sapiens             GGSKRRLSSEDSSLEPDLAEMSLDDSSLALGAEASTFGGFPES-PPPCPLHGGSRGPSTF
Pongo_abelii             GGSKRRLSSEDSSLEPDLAEMSLDDSSLALGAEASTFGGFPESPPPPCPLHSGSRGPSTF
Otolemur_garnetti        GGSKRRLSSEDSSLEPDLAEMSLDDSSLALGAEASTFGGFPES-PPPCPPPGGSRGASTF
                         012345678901234567890123456789012345678901 2-345678901234567 8
                       600         1         2         3         4          5

                            *                                                    *
Brachyteles_arachnoides  LPESPDTYEEDGGVYFSEGPEPPTASAGPPGLLPGEVCTRDDLPSTDESGSGLPKAKEAA
Callithrix_jacchus       LPEPPDTYEEDGGVYFSEGPEPPTASAGPPGLLPGEICTRDDLPSTDESGSGLPKTKEAA
Aotus_nancymaae          LPEPPDTYEEDGGVYFSEGPEPPTVSAGPPGLLPGEICTRDDLPSTDESGSGLPKAKEAA
Cebus_capucinus_imitator LPEPPDTYEEDGGVYFSEGPEPPTASAGPPGLLPGEVCTRDDLPSADESGSGLPQTKEAA
Saimiri_boliviensis      LPEPPDTYEEDGGVYFSEGPEPPTASAGPPGLLPGEVCTRDDLPSTDESGSGLPQTKEAV
Macaca_nemestrina        LPEPPDTYEEDGGVYFSEGPEPPTASAGPPGLLPGDVCTRDDVPSTDESGSGLPKTKEAA
Papio_anubis             LPESPDTYEEDGGVYFSEGPEPPTTSAGPPGLLPGDVCTRDDLPSTDESGSGLPKTKEAA
Chlorocebus_sabaeus      LPEPPDTYEEDGGVYFSEGPEPPTASAGPPGLLPGDVCTRDDFPSTDESGSGLPKTKEAA
Homo_sapiens             LPEPPDTYEEDGGVYFSEGPEPPTASVGPPGLLPGDVCTQDDLPSTDESGNGLPKTKEAA
Pongo_abelii             LPEPPDTYEEDGGVYFSEGPEPPTASVGPPGLLPGDVCTQDDLPSTDESGNGLPKTKEAA
Otolemur_garnetti        LPEPPDTYEEDGGVYFSEGPEPPTVSAGPSGLLPGEVCTPDDLPSTDESGNGLPKTKEVA
                         901234567890123456789012345678901234567890123456789012345678
                         660        7         8         9        700         1

Brachyteles_arachnoides  PAVGEEDDDYQAYYLNAQDGAGGEEEKAEGGAGEEHDLFAGLKPLEQESRMEVLFACAEA
Callithrix_jacchus       PAVGEEDDDYQAYYLNAQDGAGGEEEKAEGGAGEEHDLFAGLKPLEQESRMEVLFACAEA
Aotus_nancymaae          PAVGEEDDDYQAYYLNAQDGAGGEEEKAEGGAGEEHDLFAGLKPLEQESRMEVLFACAEA
Cebus_capucinus_imitator PAVGEEDDDYQAYYLNAQDGAGGEEEKAEGGAGEEHDLFAGLKPLEQESRMEVLFACAEA
Saimiri_boliviensis      PAVGEEDDDYQAYYLNAQDGAGGEEEKAEGAAGEEHDLFAGLKPLEQESRMEVLFACAEA
Macaca_nemestrina        PAVGEEDDDYQAYYLNAQDGAGGEEEKAEGGAGEEHDLFAGLKPLEQESRMEVLFACAEA
Papio_anubis             PAVGEEDDDYQAYYLNAQDGAGGEEEKAEGGAGEEHDLFAGLKPLEQESRMEVLFACAEA
Chlorocebus_sabaeus      PAAGEEDDDYQAYYLNAQDGAGGEEEKAEGGAGEEHDLFAGLKPLEQESRMEVLFACAEA
Homo_sapiens             PAVGEEDDDYQAYYLNAQDGAGGEEEKAEGGAGEEHDLFAGLKPLEQESRMEVLFACAEA
Pongo_abelii             PAVGEEDDDYQAYYLNAQDGAGGEEEKAEGGAGEEHDLFAGLKPLEQESRMEVLFACAEA
Otolemur_garnetti        PAVGEEDDDYQAYYLNAQDGAGGEEEKAEGGSGEEHDLFAGLKPLEQESRMEVLFACAEA
                         901234567890123456789012345678901234567890123456789012345678
                         720        3         4         5         6         7

Brachyteles_arachnoides  LHAHGYSNEASRLTVELAQDLLANPPDLKVEPPPAKGKKNKVSTSRQTWVATNTLSKAAF
Callithrix_jacchus       LHAHGYSSEASRLTVELAQDLLANPPDLKVEPPPAKGKKNKVSTSRQTWVATNTLSKAAF
Aotus_nancymaae          LHAHGYSSEASRLTVELAQDLLANPPDLKVEPPPAKGKKNKVSTSRQTWVATNTLSKAAF
Cebus_capucinus_imitator LHAHGYSSEASRLTVELAQDLLANPPDLKVEPPPAKGKKNKVSTSRQTWVATNTLSKAAF
Saimiri_boliviensis      LHAHGYSSEASRLTVELAQDLLANPPDLKVEPPPAKGKKNKVSTSRQTWVATNTLSKAAF
Macaca_nemestrina        LHAHGYSNEASRLTVELAQDLLANPPDLKVEPPPAKGKKNKVSTSRQTWVATNTLSKAAF
Papio_anubis             LHAHGYSNEASRLTVELAQDLLANPPDLKVEPPPAKGKKNKVSTSRQTWVATNTLSKAAF
Chlorocebus_sabaeus      LHAHGYSNEASRLTVELAQDLLANPPDLKVEPPPAKGKKNKVSTSRQTWVATNTLSKAAF
Homo_sapiens             LHAHGYSSEASRLTVELAQDLLANPPDLKVEPPPAKGKKNKVSTSRQTWVATNTLSKAAF
Pongo_abelii             LHAHGYSNEASRLTVELAQDLLANPPDLKVEPPPAKGKKNKVSTSRQTWVATNTLSKAAF
Otolemur_garnetti        LHAHGYSNEASRLTVELAQDLLANPPDLKVEPPPAKGKKNKVSTSRQTWVATNTLTKAAF
                         901234567890123456789012345678901234567890123456789012345678
                         780        9        800        1         2         3

Brachyteles_arachnoides  LLTVLSERPEHHNLAFRVGMFALELQRPPASTKALEVKLAYQESEVAALLKKIPLGPSEM
Callithrix_jacchus       LLTVLSERPEHHNLAFRVGMFALELQRPPASTKALEVKLAYQESEVAALLKKIPLGPSEM
Aotus_nancymaae          LLTVLSERPEHHNLAFRVGMFALELQRPPASTKALEVKLAYQESEVAALLKKIPLGPSEM
Cebus_capucinus_imitator LLTVLSERPEHHNLAFRVGMFALELQRPPASTKALEVKLAYQESEVAALLKKIPLGPSEM
Saimiri_boliviensis      LLTVLSERPEHHNLAFRVGMFALELQRPPASTKALEVKLAYQESEVAALLKKIPLGPSEM
Macaca_nemestrina        LLTVLSERPEHHNLAFRVGMFALELQRPPASTKALEVKLAYQESEVAALLKKIPLGPSEM
Papio_anubis             LLTVLSERPEHHNLAFRVGMFALELQRPPASTKALEVKLAYQESEVAALLKKIPLGPSEM
Chlorocebus_sabaeus      LLTVLSERPEHHNLAFRVGMFALELQRPPASTKALEVKLAYQESEVAALLKKIPLGPSEM
Homo_sapiens             LLTVLSERPEHHNLAFRVGMFALELQRPPASTKALEVKLAYQESEVAALLKKIPLGPSEM
Pongo_abelii             LLTVLSERPEHHNLAFRVGMFALELQRPPASTKALEVKLAYQESEVAALLKKIPLGPSEM
Otolemur_garnetti        LLTVLSERPEHHNLAFRVGMFALELQRPPASTKALEVKLAYQESEVAALLKKIPLGPSEM
                         901234567890123456789012345678901234567890123456789012345678
                         840        5         6         7         8         9
```

```
Brachyteles_arachnoides      STMRCRAEELREGTLCDYRPVLPLMLASFIFDVLCAPGSRPPSRNWNSETPGDEELGFEA
Callithrix_jacchus           STMRCRAEELREGTLCDYRPVLPLMLASFIFDVLCAPGSRPPSRNWNSETPGDEELGFEA
Aotus_nancymaae              STMRCRAEELREGTLCDYRPVLPLMLASFIFDVLCAPGSRPPSRNWNSETPGDEELGFEA
Cebus_capucinus_imitator     STMRCRAEELREGTLCDYRPVLPLMLASFIFDVLCAPGSRPPSRNWNSETPGDEELGFEA
Saimiri_boliviensis          STMRCRAEELREGTLCDYRPVLPLMLASFIFDVLCAPGSRPPSRNWNSETPGDEELGFEA
Macaca_nemestrina            STMRCRAEELREGTLCDYRPVLPLMLASFIFDVLCAPGSRPPSRNWNSETPGDEELGFEA
Papio_anubis                 STMRCRAEELREGTLCDYRPVLPLMLASFIFDVLCAPGSRPPSRNWNSETPGDEELGFEA
Chlorocebus_sabaeus          STMRCRAEELREGTLCDYRPVLPLMLASFIFDVLCAPGSRPPSRNWNSETPGDEELGFEA
Homo_sapiens                 STMRCRAEELREGTLCDYRPVLPLMLASFIFDVLCAPGSRPPSRNWNSETPGDEELGFEA
Pongo_abelii                 STMRCRAEELREGTLCDYRPVLPLMLASFIFDVLCAPGSRPPSRNWNSETPGDEELGFEA
Otolemur_garnetti            STMRCRAEELREGTLCDYRPVLPLMLASFIFDVLCAPGSRPPSRNWNNEMPGDEELGFEA
                             901234567890123456789012345678901234567890123456789012345678
                            900          1         2         3         4         5
```

```
Brachyteles_arachnoides      AVAALGMKTTVSEAEHPLLCEGTRREKGDLALALMITYKDDQAKLKKILDKLLDRESQTH
Callithrix_jacchus           AVAALGMKTTVSEAEHPLLCEGTRREKGDLALALMITYKDDQAKLKKILDKLLDRESQTH
Aotus_nancymaae              AVAALGMKTTVSEAEHPLLCEGTRREKGDLALALMITYKDDQAKLKKILDKLLDRESQTH
Cebus_capucinus_imitator     AVAALGMKTTVSEAEHPLLCEGTRREKGDLALALMITYKDDQAKLKKILDKLLDRESQTH
Saimiri_boliviensis          AVAALGMKTTVSEAEHPLLCEGTRREKGDLALALMITYKDDQAKLKKILDKLLDRESQTH
Macaca_nemestrina            AVAALGMKTTVSEAEHPLLCEGTRREKGDLALALMITYKDDQAKLKKILDKLLDRESQTH
Papio_anubis                 AVAALGMKTTVSEAEHPLLCEGTRREKGDLALALMITYKDDQAKLKKILDKLLDRESQTH
Chlorocebus_sabaeus          AVAALGMKTTVSEAEHPLLCEGTRREKGDLALALMITYKDDQAKLKKILDKLLDRESQTH
Homo_sapiens                 AVAALGMKTTVSEAEHPLLCEGTRREKGDLALALMITYKDDQAKLKKILDKLLDRESQTH
Pongo_abelii                 AVAALGMKTTVSEAEHPLLCEGTRREKGDLALALMITYKDDQAKLKKILDKLLDRESQTH
Otolemur_garnetti            AVAALGMKTTVSEAEHPLLCEGTRREKGDLALSLMITYKDDQAKLKKILDKLLDRESQTH
                             901234567890123456789012345678901234567890123456789012345678
                            960          7         8         9     1000          1
```

```
Brachyteles_arachnoides      KPQTLSSFYSSSRPATASQRSPSKHGGPSAPGALQPLTSGSAGPAQPGSVAGAGPGPTEG
Callithrix_jacchus           KPQTLSSFYSSSRPATASQRSPSKHGGPSAPGALQPLTSGSAGPAQPGSVAGAGPGPTEG
Aotus_nancymaae              KPQTLSSFYSSSRPATASQRSPSKHGGPSAPGALQPLTSGSAGPAQPGSVAGAGPDPTEG
Cebus_capucinus_imitator     KPQTLSSFYSSSRPATASQRSPSKHGGPSAPGALQPLTSGSAGPAQPGSVAGAGPGPTEG
Saimiri_boliviensis          KPQTLSSFYSSSRPATASQRSPSKHGGPSAPGALQPLTSGSAGPAQPGSVAGAGPGPTEG
Macaca_nemestrina            KPQTLSSFYSSSRPTTASQRSPSKHGGPSAPGALQPLTSGSAGPAQPGSVAGAGPGPTEG
Papio_anubis                 KPQTLSSFYSSSRPTTASQRSPSKHGGPSAPGALQPLTSGSAGPAQPGSVAGAGPGPTEG
Chlorocebus_sabaeus          KPQTLSSFYSSSRPTTASQRSPSKHGGPSAPGALQPLTSGSAGPAQPGSVAGAGPGPTEG
Homo_sapiens                 KPQTLSSFYSSSRPTTASQRSPSKHGGPSAPGALQPLTSGSAGPAQPGSVAGAGPGPTEG
Pongo_abelii                 KPQTLSSFYSSSRPTTASQRSPSKHGGPSAPGALQPLTSGSAGPAQPGSVAGAGPGPTEG
Otolemur_garnetti            KPQTLSSFYSSSRPATASQRSPSKHGGPSAPGALQPLTSGSAGPAQPGSVTGAGPGPTEG
                             901234567890123456789012345678901234567890123456789012345678
                           1020          3         4         5         6         7
```

```
Brachyteles_arachnoides      FTEKNVPESSPHSPCEGLPSEAALTPRPEGKVPSRLALGSRGGYNGRGWGSPGRPKKKHT
Callithrix_jacchus           FTEKNVPESSPHSPCEGLPSEAALTPRPEGKVPSRLALGSRGGYNGRGWGSPGRPKKKHT
Aotus_nancymaae              FTEKNVPESSPHSPCEGLPSEAALTPRPEGKVPSRLALGSRGGYNGRGWGSPGRPKKKHT
Cebus_capucinus_imitator     FTEKNVPESSPHSPCEGLPSEAALTPRPEGKVPSRLALGSRGGYNGRGWGSPGRPKKKHT
Saimiri_boliviensis          FTEKNVPESSPHSPCEGLPSEAALTPRPEGKVPSRLALGSRGGYNGRGWGSPGRPKKKHT
Macaca_nemestrina            FTEKNVPESSPHSPCEGLPSEAALTPRPEGKVPSRLALGSRGGYNGRGWGSPGRPKKKHT
Papio_anubis                 FTEKNVPESSPHSPCEGLPSEAALTPRPEGKVPSRLALGSRGGYNGRGWGSPGRPKKKHT
Chlorocebus_sabaeus          FTEKNVPESSPHSPCEGLPPEAALTPRPEGKVPSRLALGSRGGYNGRGWGSPGRPKKKHT
Homo_sapiens                 FTEKNVPESSPHSPCEGLPSEAALTPRPEGKVPSRLALGSRGGYNGRGWGSPGRPKKKHT
Pongo_abelii                 FTEKNVPESSPHSPCEGLPSEAALTPRPEGKVPSRLALGSRGGYNGRGWGSPGRPKKKHT
Otolemur_garnetti            FTEKNVPESSPHSPCEVLPPEAALTPRPEGKVPSRLALGSRGGYNGRGWGSPGRPKKKHT
                             901234567890123456789012345678901234567890123456789012345678
                           1080          9     1100        1         2         3
```

```
Brachyteles_arachnoides      GMASIDSSAPETTSDSSPTLSRRPLRGGWAPTSWGRGQDSDSISSSSSDSLGSSSSSGSR
Callithrix_jacchus           GMASIDSSAPETTSDSSPTLSRRPLRGGWAPTSWGRGQDSDSISSSSSDSLGSSSSSGSR
Aotus_nancymaae              GMASIDSSAPETTSDSSPTLSRRPLRGGWAPTSWGRGQDSDSISSSSSDSLGSSSSSGSR
Cebus_capucinus_imitator     GMASIDSSAPETTSDSSPTLSRRPLRGGWAPTSWGRGQDSDSISSSSSDSLGSSSSSGSR
Saimiri_boliviensis          GMASIDSSAPETTSDSSPTLSRRPLRGGWAPTSWGRGQDSDSISSSSSDSLGSSSSSGSR
Macaca_nemestrina            GMASIDSSAPETTSDSSPTLSRRPLRGGWAPTSWGRGQDSDSISSSSSDSLGSSSSSGSR
Papio_anubis                 GMASIDSSAPETTSDSSPTLSRRPLRGGWAPTSWGRGQDSDSISSSSSDSLGSSSSSGSR
Chlorocebus_sabaeus          GMASIDSSAPETTSDSSPTLSRRPLRGGWAPTSWGRGQDSDSISSSSSDSLGSSSSSGSR
Homo_sapiens                 GMASIDSSAPETTSDSSPTLSRRPLRGGWAPTSWGRGQDSDSISSSSSDSLGSSSSSGSR
Pongo_abelii                 GMASIDSSAPETTSDSSPTLSRRPLRGGWAPTSWGRGQDSDSISSSSSDSLGSSSSSGSR
Otolemur_garnetti            GMASIDSSAPETTSDSSPTLSRRPLRGGWAPTSWGRGQDSDSISSSSSDSLGSSSSSGSR
                             901234567890123456789012345678901234567890123456789012345678
                           1140          5         6         7         8         9
```

```
Brachyteles_arachnoides      RASASGGARAKTVEVGRYKGRRPESHAPHVPNQPSEAAAHFYFELAKTVLIKAGGNSSTS
Callithrix_jacchus           RASASGGARAKTVEVGRYKGRRPESHAPHVPNQPSEAAAHFYFELAKTVLIKAGGNSSTS
Aotus_nancymaae              RASASGGARAKTVEVGRYKGRRPESHAPHVPNQPSEAAAHFYFELAKTVLIKAGGNSSTS
Cebus_capucinus_imitator     RASASGGARAKTVEVGRYKGRRPESHAPHVPNQPSEAAAHFYFELAKTVLIKAGGNSSTS
Saimiri_boliviensis          RASASGGARAKTVEVGRYKGRRPESHAPHVPNQPSEAAAHFYFELAKTVLIKAGGNSSTS
Macaca_nemestrina            RASASGGARAKTVEVGRYKGRRPESHAPHVPNQPSEAAAHFYFELAKTVLIKAGGNSSTS
Papio_anubis                 RASASGGARAKTVEVGRYKGRRPESHAPHVPNQPSEAAAHFYFELAKTVLIKAGGNSSTS
Chlorocebus_sabaeus          RASASGGARAKTVEVGRYKGRRPESHAPHVPNQPSEAAAHFYFELAKTVLIKAGGNSSTS
Homo_sapiens                 RASASGGARAKTVEVGRYKGRRPESHAPHVPNQPSEAAAHFYFELAKTVLIKAGGNSSTS
Pongo_abelii                 RASTSGGARAKTVEVGRYKGRRPESHAPHVPNQPSEAAAHFYFELAKTVLIKAGGNSSTS
Otolemur_garnetti            RASASGGARAKTVEVGRYKGRRPESHAPHVPNQPSEAAAHFYFELAKMVLIKAGGNSSTS
                             901234567890123456789012345678901234567890123456789012345678
                           1200        1         2         3         4         5

Brachyteles_arachnoides      IFTHPSSSGGHQGPHRNLHLCAFEIGLYALGLHNFVSPNWLSRTYSSHVSWITGQAMEIG
Callithrix_jacchus           IFTHPSSSGGHQGPHRNLHLCAFEIGLYALGLHNFVSPNWLSRTYSSHVSWITGQAMEIG
Aotus_nancymaae              IFTHPSSSGGHQGPHRNLHLCAFEIGLYALGLHNFVSPNWLSRTYSSHVSWITGQAMEIG
Cebus_capucinus_imitator     IFTHPSSSGGHQGPHRNLHLCAFEIGLYALGLHNFVSPNWLSRTYSSHVSWITGQAMEIG
Saimiri_boliviensis          IFTHPSSSGGHQGPHRNLHLCAFEIGLYALGLHNFVSPNWLSRTYSSHVSWITGQAMEIG
Macaca_nemestrina            IFTHPSSSGGHQGPHRNLHLCAFEIGLYALGLHNFVSPNWLSRTYSSHVSWITGQAMEIG
Papio_anubis                 IFTHPSSSGGHQGPHRNLHLCAFEIGLYALGLHNFVSPNWLSRTYSSHVSWITGQAMEIG
Chlorocebus_sabaeus          IFTHPSSSGGHQGPHRNLHLCAFEIGLYALGLHNFVSPNWLSRTYSSHVSWITGQAMEIG
Homo_sapiens                 IFTHPSSSGGHQGPHRNLHLCAFEIGLYALGLHNFVSPNWLSRTYSSHVSWITGQAMEIG
Pongo_abelii                 IFTHPSSSGGHQGPHRNLHLCAFEIGLYALGLHNFVSPNWLSRTYSSHVSWITGQAMEIG
Otolemur_garnetti            IFTHPSSSGGHQGPHRNLHLCAFEIGLYALGLHNFVSPNWLSRTYSSHVSWITGQAMEIG
                             901234567890123456789012345678901234567890123456789012345678
                           1260        7         8         9       1300        1

Brachyteles_arachnoides      SAALTILVECWDGHLTPPEVASLADRASRARDSNMVRAAAELALSCLPHAHALNPNEIQR
Callithrix_jacchus           SAALTILVECWDGHLTPPEVASLADRASRARDSNMVRAAAELALSCLPHAHALNPNEIQR
Aotus_nancymaae              SAALTILVECWDGHLTPPEVASLADRASRARDSNMVRAAAELALSCLPHAHALNPNEIQR
Cebus_capucinus_imitator     SAALTILVECWDGHLTPPEVASLADRASRARDSNMVRAAAELALSCLPHAHALNPNEIQR
Saimiri_boliviensis          SAALTILVECWDGHLTPPEVASLADRASRARDSNMVRAAAELALSCLPHAHALNPNEIQR
Macaca_nemestrina            SAALTILVECWDGHLTPPEVASLADRASRARDSNMVRAAAELALSCLPHAHALNPNEIQR
Papio_anubis                 SAALTILVECWDGHLTPPEVASLADRASRARDSNMVRAAAELALSCLPHAHALNPNEIQR
Chlorocebus_sabaeus          SAALTILVECWDGHLTPPEVASLADRASRARDSNMVRAAAELALSCLPHAHALNPNEIQR
Homo_sapiens                 SAALTILVECWDGHLTPPEVASLADRASRARDSNMVRAAAELALSCLPHAHALNPNEIQR
Pongo_abelii                 SAALTILVECWDGHLTPPEVASLADRASRARDSNMVRAAAELALSCLPHAHALNPNEIQR
Otolemur_garnetti            SAALTILVECWDGHLTPPEVASLADRASRARDSNMVRAAAELALSCLPHAHALNPNEIQR
                             901234567890123456789012345678901234567890123456789012345678
                           1320        3         4         5         6         7

                                                                                       *
Brachyteles_arachnoides      ALVQCKEQDNLMLEKACMAVEEAAKGGGVYPEVLFEVAHQWFWLYEQTAGGSSTVREGST
Callithrix_jacchus           ALVQCKEQDNLMLEKACMAVEEAAKGGGVYPEVLFEVAHQWFWLYEQTAGGSSTAREGAT
Aotus_nancymaae              ALVQCKEQDNLMLEKACMAVEEAAKGGGVYPEVLFEVAHQWFWLYEQTAGGSSTAREGAT
Cebus_capucinus_imitator     ALVQCKEQDNLMLEKACMAVEEAAKGGGVYPEVLFEVAHQWFWLYEQTAGGSSTAREVAT
Saimiri_boliviensis          ALVQCKEQDNLMLEKACMAVEEAAKGGGVYPEVLFEVAHQWFWLYEQTAGGSSTAREGAT
Macaca_nemestrina            ALVQCKEQDNLMLEKACMAVEEAAKGGGVYPEVLFEVAHQWFWLYEQTAGGSSTAREGAT
Papio_anubis                 ALVQCKEQDNLMLEKACMAVEEAAKGGGVYPEVLFEVAHQWFWLYEQTAGGSSTAREGAT
Chlorocebus_sabaeus          ALVQCKEQDNLMLEKACMAVEEAAKGGGVYPEVLFEVAHQWFWLYEQTAGGSSTAREGAT
Homo_sapiens                 ALVQCKEQDNLMLEKACMAVEEAAKGGGVYPEVLFEVAHQWFWLYEQTAGGSSTAREGAT
Pongo_abelii                 ALVQCKEQDNLMLEKACMAVEEAAKGGGVYPEVLFEVAHQWFWLYEQTAGGSSTAREGAT
Otolemur_garnetti            ALVQCKEQDNLMLEKACMAVEEAAKGGGVYPEVLFEVAHQWFWLYEQTAGGSSTAREGAT
                             901234567890123456789012345678901234567890123456789012345678
                           1380        9       1400        1         2         3

Brachyteles_arachnoides      SCSASGIRAAGEAGRGLTEGRGGPGTEPVTV-AAAAVTAAATVVPVISVGSSLYPGPGLG
Callithrix_jacchus           SCSASGIRAAGEAGRGLTEGRGGPGTEPVTV-AAAAVTAAATVVPVISVGSSLYPGPGLG
Aotus_nancymaae              SCSASGIRASGEAGRGLTEGRGGPGTEPVTV-AAAAVTAAATVVPVISVGSSLYPGPGLG
Cebus_capucinus_imitator     SCSASGIRAAGEAGRGLTEGRGGPGTEPVTV-AAAAVTAAATVVPVISVGSSLYPGPGLG
Saimiri_boliviensis          GCSASGIRAAGEAGRGLTEGRGGPGTEPVTV-AAAAVTAAATVVPVISVGSSLYPGPGLG
Macaca_nemestrina            SCSASGIRVAGEAGRGMPEGRGGPGTEPVTV-AAAAVTAAATVVPVISVGSSLYPGPGLG
Papio_anubis                 SCSASGIRAAGEAGRGMPEGRGGPGTEPVTV-AAAAVTAAATVVPVISVGSSLYPGPGLG
Chlorocebus_sabaeus          SCSASGIRAAGEAGRGMPEGRGGPGTEPVTV-AAAAVTAAATVVPVISVGSSLYPGPGLG
Homo_sapiens                 SCSASGIRAGGEAGRGMPEGRGGPGTEPVTV-AAAAVTAAATVVPVISVGSSLYPGPGLG
Pongo_abelii                 SCSASGIRAAGEAGRGMPEGRGAPGTEPVTV-AAAAVTAAATVVPVISVGSSLYPGPGLG
Otolemur_garnetti            SCSASGIRAAGEAGRGLPEGRGGPGTEPVTVAAAAAVTAAATVVPVISVGSSLYPGPGLG
                             9012345678901234567890123456789-0123456789012345678901234567
                           1440        5         6          7         8         9
```

```
Brachyteles_arachnoides    HGHSPGLHPYTALQPHLPCSPQYLTHPAHPAHPMPHMPRPAVFPVPSSAYPQGVHPAFLG
Callithrix_jacchus         HGHSPGLHPYTALQPHLPCSPQYLTHPAHPAHPMPHMPRPAVFPVPSSAYPQGVHPAFLG
Aotus_nancymaae            HGHSPGLHPYTALQPHLPCSPQYLTHPAHPAHPMPHMPRPAVFPVPSSAYPQGVHPAFLG
Cebus_capucinus_imitator   HGHSPGLHPYTALQPHLPCSPQYLTHPAHPTHPMPHMPRPAVFPVPSSAYPQGVHPAFLG
Saimiri_boliviensis        HGHSPGLHPYTALQPHLPCSPQYLTHPAHPAHPMPHMPRPAVFPVPSSAYPQGVHPAFLG
Macaca_nemestrina           HGHSPGLHPYTALQPHLPCSPQYLTHPAHPAHPMPHMPRPAVFPVPSSAYPQGVHPAFLG
Papio_anubis               HGHSPGLHPYTALQPHLPCSPQYLTHPAHPAHPMPHMPRPAVFPVPSSAYPQGVHPAFLG
Chlorocebus_sabaeus        HGHSPGLHPYTALQPHLPCSPQYLTHPAHPAHPMPHMPRPAVFPVPSSAYPQGVHPAFLG
Homo_sapiens               HGHSPGLHPYTALQPHLPCSPQYLTHPAHPAHPMPHMPRPAVFPVPSSAYPQGVHPAFLG
Pongo_abelii               HGHSPGLHPYTALQPHLPCSPQYLTHPAHPAHPMPHMPRPAVFPVPSSAYPQGVHPAFLG
Otolemur_garnetti          HGHSPGLHPYTALQPHLPCSPQYLTHPAHPAHPMPHMPRPAVFPVPSSAYPQGVHPAFLG
                           890123456789012345678901234567890123456789012345678901234567
                          1500         1         2         3         4         5
```

```
Brachyteles_arachnoides    AQYPYSVTPPSLAATAVSFPVPSMAPITVHPYHTEPGLPLPTSVALSSVHPASTFPAIQG
Callithrix_jacchus         AQYPYSVTPPSLAATAVSFPVPSMAPITVHPYHTEPGLPLPTSVALSSVHPASTFPAIQG
Aotus_nancymaae            AQYPYSVTPPSLAATAVSFPVPSMAPITVHPYHTEPGLPLPTSVALSSVHPASTFPAIQG
Cebus_capucinus_imitator   AQYPYSVTPPSLAATAVSFPVPSMAPITVHPYHTEPGLPLPTSVALSSVHPASTFPAIQG
Saimiri_boliviensis        AQYPYSVTPPSLAATAVSFPVPSMAPITVHPYHTEPGLPLPTSVALSSVHPASTFPAIQG
Macaca_nemestrina          AQYPYSVTPPSLAATAVSFPVPSMAPITVHPYHTEPGLPLPTSVALSSVHPASTFPAIQG
Papio_anubis               AQYPYSVTPPSLAATAVSFPVPSMAPITVHPYHTEPGLPLPTSVALSSVHPASTFPAIQG
Chlorocebus_sabaeus        AQYPYSVTPPSLAATAVSFPVPSMAPITVHPYHTEPGLPLPTSVALSSVHPASTFPAIQG
Homo_sapiens               AQYPYSVTPPSLAATAVSFPVPSMAPITVHPYHTEPGLPLPTSVALSSVHPASTFPAIQG
Pongo_abelii               AQYPYSVTPPSLAATAVSFPVPSMAPITVHPYHTEPGLPLPTSVALSSVHPASTFPAIQG
Otolemur_garnetti          AQYPYSVTPPSLAATAVSFPVPSMAPITVHPYHTEPGLPLPTSVALSSVHPASTFPAIQG
                           890123456789012345678901234567890123456789012345678901234567
                          1560         7         8         9      1600         1
```

```
                                                          *
Brachyteles_arachnoides    ASLPALTTQPSPLVSGGFPPPEEETHSQPVSPHSLHHLHAAYRVGMLALEMLGRRAHNDH
Callithrix_jacchus         ASLPALTTQPSPLVSGGFPPPEEETHSQPVNPHSLHHLHAAYRVGMLALEMLGRRAHNDH
Aotus_nancymaae            ASLPALTTQPSPLVSGGFPPPEEETHSQPVSPHSLHHLHAAYRVGMLALEMLGRRAHNDH
Cebus_capucinus_imitator   ASLPALTTQPSPLVSGGFPPPEEETHSQPVNPHSLHHLHAAYRVGMLALEMLGRRAHNDH
Saimiri_boliviensis        ASLPALTTQPSPLVSGGFPPPEEETHSQPVNPHSLHHLHAAYRVGMLALEMLGRRAHNDH
Macaca_nemestrina          ASLPALTTQPSPLVSGGFPPPEEETHSQPVSPHSLHHLHAAYRVGMLALEMLGRRAHNDH
Papio_anubis               ASLPALTTQPSPLVSGGFPPPEEETHSQPVSPHSLHHLHAAYRVGMLALEMLGRRAHNDH
Chlorocebus_sabaeus        ASLPALTTQPSPLVSGGFPPPEEETHSQPVSPHSLHHLHAAYRVGMLALEMLGRRAHNDH
Homo_sapiens               ASLPALTTQPSPLVSGGFPPPEEETHSQPVNPHSLHHLHAAYRVGMLALEMLGRRAHNDH
Pongo_abelii               ASLPALTTQPSPLVSGGFPPPEEETHSQPVNPHSLHHLHAAYRVGMLALEMLGRRAHNDH
Otolemur_garnetti          ASLPALTTQPSPLVSGGFPPPEEETHSQPVNPHSLHHLHAAYRVGMLALEMLGRRAHNDH
                           890123456789012345678901234567890123456789012345678901234567
                          1620         3         4         5         6         7
```

```
Brachyteles_arachnoides    PNNFSRSPPYTDDVKWLLGLAAKLG
Callithrix_jacchus         PNNFSRSPPYTDDVKWLLGLAAKLG
Aotus_nancymaae            PNNFSRSPPYTDDVKWLLGLAAKLG
Cebus_capucinus_imitator   PNNFSRSPPYTDDVKWLLGLAAKLG
Saimiri_boliviensis        PNNFSRSPPYTDDVKWLLGLAAKLG
Macaca_nemestrina          PNNFSRSPPYTDDVKWLLGLAAKLG
Papio_anubis               PNNFSRSPPYTDDVKWLLGLAAKLG
Chlorocebus_sabaeus        PNNFSRSPPYTDDVKWLLGLAAKLG
Homo_sapiens               PNNFSRSPPYTDDVKWLLGLAAKLG
Pongo_abelii               PNNFSRSPPYTDDVKWLLGLAAKLG
Otolemur_garnetti          PNNFSRSPPYTDDVKWLLGLAAKLG
                           8901234567890123456789012
                          1680         9      1700
```
