# Supplementary material for: Transcriptome of the Southern Muriqui Brachyteles arachnoides (Primates:Platyrrhini), a Critically Endangered New World Monkey: Evidence of Adaptive Evolution
Source: Front Genet. 2020 Jul 31;11:831. doi: 10.3389/fgene.2020.00831 (PMC7412869; doi:10.3389/fgene.2020.00831)

**Supplementary Figure 4: Maximum likelihood trees of positively selected genes.** Trees were reconstructed with the *Brachyteles arachnoides* coding sequencing aligned with orthologous primate sequences. Nodes numbers represent bootstrap support calculated using 1000 replicates. Branch lengths correspond to nucleotide substitutions rates.

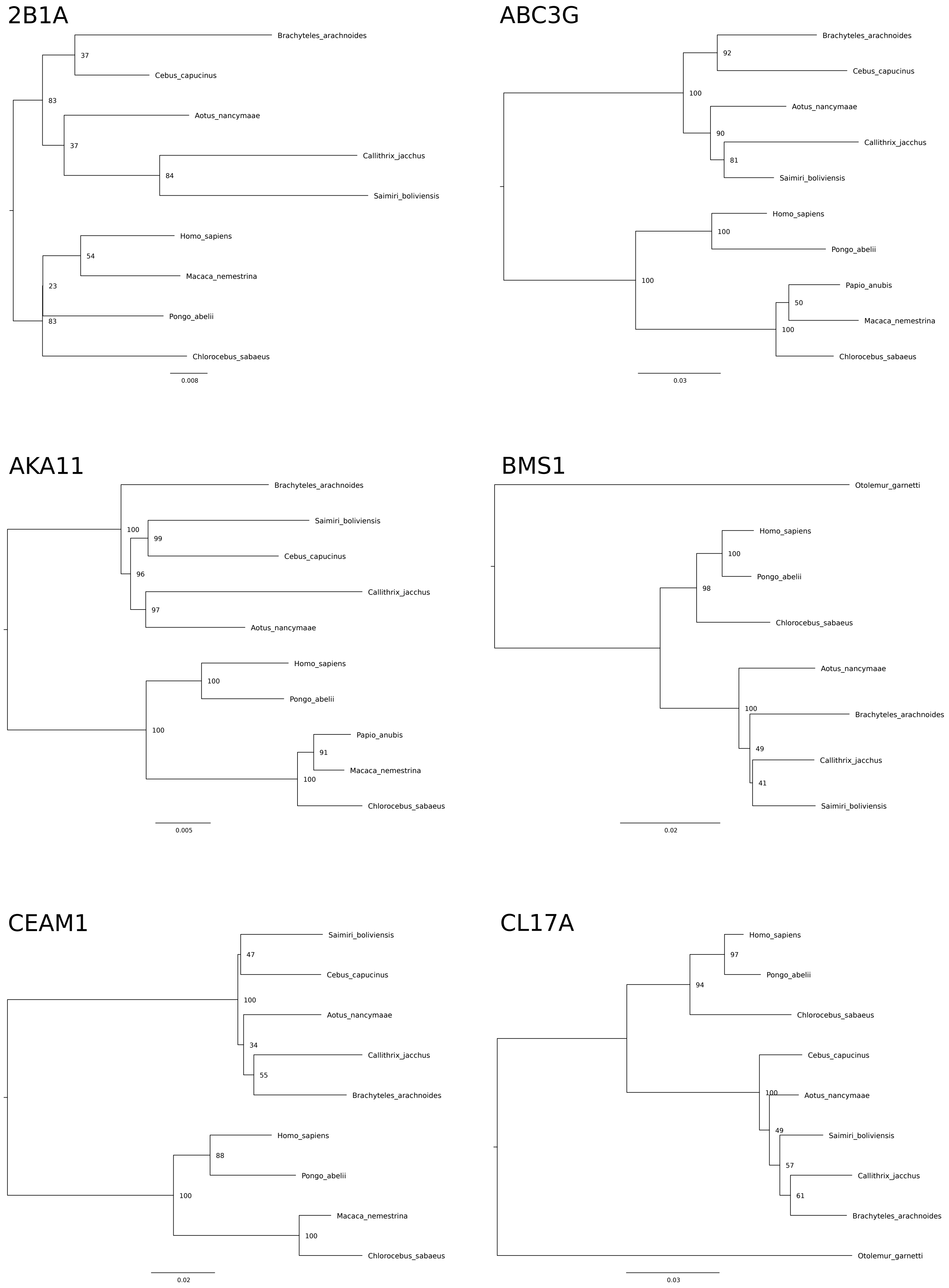
2B1A
Brachyteles_arachnoides
37
Cebus_capucinus
83
Aotus_nancymaae
37
Callithrix_jacchus
84
Saimiri_boliviensis
Homo_sapiens
54
Macaca_nemestrina
23
Pongo_abelii
83
Chlorocebus_sabaeus
0.008
ABC3G
Brachyteles_arachnoides
92
Cebus_capucinus
100
Aotus_nancymaae
90
Callithrix_jacchus
81
Saimiri_boliviensis
Homo_sapiens
100
Pongo_abelii
100
Papio_anubis
50
Macaca_nemestrina
100
Chlorocebus_sabaeus
0.03
AKA11
Brachyteles_arachnoides
100
Saimiri_boliviensis
99
Cebus_capucinus
96
Callithrix_jacchus
97
Aotus_nancymaae
Homo_sapiens
100
Pongo_abelii
100
Papio_anubis
91
Macaca_nemestrina
100
Chlorocebus_sabaeus
0.005
BMS1
Otolemur_garnetti
Homo_sapiens
100
Pongo_abelii
98
Chlorocebus_sabaeus
Aotus_nancymaae
100
Brachyteles_arachnoides
49
Callithrix_jacchus
41
Saimiri_boliviensis
0.02
CEAM1
Saimiri_boliviensis
47
Cebus_capucinus
100
Aotus_nancymaae
34
Callithrix_jacchus
55
Brachyteles_arachnoides
Homo_sapiens
88
Pongo_abelii
100
Macaca_nemestrina
100
Chlorocebus_sabaeus
0.02
CL17A
Homo_sapiens
97
Pongo_abelii
94
Chlorocebus_sabaeus
Cebus_capucinus
100
Aotus_nancymaae
49
Saimiri_boliviensis
57
Callithrix_jacchus
61
Brachyteles_arachnoides
Otolemur_garnetti
0.03

DDX52

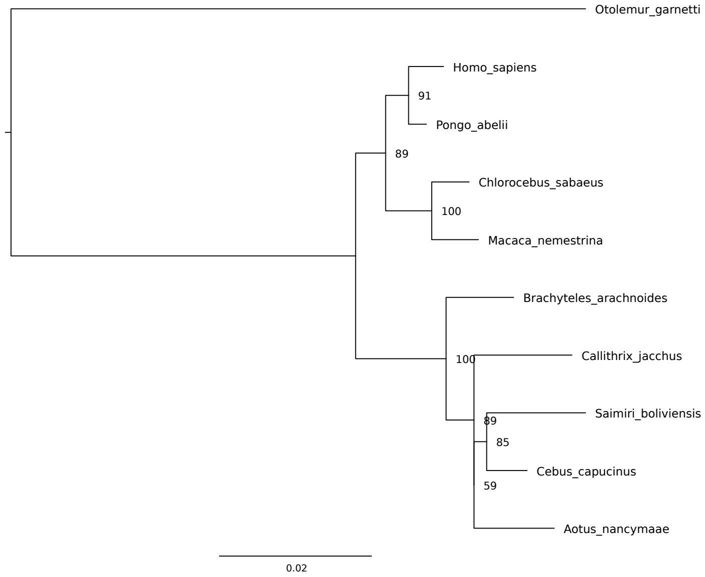


DNJC4

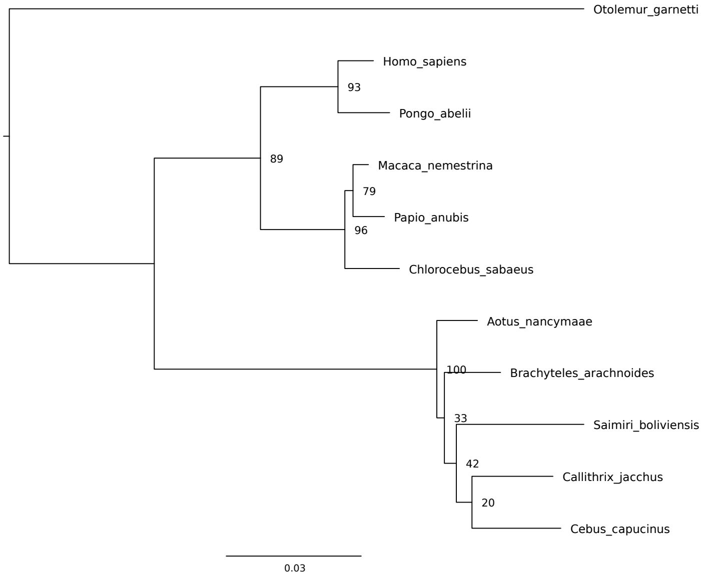


ECM1

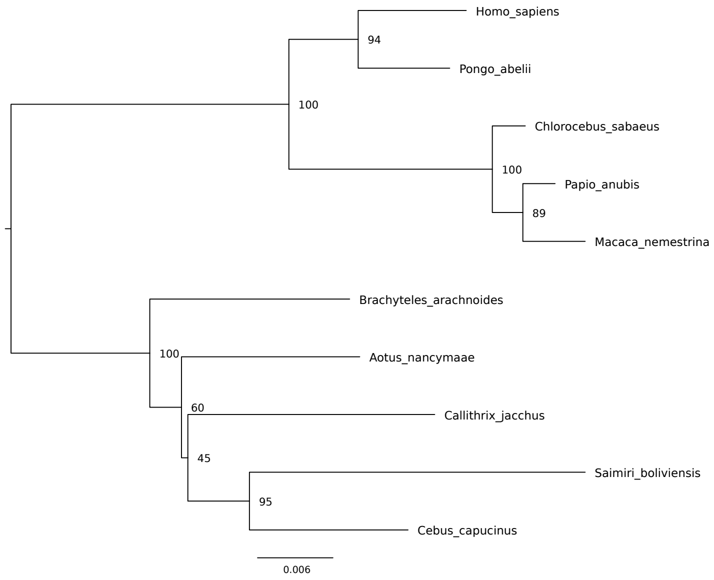


FLVC2

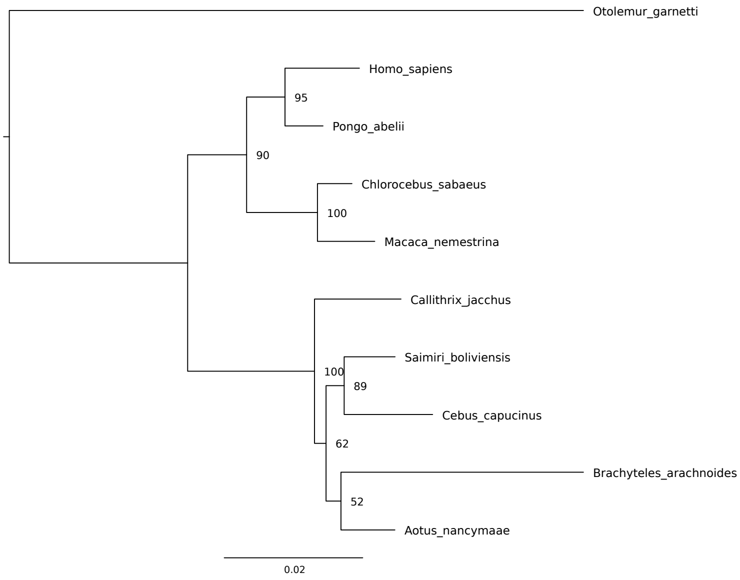


GRAB

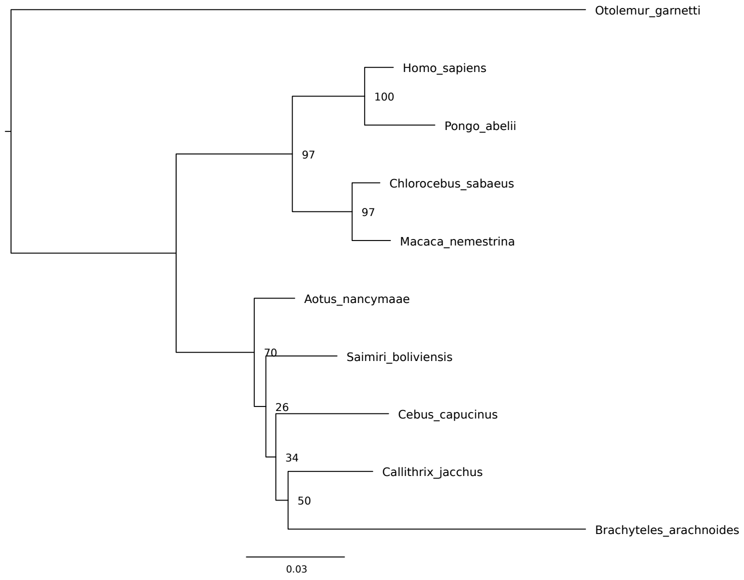


ITAM

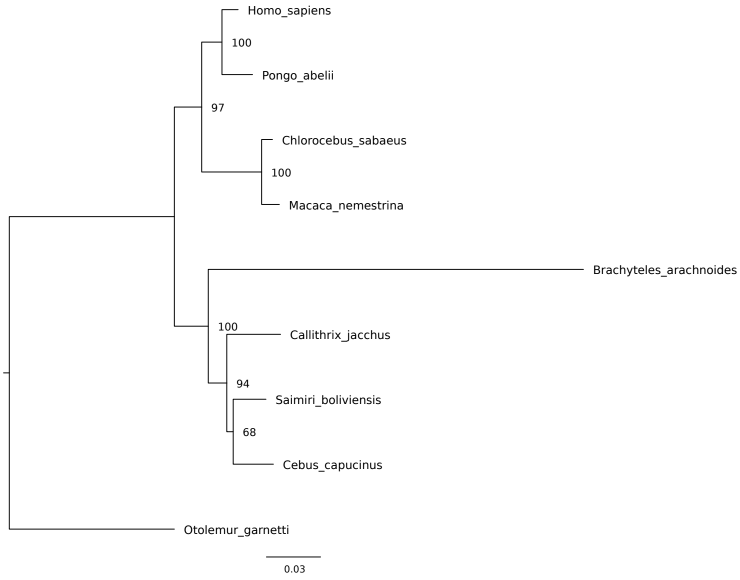

MEF2D

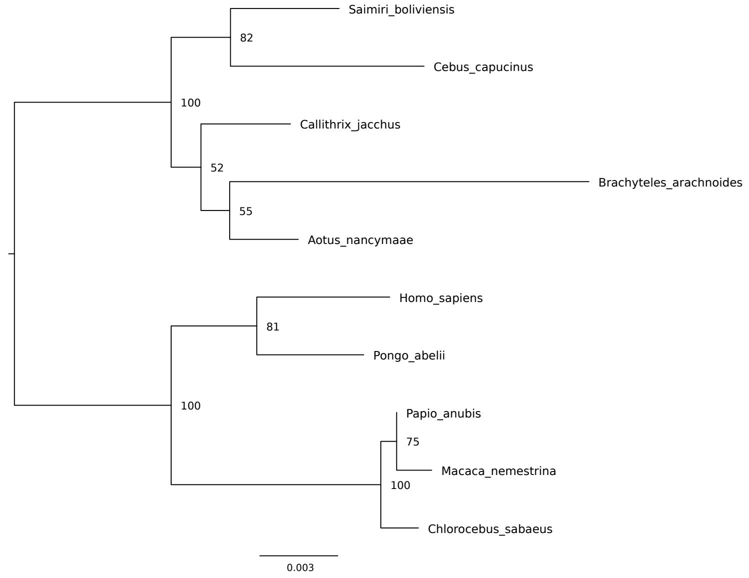


MINY2

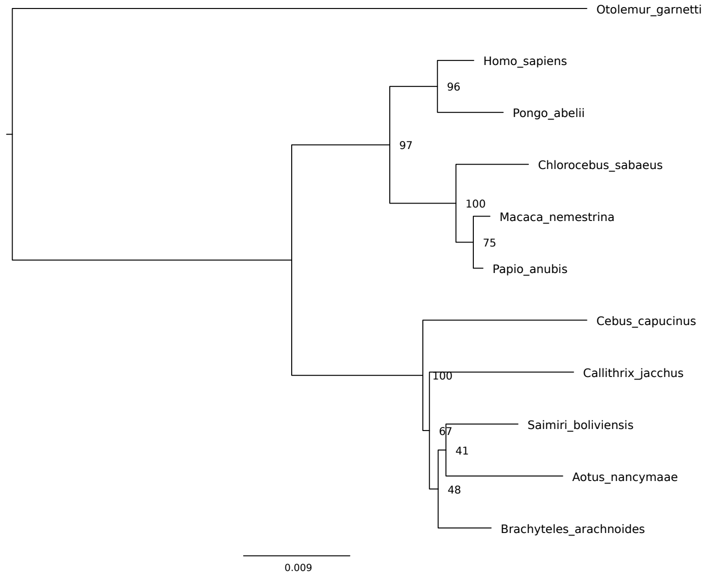


MUCEN

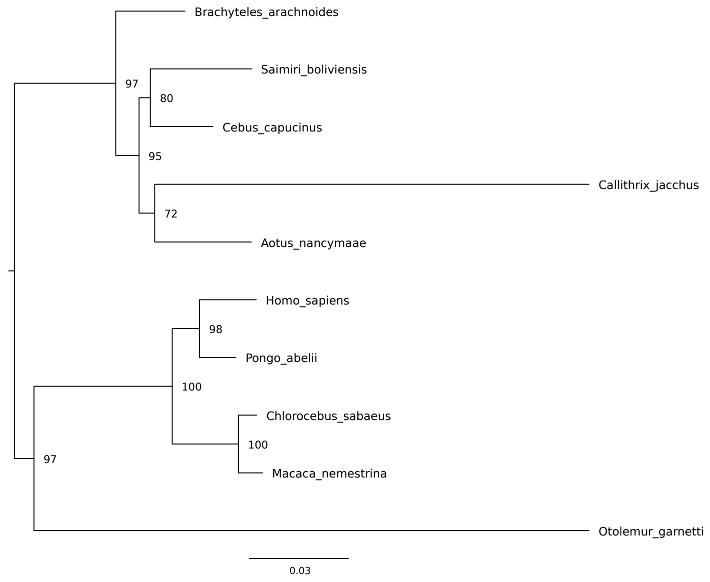


OAS2

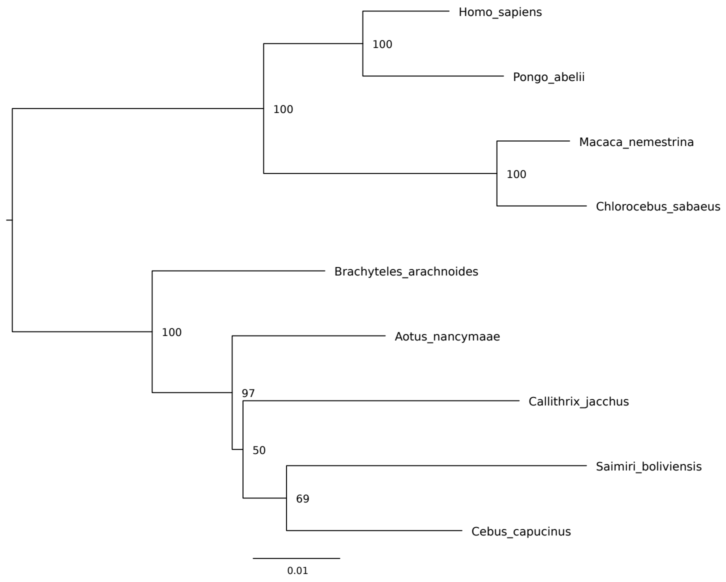


PPE1

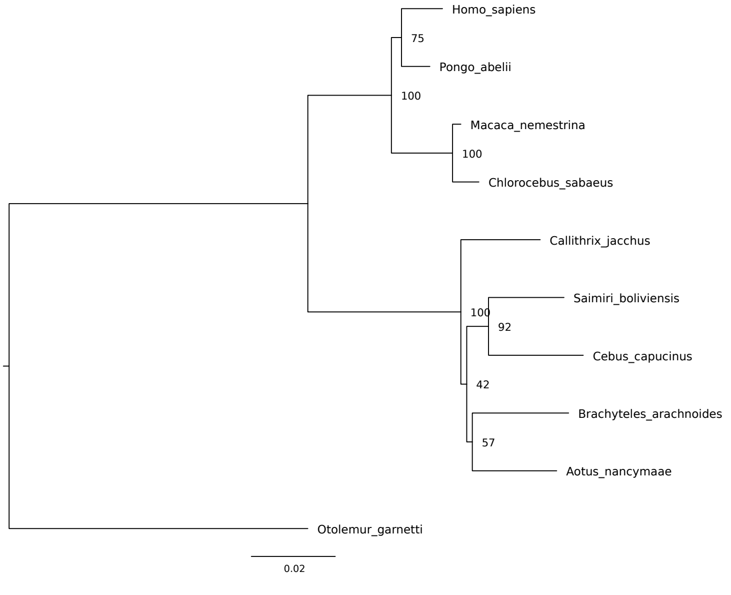


PRPS1

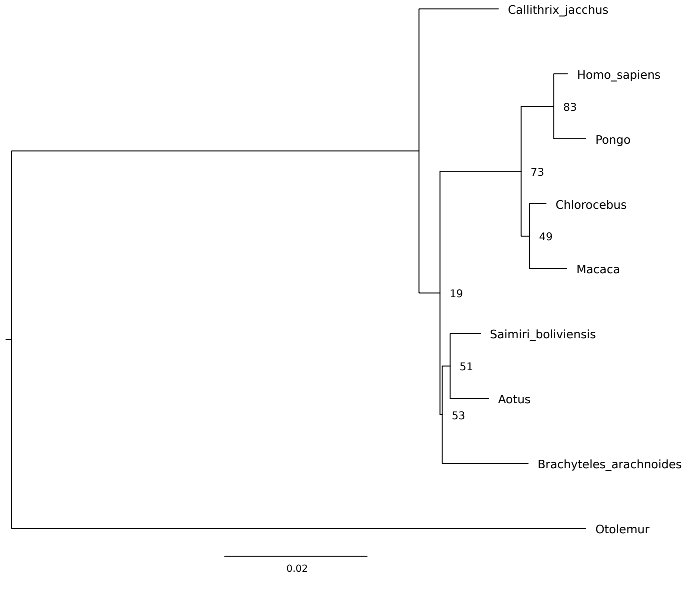

# RABE2

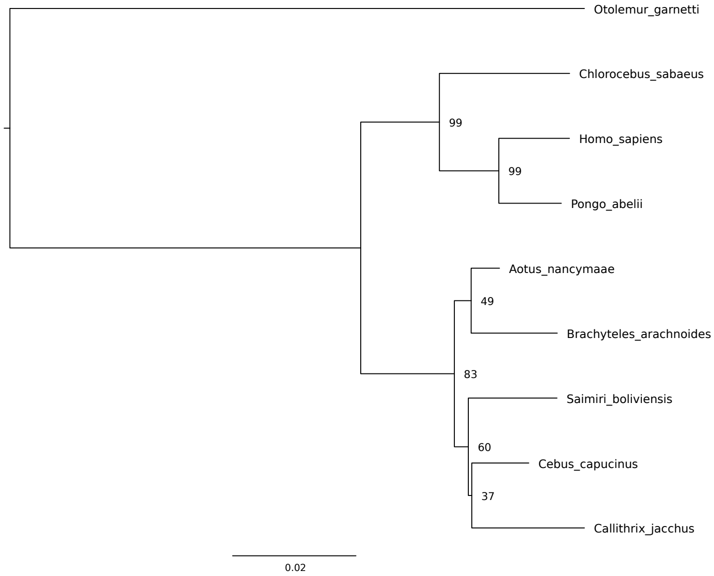


# RT14

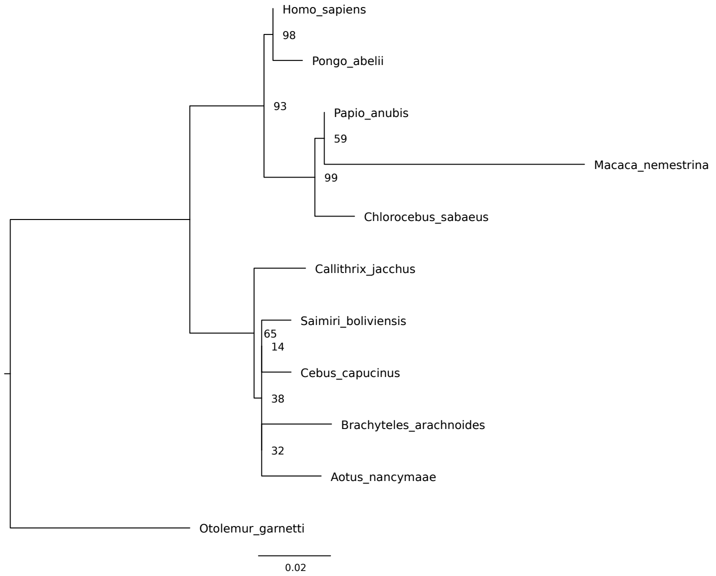


# S15A2

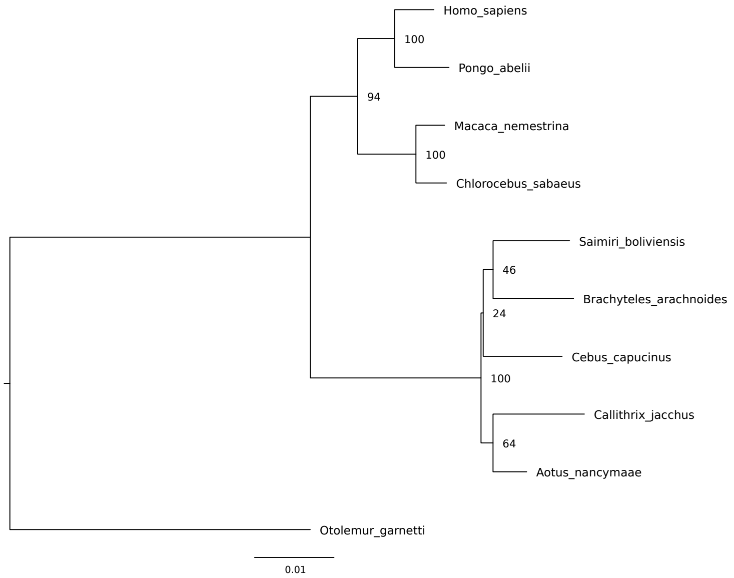


# S39AD

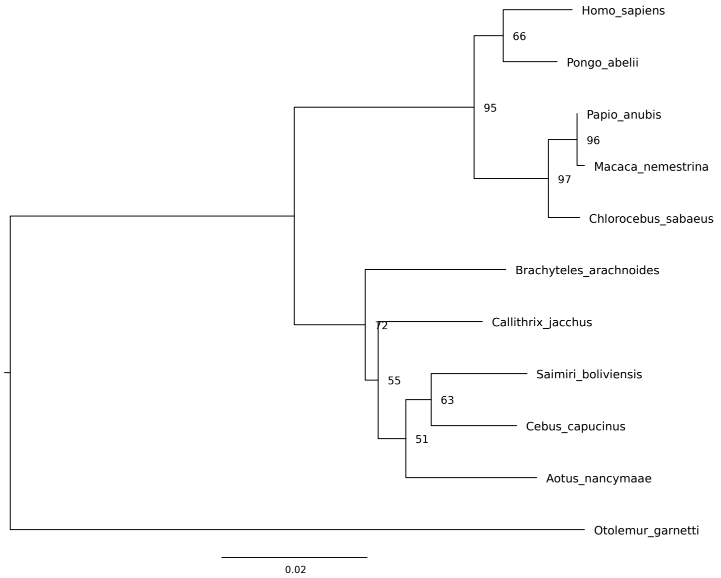


# SOAT1

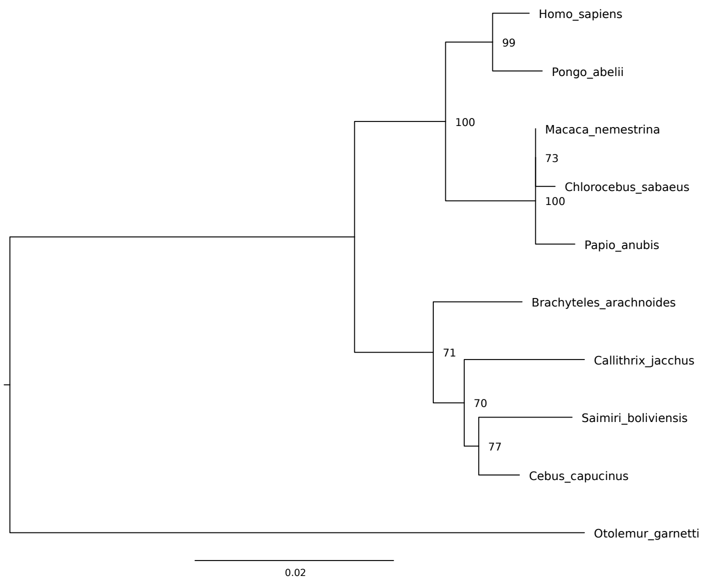


# SPIT1

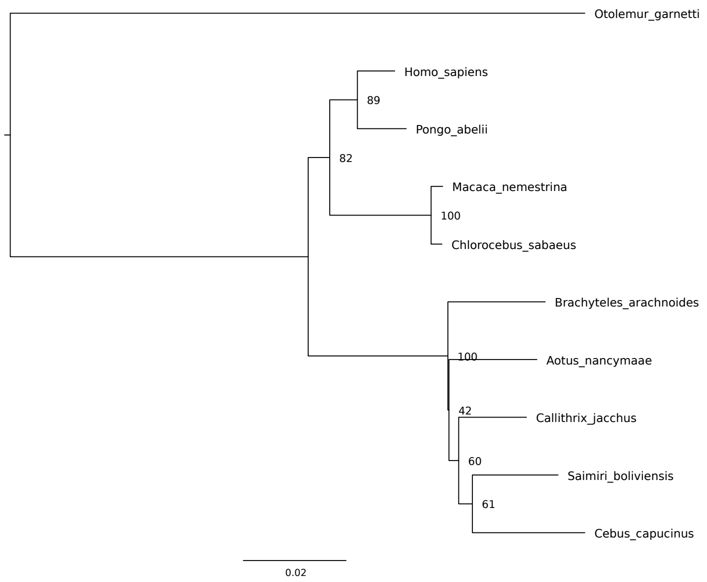

# TAF9B

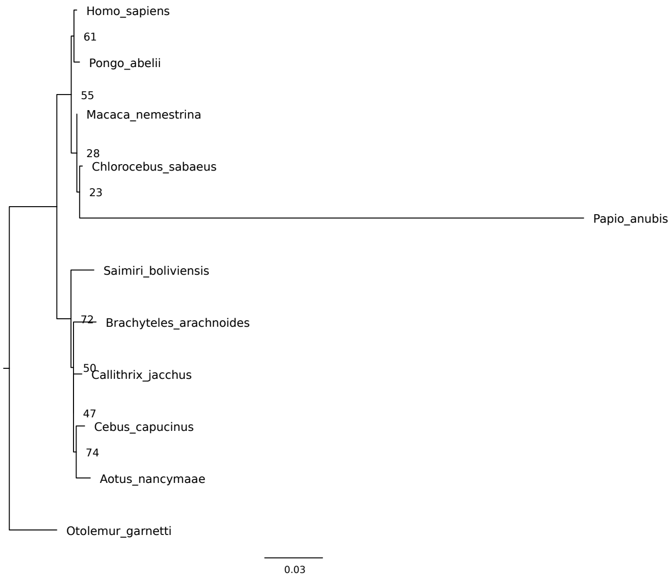


# UBX2B

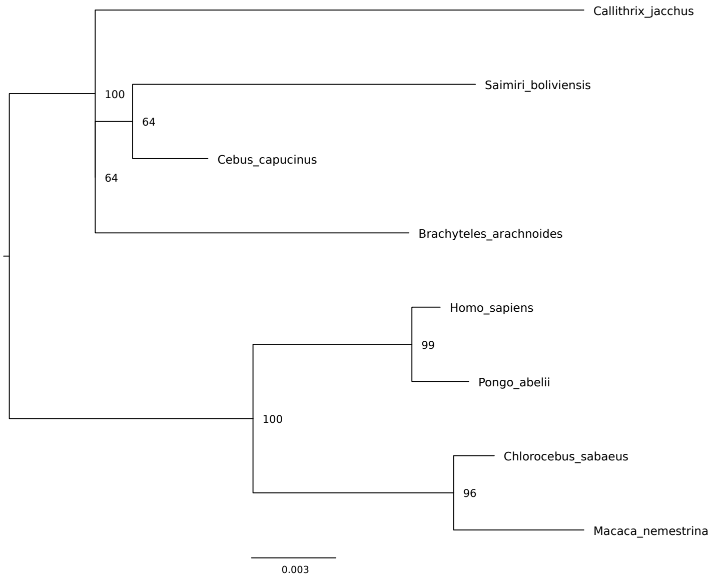


# UH1BL

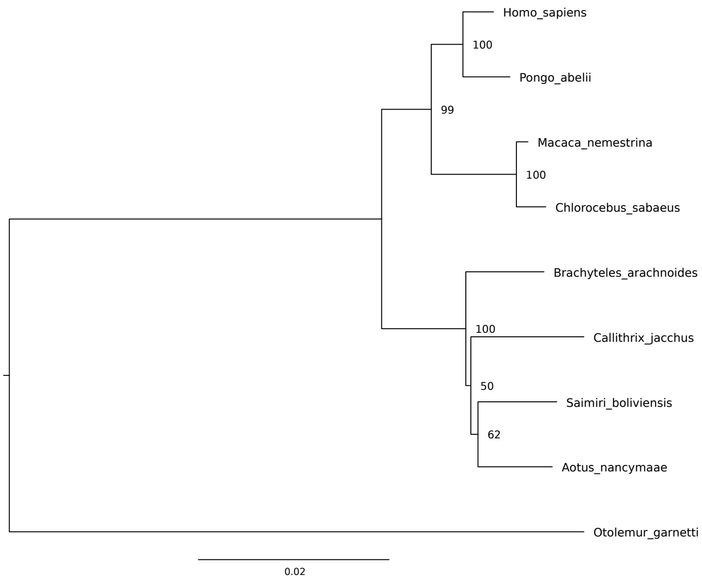


# SPIT1

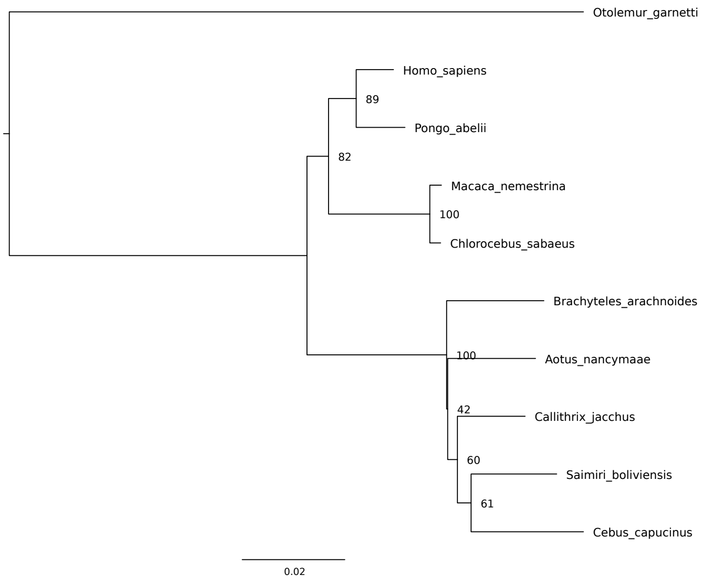


# ZBT44

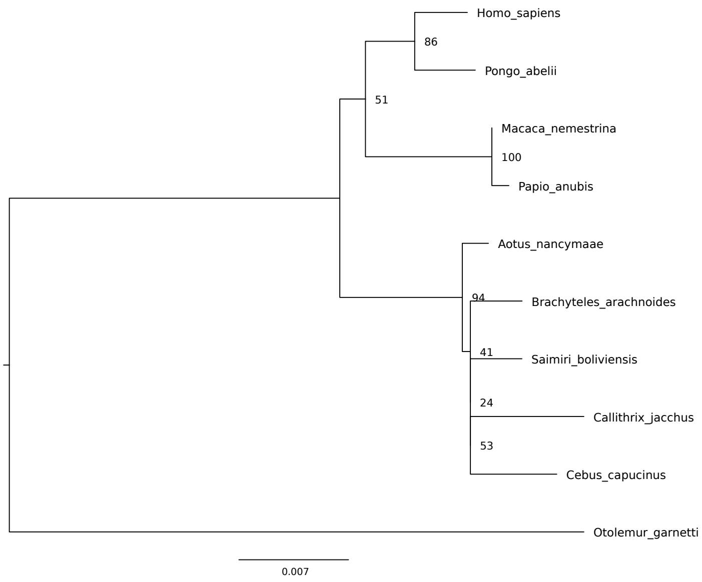


# VAPB

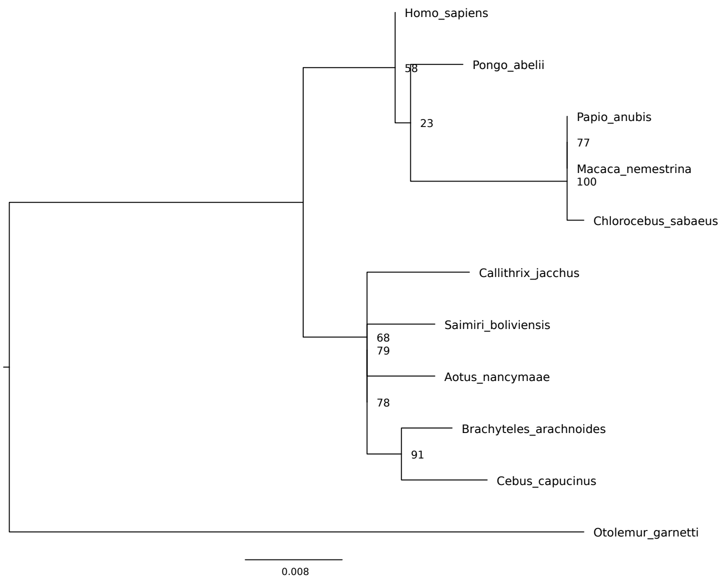

Supplement: Supplementary file 4 [file Image_4.PDF]
