# Supplementary material for: Transcriptome of the Southern Muriqui Brachyteles arachnoides (Primates:Platyrrhini), a Critically Endangered New World Monkey: Evidence of Adaptive Evolution
Source: Front Genet. 2020 Jul 31;11:831. doi: 10.3389/fgene.2020.00831 (PMC7412869; doi:10.3389/fgene.2020.00831)
Supplement: Supplementary file 5 [file Image_5.pdf]

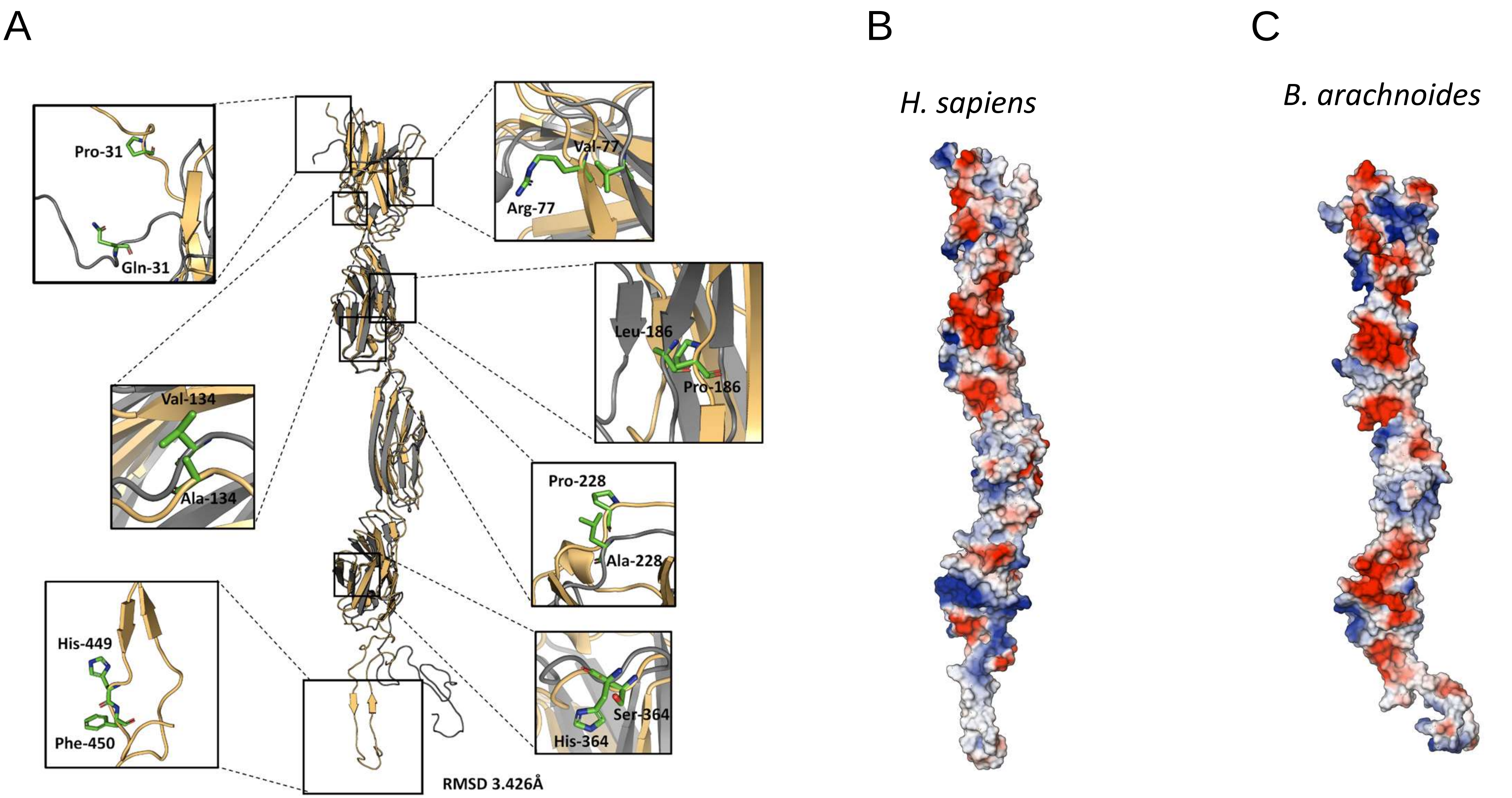

Supplementary Figure 5: 3D Model of CEACAM1. (A) Structural alignment between CEACAM1 *Homo sapiens* models (gold cartoon) and *Brachyteles arachnoides* (grey cartoon). Insets show residues (in sticks) under positive selection and its reference in human sequence. (B) and (C) Electrostatic profile of *H. sapiens* and *B. arachnoides* CEACAM1, respectively. The Bluest zones on protein surface represent values +75.093 KT/ec. The reddest zones represent values -75.093 KT/ec of the electrostatic properties. White regions mean zero values of the electrostatic potential.
