# Supplementary material for: Transcriptome of the Southern Muriqui Brachyteles arachnoides (Primates:Platyrrhini), a Critically Endangered New World Monkey: Evidence of Adaptive Evolution
Source: Front Genet. 2020 Jul 31;11:831. doi: 10.3389/fgene.2020.00831 (PMC7412869; doi:10.3389/fgene.2020.00831)
Supplement: Supplementary file 6 [file Image_6.pdf]

A

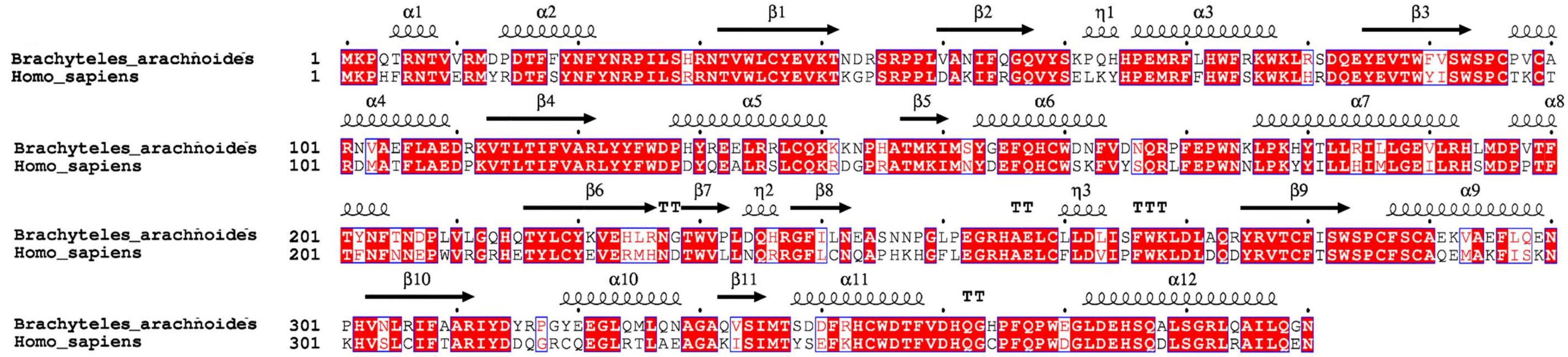


B

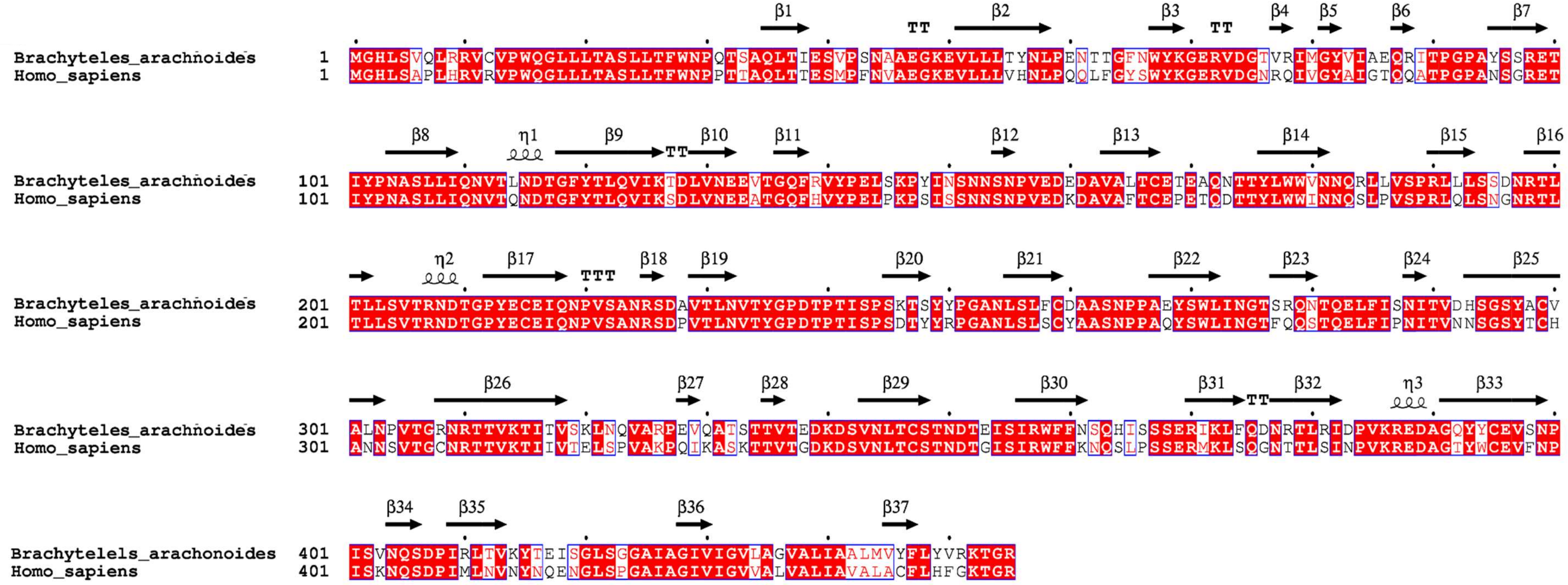


**Supplementary Figure 6: Analysis of sequences similarities in comparison with secondary structure information of homology models rendered by ESPript 3.0. (A) The sequences of APOBEC3G. (B) The sequences of CEACAM1. The proteins secondary structure (alfa helix and beta sheet) are represented on top of sequences.**
